# Laboratory Mouse Models for the Human Genome-Wide Associations

**DOI:** 10.1371/journal.pone.0013782

**Published:** 2010-11-01

**Authors:** Georgios D. Kitsios, Navdeep Tangri, Peter J. Castaldi, John P. A. Ioannidis

**Affiliations:** 1 Institute for Clinical Research and Health Policy Studies, Tufts Medical Center, Boston, Massachusetts, United States of America; 2 Tufts University School of Medicine, Boston, Massachusetts, United States of America; 3 Department of Hygiene and Epidemiology, University of Ioannina School of Medicine and Biomedical Research Institute, Foundation for Research and Technology-Hellas, Ioannina, Greece; 4 Tufts Clinical and Translational Science Institute, Tufts Medical Center, Boston, Massachusetts, United States of America; 5 Department of Medicine, Center for Genetic Epidemiology and Modeling, Tufts Medical Center, Tufts University School of Medicine, Boston, Massachusetts, United States of America; 6 Division of Nephrology, Tufts Medical Center, Boston, Massachusetts, United States of America; 7 Department of Epidemiology, Harvard School of Public Health, Harvard University, Boston, Massachusetts, United States of America; 8 Stanford Prevention Research Center, Stanford University School of Medicine, Stanford, California, United States of America; Ohio State University Medical Center, United States of America

## Abstract

The agnostic screening performed by genome-wide association studies (GWAS) has uncovered associations for previously unsuspected genes. Knowledge about the functional role of these genes is crucial and laboratory mouse models can provide such information. Here, we describe a systematic juxtaposition of human GWAS-discovered loci versus mouse models in order to appreciate the availability of mouse models data, to gain biological insights for the role of these genes and to explore the extent of concordance between these two lines of evidence. We perused publicly available data (NHGRI database for human associations and Mouse Genome Informatics database for mouse models) and employed two alternative approaches for cross-species comparisons, phenotype- and gene-centric. A total of 293 single gene-phenotype human associations (262 unique genes and 69 unique phenotypes) were evaluated. In the phenotype-centric approach, we identified all mouse models and related ortholog genes for the 51 human phenotypes with a comparable phenotype in mice. A total of 27 ortholog genes were found to be associated with the same phenotype in humans and mice, a concordance that was significantly larger than expected by chance (p<0.001). In the gene-centric approach, we were able to locate at least 1 knockout model for 60% of the 262 genes. The knockouts for 35% of these orthologs displayed pre- or post-natal lethality. For the remaining non-lethal orthologs, the same organ system was involved in mice and humans in 71% of the cases (p<0.001). Our project highlights the wealth of available information from mouse models for human GWAS, catalogues extensive information on plausible physiologic implications for many genes, provides hypothesis-generating findings for additional GWAS analyses and documents that the concordance between human and mouse genetic association is larger than expected by chance and can be informative.

## Introduction

Genome-wide association studies (GWAS) have led to the discovery of hundreds of associations between genetic loci and complex human diseases or traits [1]. These associations have very robust statistical support, but they emerge in an agnostic fashion, i.e. all variants are tested without considering any specific biological rationale or prior biological evidence for the functional importance of specific variants [2]. Discovered associations generally represent tagging markers rather than the culprit functional genetic variation. Therefore, once a marker is discovered, one needs to identify what functional variation it represents and what is the underlying biological mechanism of the observed association [3]. Such tasks are not easy; functional insights can be derived from new biological experiments and also, by integration of other lines of existing biological evidence.

One of the most extensive and readily available sources of such evidence is provided by mouse model organisms. The mouse has a fully sequenced genome, almost all (99%) mouse genes have orthologs in humans, and multiple tools are available for manipulating its genome, allowing genes to be altered efficiently and precisely. Knowledge gained from mouse models can facilitate biomedical discoveries, by uncovering the functional role of genes and enabling cross-species comparisons. Currently, the Mouse Genome Informatics (MGI) database represents the most comprehensive public resource providing integrated access to genetic and phenotypic information for thousands of curated mouse mutations [4].

Some investigators have performed focused comparisons between gene-disease associations emerging from GWAS and the mouse phenotypes observed when the respective gene loci are knocked out [5], [6]. However, the availability of comprehensive databases of both mouse models and human genome-wide associations allows a systematic effort of cross-comparisons between these two sources of evidence and may provide some mechanistic insights on the agnostically-derived gene-disease associations in humans. Here, we performed such a systematic juxtaposition of human GWAS-discovered loci versus mouse models data. We aimed to evaluate the availability of mouse models for human GWAS-discovered loci; record the range of genetic and phenotypic information for these models; and explore the extent of concordance between mouse models and human genome-wide associations.

## Materials and Methods

### Genome-wide associations

We used the NHGRI catalogue of GWAS, a comprehensive database of all published GWAS [7], [8]. In order to limit our focus to associations with robust statistical support, we extracted data on associations with p-values <10^−8^ (ref. [9]). We considered all associations listed in the catalogue as of June 5^th^, 2009. We excluded associations where the single nucleotide polymorphisms (SNPs) were located in gene deserts and no particular gene or set of genes was implicated by the authors of the GWAS. We merged entries from different GWAS where the same SNP or different SNPs from the same gene had been associated with the same phenotype. To avoid double-counting of findings for phenotypes with strong biological and clinical similarity, we also merged together similar phenotypes under a single entry. In this process, we assessed the studied phenotypes according to the Entity-Quality (EQ) methodology (affected entity (E) and how it is affected (Q)) [10]. Considering the entities affected (anatomical part, biological process, cellular component or molecular function) in the available phenotypes, we merged phenotypic entries where the same entity was affected. For example, for the entries “hypertension”, “systolic blood pressure”, “diastolic blood pressure”, and “blood pressure”, we considered the same entity –“blood pressure”– to be affected, irrespective of the exact manner that this entity was affected (increased or decreased blood pressure); thus all these entries were merged under the phenotype “blood pressure related phenotypes”. By merging such similar phenotypic entries, we obtained a final list of 69 included phenotypes (out of the initial 102 phenotypic entries), which are provided in Table S1.

Of the remaining, streamlined set of GWAS-discovered associations, we selected those where only one gene had been implicated and excluded those associations that mapped to loci with multiple potentially implicated genes. In this selection, we followed the arbitration of the GWAS authors and the curators of the NHGRI catalogue. When a single gene is listed, this does not mean that necessarily this gene is the culprit one, but the investigators of the GWAS and the NHGRI curators considered that the identified SNP is located in this specific gene and therefore this gene is more likely to be the culprit than neighbouring or distant contesters. For each one of the eligible genes, we recorded the investigated human phenotypes and the individual GWAS results, as provided by the NHGRI.

### Mouse models

All necessary information on mouse models was extracted from the MGI database (http://www.informatics.jax.org/) [4]: human and mouse ortholog genes (“orthologs”) were identified through the Mammalian Orthology section of MGI; mouse genotypic data for each phenotype of interest were extracted by manually searching the Mammalian Phenotype Browser and through automated searches of the MGI Data and Statistical Reports for Alleles and Phenotypes; mouse model and phenotypic data for all genes of interest were extracted through manual searches in the Genes and Markers section and through automated searches of the Biomart system and the MGI Batch Query module. The final searches were performed on April 22^nd^, 2010 using the 4.33 release of the MGI database.

**Figure 1:**
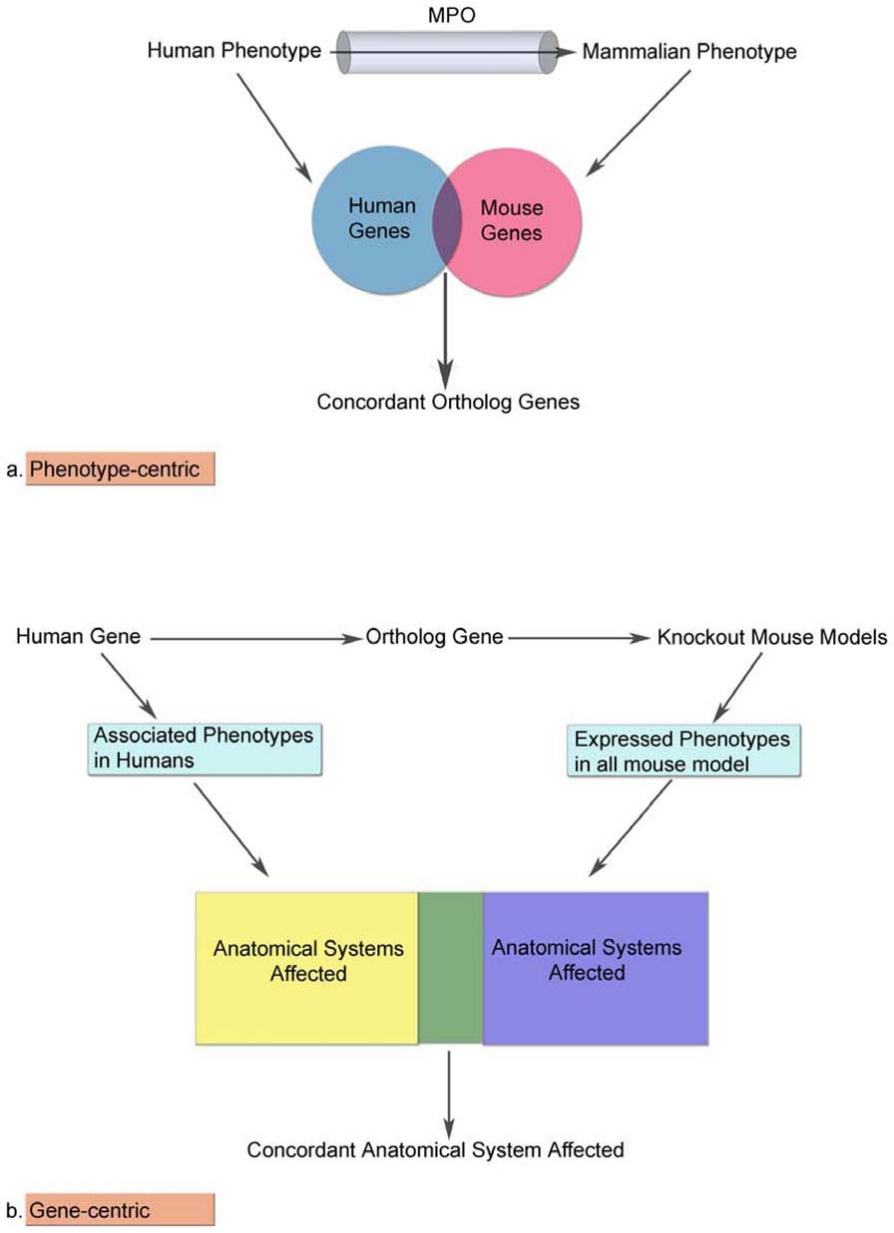
Flowchart of the phenotype-centric and the gene-centric approach. MPO: Mammalian Phenotype Ontology.

In order to search comprehensively for laboratory mouse models for all eligible human gene-disease associations, we applied two alternative and independent approaches: a phenotype-centric, where our search sample was defined by the human phenotypes studied in GWAS, and a gene-centric approach, where the search sample was formed by the GWAS-implicated genes (Figure 1). As described below, the information derived by these two approaches is different and complementary. The phenotype-centric approach is based on specific phenotypic definitions and utilizes all types of mouse genetic models, whereas the gene-centric approach focuses only on knockout mouse models and uses general phenotypic descriptors (i.e. at the level of affected organ systems).

#### Phenotype-centric approach

In this approach, we mapped the human phenotypes associated with GWAS-discovered loci to their corresponding mouse phenotypes, and we assembled a comprehensive list of mouse genes associated with these phenotypes. Then, we evaluated the extent of overlap between the mouse and human orthologs that have been associated with the same phenotype (Figure 1a).

Previous work has created hierarchical systems of human heritable phenotypes and has integrated phenotype ontologies across species, including humans and mice [10], [11], [12]. However, to our knowledge, the complex phenotypes considered in GWAS have not been standardized in an ontology vocabulary. Thus, we used the Mammalian Phenotype Ontology (MPO) to map the human phenotypes to corresponding mouse (mammalian) phenotypes [13], [14]. The MPO has a hierarchical structure (tree) from high-level, broadly descriptive terms to very low-level, highly specific terms, presented according to the anatomical systems affected in each condition. These systems (*“Anatomical Systems Affected by Phenotypes”*) comprise a high-level phenotype term list of organ systems (e.g., cardiovascular, digestive/alimentary) or syndromes (e.g., life span/aging) that MGI has phenotypes for (Table S2). In this process, we performed searches in the MPO with the Mammalian Phenotype Browser for the exact phenotypic terms used in the NHGRI database, supplemented by MeSH terms manually collected for each human phenotype. For each human phenotypic term searched, we examined the definitions of the retrieved mammalian phenotypes for biological equivalency to the human ones, based on clinical judgement and consensus among the investigators and focusing on the Entity component of the EQ methodology. Whenever a mammalian phenotype was considered equivalent to the human one, we recorded the Mammalian Phenotype (MP) accession numbers for the respective phenotype and for all lower-level phenotypes branching out from it (descendant nodes). We also exploited the information provided by these trees in order to identify which anatomical systems are affected by each phenotype and we considered the same anatomical systems to be affected in the corresponding human phenotypes. For a subset of phenotypes that could not be mapped to MPO based on the MeSH terms searches, we manually screened the Mammalian Phenotype Browser within the corresponding anatomic systems for each phenotype, in search of abnormal morphology or physiology directly related to the phenotype in question. For example, for Alzheimer's disease, we manually screened the nervous system phenotypes and identified two entries that corresponded to the landmark pathology lesions of the disease: amyloid beta deposits and neurofibrillary tangles.

In the next step, we performed systematic searches in the ‘Alleles and Phenotypes’ reports of the MGI Data and Statistical Reports for all mouse models (considering all types of mutations, apart from gene trapped markers that had been studied only in cell lines and not in living organisms) associated with each MP accession number in order to identify all mouse genes that have been associated with each particular MP. For phenotypes with descendant nodes, we also included the MP accession numbers of the descendant nodes in our searches. Finally, for each phenotype, we compared the associated human and mouse genes and we recorded all instances where the same orthologs were associated with the same phenotype in both species (“concordant orthologs”).

#### Gene-centric approach

With this second approach, our search started from the orthologs of the GWAS-derived human genes, for which we identified all knockout models and evaluated their phenotypic expression (Figure 1b). Meaningful comparisons of expressed phenotypes between human and mouse orthologs were enabled through the following arbitrations: first, we limited our analyses to knockout mouse models only; since the specific genetic variants discovered in GWAS are likely tagging markers and the causative mutations are commonly elusive, we focused only on unambiguous mutations (complete gene deletions in knockouts) in the mouse organisms. Furthermore, recognizing that the specific phenotypic manifestations of gene deletion would be expected to be qualitatively different from the phenotypic manifestations of common genetic variations studied in human GWAS, we limited our comparisons at the level of the anatomical system affected.

For each ortholog of the GWAS-derived human genes, we recorded the availability of knockout models, and we catalogued all available information on observed phenotypes in these mouse models. The observed knockout mouse phenotypes were categorized according to the anatomical system(s) affected, as described above. In cases where a knockout model displayed lethality, this was noted separately. For knockout models not expressing lethality, we explored instances of phenotypic concordance (at the level of affected anatomical system) between human gene associations and corresponding knockouts.

### Statistical analysis

For both the phenotype- and the gene-centric approach, we compared whether the observed concordance between human and mice data was significantly different from the expected concordance by chance. The expected concordance by chance was calculated by considering the marginal and grand totals of 2×2 tables with juxtaposed human and mice data.

In the phenotypic-centric approach, the expected concordance for a given phenotype X was calculated as: [(number of mouse genes associated with X) * (number of human genes associated with X)/(total number of ortholog pairs with available mouse models)]. The denominator (grand total) is approximated by the total number of orthologous genes that have been studied in a laboratory mouse model and thus have a chance to be associated with the same phenotype in humans and mice. According to MGI 4.33, the denominator was set to be equal to 12,526. Then, the expected concordances of all phenotypes were summed up and compared to the number of overall observed concordances.

In the gene-centric approach, comparisons of phenotypic concordance were performed at the level of the anatomical system affected. Consequently, the expected concordance for a given gene Y was calculated as: [(number of anatomical systems associated with Y in mice) * (number of anatomical systems associated with Y in humans)/(total number of anatomical systems affected in mice and humans]. The total number of anatomical systems equals 31. Then, the expected concordances of all genes were summed up and compared to the number of overall observed concordances using a chi-square test with 1 degree of freedom.

## Results

**Figure 2:**
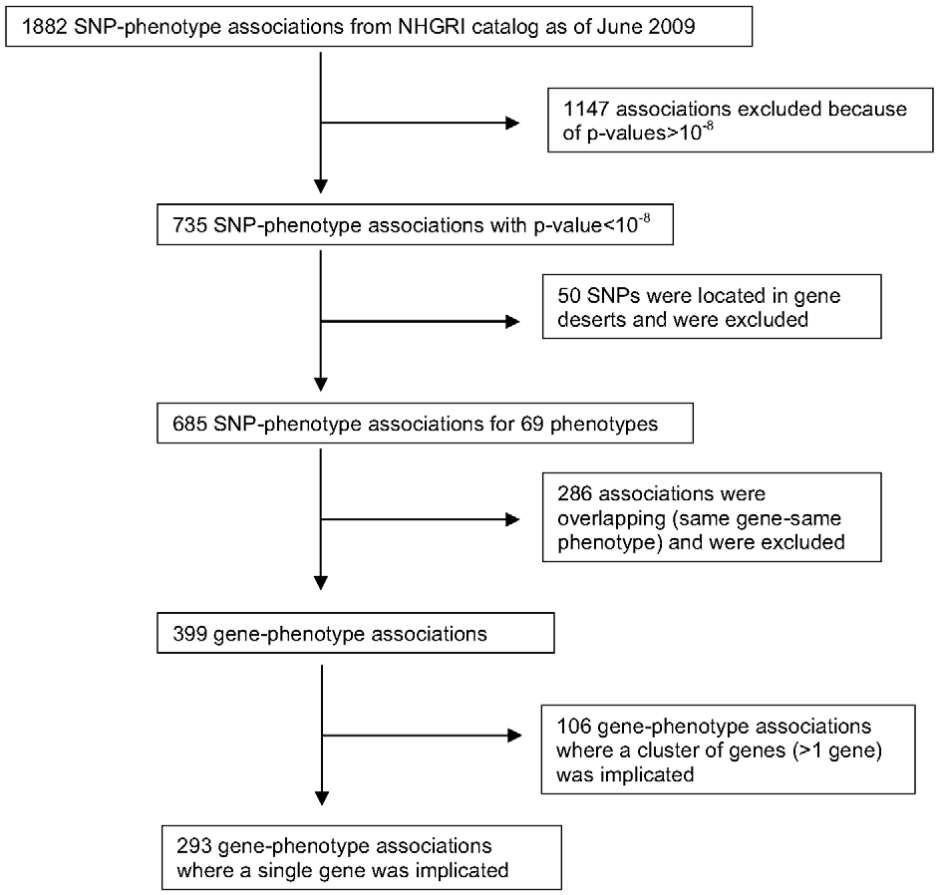
Flowchart of the selection process of GWAS-derived gene-phenotype associations.

A flowchart of the selection process of GWAS associations eligible for comparisons with knockout mice models is provided in Figure 2. Of the initial sample of 1882 SNP-phenotype associations catalogued in the NHGRI catalogue, 735 associations had robust statistical support (p-value<10^−8^). After excluding intergenic SNPs, overlapping entries and associations involving more than one gene, 293 gene-disease associations were eligible for analysis pertaining to 69 different phenotypes.

### Phenotype-centric approach

Of the 69 phenotypes investigated in humans, we reached consensus on a final list of 51 phenotypes that were considered to have a mammalian equivalent phenotype (Table S3). Forty-three of the 51 phenotypes were mapped directly to mammalian equivalents based on MeSH terms searches, whereas for eight phenotypes mapping was achieved after manual searches in the Mammalian Phenotype Browser and inferences drawn on related pathophysiology. The remaining 18 phenotypes that could not be mapped to MPO were excluded from further analyses. Twenty five of the 51 (49%) mammalian phenotypes included additional, descendant phenotypic nodes, whereas the remaining ones were lowest-level phenotypes (Tables S4 and S5). Nine mammalian phenotypes involved two anatomical systems and the remaining ones involved only one affected system.

Each mammalian phenotype has been associated with a median of 21 mouse models (interquartile range (IQR), 15–61 models), corresponding to a median of 17 different genes (IQR, 4–36) per phenotype. In the corresponding human phenotypes, a median of 3 genes (IQR, 1–6) per phenotype was implicated in GWAS.

When comparing the orthologs involved in the human and the mammalian phenotypes, 27 concordant orthologs were found in 10 phenotypes (Table S4). This total number of 27 concordances between human and mice was significantly larger (p<0.001) than the number of concordances expected by chance (expected n = 1.9). We also conducted a subgroup analysis of concordance for phenotypes stratified by type of mapping to MPO (43 phenotypes mapped by MeSH terms and eight phenotypes mapped by manual searches). Statistically significant concordance was detected for both comparisons (observed vs expected concordances  = 29 vs 1.8, p<0.001 and 5 vs 0.25, p<0.001, respectively).

Although human GWAS associations have been documented in agnostic experiments, the mouse models are typically constructed to test a specific hypothesis, which is usually based on various types of biological evidence. Consequently, the creation of certain mouse models may have been informed by human genetic associations that had already been recognized in the candidate gene era before the advent of GWAS. In order to control for this, we classified the human GWAS associations into novel ones and associations proposed by candidate-gene studies (Table S6), as previously described [15]. We then performed a sensitivity analysis by examining only the novel GWAS associations (Table S7). Concordance was now observed only for three phenotypes (inflammatory bowel disease, prostate cancer, obesity-related phenotypes) and a total of 3 genes (MST1, MSMB and SH2B1, respectively). These gene-phenotype associations were described by a total of 4 mouse models (MST1: 1 knockout; MSMB: 1 knock-in; SH2B1: 2 knockouts) [16], [17], [18], [19], all of which had been created before the publication of the GWAS that discovered a similar association in humans. The observed concordance (n = 3 genes) was only 2.5 times higher than the expected by chance (expected n = 1.17) and the difference was not nominally significant (p = 0.09). For candidate-gene study associations though, the observed concordance (n = 24 genes) was significantly larger (p<0.001) than expected by chance (expected n = 0.7).

### Gene-centric approach

Our human GWAS sample of 293 gene-disease associations involved a total of 262 unique genes, since 15 genes were associated with more than one phenotype. Orthologs were identified for 250 (95%) of them. We subsequently searched for knockout mouse models constructed for these orthologs and we were able to locate at least 1 knockout model for 150 of the 250 orthologs (60%); 73 of these orthologs had more than one knockout model available (range 2–11). Overall, 295 knockout models for the 150 orthologs were found in the MGI database, with variable types of gene deletion techniques, genetic backgrounds and phenotypic information for various allelic combinations (heterozygous, homozygous, conditional genotypes etc.). All available information on phenotypes was merged at the ortholog gene level to allow comparisons with humans. The entire range of phenotypic expression of each knocked out ortholog was catalogued (Table S8).

Thirty of the 31 anatomical systems of the MPO were affected in at least 1 knocked out ortholog. The most commonly affected anatomical systems were the immune system, the hematopoetic system, and homeostasis/metabolism, which were involved in more than 40% of the examined knocked out orthologs (Figure S1).

Fifty three of the 150 orthologs (35%) with knockout models displayed a lethal phenotype: 34 orthologs were associated with prenatal/perinatal lethality, 11 orthologs with postnatal lethality and 8 orthologs with both types of lethality (Table 1 and Table S9). Such genes may have important implications, since prenatal/perinatal death may relate to defective embryogenesis whereas postnatal death may signify perturbations of physiologic processes necessary for early survival [20]. There was no evidence for differences in the number of available knockout models for the 53 orthologs that were associated with lethality compared with the 97 non-lethal genes (median 2 [interquartile range 1 to 3] versus 1 [interquartile range 1 to 2], Mann-Whitney U test p-value = 0.13), so it is not likely that the first group had been studied far more extensively than the latter. Notably, this proportion of the orthologs displaying lethality (35%, [53/150]) was significantly different from the proportion of all knocked out genes associated with lethality in the MGI database (23% [1567/6812], p-value = 0.0006). Since knockout models for GWAS-derived genes are more likely to express a lethal phenotype compared to mouse models for all other genes, it is plausible that that the GWAS-derived genes may be involved in important developmental and physiological processes. However, given that lethality prevents the expression of other phenotypes of interest, these 53 orthologs were excluded from further comparisons of phenotypic expression between humans and mice.

**Table 1:** Ortholog genes displaying lethality in knockout mouse models.

Prenatal/perinatal lethality	Postnatal lethality	Both pre- and postnatal lethality
PTCH1, STAT3, APOB, ANGPTL3, HIST1H1D, BCL11A, BMP4, JAK2, ALPL, GATA2, HBB, HHEX, LPL, SH2B1, CYP17A1, HNF1A, HNF4A, ATG16L1, ATP2B1, CDK6, CXCL12, KCNJ2, KIF1B, MAFB, SLC2A9, TNIP1, BRSK1, GNA12, HMGCR, NKX2-1, SOX17, TCF7L2, HNF1B, LMTK2	HFE, TNFRSF11B, LDLR, GLIS3, INS, TNFAIP3, FTO, NKX2-3, FOXE1, IKZF2, LEF1	FGFR2, ABCA1, BDNF, ERBB3, GCK, PTGER4, SMAD7, MAF

We subsequently compared the phenotypic expression of the remaining 97 orthologs (i.e. those with knockout models that did not display lethality) and the corresponding phenotypes associated with these orthologs in the human GWAS. We restricted these comparisons to the affected anatomical system level, thus considering as agreement whenever an ortholog affected the same of the 31 anatomical systems (e.g. the ESR1 gene affected skeleton phenotypes in both species and was thus considered as an ortholog with concordant phenotypic information). For 69 orthologs (71%), the same anatomical system was affected in humans and mice, and for 13 of these 69 orthologs, mice and humans were concordant in two anatomical systems (Table S10). This total number of 82 phenotypic concordances between humans and mice was 4.5 times higher than the expected concordances by chance (expected n = 18.2) (p<0.001). The immune system (n = 32) and homeostasis/metabolism (n = 15) systems were the most commonly concordant systems.

In a sensitivity analysis, we considered only those orthologs for which no prior association had been proposed with the phenotype of interest by candidate-gene studies. There were 62 orthologs available (Table S11) and 40 of these orthologs were associated with the same anatomical system in humans and mice (4 orthologs showed concordance for 2 systems). The total number of observed concordances (n = 44) was again much higher (5.2-fold higher) than the expected number of concordant phenotypes (expected n = 8.5) (p<0.001). Concordance was still significant even when we focused only on the 41 orthologs that had not been proposed to be implicated in any association by candidate-gene studies (observed vs expected concordances  = 27 vs 4.8, p<0.001).

## Discussion

Our project represents a systematic comparison of GWAS-derived associations in humans and corresponding information from mouse models, based on curated and publicly available data. We used comprehensive databases from human and mouse research fields and we performed cross-species comparisons with two distinct approaches [4], [7]. Our findings highlighted the wealth of genetic and phenotypic information available in mouse models for the recently discovered genome-wide associations in humans. The two research fields were found to provide concordant information for certain gene-disease associations more often than what would have been expected by chance.

This project builds on a conceptual framework of gene-disease comparisons between different species, as developed by previous studies. Zhang et al. [21] used advanced bioinformatic methods to explore the extent of gene sharing between a broad range of human and mouse model phenotypes. The recently introduced concept of phenologs has expanded the scope of cross-species comparisons further: capitalizing on the orthology and evolutionary conservation of gene-networks, novel and non-obvious models of human disease with research utility can be uncovered [22]. Our work diverges from the previous approaches because our inferential target was the subset of human genetic associations that have emerged from agnostic investigations and thus require biological interpretation. Despite the small increments in disease risk conferred by GWAS-discovered common variants and their potentially limited clinical utility, we selected those variants with robust statistical support (p<10^−8^), which may represent genes with true biological implications. Moreover, there is increasing evidence that gene loci that harbor GWAS-discovered common variants may also harbor uncommon and rare variants and mutations that create related disease phenotypes [23], [24].

By meticulously reviewing the content of the MPO [13], [14], we were able to identify corresponding mammalian phenotypes for the majority of the examined phenotypes in humans. Because of the failure to a use a standard phenotype ontology for describing GWAS data by GWAS investigators [12], we evaluated the phenotypic matches between humans and mice based on detailed review of the mammalian phenotypic descriptions in MPO. There is some unavoidable subjectivity in this approach, but we decided upfront that it would be best to carefully juxtapose phenotypic terms and judge their similarity rather than rely on automated text mining techniques [21]. A comprehensive and standardized analysis of GWAS investigated phenotypes based on standardized ontology systems would greatly facilitate future integration with other types of experimental evidence from curated databases.

The extent of the concordant orthologs was much larger than the expected by chance, although this difference was much attenuated and lost nominal significance when focusing strictly on novel GWAS findings. This suggests that genes that have been identified to be associated with various diseases and phenotypes in the candidate gene era have been extensively and purposefully investigated in mouse models. It is also possible that the mouse models have been searched more stringently to identify relevant phenotypes proposed by candidate genes. Alternatively, for agnostically discovered genes from GWAS, there is less concordance with mouse models to-date. Nevertheless, concordance at the gene-level may underestimate true biological similarity between species. Although the specific sets of genes associated with the same phenotype in humans and mice may be different, these genes may operate within networks that determine the same biological function. Such similarities can potentially be demonstrated by future analyses that use molecular pathway ontology systems for the genes of interest, e.g. Gene Ontology. Thus, the orthologs found in our analyses to be associated with the examined phenotypes in mice only can further inform secondary analyses (either gene-focused or pathway-based) of existing datasets in humans [25].

In the gene-centric approach, we found that for the majority of the GWAS-derived genes, there are already available knockout models with deposited phenotypic information in MGI. We catalogued this phenotypic information and found extensive concordance between humans and mice, showing that certain orthologs can affect the same anatomical systems, and potentially the same biological function in both species. The concordance was still present when we excluded candidate-era genes.

This significant concordance is striking in view of the vast differences in the underlying genetic variants compared between humans and mice. In the GWAS, most of the associations for the common variants are likely due to variations altering gene function in relatively subtle ways. In contrast, knockout mouse models involve complete ablation of gene function, abolishing any activity of the corresponding protein. Furthermore, certain gene deletions were lethal in mice and thus were excluded from analyses. Despite these factors, we observed that the same anatomical systems were commonly affected in the two species; thus, our estimates of concordance may under-represent the true biological similarity that underlies the genetic associations in humans and mice.

The common variants studied in GWAS genotyping chips may tag rare variants that constitute the molecular basis of the observed associations [26]; rare variants could display more profound phenotypic effects and possibly affect the same mechanisms as the ones severely perturbed in knockout models. Moreover, common and uncommon variants affecting the same or similar phenotypes at different levels may coexist on the same gene and confer independent risks [23], [24]. Consequently, the comparisons between diametrically different mutational loads in humans and mice have meaningful biologic rationale.

Although phenotypes in many mouse models may not be agnostically or comprehensively ascertained [27], these expressed phenotypes can provide a plausible range of biological functions for the unknown mechanisms of the human GWAS-derived genes. Our database can also provide hints for secondary-hypotheses analyses in GWAS datasets and possible new discoveries, by examining the phenotypes observed in mice for certain orthologs. Deletion of GWAS orthologs in knockout mice was more often associated with lethal phenotypes compared to other genes in the MGI database. Such orthologs may have important developmental and physiological implications. This observation shows that associations gained from the agnostic GWAS have the potential to uncover important and previously unknown biology. Finally, 100 of the 250 (40%) GWAS-implicated orthologs were lacking a knockout model. Our project highlights these 100 orthologs as a subset of genes that may merit priority by laboratory mouse investigators for creation of knockout models.

Translation of GWAS discoveries into clinically meaningful diagnostic and therapeutic modalities will require an understanding of the underlying biology [2]. Our analyses showed that pre-existing mouse genetic models provide a wealth of analyzable information for the majority of human phenotypes studied and genes discovered in GWAS. We also found that significant concordance beyond chance between humans and mice exists. Current analyses are nevertheless limited by the fact that mouse phenotypic ascertainment is commonly narrow-scope and focused on prior biological hypotheses. Conversely, a significant minority of valid GWAS associations do not currently have any available genetically-modified mouse models for further investigation, a situation that can be expected to become more common as the number of GWAS-discovered genes increases. Ongoing international efforts, such as the Knockout Mouse Project [27], aim to create a comprehensive repository of knockouts for all mouse genes, with standardized phenotypic screens and publicly available information. Convergence of genomic data from different research venues [21], [22], [28] has the potential to drive new discoveries, to inform the pathophysiology of genetic associations and to accelerate the clinical translation of genomic applications.

## Supporting Information

Table S1 Phenotypes investigated in human genome-wide association studies (GWAS). Column A: initial list of eligible phenotypic entries as provided in the NHGRI catalog of GWAS; Column B: final list of 69 non-overlaping phenotypes that were obtained after merging similar phenotypes. Merged phenotypic entries are highlighted in gray color.
(0.13 MB DOC)

Table S2 The 31 Anatomical Systems according to the Mammalian Phenotype Ontology.
(0.05 MB DOC)

Table S3 Mapping of human phenotypes to the Mammalian Phenotype Ontology.
(0.09 MB DOC)

Table S4 Comparisons of the sets of ortholog genes associated with the same phenotype in humans and mice.
(0.12 MB DOC)

Table S5 All orthologs associated with human and mammalian corresponding phenotypes.
(0.18 MB DOC)

Table S6 GWAS-derived associations distinguished into novel ones and associations proposed by candidate gene studies.
(0.46 MB DOC)

Table S7 Comparisons of the sets of orthologs associated with the same phenotype in humans and mice (considering only the novel GWAS associations).
(0.14 MB DOC)

Table S8 Detailed phenotypic expression of all knocked out orthologs.
(4.11 MB DOC)

Table S9 Orthologs that displayed lethality in knocked out models.
(0.13 MB DOC)

Table S10 Comparisons of phenotypic expression between human GWAS genes and ortholog knocked out genes in mice (after excluding knockout genes associated with lethality).
(0.22 MB DOC)

Table S11 Comparisons of phenotypic expression between human GWAS genes and ortholog knocked out genes in mice (after excluding associations that had already been proposed in the candidate-gene era).
(0.18 MB DOC)

Figure S1 Anatomical Systems affected in the knockout models for the ortholog genes.
(0.39 MB TIF)
