# Supplementary material for: Laboratory Mouse Models for the Human Genome-Wide Associations
Source: PLoS One. 2010 Nov 1;5(11):e13782. doi: 10.1371/journal.pone.0013782 (PMC2967475; doi:10.1371/journal.pone.0013782)
Supplement: Table S1 — Phenotypes investigated in human genome-wide association studies (GWAS). Column A: initial list of eligible phenotypic entries as provided in the NHGRI catalog of GWAS; Column B: final list of 69 non-overlaping phenotypes that were obtained after merging similar phenotypes. Merged phenotypic entries are highlighted in gray color. (0.13 MB DOC) [file pone.0013782.s001.doc]

| **A. Eligible phenotypic entries as provided in the NHGRI catalog of GWAS** | **Entity affected** | **B. Final phenotypes analyzed** |
| --- | --- | --- |
| Alzheimer's disease |  | Alzheimer's disease |
| Asthma |  | Asthma |
| Atopic dermatitis |  | Atopic dermatitis |
| Basal cell carcinoma (cutaneous) |  | Basal cell carcinoma (cutaneous) |
| Bilirubin levels |  | Bilirubin levels |
| Bipolar disorder |  | Bipolar disorder |
| Blood pressure | **Blood pressure** | **Blood pressure related phenotypes** |
| Diastolic Blood Pressure |
| Systolic blood pressure |
| Hypertension |
| Bone mineral density | **Bone mineral density** | **Bone mineral density at various skeletal sites** |
| Bone mineral density (hip) |
| Bone mineral density (spine) |
| Breast cancer |  | Breast cancer |
| Celiac disease |  | Celiac disease |
| Chronic lymphocytic leukemia |  | Chronic lymphocytic leukemia |
| Colorectal cancer |  | Colorectal cancer |
| Coronary artery disease | **Coronary arteries** | **Coronary disease** |
| Myocardial infarction (early onset) |
| Creutzfeldt-Jakob disease |  | Creutzfeldt-Jakob disease |
| C-reactive protein |  | CRP concentration |
| Essential tremor |  | Essential tremor |
| Exfoliation glaucoma |  | Exfoliation glaucoma |
| F-cell distribution | **Fetal hemoglobin** | **Fetal hemoglobin levels** |
| Fetal hemoglobin levels |
| Folate pathway vitamins | **Vitamin B Complex** | **Folate pathway vitamins and vitamin B12 levels** |
| Plasma level of vitamin B12 |
| Gallstones |  | Gallstones |
| Height |  | Height |
| Hepatitis B |  | Hepatitis B |
| Protein quantitative trait loci (IL18)* |  | IL-18 concentration |
| Protein quantitative trait loci (IL-6sR)* |  | IL-6sR concentration |
| Crohn's disease | **Inflammatory bowel disease** | **Inflammatory bowel disease** |
| Ulcerative colitis |
| Inflammatory bowel syndrome |
| Inflammatory bowel disease |
| Intracranial aneurysm |  | Intracranial aneurysm |
| Juvenile idiopathic arthritis |  | Juvenile idiopathic arthritis |
| Cholesterol, total | **Lipids** | **Lipid phenotypes** |
| HDL cholesterol |
| LDL cholesterol |
| Triglycerides |
| Plasma Lp (a) levels |
| Morbidity-free survival |  | Longevity |
| Lung cancer |  | Lung cancer |
| Male-pattern baldness |  | Male-pattern baldness |
| Protein quantitative trait loci (MCP 1)* |  | MCP 1 concentration |
| Mean platelet volume |  | Mean platelet volume |
| Melanoma |  | Melanoma |
| Menarche (age at onset) | **Reproductive physiological phenomena** | **Menarche and/or menopause (age at onset)** |
| Menarche and menopause (age at onset) |
| Menopause (age at onset) |
| Menopause (age at onset) |
| MIPb concentration |  | MIPb concentration |
| Multiple sclerosis |  | Multiple sclerosis |
| Myeloproliferative neoplasms |  | Myeloproliferative neoplasms |
| Myopathy |  | Myopathy |
| Neuroblastoma |  | Neuroblastoma |
| Obesity | **Body weights and measures** | **Obesity related phenotypes** |
| Body mass (lean) |
| Body mass index |
| Waist circumference and related phenotypes |
| Weight |
| Panic disorder |  | Panic disorder |
| Plasma carotenoid and tocopherol levels |  | Plasma carotenoid and tocopherol levels |
| Plasma eosinophil count |  | Plasma eosinophil count |
| Plasma levels of liver enzymes |  | Plasma levels of liver enzymes |
| Prostate cancer |  | Prostate cancer |
| Psoriasis |  | Psoriasis |
| Pulmonary function measures |  | Pulmonary function measures |
| QT interval |  | QT interval |
| Biomedical quantitative traits (pulse rate)* |  | pulse rate |
| Renal function and chronic kidney disease |  | Renal function and chronic kidney disease |
| Restless legs syndrome |  | Restless legs syndrome |
| Rheumatoid arthritis |  | Rheumatoid arthritis |
| Serum IgE levels |  | Serum IgE levels |
| Serum markers of iron status |  | Serum markers of iron status |
| Serum urate | **Xanthines** | **Serum urate/uric acid** |
| Serum uric acid |
| Black vs. blond hair color | **Pigmentation** | **Skin/hair/eye color related phenotypes** |
| Black vs. red hair color |
| Blond vs. brown hair color |
| Red vs non-red hair color |
| Blue vs brown eyes |
| Iris color |
| Skin pigmentation by reflectance spectroscopy |
| Skin sensitivity to sun |
| Tanning |
| Soluble ICAM-1 |  | Soluble ICAM-1 |
| Ischemic stroke | **Cerebrovascular disorder** | **Stroke** |
| Stroke |
| Successful cognitive aging |  | Successful cognitive aging |
| Systemic lupus erythematosus | **Systemic lupus erythematosus** | **Systemic lupus erythematosus** |
| Systemic lupus erythematosus in women |
| Thyroid cancer |  | Thyroid cancer |
| Select biomarker traits (TNFa)* |  | TNFa concentration |
| Type 1 diabetes |  | Type 1 diabetes |
| Type 2 diabetes |  | Type 2 diabetes |
| Venous thromboembolism |  | Venous thromboembolism |
| Warfarin maintenance dose |  | Warfarin maintenance dose |
| Wet age-related macular degeneration |  | Wet age-related macular degeneration |
| YKL-40 levels |  | YKL-40 (chitinase-like protein) concentration |

* These phenotypic entries used vague and non-informative descriptors (e.g. “select biomarker traits”). For those phenotypic entries, detailed information from the respective GWAS publications was collected in order to define the exact phenotype investigated.
