# Supplementary material for: Laboratory Mouse Models for the Human Genome-Wide Associations
Source: PLoS One. 2010 Nov 1;5(11):e13782. doi: 10.1371/journal.pone.0013782 (PMC2967475; doi:10.1371/journal.pone.0013782)
Supplement: Table S2 — The 31 Anatomical Systems according to the Mammalian Phenotype Ontology. (0.05 MB DOC) [file pone.0013782.s002.doc]

| **Anatomical Systems** |
| --- |
| adipose tissue |
| behavior/neurological |
| cardiovascular |
| cellular |
| craniofacial |
| digestive/alimentary |
| embryogenesis |
| endocrine/exocrine glands |
| growth size |
| hearing/ear/vestibular |
| hematopoietic |
| homeostasis/metabolism |
| immune system |
| lethality/postnatal |
| lethality/prenatal-perinatal |
| life span/aging |
| limbs/digits/tail |
| liver/biliary |
| muscle |
| nervous |
| normal phenotype |
| pigmentation |
| renal/urinary |
| reproductive |
| respiratory |
| skeleton |
| skin/nails |
| taste/olfaction |
| touch/vibrissae |
| tumorigenesis |
| vision/eye |
