# Supplementary material for: Laboratory Mouse Models for the Human Genome-Wide Associations
Source: PLoS One. 2010 Nov 1;5(11):e13782. doi: 10.1371/journal.pone.0013782 (PMC2967475; doi:10.1371/journal.pone.0013782)
Supplement: Table S3 — Mapping of human phenotypes to the Mammalian Phenotype Ontology. (0.09 MB DOC) [file pone.0013782.s003.doc]

| **Human phenotypes mapped to Mammalian Phenotype Ontology using MeSH terms searches** | | |
| --- | --- | --- |
| **Human disease** | **MP term** | **MP id** |
| Serum markers of iron status | Abnormal Iron level | MP:0001770 |
| Prostate cancer | prostate adenocarcinoma | MP:0009220 |
| Coronary disease | atherosclerotic lesions | MP:0005338 |
| QT interval | abnormal QT interval | MP:0003899 |
| Type 1 diabetes | Increased susceptibility to autoimmune diabetes | MP:0004803 |
| Inflammatory bowel disease | intestinal inflammation | MP:0001858 |
| Obesity related phenotypes | Abnormal body weight | MP:0001259 |
| Lipid phenotypes | Abnormal lipid homeostasis | MP:0002118 |
| Bone mineral density | Abnormal Bone Mineral Density | MP:0010119 |
| Plasma eosinophil count | abnormal eosinophil cell number | MP:0002602 |
| Atopic dermatitis | Dermatitis | MP:0001194 |
| Rheumatoid arthritis | rheumatoid arthritis | MP:0003561 |
| Basal cell carcinoma (cutaneous) | basal cell carcinoma | MP:0004208 |
| Male-pattern baldness | Alopecia | MP:0000414 |
| Essential tremor | Tremors | MP:0000745 |
| Myopathy | Myopathy | MP:0000751 |
| Psoriasis | Psoriasis | MP:0001193 |
| Neuroblastoma | Neuroblastoma | MP:0002039 |
| CRP concentration | abnormal C-reactive protein physiology | MP:0002484 |
| Serum IgE levels | increased IgE level | MP:0002497 |
| Creutzfeldt-Jakob disease | spongiform encephalopathy | MP:0002654 |
| Gallstones | Gallstones | MP:0002830 |
| Menarche and/or menopause (age at onset) | late onset of menarche | MP:0003377 |
| Thyroid cancer | thyroid adenoma | MP:0003496 |
| Systemic lupus erythematosus | increased susceptibility to systemic lupus erythematosus | MP:0004801 |
| Venous thromboembolism | Thrombosis | MP:0005048 |
| Chronic lymphocytic leukemia | small lymphocytic lymphoma | MP:0009319 |
| Height | abnormal body height | MP:0001253 |
| Breast cancer | mammary gland tumor | MP:0006318 |
| Blood pressure related phenotypes | abnormal blood pressure | MP:0000230 |
| pulse rate | abnormal heart rate | MP:0001629 |
| Renal function and chronic kidney disease | abnormal kidney physiology | MP:0002136 |
| Bilirubin levels | abnormal circulating bilirubin level | MP:0001569 |
| Stroke | CNS ischemia | MP:0006080 |
| Intracranial aneurysm | Aneurysm | MP:0003279 |
| TNFa concentration | abnormal circulating tumor necrosis factor level | MP:0008552 |
| IL-6sR concentration | abnormal circulating interleukin-6 level | MP:0008595 |
| IL-18 concentration | abnormal circulating interleukin-18 level | MP:0008634 |
| Lung cancer | lung carcinoma | MP:0008714 |
| Serum urate/uric acid | abnormal blood uric acid level | MP:0008820 |
| Mean platelet volume | abnormal platelet volume | MP:0002586 |
| Colorectal cancer | large intestine adenocarcinoma | MP:0009310 |
| Plasma levels of liver enzymes | abnormal liver physiology | MP:0000609 |
|  |  |  |
| **Human phenotypes mapped to Mammalian Phenotype Ontology by manual Mammalian Phenotype Browser searches** | | |
| Skin/hair/eye color related phenotypes | abnormal coat/hair pigmentation - abnormal skin pigmentation | MP:0002075 /MP:0002095 |
| Type 2 diabetes | insulin resistance | MP:0005331 |
| Asthma | Abnormal Bronchial Provocation | MP:0002330 |
| Wet age-related macular degeneration | retinal cone cell degeneration | MP:0008444 |
| Multiple sclerosis | Demyelination | MP:0000921 |
| Pulmonary function measures | abnormal forced expiratory flow rates | MP:0002297 |
| Alzheimer's disease | amyloid beta deposits - neurofibrillary tangles | MP:0003329 /MP:0003214 |
| Longevity | extended life span | MP:0001661 |
|  |  |  |
| **Human phenotypes without equivalent mammalian phenotypes in the Mammalian Phenotype Ontology** | | |
| Bipolar disorder | - | - |
| Celiac disease | - | - |
| Fetal hemoglobin levels | - | - |
| Folate pathway vitamins and vitamin B12 levels | - | - |
| Hepatitis B | - | - |
| Juvenile idiopathic arthritis | - | - |
| MCP 1 concentration | - | - |
| Melanoma | - | - |
| MIPb concentration | - | - |
| Myeloproliferative neoplasms | - | - |
| Panic disorder | - | - |
| Plasma carotenoid levels | - | - |
| Plasma tocopherol levels | - | - |
| Restless legs syndrome | - | - |
| Soluble ICAM-1 | - | - |
| Successful cognitive aging | - | - |
| Warfarin maintenance dose | - | - |
| YKL-40 (chitinase-like protein) concentration | - | - |
