# Supplementary material for: Laboratory Mouse Models for the Human Genome-Wide Associations
Source: PLoS One. 2010 Nov 1;5(11):e13782. doi: 10.1371/journal.pone.0013782 (PMC2967475; doi:10.1371/journal.pone.0013782)
Supplement: Table S4 — Comparisons of the sets of ortholog genes associated with the same phenotype in humans and mice. (0.12 MB DOC) [file pone.0013782.s004.doc]

| Human disease | MP term | MP id | Anatomical system affected | Child Phenotypic terms | Number of mouse models | Number of mouse genes | Number of human genes | Number of concordances | Concordant genes |
| --- | --- | --- | --- | --- | --- | --- | --- | --- | --- |
| Serum markers of iron status | Abnormal Iron level | MP:0001770 | homeostasis/metabolism | yes | 19 | 9 | 3 | 2 | TF, HFE |
| Skin/hair/eye color related phenotypes | abnormal coat/hair pigmentation - abnormal skin pigmentation | MP:0002075 /MP:0002095 | skin/nails | no | 385 | 162 | 14 | 4 | OCA2, SLC45A2, TYR, TYRP1 |
| Prostate cancer | prostate adenocarcinoma | MP:0009220 | tumorigenesis | no | 20 | 9 | 6 | 1 | MSMB |
| Coronary disease | atherosclerotic lesions | MP:0005338 | cardiovascular | yes | 51 | 35 | 6 | 1 | LDLR |
| QT interval | abnormal QT interval | MP:0003899 | cardiovascular | yes | 9 | 6 | 11 | 1 | KCNQ1 |
| Type 1 diabetes | Increased susceptibility to autoimmune diabetes | MP:0004803 | immune system | no | 81 | 34 | 23 | 1 | INS |
| Inflammatory bowel disease | intestinal inflammation | MP:0001858 | immune system, digestive/alimentary | yes | 57 | 42 | 24 | 3 | IL10, NOD2, MST1 |
| Type 2 diabetes | insulin resistance | MP:0005331 | homeostasis/metabolism | no | 64 | 44 | 13 | 1 | GCK |
| Obesity related phenotypes | Abnormal body weight | MP:0001259 | growth size | yes | 969 | 614 | 12 | 3 | BDNF, MC4R, SH2B1 |
| Lipid phenotypes | Abnormal lipid homeostasis | MP:0002118 | homeostasis/metabolism | yes | 581 | 280 | 28 | 10 | ABCA1, APOB, APOC1, LCAT, LDLR, LIPC, LIPG, LPL, PCSK9, PLTP |
| Bone mineral density | Abnormal Bone Mineral Density | MP:0010119 | skeleton | yes | 185 | 82 | 7 | 3 | ESR1, LRP5, TNFRSF11B |
| Asthma | Abnormal Bronchial Provocation | MP:0002330 | respiratory | yes | 27 | 23 | 7 | 0 |  |
| Plasma eosinophil count | abnormal eosinophil cell number | MP:0002602 | immune system, hematopoietic | no | 2 | 2 | 5 | 0 |  |
| Atopic dermatitis | Dermatitis | MP:0001194 | skin/nails, immune system | no | 61 | 31 | 1 | 0 |  |
| Rheumatoid arthritis | rheumatoid arthritis | MP:0003561 | immune system, skeleton | no | 2 | 2 | 4 | 0 |  |
| Basal cell carcinoma (cutaneous) | basal cell carcinoma | MP:0004208 | tumorigenesis, skin/nails | no | 7 | 4 | 1 | 0 |  |
| Wet age-related macular degeneration | retinal cone cell degeneration | MP:0008444 | nervous, vision/eye | no | 5 | 5 | 1 | 0 |  |
| Male-pattern baldness | Alopecia | MP:0000414 | skin/nails | no | 124 | 91 | 2 | 0 |  |
| Essential tremor | Tremors | MP:0000745 | nervous | no | 170 | 143 | 1 | 0 |  |
| Myopathy | Myopathy | MP:0000751 | muscle | no | 23 | 17 | 1 | 0 |  |
| Multiple sclerosis | Demyelination | MP:0000921 | nervous | no | 46 | 34 | 3 | 0 |  |
| Psoriasis | Psoriasis | MP:0001193 | skin/nails | no | 5 | 2 | 5 | 0 |  |
| longevity | extended life span | MP:0001661 | life span/aging | no | 21 | 15 | 1 | 0 |  |
| Neuroblastoma | Neuroblastoma | MP:0002039 | tumorigenesis | no | 2 | 2 | 1 | 0 |  |
| Pulmonary function measures | abnormal forced expiratory flow rates | MP:0002297 | respiratory | no | 0 | 0 | 1 | 0 |  |
| CRP concentration | abnormal C-reactive protein physiology | MP:0002484 | immune system | no | 1 | 1 | 6 | 0 |  |
| Serum IgE levels | increased IgE level | MP:0002497 | immune system | no | 42 | 36 | 1 | 0 |  |
| Creutzfeldt-Jakob disease | spongiform encephalopathy | MP:0002654 | nervous | no | 8 | 6 | 1 | 0 |  |
| Gallstones | Gallstones | MP:0002830 | liver/biliary | no | 47 | 26 | 1 | 0 |  |
| Alzheimer's disease | amyloid beta deposits - neurofibrillary tangles | MP:0003329 /MP:0003214 | nervous | no | 14 | 7 | 2 | 0 |  |
| Menarche and/or menopause (age at onset) | late onset of menarche | MP:0003377 | reproductive | no | 1 | 1 | 4 | 0 |  |
| Thyroid cancer | thyroid adenoma | MP:0003496 | tumorigenesis | no | 1 | 1 | 2 | 0 |  |
| Systemic lupus erythematosus | increased susceptibility to systemic lupus erythematosus | MP:0004801 | immune system | no | 27 | 18 | 6 | 0 |  |
| Venous thromboembolism | Thrombosis | MP:0005048 | homeostasis/metabolism | no | 28 | 23 | 1 | 0 |  |
| Chronic lymphocytic leukemia | small lymphocytic lymphoma | MP:0009319 | tumorigenesis | no | 2 | 1 | 2 | 0 |  |
| Height | abnormal body height | MP:0001253 | growth size | yes | 8 | 8 | 22 | 0 |  |
| Breast cancer | mammary gland tumor | MP:0006318 | tumorigenesis | yes | 44 | 20 | 7 | 0 |  |
| Blood pressure related phenotypes | abnormal blood pressure | MP:0000230 | cardiovascular | yes | 165 | 115 | 6 | 0 |  |
| pulse rate | abnormal heart rate | MP:0001629 | cardiovascular | yes | 76 | 64 | 1 | 0 |  |
| Renal function and chronic kidney disease | abnormal kidney physiology | MP:0002136 | renal/urinary | yes | 62 | 48 | 2 | 0 |  |
| Bilirubin levels | abnormal circulating bilirubin level | MP:0001569 | homeostasis/metabolism | yes | 13 | 13 | 2 | 0 |  |
| Stroke | CNS ischemia | MP:0006080 | nervous | yes | 1 | 1 | 2 | 0 |  |
| Intracranial aneurysm | Aneurysm | MP:0003279 | cardiovascular | yes | 11 | 11 | 1 | 0 |  |
| TNFa concentration | abnormal circulating tumor necrosis factor level | MP:0008552 | immune system, homeostasis/metabolism | yes | 26 | 22 | 1 | 0 |  |
| IL-6sR concentration | abnormal circulating interleukin-6 level | MP:0008595 | immune system, homeostasis/metabolism | yes | 19 | 18 | 1 | 0 |  |
| IL-18 concentration | abnormal circulating interleukin-18 level | MP:0008634 | immune system, homeostasis/metabolism | yes | 2 | 2 | 1 | 0 |  |
| Lung cancer | lung carcinoma | MP:0008714 | tumorigenesis | yes | 46 | 29 | 2 | 0 |  |
| Serum urate/uric acid | abnormal blood uric acid level | MP:0008820 | homeostasis/metabolism | yes | 1 | 1 | 3 | 0 |  |
| Mean platelet volume | abnormal platelet volume | MP:0002586 | hematopoietic | yes | 8 | 6 | 3 | 0 |  |
| Colorectal cancer | large intestine adenocarcinoma | MP:0009310 | tumorigenesis | yes | 5 | 5 | 4 | 0 |  |
| Plasma levels of liver enzymes | abnormal liver physiology | MP:0000609 | liver/biliary | yes | 123 | 87 | 4 | 0 |  |

MP: mammalian phenotype
