# Supplementary material for: Laboratory Mouse Models for the Human Genome-Wide Associations
Source: PLoS One. 2010 Nov 1;5(11):e13782. doi: 10.1371/journal.pone.0013782 (PMC2967475; doi:10.1371/journal.pone.0013782)
Supplement: Table S5 — All orthologs associated with human and mammalian corresponding phenotypes. (0.18 MB DOC) [file pone.0013782.s005.doc]

| Human disease | MP term | MP id | Anatomical system | Child Phenotypic terms | N of mouse models | N of mouse genes | Mouse Genes | N of Human Genes | Human Genes | N of concordant genes | Concordant genes |
| --- | --- | --- | --- | --- | --- | --- | --- | --- | --- | --- | --- |
| Inflammatory bowel disease | intestinal inflammation | MP:0001858 | immune system, digestive/alimentary | MP:0003304 MP:0009481 MP:0002816 MP:0008539 MP:0008537 MP:0003305 | 57 | 42 | Cdcs1, Cdcs10, Cdcs2, Cdcs3, Cdcs4, Cdcs5, Cdcs6, Cdcs7, Cdcs8, Cdcs9, Gpx1, Gpx2, Il10, Tg(Cd4-cre)1Cwi, Pik3cd, Relb, Runx3, Tnfaip3, Dssc2, Dssc1, Il2ra, Smad3, Tcra, Il2, Ahr, Cebpe, Rag2, Gnai2, Was, Gpdc1, Gpdc2, Gpdc3, B2m, Il10rb, Tsta3, Ptgs1, Il6, Tff3, Sigirr, Ereg, Mst1, Nod2 | 24 | ATG16L1, C11orf30, CARD15, CCR6, CDKAL1, ICOSLG, IL10, IL12B, IL23R, IRGM, ITLN1, JAK2, MST1, NKX2-3, NOD2, ORMDL3, PSMG1, PTGER4, PTPN2, RNF186, STAT3, TNFRSF6B, TNFSF15, ZNF365 | 3 | IL10, NOD2, MST1 |
| Plasma eosinophil count | abnormal eosinophil cell number | MP:0002602 | immune system, hematopoietic |  | 2 | 2 | Ccr3, Il5ra | 5 | GATA2, IKZF2, IL1RL1, IL5, SH2B3 | 0 |  |
| TNFa concentration | abnormal circulating tumor necrosis factor level | MP:0008552 | immune system, homeostasis/metabolism | MP:0008554 MP:0008553 | 26 | 22 | Arrb2, Mbl1, Mbl2, Tnfrsf1b, Tyro3, Axl, Mertk, Cav1, Parg, Ifnar1, Dnase2a, Slamf6, Tnf, Mif, Irak4, Npy1r, Parp1, Mcpt1, Lyz2, Tg(Cd4-cre)1Cwi, Casp1, Irf5 | 1 | ABO | 0 |  |
| IL-6sR concentration | abnormal circulating interleukin-6 level | MP:0008595 | immune system, homeostasis/metabolism | MP:0008597 MP:0008596 | 19 | 18 | Mbl1, Mbl2, Ptger4, Cebpb, Itgb2, Cav1, Arrb2, Cxcr2, Il1rn, Il1r1, Irak4, Ticam1, Serpine1, Casp1, Tg(Il1rn)1Dih, Irf5, Tlr4, Spp1 | 1 | IL6R | 0 |  |
| IL-18 concentration | abnormal circulating interleukin-18 level | MP:0008634 | immune system, homeostasis/metabolism | MP:0008636 MP:0008635 | 2 | 2 | Dnase2a, Ifnar1 | 1 | IL18 | 0 |  |
| Atopic dermatitis | dermatitis | MP:0001194 | skin/nails, immune system |  | 61 | 31 | Areg, B2m, Blmh, Card11, Cbl, Ctsl, Derm1, Derms, Dsc1, Dsg4, EIF1AX, Elovl3, Ereg, Foxo3, Foxp3, Hic1, ITGA2, ITGB1, Itgb2, Itgb3, Kitl, Mme, Nfkbiz, Plcg2, Rag1, Relb, Slc30a4, Top3b, Trpv3, Vdr | 1 | C11orf30 | 0 |  |
| Rheumatoid arthritis | rheumatoid arthritis | MP:0003561 | immune system, skeleton |  | 2 | 2 | Dnase2a, Ptger4 | 4 | CD40, HLA-DRB1, PTPN22, TRAF1 | 0 |  |
| Basal cell carcinoma (cutaneous) | basal cell carcinoma | MP:0004208 | tumorigenesis, skin/nails |  | 7 | 4 | Mlh1, Msh6, Ptch1, Tom1l2 | 1 | RHOU | 0 |  |
| Wet age-related macular degeneration | retinal cone cell degeneration | MP:0008444 | nervous, vision/eye |  | 5 | 5 | Axl, Pde6c, Rho, Rp1, Rpgr | 1 | HTRA1 | 0 |  |
| Blood pressure related phenotypes | abnormal blood pressure | MP:0000230 | cardiovascular | MP:0003882 MP:0006266 MP:0006265 MP:0002843 MP:0006263 MP:0004876 MP:0006264 MP:0001596 MP:0002842 MP:0000231 MP:0004216 MP:0004217 MP:0006143 MP:0004875 MP:0006144 | 165 | 115 | Adra1b, Abbp1, Abbp2, Abbp3, Abbp4, Ace, Ace2, Actc1, Adh5, Adm, Adra1a, Adra1d, Adra2a, Agtr1a, Agtr2, Aplnr, Apoe, Atp2a2, Bpq1, Bpq2, Bpq3, Bpq4, Bpq5, Bpq6, Bpq7, Bpq8, Bpq9, Brs3, Calca, Chga, Clock, Corin, Crhr2, Cyp11b2, Cyp19a1, Drd5, Dusp1, Edn1, Egfr, Ephx2, Esr2, Fbln5, Fgf2, Gja1, Guca2b, Hlb10, Hlb12, Hlb13, hlb15, Hlb27, Hlb39, Hlb40, hlb54, Hlb59, Hlb60, Hlb66, Hlb68, hlb72, Hlb73, Hlb75, Hlb76, Hprt, Hypt, Igf1, Ighmbp2, Irs1, Kcnj1, Kcnma1, Kcnmb1, Kras, Lepr, Mkks, Mrvi1, Myh11, Myh6, Nos2, Nos3, Nppa, Npr1, Oxt, P2rx1, Pcsk2, Pln, Podxl, Pparg, Prkg1, Ptger1, Ptger2, Ptgs1, Ptgs2, Ptpn11, Ren1, Rgs2, Slc12a2, Slc12a3, Slc12a6, Slc2a4, Slc6a18, Slc9a3, Spp1, Sysbp1, Sysbp2, Tg(Alb1-Ren)1Unc, Tg(AQP2-cre)2Dek, Tg(DM15)26Bew, Tg(Edn1-NOS3)22Yky, Tg(Myh6-Actg2)1Jll, Tg(Myh6-Gnaq)25Gwd, Tg(NOS3)2Crom, Tg(NOS3)3Crom, Tg(Tagln-cre)1Her, Tg(Tek-cre)1Dlg, Tph1, Vdr, Wnk1 | 6 | ATP2B1, CDH13, CYP17A1, PLEKHA7, SH2B3, ULK4 | 0 |  |
| Male-pattern baldness | alopecia | MP:0000414 | skin/nails |  | 124 | 91 | Acp2, Aft/Aft, Ahr, Al/Al, Alaa1, alp/alp, ao/ao, ap/ap, Areg, ba/ba, Bmpr1a, Ctnnb1, Ctsl, cub/cub, Cux1, Dsc1, Dsg3, Dsg4, Ebp, Egfr, Ercc2, Fhit, Gsdma3, Hoxc13, Hr, Itgb2, Itgb6, Krt17, Krt71, Ksr1, Lef1, Lrig1, Msx2, Nfkbiz, Nsdhl, Ny/Ny, pfz/pfz, Plcd1, Polg, Pts, qbal/qbal, Recql4, Relb, Scd1, Scg5, Sgk3, sgl/sgl, Shh, Slc30a4, Soat1, Sox18, Sox9, St14, Terc, Tg(APOC1)3Lmh/Y, Tg(BMP4)6Blh/0, Tg(Crh)227.1Pbl/0, Tg(EGFR)0Jlj/0, Tg(EGFR)1Jlj/0, Tg(EGFR)2Jlj/0, Tg(EGFR)3Jlj/0, Tg(EGFR)4Jlj/0, Tg(EGFR)5Jlj/0, Tg(EIF1AX-Il31ra)1Jagr/0, Tg(FGF7)11Efu/0, Tg(FGF7)29Efu/0, Tg(Foxn1)6Jlb/0, Tg(Hoxc13)61B1Awg/0, Tg(Hoxc13)61U4Awg/0, Tg(Il1a)1.1Tsk/0, Tg(Il1a)1.2Tsk/0, Tg(Il6)1Efu/0, Tg(K5-Ptgs2)19Kmd/0, Tg(K5-Ptgs2)667Kmd/0, Tg(K5-Ptgs2)675Kmd/Tg(K5-Ptgs2)675Kmd, Tg(Krt1-14-GLI)1Oro/0, Tg(Krt14-dnLef1)LFmw/?, Tg(KRT16)10Cou/Tg(KRT16)10Cou, Tg(Krt2-9)1Grog/0, Tg(Lck-Il31ra)1Jagr/0, Tg(Notch1)A5Rko/0, Tg(Notch1)A7Rko/0, Tg(rv-CYCLIN)29Dlh/0, Tg(Wnt3)7Gsb/0, Tg(Wnt3)7Gsb/Tg(Wnt3)7Gsb, Tg(Zfp38)A4Htz/0, Tg(Zfp38)D1Htz/0, Traf6, Ttc7, Vdr, Zfp36 | 2 | AR, PAX1 | 0 |  |
| Plasma levels of liver enzymes | abnormal liver physiology | MP:0000609 | liver/biliary | MP:0010080 MP:0008891 MP:0004001 MP:0003887 MP:0003893 MP:0003065 MP:0003067 MP:0003066 MP:0005517 MP:0005516 MP:0010042 MP:0003325 MP:0009356 MP:0010027 MP:0009355 MP:0002310 MP:0005415 MP:0000611 MP:0003326 | 123 | 87 | Abca5, Abcg5, Acox1, Ahr, Apoa1, Apoe, Asgr1, Atg7, Tg(Mx1-cre)1Cgn, Atp8b1, Cav1, Clcn3, Clock, Cno, Cyp19a1, Cyp8b1, Ercc1, Gba, Ghrh, Gjb1, Tg(Alb-cre)21Mgn, Insr, Klb, Lipa, Mat1a, Mlxipl, Mttp, Pemt, Ppard, Ppargc1a, Pxmp3, Rbp1, Scd2, Sele, Sell, Selp, Slc10a2, Stat5b, Stat5a, Tnf, Fech, Jag1, Notch2, Spna1, Ank1, Invs, Spnb1, Tg(Alb1-cre)7Gsc, Hnf1b, Onecut1, T(2;10)67Gso, Foxp3, Soat2, Trpv1, Prkar2b, Npc1l1, Atp7b, Cp, Atox1, Pkd1, Ikbkb, Cbs, Rgn, Epor, Jun, Adar, Met, Raf1, Timp3, Tg(Fabp1-Ccnd1)4Rdb, Igfbp1, Rxra, Prox1, Ada, Abcb11, Abcb4, Crem, Rps6, C3, Nr0b2, Tg(aP2-SREBF1c)9884Reh, Gpam, Scp2, Npc1, Abcb1a, Stard3, Npc2 | 4 | ADAMTS13, GPLD1, JMJD1C, REEP3 | 0 |  |
| Essential tremor | tremors | MP:0000745 | nervous |  | 170 | 143 | Abca5, Abcb1a, Ache, Acp2, Adam23, Aifm1, Ank1, Ank3, anx/anx, Araf, Arsa, Aspa, Atf2, Atp1b2, Atp2b2, Atp7b, Atrn, awag/awag, awg, Bmi1, Bmp7, Cacna1a, Cacna2d2, Camk4, Ccr10/Cntnap1, Cdkn1b, ci/ci, Cit, cla/cla, Cldn11, Cnp, Colq, Cpe, Ctsa, Cys1, Dmd, Dst, Egr2, Ercc2, Gal3st1, Galc, Gjc2, Glb1, Glra1, Gria2, Grm1, gt/gt, Hexb, Hip1, Hprt, Htra2, Htt, Ireb2, Kcna1, Kcnc1, Kcnj10, Kcnma1, Kif5a, Lamc1, Liph, Lyst, Maf, Mag, Mapt, Mecp2, Mgat2, Mpz, Mtmr2, Nab1, Nbn, Nfia, Ngf, nmf111/nmf111, nmf118/nmf118, nmf206/nmf206, Nmf220/?, nmf313/nmf313, nmf4/nmf4, nmf419/nmf419, Npc2, Nphp3, nur1/nur1, nur10/nur10, nur20/nur20, nur38/nur38, nur4/nur4, nur47/nur47, nur5/nur5, nur50/nur50, nur60/nur60, nur61/nur61, nur62/nur62, nur63/nur63, nur68/nur68, nur69/nur69, nur70/nur70, Pitpna, Plp1, Prnp, Pts, Pura, Reln, Rora, Scn8a, shmy, Slc6a5, Slc7a10, Smn1, Smpd1, Spnb4, Syngap1, Tcfap2b, Tg(HD82Gln)81Dbo/0, Tg(HDexon1)62Gpb/0, Tg(Mpz)80.2Wra/0, Tg(Mpz)80.3Wra/Tg(Mpz)80.3Wra, Tg(NEFH)200Jpj/0, Tg(NEFH)200Jpj/Tg(NEFH)200Jpj, Tg(NFH)120Jpj/0, Tg(NFH)120Jpj/Tg(NFH)120Jpj, Tg(Plp)66Kan/Tg(Plp)66Kan, Tg(Prnp-SNCA*A53T)83Vle/Tg(Prnp-SNCA*A53T)83Vle, Tg(SOD1*G93A)2Gur/0, tm/tm, to/to, Tpp1, trm/trm, tth/tth, Ttpa, twit/twit, Twsg1, Ty/Ty, Ty/Y, Tyl/Tyl, Tyl/Y, Uchl1, Ugt8a, Usp14, Usp18, Vac14, Wasf1, wl, Zfp423 | 1 | LINGO1 | 0 |  |
| Myopathy | myopathy | MP:0000751 | muscle |  | 23 | 17 | Cav3, Col13a1, Col15a1, Col6a1, Dmd, Gaa, Itga7, Lama2, Large, Ldb3, Mtm1, Plau, Plec, Slc25a4, Stmn1, Tg(Ckm-Mkl1)1Eno/0, Tg(DM15)26Bew/0 | 1 | SLCO1B1 | 0 |  |
| Multiple sclerosis | demyelination | MP:0000921 | nervous |  | 46 | 34 | Arsa, Cd4, Cd8a, Cnp, Dst, Eae14, Eae19, Eae20, Edaradd, Galc, Gjc2, gt/gt, H2-D1, Ifnb1, Lama2, Mpz, Ndrg1, nmf161/nmf161, Pitpna, Plp1, Pmp22, Psap, Ptpre, Scarb2, Scn8a, Slc12a6, Stmn1, Tg(Plp)66Kan/Tg(Plp)66Kan, Tmevd5, Tmevd6, Tmevd7, Tmevd8, Tmevd9, Tpp1, Ugt8a | 3 | HLA-DRA, HLA-DRB1, KIF1B | 0 |  |
| Psoriasis | psoriasis | MP:0001193 | skin/nails |  | 5 | 2 | itgb2, Irig1 | 5 | HLA-C, IL12B, IL13, TNFAIP3, TNIP1 | 0 |  |
| Height | abnormal body height | MP:0001253 | growth size | MP:0001255 MP:0001254 | 8 | 8 | Nkx3-2, Mgp, Lmna, Lpar1, Acp2, Smpd3, Ihh, Med1 | 22 | BMP6, CDK6, C6orf106, DLEU7, EFEMP1, GDF5, GNA12, GPR126, HHIP, HIST1H1D, HMGA2, JAZF1, LCORL, PLAG1, PTCH1, SOCS2, SPAG17, BFZB, UQCC, ZBTB38, LOC387103, ZNF678 | 0 |  |
| Obesity related phenotypes | Abnormal body weight | MP:0001259 | growth size | MP:0001262 MP:0006086 MP:0003213 MP:0005659 MP:0008489 MP:0001263 MP:0005150 MP:0006087 MP:0003212 MP:0005658 MP:0001261 MP:0005456 MP:0005455 | 969 | 614 | Abca1, Abcb11, Abcc9, Abl2, Acacb, Ache, Acox1, Acp2, Acta1, Ad, Ada, Adam22, Adamts1, Adcy3, Adcyap1, Adcyap1r1, Adipoq, Adk, Ahr, Airn, Akt1, Akt2, Aldh5a1, Alms1, alp, Alpl, Amacr, Angptl6, Ank, Ank2, anx, Anxa2, Ap1g1, Aplp1, Apoe, App, Aprt, Aqp1, Aqp5, Araf, Arsb, Aspa, Atf2, Atf4, Atg7, Atm, Atp12a, Atp8b1, Avpr2, Bad, Bbs1, Bbs2, Bbs4, Bcat2, Bcl2, Bcl6, bd, Bdnf, Bdw1, Bfq1, bgby, Bgn, blb2, Bmd8, Bmp5, Bmp7, Brs3, Bsbob, Bsg, Bub1b, Bw15, Bw17, Bw18, Bw8q1, C10bw2, C10bw3, C10bw4, C10bw6, C3, Cacna1d, Cacna2d2, Camk4, can, Capn3, Cartpt, Casr, Cav1, Cbs, Cbx2, Ccnb2, Ccnd1, Cd59a, Cdk4, Cdk6, Cdkn1a, Cdkn1c, Cdkn2c, Cdx2, Cebpa, Cenpb, Cftr, Cga, cha, Chrm2, Chrm3, Chrna3, Chrne, cir, Cit, cl, Clcn3, Cldn1, Clock, Clps, Cngb1, Cnp, Cnr1, Cntn1, Cntnap1, Col19a1, Col1a1, Col2a1, Col4a3, Col5a2, Cops5, Cpe, Crebbp, Crkl, crsp, Cryab, Cst6, Ctnna2, Ctsd, Ctsl, cu, Cux1, Cycs, Cyp19a1, Cyp27b1, Dbf, Del(10AI646023-Ggt5)1Bayer, Del(11Cops3-4933439F18Rik)2Jrl, Del(11Cops3-Zfp179)1Jrl, Dgat2, Dhcr24, Dnaja1, Dnajc3, Dnajc5, Dock7, Dpp4, Drd2, Dsc1, Dsg3, Dsg4, Dst, E2f4, Ebp, Edaradd, Ednrb, Egfr, Egr1, Egr2, Eif2ak3, Eng, Erbb3, Ercc2, Ercc4, Ercc6, Ereg, Esam, Esr1, Esrra, Etv5, Fabp2, Fanca, Fancd2, Fbxo11, Fcgr2b, Fgf23, Fgfr3, Fnld, Fob3a, Fob3b, Foxb1, Foxj1, Foxl2, Foxn1, Foxp3, Fus, Gad2, Galc, Gamt, Gba, Gcgr, Gck, Gdf5, Gfi1, Gfra2, Ggt1, Ghr, Ghrh, Ghrhr, Ghsr, Gjb1, Gnai2, Gnas, Gnaz, Gnb5, Gnf1, Gng3, Gnpat, Gnrh1, Gpam, Gpd2, Gpx1, gr, Grb14, gri, Gria2, Grid2, Grik2, Grin1, gro, gro14, gro17, gro24, gro25, gro26, gro27, gro28, gro29, gro30, gro31, gro32, gro34, gro35, gro36, gro38, gro4, Gstz1, Gsx1, Gt(ROSA)26Sor, Gulo, Gvhd3, Gy, Hap1, Hba, Hexa, Hip1, hlb124, Hlb125, Hlb131, Hlb132, Hlb147, Hlb163, Hlb181, Hlb197, Hlb199, Hlb228, Hlb230, Hlb44, hlb52, Hlb80, Hlb81, Hlb93, Hmga2, Hmgb1, Hmox1, Hoxc13, Hoxd13, Hspa1a, Hspa1b, Hspb2, Htr3a, Htr4, Htra2, Htt, Id4, Ifrd1, Igf1, Igf2, Igf2bp1, Igfals, Ighmbp2, Il15, Il1rn, Il2, Il2ra, Il2rb, Il6, In(7p;7Sox6)100H, Inha, Inppl1, Ins2, Insr, Invs, Irs1, Irs2, Irs4, Jak1, Jun, Kcna3, Kcnj10, Kcnj6, Kcnk5, Kcnma1, Kcnq1, Kcnq1ot1, Keap1, Kitl, Kl, Klb, Krt10, Krt8, l15R9, Lama2, Lama3, Lamp2, Large, Lats1, Ldb3, Lep, Lepr, Lgmn, Lgr4, Lhcgr, Lif, Lifr, Lipa, Lipe, Liph, Lmf1, Lmna, Lpar1, Ltbp3, Lum, Mafg, Man2b1, Mapt, Mark2, Mc4r, Mchr1, Mecp2, Mest, Minute, Mity, Mkks, Mmp14, Mmp19, Mob1, Mtap1b, Mtap2, Mtgq4, Mthfr, Mtm1, Mtmr2, Myh1, Myh11, Myh4, Myo7a, Myst4, Naglu, Nav2, Nbn, Ncoa3, Neurl2, Neurod2, Neurod4, Ngf, Nhlh2, Nidd2k, Nidds, Nmf15, nmf65, Nmu, Nod2, Nos3, Nox3, Npbwr1, Npc1, Npc1l1, Npc2, Nppc, Npr2, Npy5r, Nr0b2, Nr1h4, Nr1i3, Nr2e1, Nr3c2, Nrcam, Nrg1, Nrg2, Nsdhl, Ntf3, Ntrk2, Obq16, Obq17, Obq19, Oca2, Ostm1, Otx1, Ovol1, P2rx7, Pank2, Pappa, Papss2, Park2, Pbwg1, Pbwg2, Pbwg3, Pbwg4, Pbwg5, Pbwg6, Pbwg7, Pcsk1, Pde4d, Pdx1, Pea15a, Peg3, Per2, Pex11b, Pex5, Pex7, Phex, Phox2a, Pin1, Pip4k2b, Pitpna, Pkcp, Pkd1, Pla2g1b, Plcb4, Plec, Plp1, Plscr3, Pmch, Polg, Pomc, Pparg, Ppargc1a, Ppp1r13l, Ppp1r3a, Ppp1r9b, Ppp3cb, Prkaca, Prkar2b, Prkch, Prkg2, Prlr, Prodh, Prox1, Prrxl1, Prx, Psap, Psen1, Ptpn1, Ptpn11, Ptpn13, Ptprd, Pttg1, Pura, Pzp, Rad50, Raf1, Rai1, Rarg, Rasd2, Rasgrf1, Rb1, Recql4, Relb, repro10, repro15, Rgs4, Ripk1, Rln1, rmy, Rpl24, Rps6ka3, Rps6kb1, Rsc1a1, Runx1, Runx1t1, Runx2, Runx3, Ryk, Safb, Scgb1a1, Scn2a1, Scn8a, Scnn1b, Sdc3, Sema3a, Sepp1, Serpine1, Sftpc, Sgcg, Sgk1, Sh2b1, Siah1a, Sim1, Sirpa, Skil, Skp2, Slc10a2, Slc12a2, Slc12a7, Slc13a1, Slc18a2, Slc19a1, Slc26a5, Slc27a4, Slc29a1, Slc2a2, Slc2a4, Slc30a5, Slc34a1, Slc37a4, Slc3a1, Slc4a1, Slc6a2, Slc6a3, Slc6a5, Slc6a9, Slc7a1, Slc7a10, Slc9a1, Slit3, Smarcad1, Smpd1, Snap25, Socs1, Socs6, Socs7, Sod2, Sox3, Sox8, Sp4, Spg7, Spnb4, Spred1, Spred2, Src, Sstr5, St14, Star, Stat3, Stat5b, Strbp, Stzid, Surf1, Sypl2, Szt1, Tabw, Tarbp2, Tbs1, Tbs2, Tcirg1, Terc, Tg, Tg(Apcs-Lep)1Yog, Tg(AZIP/F)1Vsn, Tg(CAG-Angpt16)8.1Yo, Tg(CD4-HIV)F21380Pjo, Tg(CD4-HIV)F21388Pjo, Tg(CD4-HIV)F21407Pjo, Tg(CD4-HIV)F26605Pjo, Tg(CD4-HIV)F26622Pjo, Tg(CD4-HIV)F26990Pjo, Tg(CD4-HIV)F27011Pjo, Tg(CD4-HIV)F27367Pjo, Tg(CD4-HIV)F27372Pjo, Tg(Ckm-Mkl1)1Eno, Tg(Ckmm-Ucp1)1Boud, Tg(Crh)227.1Pbl, Tg(Fabp2-Arg1)1Wla, Tg(HBB-TNF)197Gkl, Tg(HD82Gln)81Dbo, Tg(HDexon1)62Gpb, Tg(Il6)1Efu, Tg(Mpz)80.2Wra, Tg(Ngf)56Kma, Tg(Prnp)1D4Lnq, Tg(Prnp-SNCA*A53T)83Vle, Tg(UcpDta)1Kz, Tgfb1, Tgfb2, Tgm1, Thra, Timp3, Tkt, Tnf, to, Tpst1, Traf2, Traf6, trm, Trp53bp1, Trp73, Trpv1, Tshr, Tsn, Ttc7, Ttn, Tub, Tw, Twist2, Twsg1, Ube2a, Ubr1, Uchl1, Uchl3, Ucp1, Ugt8a, Unc5c, Uncv, Vdr, Vegfa, Vgf, Vldlr, Vti1b, Wasf1, Whrn, wl, Wnt2, Wt10q1, Xdh, Zfp36, Zfx, Zic1, Zic4, Zic5 | 12 | BDNF, C12orf51, CETP, FTO, GNPDA2, MAF, MC4R, MTCH2, NEGR1, SH2B1, TMEM18, TRHR | 3 | BDNF, MC4R, SH2B1 |
| Bilirubin levels | abnormal circulating bilirubin level | MP:0001569 | homeostasis/metabolism | MP:0005635 MP:0005344 | 13 | 13 | Abcb4, Fech, Spna1, Hadha, Tg(Alb1-cre)7Gsc, Hnf1b, Rdx, Pitpna, Hbb-b1, Hbb-b2, Abcg2, Atp8b1, Hk1 | 2 | SLCO1B3, UGT1A1 | 0 |  |
| pulse rate | abnormal heart rate | MP:0001629 | cardiovascular | MP:0004122 MP:0003929 MP:0003928 MP:0005333 MP:0002626 MP:0008950 | 76 | 64 | Nos3, Egfr, Adra2a, Jup, Mgp, Cyp19a1, Cry1, Cry2, Calca, Npy2r, Thra, Hrq1, Hrq2, Hrq3, Sox4, Kcnj3, Tg(Myh6-cre)2182Mds, Gja1, Nppa, Mchr1, Tg(Myh6-cre/Esr1*)1Jmk, Cdh2, Tph1, Spp1, Ptger1, Ighmbp2, Idua, Adra1a, Cacna1d, Tbx5, Nrg1, Ntf3, Rxra, Tg(Tek-cre)1Dlg, Slc8a1, Adrb1, Adrb2, Kcnh2, Tg(Myh6-Gnaq)25Gwd, Braf, Lepr, Clock, Pts, Sp4, Adra1b, Mef2a, Slc30a5, Epha3, Th, Tead1, Csrp3, Pln, Tg(Alb1-Ren)1Unc, Adra2b, Adra2c, Bmp10, Vegfa, Cacna2d2, Foxp1, Mrvi1, Ppargc1a, Myl2, Sfrs1, Lmna | 1 | LOC644502 | 0 |  |
| longevity | extended life span | MP:0001661 | life span/aging |  | 21 | 15 | Coq7, Shc1, Tgm2, Cd40lg, Ide, Psen1, Slc30a3, App, Klc1, Tg(Myh6-CASQ2)1Mord, Tg(HDexon1)62Gpb, Tg(APPSWE)2576Kha, Tg(Thy1-cre)1Vln, Tg(Thy1-APP<SL>)28Lpr, Tg(APP695)3Dbo, | 1 | DPT |  |  |
| Serum markers of iron status | Abnormal Iron level | MP:0001770 | homeostasis/metabolism | MP:0004152 MP:0004151 MP:0008810 | 19 | 9 | Tfrc, Alas2, Hfe, Cp, Ttc7, Hfe2, Hmox1, Trf, Trfr2, Ltf | 3 | HFE, PAFAH1B2, TF | 2 | TF, HFE |
| Neuroblastoma | neuroblastoma | MP:0002039 | tumorigenesis |  | 2 | 2 | Htatip2, Nf1 | 1 | BARD1 |  |  |
| Skin/hair/eye color related phenotypes | abnormal coat/hair pigmentation - abnormal skin pigmentation | MP:0002075 - MP:0002095 | skin/nails |  | 385 | 162 | a, Adam17, ap, Ap3b1, Atp7a, Atrn, Bcl2, brwd, Cyp19a1, dds, Del(10AI646023-Ggt5)1Bayer, din, Eda, Edaradd, Ednrb, fe, Frem2, Ga, ge, Ggt1, Gn, Kit, Kitl, l4Jus12, Mitf, Myo5a, Ods, Ostm1, Pax3, Ph, Polg, Rab38, Rlbp1, rmy, rs, sgl, Shmu, skc16, skc17, skc18, skc28, skc29, skc30, skc31, Slc45a2, Sox10, Sta, Stol, Tbx15, Tg(JUP)21Pac, Tg(JUP)45Pac, Tg(JUP)4Pac, Tg(JUP)9Pac, Tyr, wam, wn, Fgfr2, Brca1, a, Ascl1, Spna1, Adamts20, Ank1, Snai2, Dsg4, Tmgc21, Foxn1, Traf6, Zfp141, Lgt, Gnaq, Rps20, Rps19, Krt2, Egfr, Gsfsco7, Ttc7, Cal7, Barx2, Dst, Hps4, Tg(BMP4)6Blh, exma, Lyst, Rn, pwk, da, Si, Gsfwnw, dal, lgr, baw, Nsdhl, gr, Recql4, Tcfe3, Tcfec, Hps5, Src, Hps3, Bcl2l11, baln2, Tw, Hps6, Oca2, Tyrp1, Cno, Tmgc18, tmgc14, Pts, Dtnbp1, dp, chtl, Dock7, gri, Slc7a11, plto, Rabggta, Och, Mchm1, Mlph, Fw, smk, dill, Pomc, nur15, nur16, nur17, Dct, skc10, skc12, skc14, skc19, skc21, skc22, skc23, skc6, skc9, sea, Vps33a, Tg(Dct-SOX9)aCeb, titanm, powder, gand, Ap3d1, Vac14, Sox2, Hps1, Mreg, Muted, Pldn, Bloc1s3, Acd, Gna11, Dsk9, skc15, Bswt, Dsk6, Gsfwt, Tcfap2a, Tg(Wnt1-cre)11Rth, Skc43 | 14 | ASIP, EXOC2, GRM5, HERC2, IRF4, KITLG, MC1R, OCA2, SLC24A4, SLC24A5, SLC45A2, TPCN2, TYR, TYRP1 | 4 | OCA2, SLC45A2, TYR, TYRP1 |
| Lipid phenotypes | Abnormal lipid homeostasis | MP:0002118 | homeostasis/metabolism | MP:0005278 MP:0003947 MP:0005342 MP:0002645 MP:0002647 MP:0002646 MP:0001547 MP:0003947 MP:0000180 MP:0000184 MP:0000186 MP:0001556 MP:0000181 MP:0000183 MP:0000182 MP:0005144 MP:0005146 MP:0005145 MP:0005179 MP:0003457 MP:0003458 MP:0002575 MP:0002644 MP:0003976 | 581 | 280 | Scarb1, Pltp, Gck, Lipc, Apoe, Npc1, Lipe, Apob, Chol6, Dhcr24, Nhdlq1, Nhdlq2, Scp2, Oca2, Pemt, Amacr, Plscr3, Nhdlq6, Nhdlq4, Nhdlq5, Nhdlq7, Lprq4, Pnhdlc1, Pnhdlc2, Pnhdlc3, Pla2g15, Ldlrap1, Npc1l1, Tg(APOB)11Rub, Tg(UcpDta)1Kz, Lipa, Nr1h3, Tg(APOC1)1Bres, Pon1, Slc3a1, Cpe, Hdl5, Gcgr, Chohd1, Chohd5, Chohd6, chohr2, chohr3, Diet1, Abca1, Soat2, Lpl, Angptl3, Mttp, Tg(Mx1-cre)1Cgn, Prkar2b, Cx3cl1, Ldlr, Pcsk9, Tg(Alb-cre)21Mgn, Ppara, Lch, Lch2, Tg(LIPG)1Tq, Apoa1, Apoa2, Gpam, Hlb216, hlb218, Hlb237, Hlb295, Hlb206, Hlb252, Hdlq5, Hdlq6, Hdlq7, Hdlq9, Hdlq8, Hdlq14, Hdlq15, Hdlq19, Pxmp3, Tnfsf4, Ath2, Hyplip2, Lprq2, Lcat, Abca7, hlb241, Hlb320, Gulo, Irs1, Hdl36, Hdl35, Hdl40, Adipoq, Akp3, Cav1, Cyp11a1, Dgat2, Fabp1, Fcer1g, Hexb, Gpx1, Lrpap1, Tg(Myh6-cre)2182Mds, Nr2f2, Tg(Ins2-cre)25Mgn, Pex7, Phospq1, Ppard, Psap, Slc27a1, Tg(Ckmm-cre)5Khn, Slc2a4, Soat1, Tg(Apcs-Lep)1Yog, Tg(APOA1)1Rub, Tg(rPEPCKSREBF2)788Reh, Ugt8a, Lipg, Cyp19a1, Ath9, Hdlq4, Hdlq1, Hdlq2, Hdlq3, Hdl1, Hlb211, Hlb212, Hlb239, Hlb250, Hlb240, Hdlq10, Hdlq11, Hdlq12, Smpd1, Hdl6, Lipq1, Lipq2, Lipq3, Hdlq17, Hdlq18, Hdlq20, Hdlq21, Hdlq22, Hdlq23, Hdlq24, Hdlq25, Hdlq26, Hdlq27, Hdlq28, Hdlq29, Hdlq30, Hdlq31, Hdlq32, Hdl2, Hdl4, Hdl3, Lprq3, chohr1, Tg(Ttr-Foxo1)307Dac, Hdl34, Phdlc1, Phdlc2, Hdlcl1, Hdlcl2, Hlb396, Hlb280, Hlb397, Irs2, Cd36, Hdlq16, Cebpb, Tnf, Cebpa, Ptgs2, Tg(Mx1-cre)29-4Her, Lrp1, Abcd1, Acadvl, Niacr1, Nr1h2, Ucp1, Arntl, Arsa, Asah1, St3gal5, Nr0b1, Ttpa, Gnpat, Tg(Alb1-cre)7Gsc, Hnf1b, Akt2, Akt1, Mt1, Mt2, Lith6, Lith12, Lith13, Lith14, Cidea, Slc10a2, G6pc, Sftpb, Fabp3, Acacb, Esrra, Pik3r1, Slc2a2, Insr, Nhdlq3, Mlxipl, Npc2, Slc37a4, Plscr1, Nmu, C3, Lep, Tg(AZIP/F)1Vsn, Asgr2, Ppargc1a, Npbwr1, Inppl1, Mthfr, Angptl6, Gab1, Sod2, Cyp8b1, Adcyap1, Scd1, Tg(Pax6-cre,GFP)1Pgr, Pax6, Ins2, Aqp3, Fabp4, Ubr1, Por, Clps, Pitpna, Fgf23, Crhr2, Tgls1, Socs3, Tg(Nes-cre)1Kln, Tg(Syn1-cre)671Jxm, Irs3, Chrm3, Spp1, Scd2, Chab1, Chab2, Chab3, Chab4, Chab5, Cckar, Chab6, Chab7, Pnlip, Muc1, skimp, Tg(APOC1)1Lmh, Tg(APOC1)3Lmh, Fn1, Lprq1, Tg(NOS3)2Crom, Tg(NOS3)3Crom, Nr0b2, Gusb, gro22, Abcg5, Mob1, Fabp2, Nr5a2, Tg(CMV-EGFP,Rnu6-siAbca1)#Wcyy, Tg(ALB-cre,CMV-rtTA)#Wcyy, Abcg8, Il1rn, Gt(ROSA)26Sor, Sc5d, Cyp46a1, Cyp7b1, Nphp3, Pnliprp2, Pla2g1b | 28 | ABCA1, ABCG5, ABCG8, ANGPTL3, ANKRD30A, APOB, APOC1, CELSR2, CETP, DNAH11, DOCK7, GALNT2, GCKR, HMGCR, HNF4A, LCAT, LDLR, LIPC, LIPG, LPA, LPL, MAFB, MLXIPL, NCAN, PCSK9, PLEK, PLTP, TMEM57, TRIB1, TTC39B | 10 | ABCA1, APOB, APOC1, LCAT, LDLR, LIPC, LIPG, LPL, PCSK9, PLTP |
| Renal function and chronic kidney disease | abnormal kidney physiology | MP:0002136 | renal/urinary | MP:0005556 MP:0005558 MP:0005557 MP:0005555 MP:0002847 MP:0005528 MP:0005527 | 62 | 48 | Actn4, Akr1b3, Aprt, Aqp1, Aqp3, Aqp4, Avpr2, Cav1, Cd2ap, Clcn5, Cno, Dapk1, Drd5, Dtnbp1, Esr1, Fgf23, Guca2b, Hps1, Mfge8, Pkd1, Pldn, Podxl, Pxmp3, Rends, Rrm2b, Slc12a1, Slc14a1, Slc15a2, Slc22a5, Slc4a1, Slc6a18, Slc9a3, Tcfap2b, Tg(Igk-V21-Bax)1967Bvn, Trpv5, Vps33a, Serpinc1, Fxyd4, Ptgs1, Col4a3, Ptpro, Sgk1, Kcnj1, Umod, Hps5, Slc13a1, Kcnk5, Adm | 2 | SHROOM3, UMOD | 0 |  |
| Pulmonary function measures | abnormal forced expiratory flow rates | MP:0002297 | respiratory |  | 0 | 0 |  | 1 | HHIP | 0 |  |
| Asthma | Abnormal Bronchial Provocation | MP:0002330 | respiratory | MP:0002334 MP:0002335 MP:0001952 | 27 | 23 | Tbx21, Aqp5, Ccr3, Hlb171, Hlb173, Il4ra, Icam2, Hlb102, Asthm1, Asthm2, Bhr3, Bhr2, Runx3, Spred1, Bhr1, Bhr5, Cxcr2, Hlb413, C3ar1, Calca, Il10 | 7 | C6orf97, FGFR2, LSP1, MAP3K1, TNRC9, TOX3, ORMDL3 | 0 |  |
| CRP concentration | abnormal C-reactive protein physiology | MP:0002484 | immune system |  | 1 | 1 | Meox2 | 6 | APOE, CRP, GCKR, HNF1A, LEF1, LEPR | 0 |  |
| Serum IgE levels | increased IgE level | MP:0002497 | immune system |  | 42 | 36 | Bhlhe40, Card11, Ccr7, Csf2, Cxcr2, Dsg3, Dsg4, Fcer1g, Fcer2a, Hbnr6, Ikzf3, Il10, Il13ra2, Il2, Il2rb, Il4ra, Lat, Lax1, Lmr10, Lmr12, Lmr14, Lmr3, Lmr5, Lmr8, Lmr9, Nfkbiz, Noa/Noa, Ppia, Ptpn22, Rabgef1, Relb, Runx3, Tg(Igk-V21-Bax)1967Bvn/0, Traf3ip2, Wipf1, Igh-J | 1 | FCER1A | 0 |  |
| Mean platelet volume | abnormal platelet volume | MP:0002586 | hematopoietic | MP:0008935 MP:0002599 | 8 | 6 | Mpl, Thpo, Gp1ba, Rabggta, Gp1bb, Kit | 3 | ARHGEF3, TAOK1, WDR66 | 0 |  |
| Creutzfeldt-Jakob disease | spongiform encephalopathy | MP:0002654 | nervous |  | 8 | 6 | Fxn, Aspa, gr, Ppargc1a, Atrn, Vac14 | 1 | PRNP | 0 |  |
| Gallstones | gallstones | MP:0002830 | liver/biliary |  | 47 | 26 | Ank1, Cckar, Hdl37, Hdl39, Lith1, Lith10, Lith11, Lith12, Lith14, Lith15, Lith16, Lith17, Lith18, Lith19, Lith2, Lith20, Lith21, Lith3, Lith4, Lith5, Lith6, Lith7, Lith8, Lith9, Muc1, Pzp, Vti1b | 1 | ABCG8 | 0 |  |
| Intracranial aneurysm | Aneurysm | MP:0003279 | cardiovascular | MP:0006278 MP:0003275 MP:0003274 | 11 | 11 | Ntf3, Hspg2, Tg(Tie1-cre)9Ref, Pdgfb, Runx1, Gja1, Nphp3, Mgp, Spp1, Lox, Timp1, Pkd1 | 1 | SOX17 | 0 |  |
| Alzheimer's disease | amyloid beta deposits - neurofibrillary tangles | MP:0003329 - MP:0003214 | nervous |  | 14 | 7 | Msr1, Psen1, Cd40lg, Ide, Slc30a3, App, Klc1 | 2 | APOE, GAB2 | 0 |  |
| Menarche and/or menopause (age at onset) | late onset of menarche | MP:0003377 | reproductive |  | 1 | 1 | Magel2 | 4 | BRSK1, LIN28B, MCM8, UIMC1 | 0 |  |
| Thyroid cancer | thyroid adenoma | MP:0003496 | tumorigenesis |  | 1 | 1 | Nbn | 2 | FOXE1, NKX2-1 | 0 |  |
| QT interval | abnormal QT interval | MP:0003899 | cardiovascular | MP:0004156 MP:0003233 MP:0003900 | 9 | 6 | Egfr, Ptpn11, Kcne1, Vegfa, Kcnq1, Gja1 | 11 | TF, ATP1B1, KCNH2, KCNJ2, KCNQ1, LITAF, NDRG4, NOS1AP, PLN, RNF207, SCN5A | 1 | KCNQ1 |
| Systemic lupus erythematosus | increased susceptibility to systemic lupus erythematosus | MP:0004801 | immune system |  | 27 | 18 | Agnm1, Agnm2, Agnm3, Apoe, Asbb1, Asbb2, Asbb3, Cgnz1, Imh1, Lbw5, Lbw6, Lbw7, Rasgrp1, Sle6, Sles1, Sles2, Sles3, Sles4 | 6 | BANK1, HLA-DQA1, PHRF1, PXK, STAT4, TNFAIP3 | 0 |  |
| Type 1 diabetes | Increased susceptibility to autoimmune diabetes | MP:0004803 | immune system |  | 81 | 34 | Aire, B2m, Cd274, Cd28, Cd38, Cd4, Cd40lg, Cd80, Cd86, Cdk2, Cdk4, E2f1, Fas, Faslpr, Fcgr2b, Gna12, Gna13, H2-Ab1, Igh-6, Ins1, Ins2, Irs1, Irs2, Lepr, Pdcd1, Prkdc, Ptprn, Rag1, Stat4, Stat6, Tlr5, Tnfrsf1a, Tnfrsf1b, Tnfsf10, Vdr | 23 | BACH2, C10orf59, C12orf30, C6orf173, CD69, CLEC16A, CTLA4, CTSH, ERBB3, GLIS3, HLA-E, IFIH1, IL10, IL2, IL27, IL2RA, INS, ORMDL3, PRKCQ, PTPN2, PTPN22, SH2B3, UBASH3A | 1 | INS |
| Venous thromboembolism | thrombosis | MP:0005048 | homeostasis/metabolism |  | 28 | 23 | Abca5, Actc1, Ahr, Alox12, Anxa2, Axl, Ctsd, F5, Hmox1, Pf4, Plat, Plau, Ppp1r13l, Prdx6, Proc, Procr, Ptgir, Ptgs2, Rap1b, Serpinc1, Serpind1, Sult1e1, Tfpi | 1 | ABO | 0 |  |
| Type 2 diabetes | insulin resistance | MP:0005331 | homeostasis/metabolism |  | 64 | 44 | Adipoq, Adipoq, Akt2, Angptl6, Bdnf, Cav1, Cpe, Crebbp, Cyp19a1, Dbh, Dbsty3, Fabp4, Gck, Gnas, Hadha, Hmga1, Hrh3, Insr, Irs1, Irs2, Lepr, Lipa, Lipe, Lnpep, Mlxipl, Nos3, Nr2f2, Plscr3, Ppara, Pparg, Prkaa2, Prlhr, Rps6ka3, Sh2b1, Slc2a4, Slc6a6, Stx4a, Stxbp3a, Tabw2, Tg(aP2-SREBF1c)9884Reh/0, Tg(APOB)11Rub/0,Tg(UcpDta)1Kz/0, Tg(SLC2A4-OGT)15Jaha/0, Tg(UcpDta)1Kz/?, Thra | 13 | CDKAL1, FTO, G6PC2, GCK, HHEX, IGF2BP2, JAZF1, KCNJ11, KCNQ1, MTNR1B, SLC30A8, TCF7L2, THADA | 1 | GCK |
| Coronary disease | atherosclerotic lesions | MP:0005338 | cardiovascular | MP:0005340 MP:0005341 MP:0005339 | 51 | 35 | Apoe, Msr1, Tg(APOC1)1Bres, Pon1, Aorls1, Aorls2, Ath6, Hyplip2, Shc1, Athla1, Ath8, Pla2g15, Athsq1, Artles, Athsq2, Ath18, Ath19, Ath20, Ath21, Tnfsf4, Ath2, Hps1, Tlr4, Cx3cr1, Tg(NOS3)2Crom, Tg(NOS3)3Crom, Ath17, Fn1, Plau, Cx3cl1, Ldlr, Tg(Il1rn)1Dih, Tg(APOA1)1Rub, Hps4, Spp1 | 6 | CXCL12, LDLR, MIA3, MRAS, PHACTR1, PSRC1 | 1 | LDLR |
| Stroke | CNS ischemia | MP:0006080 | nervous | MP:0006081 | 1 | 1 | Gpx1 | 2 | NINJ2, NR | 0 |  |
| Breast cancer | mammary gland tumor | MP:0006318 | tumorigenesis | MP:0001883 MP:0006422 MP:0009508 | 44 | 20 | Actb, Apc, Apmt1, Apmt2, Becn1, Brca1, Brca2, Chek1, Dkc1, Etv4, Grb2, Mmp11, Mtes1, Nbn, Prdx1, Pten, S100a4, Trp53, Wap | 7 | C6orf97, FGFR2, LSP1, MAP3K1, TNRC9, TOX3, ORMDL3 | 0 |  |
| Lung cancer | lung carcinoma | MP:0008714 | tumorigenesis | MP:0008716 MP:0002027 MP:0008718 MP:0008717 MP:0008715 | 46 | 29 | Nf1, Nf2, Tg(SFTPC-MST1R)71Mhw, Tg(MMTV-ERBB3)1Slg, Tg(MMTVneu)202Mul, Prdx1, Nbn, Becn1, Sluc5, Sluc25, Sluc9, Sluc10, Sluc18, Sluc26, Pold1, Prdm2, Msh2, Ogg1, Mutyh, Nudt1, Mad2l1, Fntb, Tg(CMV-cre/ERT)1Ipc, Kras, Tg(CMV-cre)1Cgn, Trp53, Dkc1, Cdkn2a, S100a4 | 2 | CLPTM1L, NR | 0 |  |
| Serum urate/uric acid | abnormal blood uric acid level | MP:0008820 | homeostasis/metabolism | MP:0008822 MP:0008821 | 1 | 1 | anx | 3 | ABCG2, SLC17A3, SLC2A9 | 0 |  |
| Prostate cancer | prostate adenocarcinoma | MP:0009220 | tumorigenesis |  | 20 | 9 | Hprt, Kras, Msmb, Nkx3-1, Psca, Pten, Tbx21, Pbsn, Tramp | 6 | EHBP1, HNF1B, KLK3, LMTK2, MSMB, SLC22A3 | 1 | MSMB |
| Colorectal cancer | large intestine adenocarcinoma | MP:0009310 | tumorigenesis | MP:0009314 MP:0009316 MP:0009313 MP:0009315 | 5 | 5 | H2afx, lig4, Pole, Rab25, tgfb1 | 4 | BMP4, EIF3H, RHPN2, SMAD7 | 0 |  |
| Bone mineral density | Abnormal Bone Mineral Density | MP:0010119 | skeleton | MP:0010121, MP:0010120 | 185 | 82 | Acp5, Acp5, Ager, Ali18, Ankrd11, Arhgap1, Aspa, Bdkrb2, Bmp3, Calca, Calcr, Creb3l1, Csf1r, Ctsk, Darc, Ebf2, Ephb6, Ercc2, Esr1, Esr2, Fgf23, Fgfr2, Fgfr3, Flnb, Ghr, Ghrhlit, Gja1, Grm1, Gulosfx, Hdac6, He, Hlb, Idua, Igf1, Il6, Il6st, Ins2, Irs1, Itgb2, Junb, Khdrbs1, Kitl, Kl, Klf10, Klkl, Lect1, Lifr, Lmna, Lrp5, Meox2, Mstn, Ncoa1, Nos1, Nos3, Nov, Nr3c1, Pthlh, Ptpn1, Pxmp3, Pyy, Pzp, Qrfpr, Rag1, Sh3bp2, Sirt6, Slc39a13, Slc9a3r1, Smad2, Smad3, Spp1, Tcirg1, Thra, Tlr4, Tnfrsf11a, Tnfrsf11b, Tnfsf11, Traf6, Trps1, Twist2, Tyrobp, Vdr, Zmpste24 | 7 | ESR1, FAM3C, LRP5, OPG, RANKL, TNFRSF11B, ZBTB40 | 3 | ESR1, LRP5, TNFRSF11B |
| Chronic lymphocytic leukemia | small lymphocytic lymphoma | MP:0009319 | tumorigenesis |  | 2 | 1 | Cdkn2a | 2 | GRAMD1B, IRF4 | 0 |  |
