# Supplementary material for: Laboratory Mouse Models for the Human Genome-Wide Associations
Source: PLoS One. 2010 Nov 1;5(11):e13782. doi: 10.1371/journal.pone.0013782 (PMC2967475; doi:10.1371/journal.pone.0013782)
Supplement: Table S6 — GWAS-derived associations distinguished into novel ones and associations proposed by candidate gene studies. (0.46 MB DOC) [file pone.0013782.s006.doc]

| disease | MP term | MP id | Reported Gene(s) | Homolog gene Symbol |
| --- | --- | --- | --- | --- |
| **Novel GWAS-derived gene-phenotype associations** | | | | |
| Blood pressure related phenotypes | abnormal blood pressure | MP:0000230 | ATP2B1 | Atp2b1 |
| Blood pressure related phenotypes | abnormal blood pressure | MP:0000230 | CDH13 | Cdh13 |
| Blood pressure related phenotypes | abnormal blood pressure | MP:0000230 | CYP17A1 | Cyp17a1 |
| Blood pressure related phenotypes | abnormal blood pressure | MP:0000230 | PLEKHA7 | Plekha7 |
| Blood pressure related phenotypes | abnormal blood pressure | MP:0000230 | SH2B3 | Sh2b3 |
| Blood pressure related phenotypes | abnormal blood pressure | MP:0000230 | ULK4 | Ulk4 |
| Male-pattern baldness | alopecia | MP:0000414 | PAX1 | Pax1 |
| Plasma levels of liver enzymes | abnormal liver physiology | MP:0000609 | ABO | Abo |
| Plasma levels of liver enzymes | abnormal liver physiology | MP:0000609 | ADAMTS13 | Adamts13 |
| Plasma levels of liver enzymes | abnormal liver physiology | MP:0000609 | GPLD1 | Gpld1 |
| Plasma levels of liver enzymes | abnormal liver physiology | MP:0000609 | JMJD1C | Jmjd1c |
| Plasma levels of liver enzymes | abnormal liver physiology | MP:0000609 | REEP3 | Reep3 |
| Essential tremor | tremors | MP:0000745 | LINGO1 | Lingo1 |
| Multiple sclerosis | demyelination | MP:0000921 | KIF1B | Kif1b |
| Psoriasis | psoriasis | MP:0001193 | TNFAIP3 | Tnfaip3 |
| Psoriasis | psoriasis | MP:0001193 | TNIP1 | Tnip1 |
| Atopic dermatitis | dermatitis | MP:0001194 | C11orf30 | 2210018M11Rik |
| Height | abnormal body height | MP:0001253 | BMP6 | Bmp6 |
| Height | abnormal body height | MP:0001253 | CDK6 | Cdk6 |
| Height | abnormal body height | MP:0001253 | C6orf106 | D17Wsu92e |
| Height | abnormal body height | MP:0001253 | DLEU7 | Dleu7 |
| Height | abnormal body height | MP:0001253 | EFEMP1 | Efemp1 |
| Height | abnormal body height | MP:0001253 | GDF5 | Gdf5 |
| Height | abnormal body height | MP:0001253 | GNA12 | Gna12 |
| Height | abnormal body height | MP:0001253 | GPR126 | Gpr126 |
| Height | abnormal body height | MP:0001253 | HHIP | Hhip |
| Height | abnormal body height | MP:0001253 | HIST1H1D | Hist1h1d |
| Height | abnormal body height | MP:0001253 | HMGA2 | Hmga2 |
| Height | abnormal body height | MP:0001253 | JAZF1 | Jazf1 |
| Height | abnormal body height | MP:0001253 | LCORL | Lcorl |
| Height | abnormal body height | MP:0001253 | PLAG1 | Plag1 |
| Height | abnormal body height | MP:0001253 | PTCH1 | Ptch1 |
| Height | abnormal body height | MP:0001253 | SOCS2 | Socs2 |
| Height | abnormal body height | MP:0001253 | SPAG17 | Spag17 |
| Height | abnormal body height | MP:0001253 | UQCC | Uqcc |
| Height | abnormal body height | MP:0001253 | UQCC | Uqcc |
| Height | abnormal body height | MP:0001253 | ZBTB38 | Zbtb38 |
| Height | abnormal body height | MP:0001253 | LOC387103 | |
| Height | abnormal body height | MP:0001253 | ZNF678 |  |
| Obesity related phenotypes | Abnormal body weight | MP:0001259 | FTO | Fto |
| Obesity related phenotypes | Abnormal body weight | MP:0001259 | GNPDA2 | Gnpda2 |
| Obesity related phenotypes | Abnormal body weight | MP:0001259 | MAF | Maf |
| Obesity related phenotypes | Abnormal body weight | MP:0001259 | MTCH2 | Mtch2 |
| Obesity related phenotypes | Abnormal body weight | MP:0001259 | NEGR1 | Negr1 |
| Obesity related phenotypes | Abnormal body weight | MP:0001259 | SH2B1 | Sh2b1 |
| Obesity related phenotypes | Abnormal body weight | MP:0001259 | TMEM18 | Tmem18 |
| Obesity related phenotypes | Abnormal body weight | MP:0001259 | TRHR | Trhr |
| Obesity related phenotypes | Abnormal body weight | MP:0001259 | C12orf51 |  |
| Bilirubin levels | abnormal circulating bilirubin level | MP:0001569 | SLCO1B3 | Slco1b2 |
| pulse rate | abnormal heart rate | MP:0001629 | LOC644502 | |
| longevity | extended life span | MP:0001661 | DPT | Dpt |
| Serum markers of iron status | Abnormal Iron level | MP:0001770 | PAFAH1B2 | Pafah1b2 |
| Inflammatory bowel disease | intestinal inflammation | MP:0001858 | C11orf30 | 2210018M11Rik |
| Inflammatory bowel disease | intestinal inflammation | MP:0001858 | ATG16L1 | Atg16l1 |
| Inflammatory bowel disease | intestinal inflammation | MP:0001858 | CCR6 | Ccr6 |
| Inflammatory bowel disease | intestinal inflammation | MP:0001858 | IL23R | Il23r |
| Inflammatory bowel disease | intestinal inflammation | MP:0001858 | IRGM | Irgm1 |
| Inflammatory bowel disease | intestinal inflammation | MP:0001858 | ITLN1 | Itln1 |
| Inflammatory bowel disease | intestinal inflammation | MP:0001858 | JAK2 | Jak2 |
| Inflammatory bowel disease | intestinal inflammation | MP:0001858 | MST1 | Mst1 |
| Inflammatory bowel disease | intestinal inflammation | MP:0001858 | NKX2-3 | Nkx2-3 |
| Inflammatory bowel disease | intestinal inflammation | MP:0001858 | ORMDL3 | Ormdl3 |
| Inflammatory bowel disease | intestinal inflammation | MP:0001858 | PSMG1 | Psmg1 |
| Inflammatory bowel disease | intestinal inflammation | MP:0001858 | PTGER4 | Ptger4 |
| Inflammatory bowel disease | intestinal inflammation | MP:0001858 | PTPN2 | Ptpn2 |
| Inflammatory bowel disease | intestinal inflammation | MP:0001858 | RNF186 | Rnf186 |
| Inflammatory bowel disease | intestinal inflammation | MP:0001858 | STAT3 | Stat3 |
| Inflammatory bowel disease | intestinal inflammation | MP:0001858 | TNFSF15 | Tnfsf15 |
| Inflammatory bowel disease | intestinal inflammation | MP:0001858 | ZNF365 | Zfp365 |
| Inflammatory bowel disease | intestinal inflammation | MP:0001858 | TNFRSF6B | |
| Neuroblastoma | neuroblastoma | MP:0002039 | BARD1 | Bard1 |
| Skin/hair/eye color related phenotypes | abnormal coat/hair pigmentation - abnormal skin pigmentation | MP:0002075 - MP:0002095 | ASIP | a |
| Skin/hair/eye color related phenotypes | abnormal coat/hair pigmentation - abnormal skin pigmentation | MP:0002075 - MP:0002095 | EXOC2 | Exoc2 |
| Skin/hair/eye color related phenotypes | abnormal coat/hair pigmentation - abnormal skin pigmentation | MP:0002075 - MP:0002095 | GRM5 | Grm5 |
| Skin/hair/eye color related phenotypes | abnormal coat/hair pigmentation - abnormal skin pigmentation | MP:0002075 - MP:0002095 | HERC2 | Herc2 |
| Skin/hair/eye color related phenotypes | abnormal coat/hair pigmentation - abnormal skin pigmentation | MP:0002075 - MP:0002095 | IRF4 | Irf4 |
| Skin/hair/eye color related phenotypes | abnormal coat/hair pigmentation - abnormal skin pigmentation | MP:0002075 - MP:0002095 | KITLG | Kitl |
| Skin/hair/eye color related phenotypes | abnormal coat/hair pigmentation - abnormal skin pigmentation | MP:0002075 - MP:0002095 | SLC24A4 | Slc24a4 |
| Skin/hair/eye color related phenotypes | abnormal coat/hair pigmentation - abnormal skin pigmentation | MP:0002075 - MP:0002095 | SLC24A5 | Slc24a5 |
| Skin/hair/eye color related phenotypes | abnormal coat/hair pigmentation - abnormal skin pigmentation | MP:0002075 - MP:0002095 | TPCN2 | Tpcn2 |
| Lipid phenotypes | Abnormal lipid homeostasis | MP:0002118 | ANGPTL3 | Angptl3 |
| Lipid phenotypes | Abnormal lipid homeostasis | MP:0002118 | CELSR2 | Celsr2 |
| Lipid phenotypes | Abnormal lipid homeostasis | MP:0002118 | DNAH11 | Dnahc11 |
| Lipid phenotypes | Abnormal lipid homeostasis | MP:0002118 | DOCK7 | Dock7 |
| Lipid phenotypes | Abnormal lipid homeostasis | MP:0002118 | GALNT2 | Galnt2 |
| Lipid phenotypes | Abnormal lipid homeostasis | MP:0002118 | MAFB | Mafb |
| Lipid phenotypes | Abnormal lipid homeostasis | MP:0002118 | MLXIPL | Mlxipl |
| Lipid phenotypes | Abnormal lipid homeostasis | MP:0002118 | NCAN | Ncan |
| Lipid phenotypes | Abnormal lipid homeostasis | MP:0002118 | PLEK | Plek |
| Lipid phenotypes | Abnormal lipid homeostasis | MP:0002118 | TMEM57 | Tmem57 |
| Lipid phenotypes | Abnormal lipid homeostasis | MP:0002118 | TRIB1 | Trib1 |
| Lipid phenotypes | Abnormal lipid homeostasis | MP:0002118 | TTC39B | Ttc39b |
| Lipid phenotypes | Abnormal lipid homeostasis | MP:0002118 | ANKRD30A | |
| Renal function and chronic kidney disease | abnormal kidney physiology | MP:0002136 | SHROOM3 | Shroom3 |
| Renal function and chronic kidney disease | abnormal kidney physiology | MP:0002136 | UMOD | Umod |
| Pulmonary function measures | abnormal forced expiratory flow rates | MP:0002297 | HHIP | Hhip |
| Asthma | Abnormal Bronchial Provocation | MP:0002330 | ORMDL3 | Ormdl3 |
| CRP concentration | abnormal C-reactive protein physiology | MP:0002484 | GCKR | Gckr |
| CRP concentration | abnormal C-reactive protein physiology | MP:0002484 | HNF1A | Hnf1a |
| CRP concentration | abnormal C-reactive protein physiology | MP:0002484 | LEF1 | Lef1 |
| CRP concentration | abnormal C-reactive protein physiology | MP:0002484 | LEPR | Lepr |
| Mean platelet volume | abnormal platelet volume | MP:0002586 | ARHGEF3 | Arhgef3 |
| Mean platelet volume | abnormal platelet volume | MP:0002586 | TAOK1 | Taok1 |
| Mean platelet volume | abnormal platelet volume | MP:0002586 | WDR66 | Wdr66 |
| Plasma eosinophil count | abnormal eosinophil cell number | MP:0002602 | GATA2 | Gata2 |
| Plasma eosinophil count | abnormal eosinophil cell number | MP:0002602 | IKZF2 | Ikzf2 |
| Plasma eosinophil count | abnormal eosinophil cell number | MP:0002602 | IL1RL1 | Il1rl1 |
| Plasma eosinophil count | abnormal eosinophil cell number | MP:0002602 | SH2B3 | Sh2b3 |
| Gallstones | gallstones | MP:0002830 | ABCG8 |  |
| Intracranial aneurysm | Aneurysm | MP:0003279 | SOX17 | Sox17 |
| Alzheimer's disease | amyloid beta deposits - neurofibrillary tangles | MP:0003329 - MP:0003214 | GAB2 | Gab2 |
| Menarche and/or menopause (age at onset) | late onset of menarche | MP:0003377 | BRSK1 | Brsk1 |
| Menarche and/or menopause (age at onset) | late onset of menarche | MP:0003377 | LIN28B | Lin28b |
| Menarche and/or menopause (age at onset) | late onset of menarche | MP:0003377 | MCM8 | Mcm8 |
| Menarche and/or menopause (age at onset) | late onset of menarche | MP:0003377 | UIMC1 | Uimc1 |
| Thyroid cancer | thyroid adenoma | MP:0003496 | FOXE1 | Foxe1 |
| Thyroid cancer | thyroid adenoma | MP:0003496 | NKX2-1 | Nkx2-1 |
| Rheumatoid arthritis | rheumatoid arthritis | MP:0003561 | CD40 | Cd40 |
| Rheumatoid arthritis | rheumatoid arthritis | MP:0003561 | TRAF1 | Traf1 |
| QT interval | abnormal QT interval | MP:0003899 | ATP1B1 | Atp1b1 |
| QT interval | abnormal QT interval | MP:0003899 | LITAF | Litaf |
| QT interval | abnormal QT interval | MP:0003899 | NDRG4 | Ndrg4 |
| QT interval | abnormal QT interval | MP:0003899 | NOS1AP | Nos1ap |
| QT interval | abnormal QT interval | MP:0003899 | RNF207 | Rnf207 |
| Basal cell carcinoma (cutaneous) | basal cell carcinoma | MP:0004208 | RHOU | Rhou |
| Systemic lupus erythematosus | increased susceptibility to systemic lupus erythematosus | MP:0004801 | BANK1 | Bank1 |
| Systemic lupus erythematosus | increased susceptibility to systemic lupus erythematosus | MP:0004801 | PHRF1 | Phrf1 |
| Systemic lupus erythematosus | increased susceptibility to systemic lupus erythematosus | MP:0004801 | PXK | Pxk |
| Systemic lupus erythematosus | increased susceptibility to systemic lupus erythematosus | MP:0004801 | TNFAIP3 | Tnfaip3 |
| Type 1 diabetes | Increased susceptibility to autoimmune diabetes | MP:0004803 | C6orf173 | 2610036L11Rik |
| Type 1 diabetes | Increased susceptibility to autoimmune diabetes | MP:0004803 | BACH2 | Bach2 |
| Type 1 diabetes | Increased susceptibility to autoimmune diabetes | MP:0004803 | CD69 | Cd69 |
| Type 1 diabetes | Increased susceptibility to autoimmune diabetes | MP:0004803 | CLEC16A | Clec16a |
| Type 1 diabetes | Increased susceptibility to autoimmune diabetes | MP:0004803 | CTSH | Ctsh |
| Type 1 diabetes | Increased susceptibility to autoimmune diabetes | MP:0004803 | ERBB3 | Erbb3 |
| Type 1 diabetes | Increased susceptibility to autoimmune diabetes | MP:0004803 | GLIS3 | Glis3 |
| Type 1 diabetes | Increased susceptibility to autoimmune diabetes | MP:0004803 | IFIH1 | Ifih1 |
| Type 1 diabetes | Increased susceptibility to autoimmune diabetes | MP:0004803 | IL27 | Il27 |
| Type 1 diabetes | Increased susceptibility to autoimmune diabetes | MP:0004803 | C12orf30 | Naa25 |
| Type 1 diabetes | Increased susceptibility to autoimmune diabetes | MP:0004803 | ORMDL3 | Ormdl3 |
| Type 1 diabetes | Increased susceptibility to autoimmune diabetes | MP:0004803 | PRKCQ | Prkcq |
| Type 1 diabetes | Increased susceptibility to autoimmune diabetes | MP:0004803 | PTPN2 | Ptpn2 |
| Type 1 diabetes | Increased susceptibility to autoimmune diabetes | MP:0004803 | C10orf59 | Rnls |
| Type 1 diabetes | Increased susceptibility to autoimmune diabetes | MP:0004803 | SH2B3 | Sh2b3 |
| Type 1 diabetes | Increased susceptibility to autoimmune diabetes | MP:0004803 | UBASH3A | Ubash3a |
| Type 2 diabetes | insulin resistance | MP:0005331 | CDKAL1 | Cdkal1 |
| Type 2 diabetes | insulin resistance | MP:0005331 | FTO | Fto |
| Type 2 diabetes | insulin resistance | MP:0005331 | G6PC2 | G6pc2 |
| Type 2 diabetes | insulin resistance | MP:0005331 | HHEX | Hhex |
| Type 2 diabetes | insulin resistance | MP:0005331 | IGF2BP2 | Igf2bp2 |
| Type 2 diabetes | insulin resistance | MP:0005331 | JAZF1 | Jazf1 |
| Type 2 diabetes | insulin resistance | MP:0005331 | KCNQ1 | Kcnq1 |
| Type 2 diabetes | insulin resistance | MP:0005331 | MTNR1B | Mtnr1b |
| Type 2 diabetes | insulin resistance | MP:0005331 | SLC30A8 | Slc30a8 |
| Type 2 diabetes | insulin resistance | MP:0005331 | THADA | Thada |
| Coronary disease | atherosclerotic lesions | MP:0005338 | MIA3 | Mia3 |
| Coronary disease | atherosclerotic lesions | MP:0005338 | MRAS | Mras |
| Coronary disease | atherosclerotic lesions | MP:0005338 | PHACTR1 | Phactr1 |
| Coronary disease | atherosclerotic lesions | MP:0005338 | PSRC1 | Psrc1 |
| Stroke | CNS ischemia | MP:0006080 | NINJ2 | Ninj2 |
| Stroke | CNS ischemia | MP:0006080 | NR | nr |
| Breast cancer | mammary gland tumor | MP:0006318 | FGFR2 | Fgfr2 |
| Breast cancer | mammary gland tumor | MP:0006318 | C6orf97 | Gm221 |
| Breast cancer | mammary gland tumor | MP:0006318 | LSP1 | Lsp1 |
| Breast cancer | mammary gland tumor | MP:0006318 | MAP3K1 | Map3k1 |
| Breast cancer | mammary gland tumor | MP:0006318 | TNRC9 | Tox3 |
| Breast cancer | mammary gland tumor | MP:0006318 | TOX3 | Tox3 |
| TNFa concentration | abnormal circulating tumor necrosis factor level | MP:0008552 | ABO | Abo |
| Lung cancer | lung carcinoma | MP:0008714 | CLPTM1L | Clptm1l |
| Serum urate/uric acid | abnormal blood uric acid level | MP:0008820 | ABCG2 | Abcg2 |
| Serum urate/uric acid | abnormal blood uric acid level | MP:0008820 | SLC17A3 | Slc17a3 |
| Serum urate/uric acid | abnormal blood uric acid level | MP:0008820 | SLC2A9 | Slc2a9 |
| Prostate cancer | prostate adenocarcinoma | MP:0009220 | EHBP1 | Ehbp1 |
| Prostate cancer | prostate adenocarcinoma | MP:0009220 | HNF1B | Hnf1b |
| Prostate cancer | prostate adenocarcinoma | MP:0009220 | KLK3 | Klkb1 |
| Prostate cancer | prostate adenocarcinoma | MP:0009220 | LMTK2 | Lmtk2 |
| Prostate cancer | prostate adenocarcinoma | MP:0009220 | MSMB | Msmb |
| Prostate cancer | prostate adenocarcinoma | MP:0009220 | SLC22A3 | Slc22a3 |
| Colorectal cancer | large intestine adenocarcinoma | MP:0009310 | BMP4 | Bmp4 |
| Colorectal cancer | large intestine adenocarcinoma | MP:0009310 | EIF3H | Eif3h |
| Colorectal cancer | large intestine adenocarcinoma | MP:0009310 | RHPN2 | Rhpn2 |
| Colorectal cancer | large intestine adenocarcinoma | MP:0009310 | SMAD7 | Smad7 |
| Chronic lymphocytic leukemia | small lymphocytic lymphoma | MP:0009319 | GRAMD1B | Gramd1b |
| Chronic lymphocytic leukemia | small lymphocytic lymphoma | MP:0009319 | IRF4 | Irf4 |
| Bone mineral density | Abnormal Bone Mineral Density | MP:0010119 | FAM3C | Fam3c |
| **Associations proposed in the candidate gene era** | | | | |
| Serum markers of iron status | Abnormal Iron level | MP:0001770 | HFE | Hfe |
| Serum markers of iron status | Abnormal Iron level | MP:0001770 | TF | Trf |
| Obesity related phenotypes | Abnormal body weight | MP:0001259 | BDNF | Bdnf |
| Obesity related phenotypes | Abnormal body weight | MP:0001259 | MC4R | Mc4r |
| Obesity related phenotypes | Abnormal body weight | MP:0001259 | CETP | Tg(CETP)5203Tall |
| Bone mineral density | Abnormal Bone Mineral Density | MP:0010119 | ESR1 | Esr1 |
| Bone mineral density | Abnormal Bone Mineral Density | MP:0010119 | LRP5 | Lrp5 |
| Bone mineral density | Abnormal Bone Mineral Density | MP:0010119 | OPG | Tnfrsf11b |
| Bone mineral density | Abnormal Bone Mineral Density | MP:0010119 | TNFRSF11B | Tnfrsf11b |
| Bone mineral density | Abnormal Bone Mineral Density | MP:0010119 | RANKL | Tnfsf11 |
| Bone mineral density | Abnormal Bone Mineral Density | MP:0010119 | ZBTB40 | Zbtb40 |
| Bilirubin levels | abnormal circulating bilirubin level | MP:0001569 | UGT1A1 | Ugt1a1 |
| IL-18 concentration | abnormal circulating interleukin-18 level | MP:0008634 | IL18 | Il18 |
| IL-6sR concentration | abnormal circulating interleukin-6 level | MP:0008595 | IL6R | Il6ra |
| Skin/hair/eye color related phenotypes | abnormal coat/hair pigmentation - abnormal skin pigmentation | MP:0002075 - MP:0002095 | MC1R | Mc1r |
| Skin/hair/eye color related phenotypes | abnormal coat/hair pigmentation - abnormal skin pigmentation | MP:0002075 - MP:0002095 | OCA2 | Oca2 |
| Skin/hair/eye color related phenotypes | abnormal coat/hair pigmentation - abnormal skin pigmentation | MP:0002075 - MP:0002095 | SLC45A2 | Slc45a2 |
| Skin/hair/eye color related phenotypes | abnormal coat/hair pigmentation - abnormal skin pigmentation | MP:0002075 - MP:0002095 | TYR | Tyr |
| Skin/hair/eye color related phenotypes | abnormal coat/hair pigmentation - abnormal skin pigmentation | MP:0002075 - MP:0002095 | TYRP1 | Tyrp1 |
| CRP concentration | abnormal C-reactive protein physiology | MP:0002484 | APOE | Apoe |
| CRP concentration | abnormal C-reactive protein physiology | MP:0002484 | CRP | Crp |
| Plasma eosinophil count | abnormal eosinophil cell number | MP:0002602 | IL5 | Il5 |
| Lipid phenotypes | Abnormal lipid homeostasis | MP:0002118 | ABCA1 | Abca1 |
| Lipid phenotypes | Abnormal lipid homeostasis | MP:0002118 | ABCG5 | Abcg5 |
| Lipid phenotypes | Abnormal lipid homeostasis | MP:0002118 | ABCG8 | Abcg8 |
| Lipid phenotypes | Abnormal lipid homeostasis | MP:0002118 | APOB | Apob |
| Lipid phenotypes | Abnormal lipid homeostasis | MP:0002118 | APOC1 | Apoc1 |
| Lipid phenotypes | Abnormal lipid homeostasis | MP:0002118 | GCKR | Gckr |
| Lipid phenotypes | Abnormal lipid homeostasis | MP:0002118 | HMGCR | Hmgcr |
| Lipid phenotypes | Abnormal lipid homeostasis | MP:0002118 | HNF4A | Hnf4a |
| Lipid phenotypes | Abnormal lipid homeostasis | MP:0002118 | LCAT | Lcat |
| Lipid phenotypes | Abnormal lipid homeostasis | MP:0002118 | LDLR | Ldlr |
| Lipid phenotypes | Abnormal lipid homeostasis | MP:0002118 | LIPC | Lipc |
| Lipid phenotypes | Abnormal lipid homeostasis | MP:0002118 | LIPG | Lipg |
| Lipid phenotypes | Abnormal lipid homeostasis | MP:0002118 | LPL | Lpl |
| Lipid phenotypes | Abnormal lipid homeostasis | MP:0002118 | PCSK9 | Pcsk9 |
| Lipid phenotypes | Abnormal lipid homeostasis | MP:0002118 | PLTP | Pltp |
| Lipid phenotypes | Abnormal lipid homeostasis | MP:0002118 | CETP | Tg(CETP)5203Tall |
| Lipid phenotypes | Abnormal lipid homeostasis | MP:0002118 | LPA |  |
| QT interval | abnormal QT interval | MP:0003899 | KCNH2 | Kcnh2 |
| QT interval | abnormal QT interval | MP:0003899 | KCNJ2 | Kcnj2 |
| QT interval | abnormal QT interval | MP:0003899 | KCNQ1 | Kcnq1 |
| QT interval | abnormal QT interval | MP:0003899 | PLN | Pln |
| QT interval | abnormal QT interval | MP:0003899 | SCN5A | Scn5a |
| Male-pattern baldness | alopecia | MP:0000414 | AR | Ar |
| Alzheimer's disease | amyloid beta deposits - neurofibrillary tangles | MP:0003329 - MP:0003214 | APOE |  |
| Coronary disease | atherosclerotic lesions | MP:0005338 | CXCL12 | Cxcl12 |
| Coronary disease | atherosclerotic lesions | MP:0005338 | LDLR | Ldlr |
| Multiple sclerosis | demyelination | MP:0000921 | HLA-DRA | H2-Ea |
| Multiple sclerosis | demyelination | MP:0000921 | HLA-DRB1 | H2-Eb1 |
| Serum IgE levels | increased IgE level | MP:0002497 | FCER1A | Fcer1a |
| Systemic lupus erythematosus | increased susceptibility to systemic lupus erythematosus | MP:0004801 | HLA-DQA1 | H2-Aa |
| Systemic lupus erythematosus | increased susceptibility to systemic lupus erythematosus | MP:0004801 | STAT4 | Stat4 |
| Type 1 diabetes | Increased susceptibility to autoimmune diabetes | MP:0004803 | CTLA4 | Ctla4 |
| Type 1 diabetes | Increased susceptibility to autoimmune diabetes | MP:0004803 | HLA-E | H2-T23 |
| Type 1 diabetes | Increased susceptibility to autoimmune diabetes | MP:0004803 | IL10 | Il10 |
| Type 1 diabetes | Increased susceptibility to autoimmune diabetes | MP:0004803 | IL2 | Il2 |
| Type 1 diabetes | Increased susceptibility to autoimmune diabetes | MP:0004803 | IL2RA | Il2ra |
| Type 1 diabetes | Increased susceptibility to autoimmune diabetes | MP:0004803 | INS | Ins2 |
| Type 1 diabetes | Increased susceptibility to autoimmune diabetes | MP:0004803 | PTPN22 | Ptpn22 |
| Type 2 diabetes | insulin resistance | MP:0005331 | GCK | Gck |
| Type 2 diabetes | insulin resistance | MP:0005331 | KCNJ11 | Kcnj11 |
| Type 2 diabetes | insulin resistance | MP:0005331 | TCF7L2 | Tcf7l2 |
| Inflammatory bowel disease | intestinal inflammation | MP:0001858 | CDKAL1 | Cdkal1 |
| Inflammatory bowel disease | intestinal inflammation | MP:0001858 | ICOSLG | Icosl |
| Inflammatory bowel disease | intestinal inflammation | MP:0001858 | IL10 | Il10 |
| Inflammatory bowel disease | intestinal inflammation | MP:0001858 | IL12B | Il12b |
| Inflammatory bowel disease | intestinal inflammation | MP:0001858 | NOD2 | Nod2 |
| Lung cancer | lung carcinoma | MP:0008714 | NR | nr |
| Myopathy | myopathy | MP:0000751 | SLCO1B1 |  |
| Psoriasis | psoriasis | MP:0001193 | HLA-C | H2-K1 |
| Psoriasis | psoriasis | MP:0001193 | IL12B | Il12b |
| Psoriasis | psoriasis | MP:0001193 | IL13 | Il13 |
| Wet age-related macular degeneration | retinal cone cell degeneration | MP:0008444 | HTRA1 | Htra1 |
| Rheumatoid arthritis | rheumatoid arthritis | MP:0003561 | HLA-DRB1 | H2-Eb1 |
| Rheumatoid arthritis | rheumatoid arthritis | MP:0003561 | PTPN22 | Ptpn22 |
| Creutzfeldt-Jakob disease | spongiform encephalopathy | MP:0002654 | PRNP | Prnp |
| Venous thromboembolism | thrombosis | MP:0005048 | ABO | Abo |
|  |  |  |  |  |
