# Supplementary material for: Laboratory Mouse Models for the Human Genome-Wide Associations
Source: PLoS One. 2010 Nov 1;5(11):e13782. doi: 10.1371/journal.pone.0013782 (PMC2967475; doi:10.1371/journal.pone.0013782)
Supplement: Table S7 — Comparisons of the sets of orthologs associated with the same phenotype in humans and mice (considering only the novel GWAS associations). (0.14 MB DOC) [file pone.0013782.s007.doc]

| **disease** | **MP term** | **MP id** | **Anatomical system** | **N of mouse models** | **N of mouse genes** | **N of human genes** | **Human Genes** | **N of concordances** | **Concordant genes with humans** |
| --- | --- | --- | --- | --- | --- | --- | --- | --- | --- |
| Inflammatory bowel disease | intestinal inflammation | MP:0001858 | immune system | 57 | 42 | 18 | ATG16L1, C11orf30, CCR6, IL23R, IRGM, ITLN1, JAK2, MST1, NKX2-3, NOD2, ORMDL3, PSMG1, PTGER4, PTPN2, RNF186, STAT3, TNFRSF6B, TNFSF15, ZNF365 | 1 | MST1 |
| Obesity related phenotypes | Abnormal body weight | MP:0001259 | growth size | 969 | 614 | 9 | C12orf51, FTO, GNPDA2, MAF, MTCH2, NEGR1, SH2B1, TMEM18, TRHR | 1 | SH2B1 |
| Prostate cancer | prostate adenocarcinoma | MP:0009220 | tumorigenesis | 20 | 9 | 6 | EHBP1, HNF1B, KLK3, LMTK2, MSMB, SLC22A3 | 1 | MSMB |
| Blood pressure related phenotypes | abnormal blood pressure | MP:0000230 | cardiovascular | 165 | 115 | 6 | ATP2B1, CDH13, CYP17A1, PLEKHA7, SH2B3, ULK4 | 0 |  |
| Male-pattern baldness | alopecia | MP:0000414 | skin/nails | 124 | 91 | 1 | PAX1 | 0 |  |
| Plasma levels of liver enzymes | abnormal liver physiology | MP:0000609 | liver/biliary | 123 | 87 | 4 | ADAMTS13, GPLD1, JMJD1C, REEP3 | 0 |  |
| Essential tremor | tremors | MP:0000745 | nervous | 170 | 143 | 1 | LINGO1 | 0 |  |
| Myopathy | myopathy | MP:0000751 | muscle | 23 | 17 | 0 |  | 0 |  |
| Multiple sclerosis | demyelination | MP:0000921 | nervous | 46 | 34 | 1 | KIF1B | 0 |  |
| Psoriasis | psoriasis | MP:0001193 | skin/nails | 5 | 2 | 2 | TNFAIP3, TNIP1 | 0 |  |
| Atopic dermatitis | dermatitis | MP:0001194 | skin/nails | 61 | 31 | 1 | C11orf30 | 0 |  |
| Height | abnormal body height | MP:0001253 | growth size | 8 | 8 | 22 | BMP6, CDK6, C6orf106, DLEU7, EFEMP1, GDF5, GNA12, GPR126, HHIP, HIST1H1D, HMGA2, JAZF1, LCORL, PLAG1, PTCH1, SOCS2, SPAG17, BFZB, UQCC, ZBTB38, LOC387103, ZNF678 | 0 |  |
| Bilirubin levels | abnormal circulating bilirubin level | MP:0001569 | homeostasis/metabolism | 13 | 13 | 2 | SLCO1B3, UGT1A1 | 0 |  |
| pulse rate | abnormal heart rate | MP:0001629 | cardiovascular | 76 | 64 | 1 | LOC644502 | 0 |  |
| longevity | extended life span | MP:0001661 | life span/aging | 21 | 15 | 1 | DPT | 0 |  |
| Serum markers of iron status | Abnormal Iron level | MP:0001770 | homeostasis/metabolism | 19 | 9 | 1 | PAFAH1B2 | 0 |  |
| Neuroblastoma | neuroblastoma | MP:0002039 | tumorigenesis | 2 | 2 | 1 | BARD1 | 0 |  |
| Skin/hair/eye color related phenotypes | abnormal coat/hair pigmentation - abnormal skin pigmentation | MP:0002075 - MP:0002095 | skin/nails | 385 | 162 | 9 | ASIP, EXOC2, GRM5, HERC2, IRF4, KITLG, SLC24A4, SLC45A2, TPCN2 | 0 |  |
| Lipid phenotypes | Abnormal lipid homeostasis | MP:0002118 | homeostasis/metabolism | 581 | 280 | 13 | ANGPTL3, ANKRD30A, CELSR2, DNAH11, DOCK7, GALNT2, MAFB, MLXIPL, NCAN, PLEK, TMEM57, TRIB1, TTC39B | 0 |  |
| Renal function and chronic kidney disease | abnormal kidney physiology | MP:0002136 | renal/urinary | 62 | 48 | 2 | SHROOM3, UMOD | 0 |  |
| Pulmonary function measures | abnormal forced expiratory flow rates | MP:0002297 | respiratory | 0 | 0 | 1 | HHIP | 0 |  |
| Asthma | Abnormal Bronchial Provocation | MP:0002330 | respiratory | 27 | 23 | 1 | ORMDL3 | 0 |  |
| CRP concentration | abnormal C-reactive protein physiology | MP:0002484 | immune system | 1 | 1 | 4 | GCKR, HNF1A, LEF1, LEPR | 0 |  |
| Serum IgE levels | increased IgE level | MP:0002497 | immune system | 42 | 36 | 1 | FCER1A | 0 |  |
| Mean platelet volume | abnormal platelet volume | MP:0002586 | hematopoietic | 8 | 6 | 3 | ARHGEF3, TAOK1, WDR66 | 0 |  |
| Plasma eosinophil count | abnormal eosinophil cell number | MP:0002602 | immune system | 2 | 2 | 4 | GATA2, IKZF2, IL1RL1, SH2B3 | 0 |  |
| Creutzfeldt-Jakob disease | spongiform encephalopathy | MP:0002654 | nervous | 8 | 6 | 0 |  | 0 |  |
| Gallstones | gallstones | MP:0002830 | liver/biliary | 47 | 26 | 1 | ABCG8 | 0 |  |
| Intracranial aneurysm | Aneurysm | MP:0003279 | cardiovascular | 11 | 11 | 1 | SOX17 | 0 |  |
| Alzheimer's disease | amyloid beta deposits - neurofibrillary tangles | MP:0003329 - MP:0003214 | nervous | 14 | 7 | 1 | GAB2 | 0 |  |
| Menarche and/or menopause (age at onset) | late onset of menarche | MP:0003377 | reproductive | 1 | 1 | 4 | BRSK1, LIN28B, MCM8, UIMC1 | 0 |  |
| Thyroid cancer | thyroid adenoma | MP:0003496 | tumorigenesis | 1 | 1 | 2 | FOXE1, NKX2-1 | 0 |  |
| Rheumatoid arthritis | rheumatoid arthritis | MP:0003561 | immune system | 2 | 2 | 4 | CD40, HLA-DRB1, PTPN22, TRAF1 | 0 |  |
| QT interval | abnormal QT interval | MP:0003899 | cardiovascular | 9 | 6 | 5 | TF, ATP1B1, LITAF, NDRG4, NOS1AP, RNF207 | 0 |  |
| Basal cell carcinoma (cutaneous) | basal cell carcinoma | MP:0004208 | tumorigenesis | 7 | 4 | 1 | RHOU | 0 |  |
| Systemic lupus erythematosus | increased susceptibility to systemic lupus erythematosus | MP:0004801 | immune system | 27 | 18 | 4 | BANK1, PHRF1, PXK, TNFAIP3 | 0 |  |
| Type 1 diabetes | Increased susceptibility to autoimmune diabetes | MP:0004803 | immune system | 81 | 34 | 16 | BACH2, C10orf59, C12orf30, C6orf173, CD69, CLEC16A, CLEC16A ,CTSH, ERBB3, GLIS3, IFIH1, IL27, ORMDL3, PRKCQ, PTPN2, SH2B3, UBASH3A | 0 |  |
| Venous thromboembolism | thrombosis | MP:0005048 | homeostasis/metabolism | 28 | 23 | 0 | ABO | 0 |  |
| Type 2 diabetes | insulin resistance | MP:0005331 | homeostasis/metabolism | 64 | 44 | 10 | CDKAL1, FTO, G6PC2, HHEX, IGF2BP2, JAZF1, KCNQ1, MTNR1B, SLC30A8, THADA | 0 |  |
| Coronary disease | atherosclerotic lesions | MP:0005338 | cardiovascular | 51 | 35 | 4 | MIA3, MRAS, PHACTR1, PSRC1 | 0 |  |
| Stroke | CNS ischemia | MP:0006080 | nervous | 1 | 1 | 2 | NINJ2, NR | 0 |  |
| Breast cancer | mammary gland tumor | MP:0006318 | tumorigenesis | 44 | 20 | 6 | C6orf97, FGFR2, LSP1, MAP3K1, TNRC9, TOX3, ORMDL3 | 0 |  |
| Wet age-related macular degeneration | retinal cone cell degeneration | MP:0008444 | nervous | 5 | 5 | 0 |  | 0 |  |
| TNFa concentration | abnormal circulating tumor necrosis factor level | MP:0008552 | immune system | 26 | 22 | 1 | ABO | 0 |  |
| IL-6sR concentration | abnormal circulating interleukin-6 level | MP:0008595 | immune system | 19 | 18 | 0 |  | 0 |  |
| IL-18 concentration | abnormal circulating interleukin-18 level | MP:0008634 | immune system | 2 | 2 | 0 |  | 0 |  |
| Lung cancer | lung carcinoma | MP:0008714 | tumorigenesis | 46 | 29 | 1 | CLPTM1L | 0 |  |
| Serum urate/uric acid | abnormal blood uric acid level | MP:0008820 | homeostasis/metabolism | 1 | 1 | 3 | ABCG2, SLC17A3, SLC2A9 | 0 |  |
| Colorectal cancer | large intestine adenocarcinoma | MP:0009310 | tumorigenesis | 5 | 5 | 4 | BMP4, EIF3H, RHPN2, SMAD7 | 0 |  |
| Chronic lymphocytic leukemia | small lymphocytic lymphoma | MP:0009319 | tumorigenesis | 2 | 1 | 2 | GRAMD1B, IRF4 | 0 |  |
| Bone mineral density | Abnormal Bone Mineral Density | MP:0010119 | skeleton | 185 | 82 | 2 | FAM3C, ZBTB40 | 0 |  |
