# Supplementary material for: Laboratory Mouse Models for the Human Genome-Wide Associations
Source: PLoS One. 2010 Nov 1;5(11):e13782. doi: 10.1371/journal.pone.0013782 (PMC2967475; doi:10.1371/journal.pone.0013782)
Supplement: Table S8 — Detailed phenotypic expression of all knocked out orthologs. (4.11 MB DOC) [file pone.0013782.s008.doc]

| **Ortholog** | **Knockout Model** | **Observed Phenotypes** | **MP accession number** |
| --- | --- | --- | --- |
| [Hmga2](http://www.informatics.jax.org/javawi2/servlet/WIFetch?page=markerDetail&id=MGI:101761) | MGI:2386412 | [shortened head](http://www.informatics.jax.org/searches/Phat.cgi?id=MP:0000435) | MP:0000435 |
|  |  | [abnormal body weight](http://www.informatics.jax.org/searches/Phat.cgi?id=MP:0001259) | MP:0001259 |
|  |  | [decreased body weight](http://www.informatics.jax.org/searches/Phat.cgi?id=MP:0001262) | MP:0001262 |
|  |  | decreased body size | MP:0001265 |
|  |  | [male infertility](http://www.informatics.jax.org/searches/Phat.cgi?id=MP:0001925) | MP:0001925 |
|  |  | [azoospermia](http://www.informatics.jax.org/searches/Phat.cgi?id=MP:0005159) | MP:0005159 |
|  |  | [abnormal fat pad morphology](http://www.informatics.jax.org/searches/Phat.cgi?id=MP:0005334) | MP:0005334 |
|  |  | [increased resistance to diet-induced obesity](http://www.informatics.jax.org/searches/Phat.cgi?id=MP:0005659) | MP:0005659 |
|  |  | [abnormal spermatogonia morphology](http://www.informatics.jax.org/searches/Phat.cgi?id=MP:0006378) | MP:0006378 |
|  |  | [decreased inguinal fat pad weight](http://www.informatics.jax.org/searches/Phat.cgi?id=MP:0009293) | MP:0009293 |
|  |  | [decreased mesenteric fat pad weight](http://www.informatics.jax.org/searches/Phat.cgi?id=MP:0009299) | MP:0009299 |
|  |  | [decreased birth weight](http://www.informatics.jax.org/searches/Phat.cgi?id=MP:0009674) | MP:0009674 |
| [Traf1](http://www.informatics.jax.org/javawi2/servlet/WIFetch?page=markerDetail&id=MGI:101836) | MGI:2386957 | [increased lymphocyte cell number](http://www.informatics.jax.org/searches/Phat.cgi?id=MP:0005013) | MP:0005013 |
|  |  | [increased T cell proliferation](http://www.informatics.jax.org/searches/Phat.cgi?id=MP:0005348) | MP:0005348 |
|  |  | [abnormal lymph node cell ratio](http://www.informatics.jax.org/searches/Phat.cgi?id=MP:0008828) | MP:0008828 |
|  |  | [increased sensitivity to induced cell death](http://www.informatics.jax.org/searches/Phat.cgi?id=MP:0008943) | MP:0008943 |
| [Umod](http://www.informatics.jax.org/javawi2/servlet/WIFetch?page=markerDetail&id=MGI:102674) | MGI:3574985 | [polyuria](http://www.informatics.jax.org/searches/Phat.cgi?id=MP:0001762) | MP:0001762 |
|  |  | [abnormal juxtaglomerular apparatus](http://www.informatics.jax.org/searches/Phat.cgi?id=MP:0002829) | MP:0002829 |
|  |  | [abnormal urine osmolality](http://www.informatics.jax.org/searches/Phat.cgi?id=MP:0002987) | MP:0002987 |
|  |  | [abnormal loop of Henle](http://www.informatics.jax.org/searches/Phat.cgi?id=MP:0004755) | MP:0004755 |
|  |  | [decreased renal glomerular filtration rate](http://www.informatics.jax.org/searches/Phat.cgi?id=MP:0005528) | MP:0005528 |
|  |  | [decreased creatinine clearance](http://www.informatics.jax.org/searches/Phat.cgi?id=MP:0005558) | MP:0005558 |
|  | MGI:3526897 | [increased susceptibility to bacterial infection](http://www.informatics.jax.org/searches/Phat.cgi?id=MP:0002412) | MP:0002412 |
|  |  | [nephrocalcinosis](http://www.informatics.jax.org/searches/Phat.cgi?id=MP:0003197) | MP:0003197 |
|  |  | [renal fibrosis](http://www.informatics.jax.org/searches/Phat.cgi?id=MP:0003985) | MP:0003985 |
|  |  | [dilated kidney collecting duct](http://www.informatics.jax.org/searches/Phat.cgi?id=MP:0004860) | MP:0004860 |
|  |  | [increased urine sodium level](http://www.informatics.jax.org/searches/Phat.cgi?id=MP:0006316) | MP:0006316 |
|  |  | [abnormal urine homeostasis](http://www.informatics.jax.org/searches/Phat.cgi?id=MP:0009643) | MP:0009643 |
|  |  | [crystalluria](http://www.informatics.jax.org/searches/Phat.cgi?id=MP:0009645) | MP:0009645 |
|  |  | [increased urine uric acid level](http://www.informatics.jax.org/searches/Phat.cgi?id=MP:0009810) | MP:0009810 |
| [Stat3](http://www.informatics.jax.org/javawi2/servlet/WIFetch?page=markerDetail&id=MGI:103038) | MGI:2673309 | [increased susceptibility to endotoxin shock](http://www.informatics.jax.org/searches/Phat.cgi?id=MP:0008735) | MP:0008735 |
|  | MGI:3040152 | [perinatal lethality](http://www.informatics.jax.org/searches/Phat.cgi?id=MP:0002081) | MP:0002081 |
|  |  | [abnormal macrophage physiology](http://www.informatics.jax.org/searches/Phat.cgi?id=MP:0002451) | MP:0002451 |
|  |  | [increased tumor necrosis factor secretion](http://www.informatics.jax.org/searches/Phat.cgi?id=MP:0008560) | MP:0008560 |
|  |  | [increased interleukin-6 secretion](http://www.informatics.jax.org/searches/Phat.cgi?id=MP:0008705) | MP:0008705 |
|  | MGI:3040161 | [abnormal macrophage physiology](http://www.informatics.jax.org/searches/Phat.cgi?id=MP:0002451) | MP:0002451 |
|  | MGI:3768533 | [increased neutrophil cell number](http://www.informatics.jax.org/searches/Phat.cgi?id=MP:0000219) | MP:0000219 |
|  |  | [increased monocyte cell number](http://www.informatics.jax.org/searches/Phat.cgi?id=MP:0000220) | MP:0000220 |
|  |  | [decreased hepatocyte proliferation](http://www.informatics.jax.org/searches/Phat.cgi?id=MP:0004001) | MP:0004001 |
|  |  | [increased sensitivity to induced morbidity/mortality](http://www.informatics.jax.org/searches/Phat.cgi?id=MP:0009763) | MP:0009763 |
|  | MGI:1926814 | [abnormal motor neuron morphology](http://www.informatics.jax.org/searches/Phat.cgi?id=MP:0000937) | MP:0000937 |
|  |  | [absent mesoderm](http://www.informatics.jax.org/searches/Phat.cgi?id=MP:0001683) | MP:0001683 |
|  |  | [failure to gastrulate](http://www.informatics.jax.org/searches/Phat.cgi?id=MP:0001696) | MP:0001696 |
|  |  | [decreased embryo size](http://www.informatics.jax.org/searches/Phat.cgi?id=MP:0001698) | MP:0001698 |
|  |  | [abnormal inner cell mass](http://www.informatics.jax.org/searches/Phat.cgi?id=MP:0002718) | MP:0002718 |
|  |  | [abnormal bone structure](http://www.informatics.jax.org/searches/Phat.cgi?id=MP:0003795) | MP:0003795 |
|  |  | [abnormal neuron physiology](http://www.informatics.jax.org/searches/Phat.cgi?id=MP:0004811) | MP:0004811 |
|  |  | [increased susceptibility to injury](http://www.informatics.jax.org/searches/Phat.cgi?id=MP:0005165) | MP:0005165 |
|  |  | [embryonic lethality before somite formation](http://www.informatics.jax.org/searches/Phat.cgi?id=MP:0006205) | MP:0006205 |
|  |  | [abnormal bone ossification](http://www.informatics.jax.org/searches/Phat.cgi?id=MP:0008271) | MP:0008271 |
|  |  | [abnormal interleukin level](http://www.informatics.jax.org/searches/Phat.cgi?id=MP:0008751) | MP:0008751 |
|  |  | [decreased cancellous bone thickness](http://www.informatics.jax.org/searches/Phat.cgi?id=MP:0009346) | MP:0009346 |
| [Stat4](http://www.informatics.jax.org/javawi2/servlet/WIFetch?page=markerDetail&id=MGI:103062) | MGI:2388732 | [increased susceptibility to autoimmune diabetes](http://www.informatics.jax.org/searches/Phat.cgi?id=MP:0004803) | MP:0004803 |
|  |  | [decreased susceptibility to autoimmune diabetes](http://www.informatics.jax.org/searches/Phat.cgi?id=MP:0004804) | MP:0004804 |
|  |  | [abnormal NK cell physiology](http://www.informatics.jax.org/searches/Phat.cgi?id=MP:0005069) | MP:0005069 |
|  |  | [increased interferon-gamma secretion](http://www.informatics.jax.org/searches/Phat.cgi?id=MP:0008566) | MP:0008566 |
|  |  | [decreased circulating interferon-gamma level](http://www.informatics.jax.org/searches/Phat.cgi?id=MP:0008578) | MP:0008578 |
|  |  | [increased interleukin-4 secretion](http://www.informatics.jax.org/searches/Phat.cgi?id=MP:0008699) | MP:0008699 |
|  |  | [decreased interleukin-4 secretion](http://www.informatics.jax.org/searches/Phat.cgi?id=MP:0008700) | MP:0008700 |
|  | MGI:1857248 | [abnormal circulating glucose level](http://www.informatics.jax.org/searches/Phat.cgi?id=MP:0000188) | MP:0000188 |
|  |  | [abnormal inflammatory response](http://www.informatics.jax.org/searches/Phat.cgi?id=MP:0001845) | MP:0001845 |
|  |  | [altered susceptibility to autoimmune disorder](http://www.informatics.jax.org/searches/Phat.cgi?id=MP:0002425) | MP:0002425 |
|  |  | [abnormal insulin secretion](http://www.informatics.jax.org/searches/Phat.cgi?id=MP:0003564) | MP:0003564 |
|  |  | [abnormal lymphocyte physiology](http://www.informatics.jax.org/searches/Phat.cgi?id=MP:0003945) | MP:0003945 |
|  |  | [insulitis](http://www.informatics.jax.org/searches/Phat.cgi?id=MP:0004031) | MP:0004031 |
|  |  | [decreased susceptibility to autoimmune diabetes](http://www.informatics.jax.org/searches/Phat.cgi?id=MP:0004804) | MP:0004804 |
|  |  | [abnormal NK cell physiology](http://www.informatics.jax.org/searches/Phat.cgi?id=MP:0005069) | MP:0005069 |
|  |  | [abnormal cytotoxic T cell physiology](http://www.informatics.jax.org/searches/Phat.cgi?id=MP:0005078) | MP:0005078 |
|  |  | [abnormal T cell proliferation](http://www.informatics.jax.org/searches/Phat.cgi?id=MP:0005094) | MP:0005094 |
|  |  | [abnormal pancreatic beta cell morphology](http://www.informatics.jax.org/searches/Phat.cgi?id=MP:0005217) | MP:0005217 |
|  |  | [abnormal T-helper 1 physiology](http://www.informatics.jax.org/searches/Phat.cgi?id=MP:0005465) | MP:0005465 |
|  |  | [abnormal T-helper 2 physiology](http://www.informatics.jax.org/searches/Phat.cgi?id=MP:0005466) | MP:0005466 |
|  |  | [abnormal CD4-positive T cell differentiation](http://www.informatics.jax.org/searches/Phat.cgi?id=MP:0008076) | MP:0008076 |
|  |  | [decreased interferon-gamma secretion](http://www.informatics.jax.org/searches/Phat.cgi?id=MP:0008567) | MP:0008567 |
|  |  | [decreased circulating interferon-gamma level](http://www.informatics.jax.org/searches/Phat.cgi?id=MP:0008578) | MP:0008578 |
|  |  | [decreased circulating interleukin-2 level](http://www.informatics.jax.org/searches/Phat.cgi?id=MP:0008600) | MP:0008600 |
|  |  | [abnormal cytokine level](http://www.informatics.jax.org/searches/Phat.cgi?id=MP:0008713) | MP:0008713 |
| [Cxcl12](http://www.informatics.jax.org/javawi2/servlet/WIFetch?page=markerDetail&id=MGI:103556) | MGI:1934384 | [abnormal angiogenesis](http://www.informatics.jax.org/searches/Phat.cgi?id=MP:0000260) | MP:0000260 |
|  |  | [abnormal ventricular septum morphology](http://www.informatics.jax.org/searches/Phat.cgi?id=MP:0000281) | MP:0000281 |
|  |  | [decreased bone marrow cell number](http://www.informatics.jax.org/searches/Phat.cgi?id=MP:0000333) | MP:0000333 |
|  |  | [abnormal cerebellum morphology](http://www.informatics.jax.org/searches/Phat.cgi?id=MP:0000849) | MP:0000849 |
|  |  | [thin external granule cell layer](http://www.informatics.jax.org/searches/Phat.cgi?id=MP:0000873) | MP:0000873 |
|  |  | [impaired myelopoiesis](http://www.informatics.jax.org/searches/Phat.cgi?id=MP:0001602) | MP:0001602 |
|  |  | [neonatal lethality](http://www.informatics.jax.org/searches/Phat.cgi?id=MP:0002058) | MP:0002058 |
|  |  | [abnormal primordial germ cell migration](http://www.informatics.jax.org/searches/Phat.cgi?id=MP:0002982) | MP:0002982 |
|  |  | [lethality throughout fetal growth and development](http://www.informatics.jax.org/searches/Phat.cgi?id=MP:0006208) | MP:0006208 |
|  |  | [decreased pro-B cell number](http://www.informatics.jax.org/searches/Phat.cgi?id=MP:0008208) | MP:0008208 |
|  |  | [decreased pre-B cell number](http://www.informatics.jax.org/searches/Phat.cgi?id=MP:0008209) | MP:0008209 |
| [Ptger4](http://www.informatics.jax.org/javawi2/servlet/WIFetch?page=markerDetail&id=MGI:104311) | MGI:2137850 | [abnormal vascular regression](http://www.informatics.jax.org/searches/Phat.cgi?id=MP:0000364) | MP:0000364 |
|  |  | [abnormal lung morphology](http://www.informatics.jax.org/searches/Phat.cgi?id=MP:0001175) | MP:0001175 |
|  |  | [lung hemorrhage](http://www.informatics.jax.org/searches/Phat.cgi?id=MP:0001182) | MP:0001182 |
|  |  | [postnatal lethality](http://www.informatics.jax.org/searches/Phat.cgi?id=MP:0002082) | MP:0002082 |
|  |  | [abnormal pulmonary circulation](http://www.informatics.jax.org/searches/Phat.cgi?id=MP:0002295) | MP:0002295 |
|  |  | [abnormal macrophage physiology](http://www.informatics.jax.org/searches/Phat.cgi?id=MP:0002451) | MP:0002451 |
|  |  | [abnormal serum amyloid protein physiology](http://www.informatics.jax.org/searches/Phat.cgi?id=MP:0002486) | MP:0002486 |
|  |  | [hepatic steatosis](http://www.informatics.jax.org/searches/Phat.cgi?id=MP:0002628) | MP:0002628 |
|  |  | [decreased pulmonary vascular resistance](http://www.informatics.jax.org/searches/Phat.cgi?id=MP:0003035) | MP:0003035 |
|  |  | [patent ductus arteriosus](http://www.informatics.jax.org/searches/Phat.cgi?id=MP:0003139) | MP:0003139 |
|  |  | [decreased susceptibility to induced arthritis](http://www.informatics.jax.org/searches/Phat.cgi?id=MP:0003436) | MP:0003436 |
|  |  | [rheumatoid arthritis](http://www.informatics.jax.org/searches/Phat.cgi?id=MP:0003561) | MP:0003561 |
|  |  | [abnormal capillary morphology](http://www.informatics.jax.org/searches/Phat.cgi?id=MP:0003658) | MP:0003658 |
|  |  | [pulmonary edema](http://www.informatics.jax.org/searches/Phat.cgi?id=MP:0003828) | MP:0003828 |
|  |  | [lymphangiectasis](http://www.informatics.jax.org/searches/Phat.cgi?id=MP:0004038) | MP:0004038 |
|  |  | [increased lung weight](http://www.informatics.jax.org/searches/Phat.cgi?id=MP:0005630) | MP:0005630 |
|  |  | [abnormal circulating interleukin-6 level](http://www.informatics.jax.org/searches/Phat.cgi?id=MP:0008595) | MP:0008595 |
|  |  | [abnormal cytokine level](http://www.informatics.jax.org/searches/Phat.cgi?id=MP:0008713) | MP:0008713 |
|  |  | [abnormal prostaglandin level](http://www.informatics.jax.org/searches/Phat.cgi?id=MP:0009811) | MP:0009811 |
|  |  | [lung vascular congestion](http://www.informatics.jax.org/searches/Phat.cgi?id=MP:0010018) | MP:0010018 |
|  |  | [liver vascular congestion](http://www.informatics.jax.org/searches/Phat.cgi?id=MP:0010019) | MP:0010019 |
|  | MGI:2137851 | [abnormal pulmonary artery morphology](http://www.informatics.jax.org/searches/Phat.cgi?id=MP:0000484) | MP:0000484 |
|  |  | [abnormal osteoclast physiology](http://www.informatics.jax.org/searches/Phat.cgi?id=MP:0001541) | MP:0001541 |
|  |  | [abnormal T cell activation](http://www.informatics.jax.org/searches/Phat.cgi?id=MP:0001828) | MP:0001828 |
|  |  | [preneoplasia](http://www.informatics.jax.org/searches/Phat.cgi?id=MP:0002009) | MP:0002009 |
|  |  | [postnatal lethality](http://www.informatics.jax.org/searches/Phat.cgi?id=MP:0002082) | MP:0002082 |
|  |  | [abnormal respiratory alveoli morphology](http://www.informatics.jax.org/searches/Phat.cgi?id=MP:0002270) | MP:0002270 |
|  |  | [dilated heart left ventricle](http://www.informatics.jax.org/searches/Phat.cgi?id=MP:0002753) | MP:0002753 |
|  |  | [patent ductus arteriosus](http://www.informatics.jax.org/searches/Phat.cgi?id=MP:0003139) | MP:0003139 |
|  |  | [pallor](http://www.informatics.jax.org/searches/Phat.cgi?id=MP:0003717) | MP:0003717 |
|  |  | [decreased incidence of chemically-induced tumors](http://www.informatics.jax.org/searches/Phat.cgi?id=MP:0004502) | MP:0004502 |
|  |  | [decreased T cell proliferation](http://www.informatics.jax.org/searches/Phat.cgi?id=MP:0005095) | MP:0005095 |
|  |  | [lethargy](http://www.informatics.jax.org/searches/Phat.cgi?id=MP:0005202) | MP:0005202 |
|  |  | [abnormal Langerhans cell physiology](http://www.informatics.jax.org/searches/Phat.cgi?id=MP:0005362) | MP:0005362 |
|  |  | [decreased susceptibility to type IV hypersensitivity reaction](http://www.informatics.jax.org/searches/Phat.cgi?id=MP:0005616) | MP:0005616 |
|  |  | [congestive heart failure](http://www.informatics.jax.org/searches/Phat.cgi?id=MP:0006138) | MP:0006138 |
|  |  | [abnormal osteoclast differentiation](http://www.informatics.jax.org/searches/Phat.cgi?id=MP:0008396) | MP:0008396 |
|  |  | [decreased interferon-gamma secretion](http://www.informatics.jax.org/searches/Phat.cgi?id=MP:0008567) | MP:0008567 |
|  |  | [decreased survivor rate](http://www.informatics.jax.org/searches/Phat.cgi?id=MP:0008770) | MP:0008770 |
|  |  | [lung vascular congestion](http://www.informatics.jax.org/searches/Phat.cgi?id=MP:0010018) | MP:0010018 |
|  | MGI:3665234 | [perinatal lethality](http://www.informatics.jax.org/searches/Phat.cgi?id=MP:0002081) | MP:0002081 |
|  |  | [patent ductus arteriosus](http://www.informatics.jax.org/searches/Phat.cgi?id=MP:0003139) | MP:0003139 |
|  |  | [abnormal calcium ion homeostasis](http://www.informatics.jax.org/searches/Phat.cgi?id=MP:0004231) | MP:0004231 |
| [Mafb](http://www.informatics.jax.org/javawi2/servlet/WIFetch?page=markerDetail&id=MGI:104555) | MGI:3663168 | [hemorrhage](http://www.informatics.jax.org/searches/Phat.cgi?id=MP:0001914) | MP:0001914 |
|  |  | [neonatal lethality](http://www.informatics.jax.org/searches/Phat.cgi?id=MP:0002058) | MP:0002058 |
|  |  | [abnormal kidney morphology](http://www.informatics.jax.org/searches/Phat.cgi?id=MP:0002135) | MP:0002135 |
|  |  | [abnormal renal tubule morphology](http://www.informatics.jax.org/searches/Phat.cgi?id=MP:0002703) | MP:0002703 |
|  |  | [anuria](http://www.informatics.jax.org/searches/Phat.cgi?id=MP:0003624) | MP:0003624 |
|  |  | [kidney cysts](http://www.informatics.jax.org/searches/Phat.cgi?id=MP:0003675) | MP:0003675 |
|  |  | [increased renal tubule apoptosis](http://www.informatics.jax.org/searches/Phat.cgi?id=MP:0004153) | MP:0004153 |
|  |  | [decreased renal glomerulus number](http://www.informatics.jax.org/searches/Phat.cgi?id=MP:0004505) | MP:0004505 |
|  |  | [increased circulating creatinine level](http://www.informatics.jax.org/searches/Phat.cgi?id=MP:0005553) | MP:0005553 |
|  |  | [inner ear cysts](http://www.informatics.jax.org/searches/Phat.cgi?id=MP:0006287) | MP:0006287 |
|  |  | [fused podocyte foot processes](http://www.informatics.jax.org/searches/Phat.cgi?id=MP:0008139) | MP:0008139 |
| [Atp2b1](http://www.informatics.jax.org/javawi2/servlet/WIFetch?page=markerDetail&id=MGI:104653) | MGI:3051541 | [prenatal lethality](http://www.informatics.jax.org/searches/Phat.cgi?id=MP:0002080) | MP:0002080 |
|  |  | [decreased vasoconstriction](http://www.informatics.jax.org/searches/Phat.cgi?id=MP:0003026) | MP:0003026 |
| [Kcnj2](http://www.informatics.jax.org/javawi2/servlet/WIFetch?page=markerDetail&id=MGI:104744) | MGI:2183430 | [abnormal maxilla morphology](http://www.informatics.jax.org/searches/Phat.cgi?id=MP:0000455) | MP:0000455 |
|  |  | [cyanosis](http://www.informatics.jax.org/searches/Phat.cgi?id=MP:0001575) | MP:0001575 |
|  |  | [abnormal vasodilation](http://www.informatics.jax.org/searches/Phat.cgi?id=MP:0001613) | MP:0001613 |
|  |  | [respiratory distress](http://www.informatics.jax.org/searches/Phat.cgi?id=MP:0001954) | MP:0001954 |
|  |  | [neonatal lethality](http://www.informatics.jax.org/searches/Phat.cgi?id=MP:0002058) | MP:0002058 |
|  |  | [palatal shelves fail to meet at midline](http://www.informatics.jax.org/searches/Phat.cgi?id=MP:0009888) | MP:0009888 |
|  |  | [cleft secondary palate](http://www.informatics.jax.org/searches/Phat.cgi?id=MP:0009890) | MP:0009890 |
| [Ptch1](http://www.informatics.jax.org/javawi2/servlet/WIFetch?page=markerDetail&id=MGI:105373) | MGI:1857935 | [abnormal apoptosis](http://www.informatics.jax.org/searches/Phat.cgi?id=MP:0001648) | MP:0001648 |
|  |  | [rhabdomyosarcoma](http://www.informatics.jax.org/searches/Phat.cgi?id=MP:0002036) | MP:0002036 |
|  |  | [holoprosencephaly](http://www.informatics.jax.org/searches/Phat.cgi?id=MP:0005157) | MP:0005157 |
|  |  | [embryonic lethality during organogenesis](http://www.informatics.jax.org/searches/Phat.cgi?id=MP:0006207) | MP:0006207 |
|  | MGI:2655210 | [infertility](http://www.informatics.jax.org/searches/Phat.cgi?id=MP:0001924) | MP:0001924 |
|  | MGI:2675358 | [abnormal craniofacial morphology](http://www.informatics.jax.org/searches/Phat.cgi?id=MP:0000428) | MP:0000428 |
|  |  | [open neural tube](http://www.informatics.jax.org/searches/Phat.cgi?id=MP:0000929) | MP:0000929 |
|  |  | [abnormal telencephalon development](http://www.informatics.jax.org/searches/Phat.cgi?id=MP:0000934) | MP:0000934 |
|  |  | [prenatal lethality](http://www.informatics.jax.org/searches/Phat.cgi?id=MP:0002080) | MP:0002080 |
|  |  | [abnormal forebrain development](http://www.informatics.jax.org/searches/Phat.cgi?id=MP:0003232) | MP:0003232 |
|  |  | [embryonic growth retardation](http://www.informatics.jax.org/searches/Phat.cgi?id=MP:0003984) | MP:0003984 |
|  | MGI:3609560 | [abnormal mandible morphology](http://www.informatics.jax.org/searches/Phat.cgi?id=MP:0000458) | MP:0000458 |
|  | MGI:3687453 | [abnormal osteogenesis](http://www.informatics.jax.org/searches/Phat.cgi?id=MP:0000057) | MP:0000057 |
|  |  | [abnormal cartilage morphology](http://www.informatics.jax.org/searches/Phat.cgi?id=MP:0000163) | MP:0000163 |
|  |  | [abnormal long bone hypertrophic chondrocyte zone](http://www.informatics.jax.org/searches/Phat.cgi?id=MP:0000165) | MP:0000165 |
|  |  | [abnormal chondrocyte morphology](http://www.informatics.jax.org/searches/Phat.cgi?id=MP:0000166) | MP:0000166 |
|  |  | [abnormal cranium morphology](http://www.informatics.jax.org/searches/Phat.cgi?id=MP:0000438) | MP:0000438 |
|  |  | [abnormal bone mineralization](http://www.informatics.jax.org/searches/Phat.cgi?id=MP:0002896) | MP:0002896 |
|  |  | [abnormal joint morphology](http://www.informatics.jax.org/searches/Phat.cgi?id=MP:0002932) | MP:0002932 |
|  |  | [fused synovial joints](http://www.informatics.jax.org/searches/Phat.cgi?id=MP:0003190) | MP:0003190 |
|  |  | [delayed endochondral bone ossification](http://www.informatics.jax.org/searches/Phat.cgi?id=MP:0003419) | MP:0003419 |
|  |  | [decreased length of long bones](http://www.informatics.jax.org/searches/Phat.cgi?id=MP:0004686) | MP:0004686 |
|  |  | [decreased osteoblast cell number](http://www.informatics.jax.org/searches/Phat.cgi?id=MP:0004989) | MP:0004989 |
|  |  | [abnormal osteoblast physiology](http://www.informatics.jax.org/searches/Phat.cgi?id=MP:0005006) | MP:0005006 |
| [Ptpn22](http://www.informatics.jax.org/javawi2/servlet/WIFetch?page=markerDetail&id=MGI:107170) | MGI:3043469 | [enlarged spleen](http://www.informatics.jax.org/searches/Phat.cgi?id=MP:0000691) | MP:0000691 |
|  |  | [enlarged lymph nodes](http://www.informatics.jax.org/searches/Phat.cgi?id=MP:0000702) | MP:0000702 |
|  |  | [abnormal Peyer's patch germinal center morphology](http://www.informatics.jax.org/searches/Phat.cgi?id=MP:0002391) | MP:0002391 |
|  |  | [abnormal T cell physiology](http://www.informatics.jax.org/searches/Phat.cgi?id=MP:0002444) | MP:0002444 |
|  |  | [increased IgE level](http://www.informatics.jax.org/searches/Phat.cgi?id=MP:0002497) | MP:0002497 |
|  |  | [increased B cell number](http://www.informatics.jax.org/searches/Phat.cgi?id=MP:0005014) | MP:0005014 |
|  |  | [increased follicular B cell number](http://www.informatics.jax.org/searches/Phat.cgi?id=MP:0008173) | MP:0008173 |
|  |  | [increased spleen germinal center number](http://www.informatics.jax.org/searches/Phat.cgi?id=MP:0008481) | MP:0008481 |
|  |  | [increased IgG1 level](http://www.informatics.jax.org/searches/Phat.cgi?id=MP:0008499) | MP:0008499 |
|  |  | [increased IgG2a level](http://www.informatics.jax.org/searches/Phat.cgi?id=MP:0008500) | MP:0008500 |
|  | MGI:3043477 | [enlarged spleen](http://www.informatics.jax.org/searches/Phat.cgi?id=MP:0000691) | MP:0000691 |
|  |  | [enlarged lymph nodes](http://www.informatics.jax.org/searches/Phat.cgi?id=MP:0000702) | MP:0000702 |
|  |  | [abnormal lymphocyte cell number](http://www.informatics.jax.org/searches/Phat.cgi?id=MP:0000717) | MP:0000717 |
|  |  | [decreased body weight](http://www.informatics.jax.org/searches/Phat.cgi?id=MP:0001262) | MP:0001262 |
|  |  | [abnormal T cell activation](http://www.informatics.jax.org/searches/Phat.cgi?id=MP:0001828) | MP:0001828 |
|  |  | [liver inflammation](http://www.informatics.jax.org/searches/Phat.cgi?id=MP:0001860) | MP:0001860 |
|  |  | [lung inflammation](http://www.informatics.jax.org/searches/Phat.cgi?id=MP:0001861) | MP:0001861 |
|  |  | [premature death](http://www.informatics.jax.org/searches/Phat.cgi?id=MP:0002083) | MP:0002083 |
|  |  | [abnormal Peyer's patch germinal center morphology](http://www.informatics.jax.org/searches/Phat.cgi?id=MP:0002391) | MP:0002391 |
|  |  | [abnormal double-positive T cell morphology](http://www.informatics.jax.org/searches/Phat.cgi?id=MP:0002408) | MP:0002408 |
|  |  | [abnormal T cell physiology](http://www.informatics.jax.org/searches/Phat.cgi?id=MP:0002444) | MP:0002444 |
|  |  | [increased IgE level](http://www.informatics.jax.org/searches/Phat.cgi?id=MP:0002497) | MP:0002497 |
|  |  | [glomerulonephritis](http://www.informatics.jax.org/searches/Phat.cgi?id=MP:0002743) | MP:0002743 |
|  |  | [increased autoantibody level](http://www.informatics.jax.org/searches/Phat.cgi?id=MP:0003725) | MP:0003725 |
|  |  | [abnormal thymocyte activation](http://www.informatics.jax.org/searches/Phat.cgi?id=MP:0003850) | MP:0003850 |
|  |  | [abnormal T cell subpopulation ratio](http://www.informatics.jax.org/searches/Phat.cgi?id=MP:0003944) | MP:0003944 |
|  |  | [pale kidney](http://www.informatics.jax.org/searches/Phat.cgi?id=MP:0004969) | MP:0004969 |
|  |  | [increased B cell number](http://www.informatics.jax.org/searches/Phat.cgi?id=MP:0005014) | MP:0005014 |
|  |  | [proteinuria](http://www.informatics.jax.org/searches/Phat.cgi?id=MP:0005160) | MP:0005160 |
|  |  | [glomerulosclerosis](http://www.informatics.jax.org/searches/Phat.cgi?id=MP:0005264) | MP:0005264 |
|  |  | [increased memory T cell number](http://www.informatics.jax.org/searches/Phat.cgi?id=MP:0008049) | MP:0008049 |
|  |  | [increased plasma cell number](http://www.informatics.jax.org/searches/Phat.cgi?id=MP:0008097) | MP:0008097 |
|  |  | [increased follicular B cell number](http://www.informatics.jax.org/searches/Phat.cgi?id=MP:0008173) | MP:0008173 |
|  |  | [decreased follicular B cell number](http://www.informatics.jax.org/searches/Phat.cgi?id=MP:0008174) | MP:0008174 |
|  |  | [increased transitional stage B cell number](http://www.informatics.jax.org/searches/Phat.cgi?id=MP:0008189) | MP:0008189 |
|  |  | [abnormal follicular B cell physiology](http://www.informatics.jax.org/searches/Phat.cgi?id=MP:0008191) | MP:0008191 |
|  |  | [abnormal B cell activation](http://www.informatics.jax.org/searches/Phat.cgi?id=MP:0008217) | MP:0008217 |
|  |  | [increased spleen germinal center number](http://www.informatics.jax.org/searches/Phat.cgi?id=MP:0008481) | MP:0008481 |
|  |  | [increased IgG1 level](http://www.informatics.jax.org/searches/Phat.cgi?id=MP:0008499) | MP:0008499 |
|  |  | [increased IgG2a level](http://www.informatics.jax.org/searches/Phat.cgi?id=MP:0008500) | MP:0008500 |
| [Kcnj11](http://www.informatics.jax.org/javawi2/servlet/WIFetch?page=markerDetail&id=MGI:107501) | MGI:2178821 | [decreased circulating insulin level](http://www.informatics.jax.org/searches/Phat.cgi?id=MP:0002727) | MP:0002727 |
|  |  | [decreased blood pressure](http://www.informatics.jax.org/searches/Phat.cgi?id=MP:0002843) | MP:0002843 |
|  |  | [increased insulin sensitivity](http://www.informatics.jax.org/searches/Phat.cgi?id=MP:0002891) | MP:0002891 |
|  |  | [impaired glucose tolerance](http://www.informatics.jax.org/searches/Phat.cgi?id=MP:0005293) | MP:0005293 |
|  |  | [abnormal action potential](http://www.informatics.jax.org/searches/Phat.cgi?id=MP:0005402) | MP:0005402 |
|  |  | [increased circulating atrial natriuretic factor](http://www.informatics.jax.org/searches/Phat.cgi?id=MP:0005522) | MP:0005522 |
|  | MGI:3528825 |  |  |
| [Hist1h1d](http://www.informatics.jax.org/javawi2/servlet/WIFetch?page=markerDetail&id=MGI:107502) | MGI:2182776 | [no abnormal phenotype detected](http://www.informatics.jax.org/searches/Phat.cgi?id=MP:0002169) | MP:0002169 |
|  | MGI:2386170 | [no abnormal phenotype detected](http://www.informatics.jax.org/searches/Phat.cgi?id=MP:0002169) | MP:0002169 |
|  | MGI:2666743 | [enlarged pericardium](http://www.informatics.jax.org/searches/Phat.cgi?id=MP:0000291) | MP:0000291 |
|  |  | [short tail](http://www.informatics.jax.org/searches/Phat.cgi?id=MP:0000592) | MP:0000592 |
|  |  | [small thymus](http://www.informatics.jax.org/searches/Phat.cgi?id=MP:0000706) | MP:0000706 |
|  |  | [decreased body size](http://www.informatics.jax.org/searches/Phat.cgi?id=MP:0001265) | MP:0001265 |
|  |  | [decreased embryo size](http://www.informatics.jax.org/searches/Phat.cgi?id=MP:0001698) | MP:0001698 |
|  |  | [abnormal yolk sac morphology](http://www.informatics.jax.org/searches/Phat.cgi?id=MP:0001718) | MP:0001718 |
|  |  | [absent vitelline blood vessels](http://www.informatics.jax.org/searches/Phat.cgi?id=MP:0001719) | MP:0001719 |
|  |  | [pale yolk sac](http://www.informatics.jax.org/searches/Phat.cgi?id=MP:0001722) | MP:0001722 |
|  |  | [reduced fertility](http://www.informatics.jax.org/searches/Phat.cgi?id=MP:0001921) | MP:0001921 |
|  |  | [prenatal lethality](http://www.informatics.jax.org/searches/Phat.cgi?id=MP:0002080) | MP:0002080 |
|  |  | [premature death](http://www.informatics.jax.org/searches/Phat.cgi?id=MP:0002083) | MP:0002083 |
|  |  | [abnormal extraembryonic tissue morphology](http://www.informatics.jax.org/searches/Phat.cgi?id=MP:0002086) | MP:0002086 |
|  |  | [abnormal neural tube morphology/development](http://www.informatics.jax.org/searches/Phat.cgi?id=MP:0002151) | MP:0002151 |
|  |  | [abnormal rostral-caudal axis patterning](http://www.informatics.jax.org/searches/Phat.cgi?id=MP:0005221) | MP:0005221 |
|  |  | [embryonic lethality during organogenesis](http://www.informatics.jax.org/searches/Phat.cgi?id=MP:0006207) | MP:0006207 |
|  |  | [failure of chorioallantoic fusion](http://www.informatics.jax.org/searches/Phat.cgi?id=MP:0009657) | MP:0009657 |
| [Irgm1](http://www.informatics.jax.org/javawi2/servlet/WIFetch?page=markerDetail&id=MGI:107567) | MGI:3027112 | [increased susceptibility to bacterial infection](http://www.informatics.jax.org/searches/Phat.cgi?id=MP:0002412) | MP:0002412 |
|  |  | [increased susceptibility to parasitic infection](http://www.informatics.jax.org/searches/Phat.cgi?id=MP:0005027) | MP:0005027 |
|  |  | [increased circulating interferon-gamma level](http://www.informatics.jax.org/searches/Phat.cgi?id=MP:0008577) | MP:0008577 |
|  |  | [increased circulating interleukin-12 level](http://www.informatics.jax.org/searches/Phat.cgi?id=MP:0008617) | MP:0008617 |
| [Il18](http://www.informatics.jax.org/javawi2/servlet/WIFetch?page=markerDetail&id=MGI:107936) | MGI:2385814 | [decreased susceptibility to bacterial infection](http://www.informatics.jax.org/searches/Phat.cgi?id=MP:0002411) | MP:0002411 |
|  |  | [abnormal nitric oxide homeostasis](http://www.informatics.jax.org/searches/Phat.cgi?id=MP:0003957) | MP:0003957 |
|  |  | [increased macrophage cell number](http://www.informatics.jax.org/searches/Phat.cgi?id=MP:0005425) | MP:0005425 |
|  |  | [abnormal T cell morphology](http://www.informatics.jax.org/searches/Phat.cgi?id=MP:0008037) | MP:0008037 |
|  |  | [increased NK cell number](http://www.informatics.jax.org/searches/Phat.cgi?id=MP:0008044) | MP:0008044 |
|  |  | [increased dendritic cell number](http://www.informatics.jax.org/searches/Phat.cgi?id=MP:0008126) | MP:0008126 |
|  |  | [increased circulating tumor necrosis factor level](http://www.informatics.jax.org/searches/Phat.cgi?id=MP:0008553) | MP:0008553 |
|  |  | [decreased tumor necrosis factor secretion](http://www.informatics.jax.org/searches/Phat.cgi?id=MP:0008561) | MP:0008561 |
|  |  | [decreased interferon-gamma secretion](http://www.informatics.jax.org/searches/Phat.cgi?id=MP:0008567) | MP:0008567 |
|  |  | [decreased circulating interferon-gamma level](http://www.informatics.jax.org/searches/Phat.cgi?id=MP:0008578) | MP:0008578 |
|  |  | [decreased circulating interleukin-6 level](http://www.informatics.jax.org/searches/Phat.cgi?id=MP:0008597) | MP:0008597 |
|  |  | [decreased circulating interleukin-18 level](http://www.informatics.jax.org/searches/Phat.cgi?id=MP:0008636) | MP:0008636 |
|  |  | [abnormal cytokine level](http://www.informatics.jax.org/searches/Phat.cgi?id=MP:0008713) | MP:0008713 |
|  |  | [decreased susceptibility to endotoxin shock](http://www.informatics.jax.org/searches/Phat.cgi?id=MP:0008734) | MP:0008734 |
|  | MGI:2136769 | [increased white adipose tissue amount](http://www.informatics.jax.org/searches/Phat.cgi?id=MP:0000008) | MP:0000008 |
|  |  | [abnormal microglial cell morphology](http://www.informatics.jax.org/searches/Phat.cgi?id=MP:0000136) | MP:0000136 |
|  |  | [gastrointestinal hemorrhage](http://www.informatics.jax.org/searches/Phat.cgi?id=MP:0000465) | MP:0000465 |
|  |  | [polyphagia](http://www.informatics.jax.org/searches/Phat.cgi?id=MP:0001433) | MP:0001433 |
|  |  | [stomach inflammation](http://www.informatics.jax.org/searches/Phat.cgi?id=MP:0001873) | MP:0001873 |
|  |  | [increased susceptibility to bacterial infection](http://www.informatics.jax.org/searches/Phat.cgi?id=MP:0002412) | MP:0002412 |
|  |  | [abnormal macrophage physiology](http://www.informatics.jax.org/searches/Phat.cgi?id=MP:0002451) | MP:0002451 |
|  |  | [abnormal retinal vasculature morphology](http://www.informatics.jax.org/searches/Phat.cgi?id=MP:0002792) | MP:0002792 |
|  |  | [increased susceptibility to age related obesity](http://www.informatics.jax.org/searches/Phat.cgi?id=MP:0003212) | MP:0003212 |
|  |  | [abnormal heart left ventricle morphology](http://www.informatics.jax.org/searches/Phat.cgi?id=MP:0003921) | MP:0003921 |
|  |  | [decreased energy expenditure](http://www.informatics.jax.org/searches/Phat.cgi?id=MP:0004890) | MP:0004890 |
|  |  | [impaired NK cell cytolysis](http://www.informatics.jax.org/searches/Phat.cgi?id=MP:0005070) | MP:0005070 |
|  |  | [decreased oxygen consumption](http://www.informatics.jax.org/searches/Phat.cgi?id=MP:0005290) | MP:0005290 |
|  |  | [increased percent body fat](http://www.informatics.jax.org/searches/Phat.cgi?id=MP:0005458) | MP:0005458 |
|  |  | [abnormal T-helper 1 physiology](http://www.informatics.jax.org/searches/Phat.cgi?id=MP:0005465) | MP:0005465 |
|  |  | [decreased ventricle muscle contractility](http://www.informatics.jax.org/searches/Phat.cgi?id=MP:0005598) | MP:0005598 |
|  |  | [decreased cerebral infarction size](http://www.informatics.jax.org/searches/Phat.cgi?id=MP:0006058) | MP:0006058 |
|  |  | [retinal hemorrhage](http://www.informatics.jax.org/searches/Phat.cgi?id=MP:0006185) | MP:0006185 |
|  |  | [hyperchlorhydria](http://www.informatics.jax.org/searches/Phat.cgi?id=MP:0008002) | MP:0008002 |
|  |  | [decreased interferon-gamma secretion](http://www.informatics.jax.org/searches/Phat.cgi?id=MP:0008567) | MP:0008567 |
|  |  | [decreased circulating interferon-gamma level](http://www.informatics.jax.org/searches/Phat.cgi?id=MP:0008578) | MP:0008578 |
|  |  | [decreased circulating interleukin-18 level](http://www.informatics.jax.org/searches/Phat.cgi?id=MP:0008636) | MP:0008636 |
|  |  | [impaired neutrophil migration](http://www.informatics.jax.org/searches/Phat.cgi?id=MP:0008720) | MP:0008720 |
|  |  | [increased total fat pad weight](http://www.informatics.jax.org/searches/Phat.cgi?id=MP:0008908) | MP:0008908 |
| [Kcnq1](http://www.informatics.jax.org/javawi2/servlet/WIFetch?page=markerDetail&id=MGI:108083) | MGI:1931858 | [abnormal vestibule morphology](http://www.informatics.jax.org/searches/Phat.cgi?id=MP:0000034) | MP:0000034 |
|  |  | [organ of Corti degeneration](http://www.informatics.jax.org/searches/Phat.cgi?id=MP:0000043) | MP:0000043 |
|  |  | [abnormal stria vascularis](http://www.informatics.jax.org/searches/Phat.cgi?id=MP:0000048) | MP:0000048 |
|  |  | [circling](http://www.informatics.jax.org/searches/Phat.cgi?id=MP:0001394) | MP:0001394 |
|  |  | [hyperactivity](http://www.informatics.jax.org/searches/Phat.cgi?id=MP:0001399) | MP:0001399 |
|  |  | [stereotypic behavior](http://www.informatics.jax.org/searches/Phat.cgi?id=MP:0001408) | MP:0001408 |
|  |  | [head bobbing](http://www.informatics.jax.org/searches/Phat.cgi?id=MP:0001410) | MP:0001410 |
|  |  | [spinning](http://www.informatics.jax.org/searches/Phat.cgi?id=MP:0001411) | MP:0001411 |
|  |  | [deafness](http://www.informatics.jax.org/searches/Phat.cgi?id=MP:0001967) | MP:0001967 |
|  |  | [abnormal semicircular canal](http://www.informatics.jax.org/searches/Phat.cgi?id=MP:0002428) | MP:0002428 |
|  |  | [cochlear ganglion degeneration](http://www.informatics.jax.org/searches/Phat.cgi?id=MP:0002857) | MP:0002857 |
|  |  | [abnormal otolith morphology](http://www.informatics.jax.org/searches/Phat.cgi?id=MP:0002894) | MP:0002894 |
|  |  | [detached otolithic membrane](http://www.informatics.jax.org/searches/Phat.cgi?id=MP:0003145) | MP:0003145 |
|  |  | [abnormal tectorial membrane morphology](http://www.informatics.jax.org/searches/Phat.cgi?id=MP:0003149) | MP:0003149 |
|  |  | [enlarged stomach](http://www.informatics.jax.org/searches/Phat.cgi?id=MP:0003883) | MP:0003883 |
|  |  | [abnormal gastric gland](http://www.informatics.jax.org/searches/Phat.cgi?id=MP:0003892) | MP:0003892 |
|  |  | [abnormal mucous neck cell morphology](http://www.informatics.jax.org/searches/Phat.cgi?id=MP:0004138) | MP:0004138 |
|  |  | [abnormal gastric parietal cell morphology](http://www.informatics.jax.org/searches/Phat.cgi?id=MP:0004139) | MP:0004139 |
|  |  | [abnormal chief cell morphology](http://www.informatics.jax.org/searches/Phat.cgi?id=MP:0004140) | MP:0004140 |
|  |  | [abnormal crista ampullaris morphology](http://www.informatics.jax.org/searches/Phat.cgi?id=MP:0004249) | MP:0004249 |
|  |  | [absent organ of Corti supporting cells](http://www.informatics.jax.org/searches/Phat.cgi?id=MP:0004301) | MP:0004301 |
|  |  | [abnormal basilar membrane](http://www.informatics.jax.org/searches/Phat.cgi?id=MP:0004308) | MP:0004308 |
|  |  | [absent vestibular hair cells](http://www.informatics.jax.org/searches/Phat.cgi?id=MP:0004325) | MP:0004325 |
|  |  | [utricular macular degeneration](http://www.informatics.jax.org/searches/Phat.cgi?id=MP:0004334) | MP:0004334 |
|  |  | [thin stria vascularis](http://www.informatics.jax.org/searches/Phat.cgi?id=MP:0004364) | MP:0004364 |
|  |  | [absent cochlear hair cells](http://www.informatics.jax.org/searches/Phat.cgi?id=MP:0004405) | MP:0004405 |
|  |  | [abnormal vestibular labyrinth](http://www.informatics.jax.org/searches/Phat.cgi?id=MP:0004427) | MP:0004427 |
|  |  | [absent vestibular hair cell stereocilia](http://www.informatics.jax.org/searches/Phat.cgi?id=MP:0004518) | MP:0004518 |
|  |  | [increased circulating gastrin level](http://www.informatics.jax.org/searches/Phat.cgi?id=MP:0004731) | MP:0004731 |
|  |  | [absent brainstem auditory evoked potential](http://www.informatics.jax.org/searches/Phat.cgi?id=MP:0004763) | MP:0004763 |
|  |  | [abnormal gastric mucosa morphology](http://www.informatics.jax.org/searches/Phat.cgi?id=MP:0005209) | MP:0005209 |
|  |  | [increased thickness of gastric mucosa](http://www.informatics.jax.org/searches/Phat.cgi?id=MP:0005211) | MP:0005211 |
|  |  | [collapsed Reissner membrane](http://www.informatics.jax.org/searches/Phat.cgi?id=MP:0006024) | MP:0006024 |
|  |  | [hypochlorhydria](http://www.informatics.jax.org/searches/Phat.cgi?id=MP:0008001) | MP:0008001 |
|  |  | [increased stomach pH](http://www.informatics.jax.org/searches/Phat.cgi?id=MP:0008006) | MP:0008006 |
|  |  | [small scala media](http://www.informatics.jax.org/searches/Phat.cgi?id=MP:0008308) | MP:0008308 |
|  | MGI:2680653 | [abnormal inner ear morphology](http://www.informatics.jax.org/searches/Phat.cgi?id=MP:0000026) | MP:0000026 |
|  |  | [bi-directional circling](http://www.informatics.jax.org/searches/Phat.cgi?id=MP:0001395) | MP:0001395 |
|  |  | [hyperactivity](http://www.informatics.jax.org/searches/Phat.cgi?id=MP:0001399) | MP:0001399 |
|  |  | [head bobbing](http://www.informatics.jax.org/searches/Phat.cgi?id=MP:0001410) | MP:0001410 |
|  |  | [impaired righting response](http://www.informatics.jax.org/searches/Phat.cgi?id=MP:0001523) | MP:0001523 |
|  |  | [cardiac hypertrophy](http://www.informatics.jax.org/searches/Phat.cgi?id=MP:0001625) | MP:0001625 |
|  |  | [deafness](http://www.informatics.jax.org/searches/Phat.cgi?id=MP:0001967) | MP:0001967 |
|  |  | [abnormal inner ear canal morphology](http://www.informatics.jax.org/searches/Phat.cgi?id=MP:0002729) | MP:0002729 |
|  |  | [increased heart weight](http://www.informatics.jax.org/searches/Phat.cgi?id=MP:0002833) | MP:0002833 |
|  |  | [abnormal vestibular response](http://www.informatics.jax.org/searches/Phat.cgi?id=MP:0002979) | MP:0002979 |
|  |  | [abnormal impulse conducting system conduction](http://www.informatics.jax.org/searches/Phat.cgi?id=MP:0003137) | MP:0003137 |
|  |  | [prolonged QT interval](http://www.informatics.jax.org/searches/Phat.cgi?id=MP:0003233) | MP:0003233 |
|  |  | [abnormal cochlear sensory epithelium morphology](http://www.informatics.jax.org/searches/Phat.cgi?id=MP:0003308) | MP:0003308 |
|  |  | [prolonged PQ interval](http://www.informatics.jax.org/searches/Phat.cgi?id=MP:0003896) | MP:0003896 |
|  |  | [abnormal QRS complex](http://www.informatics.jax.org/searches/Phat.cgi?id=MP:0003898) | MP:0003898 |
|  |  | [cochlear inner hair cell degeneration](http://www.informatics.jax.org/searches/Phat.cgi?id=MP:0004398) | MP:0004398 |
|  |  | [cochlear outer hair cell degeneration](http://www.informatics.jax.org/searches/Phat.cgi?id=MP:0004404) | MP:0004404 |
|  |  | [abnormal crista ampullaris neuroepithelium morphology](http://www.informatics.jax.org/searches/Phat.cgi?id=MP:0004409) | MP:0004409 |
|  |  | [collapsed Reissner membrane](http://www.informatics.jax.org/searches/Phat.cgi?id=MP:0006024) | MP:0006024 |
|  |  | [abnormal saccule morphology](http://www.informatics.jax.org/searches/Phat.cgi?id=MP:0006089) | MP:0006089 |
|  |  | [abnormal utricle morphology](http://www.informatics.jax.org/searches/Phat.cgi?id=MP:0006090) | MP:0006090 |
|  |  | [syndromic hearing impairment](http://www.informatics.jax.org/searches/Phat.cgi?id=MP:0006330) | MP:0006330 |
|  |  | [absent pinna reflex](http://www.informatics.jax.org/searches/Phat.cgi?id=MP:0006358) | MP:0006358 |
| [Kif1b](http://www.informatics.jax.org/javawi2/servlet/WIFetch?page=markerDetail&id=MGI:108426) | MGI:2387573 | [lordosis](http://www.informatics.jax.org/searches/Phat.cgi?id=MP:0000162) | MP:0000162 |
|  |  | [progressive muscle weakness](http://www.informatics.jax.org/searches/Phat.cgi?id=MP:0000748) | MP:0000748 |
|  |  | [decreased brain size](http://www.informatics.jax.org/searches/Phat.cgi?id=MP:0000774) | MP:0000774 |
|  |  | [abnormal hippocampus morphology](http://www.informatics.jax.org/searches/Phat.cgi?id=MP:0000807) | MP:0000807 |
|  |  | [impaired coordination](http://www.informatics.jax.org/searches/Phat.cgi?id=MP:0001405) | MP:0001405 |
|  |  | [unresponsive to tactile stimuli](http://www.informatics.jax.org/searches/Phat.cgi?id=MP:0001491) | MP:0001491 |
|  |  | [abnormal motor coordination/ balance](http://www.informatics.jax.org/searches/Phat.cgi?id=MP:0001516) | MP:0001516 |
|  |  | [respiratory failure](http://www.informatics.jax.org/searches/Phat.cgi?id=MP:0001953) | MP:0001953 |
|  |  | [neonatal lethality](http://www.informatics.jax.org/searches/Phat.cgi?id=MP:0002058) | MP:0002058 |
|  |  | [abnormal brain commissure morphology](http://www.informatics.jax.org/searches/Phat.cgi?id=MP:0002199) | MP:0002199 |
|  |  | [abnormal axonal transport](http://www.informatics.jax.org/searches/Phat.cgi?id=MP:0004768) | MP:0004768 |
|  |  | [abnormal synaptic vesicle number](http://www.informatics.jax.org/searches/Phat.cgi?id=MP:0004792) | MP:0004792 |
|  |  | [abnormal spinal cord ventral horn morphology](http://www.informatics.jax.org/searches/Phat.cgi?id=MP:0005112) | MP:0005112 |
|  |  | [abnormal brainstem morphology](http://www.informatics.jax.org/searches/Phat.cgi?id=MP:0005277) | MP:0005277 |
|  |  | [decreased neuron number](http://www.informatics.jax.org/searches/Phat.cgi?id=MP:0008948) | MP:0008948 |
| [Hfe](http://www.informatics.jax.org/javawi2/servlet/WIFetch?page=markerDetail&id=MGI:109191) | MGI:1934013 | [short tail](http://www.informatics.jax.org/searches/Phat.cgi?id=MP:0000592) | MP:0000592 |
|  |  | [decreased body length](http://www.informatics.jax.org/searches/Phat.cgi?id=MP:0001258) | MP:0001258 |
|  |  | [impaired coordination](http://www.informatics.jax.org/searches/Phat.cgi?id=MP:0001405) | MP:0001405 |
|  |  | [abnormal gait](http://www.informatics.jax.org/searches/Phat.cgi?id=MP:0001406) | MP:0001406 |
|  |  | [abnormal pilomotor reflex](http://www.informatics.jax.org/searches/Phat.cgi?id=MP:0001492) | MP:0001492 |
|  |  | [abnormal postural reflex](http://www.informatics.jax.org/searches/Phat.cgi?id=MP:0002980) | MP:0002980 |
|  |  | [abnormal iron homeostasis](http://www.informatics.jax.org/searches/Phat.cgi?id=MP:0005637) | MP:0005637 |
|  |  | [hemochromatosis](http://www.informatics.jax.org/searches/Phat.cgi?id=MP:0005638) | MP:0005638 |
|  |  | [increased liver iron level](http://www.informatics.jax.org/searches/Phat.cgi?id=MP:0008807) | MP:0008807 |
|  |  | [increased spleen iron level](http://www.informatics.jax.org/searches/Phat.cgi?id=MP:0008809) | MP:0008809 |
|  |  | [increased circulating iron level](http://www.informatics.jax.org/searches/Phat.cgi?id=MP:0008810) | MP:0008810 |
|  |  | [induced hyperactivity](http://www.informatics.jax.org/searches/Phat.cgi?id=MP:0008911) | MP:0008911 |
|  | MGI:2181388 | [hemochromatosis](http://www.informatics.jax.org/searches/Phat.cgi?id=MP:0005638) | MP:0005638 |
|  |  | [increased liver iron level](http://www.informatics.jax.org/searches/Phat.cgi?id=MP:0008807) | MP:0008807 |
|  | MGI:2385353 | [decreased hematocrit](http://www.informatics.jax.org/searches/Phat.cgi?id=MP:0000208) | MP:0000208 |
|  |  | [postnatal growth retardation](http://www.informatics.jax.org/searches/Phat.cgi?id=MP:0001732) | MP:0001732 |
|  |  | [abnormal iron level](http://www.informatics.jax.org/searches/Phat.cgi?id=MP:0001770) | MP:0001770 |
|  |  | [postnatal lethality](http://www.informatics.jax.org/searches/Phat.cgi?id=MP:0002082) | MP:0002082 |
|  |  | [increased porphyrin level](http://www.informatics.jax.org/searches/Phat.cgi?id=MP:0004147) | MP:0004147 |
|  |  | [hypoferremia](http://www.informatics.jax.org/searches/Phat.cgi?id=MP:0004151) | MP:0004151 |
|  |  | [hypochromic microcytic anemia](http://www.informatics.jax.org/searches/Phat.cgi?id=MP:0008388) | MP:0008388 |
|  |  | [abnormal intestinal iron level](http://www.informatics.jax.org/searches/Phat.cgi?id=MP:0008740) | MP:0008740 |
|  |  | [decreased liver iron level](http://www.informatics.jax.org/searches/Phat.cgi?id=MP:0008743) | MP:0008743 |
|  |  | [increased liver iron level](http://www.informatics.jax.org/searches/Phat.cgi?id=MP:0008807) | MP:0008807 |
|  |  | [decreased spleen iron level](http://www.informatics.jax.org/searches/Phat.cgi?id=MP:0008808) | MP:0008808 |
|  |  | [increased circulating iron level](http://www.informatics.jax.org/searches/Phat.cgi?id=MP:0008810) | MP:0008810 |
|  | MGI:3775648 | [abnormal iron homeostasis](http://www.informatics.jax.org/searches/Phat.cgi?id=MP:0005637) | MP:0005637 |
|  |  | [hemochromatosis](http://www.informatics.jax.org/searches/Phat.cgi?id=MP:0005638) | MP:0005638 |
|  |  | [increased liver iron level](http://www.informatics.jax.org/searches/Phat.cgi?id=MP:0008807) | MP:0008807 |
|  |  | [decreased spleen iron level](http://www.informatics.jax.org/searches/Phat.cgi?id=MP:0008808) | MP:0008808 |
|  |  | [increased circulating iron level](http://www.informatics.jax.org/searches/Phat.cgi?id=MP:0008810) | MP:0008810 |
| [Tnfrsf11b](http://www.informatics.jax.org/javawi2/servlet/WIFetch?page=markerDetail&id=MGI:109587) | MGI:2179712 | [abnormal aorta morphology](http://www.informatics.jax.org/searches/Phat.cgi?id=MP:0000272) | MP:0000272 |
|  |  | [abnormal mandible morphology](http://www.informatics.jax.org/searches/Phat.cgi?id=MP:0000458) | MP:0000458 |
|  |  | [abnormal femur morphology](http://www.informatics.jax.org/searches/Phat.cgi?id=MP:0000559) | MP:0000559 |
|  |  | [abnormal immune system morphology](http://www.informatics.jax.org/searches/Phat.cgi?id=MP:0000685) | MP:0000685 |
|  |  | [abnormal blood vessel morphology](http://www.informatics.jax.org/searches/Phat.cgi?id=MP:0001614) | MP:0001614 |
|  |  | [abnormal immune system physiology](http://www.informatics.jax.org/searches/Phat.cgi?id=MP:0001790) | MP:0001790 |
|  |  | [abnormal dendritic cell antigen presentation](http://www.informatics.jax.org/searches/Phat.cgi?id=MP:0002455) | MP:0002455 |
|  |  | [abnormal immunoglobulin level](http://www.informatics.jax.org/searches/Phat.cgi?id=MP:0002490) | MP:0002490 |
|  |  | [abnormal long bone morphology](http://www.informatics.jax.org/searches/Phat.cgi?id=MP:0003723) | MP:0003723 |
|  |  | [abnormal bone structure](http://www.informatics.jax.org/searches/Phat.cgi?id=MP:0003795) | MP:0003795 |
|  |  | [abnormal cortical bone morphology](http://www.informatics.jax.org/searches/Phat.cgi?id=MP:0003796) | MP:0003796 |
|  |  | [increased B cell number](http://www.informatics.jax.org/searches/Phat.cgi?id=MP:0005014) | MP:0005014 |
|  |  | [atherosclerotic lesions](http://www.informatics.jax.org/searches/Phat.cgi?id=MP:0005338) | MP:0005338 |
|  |  | [increased diameter of long bones](http://www.informatics.jax.org/searches/Phat.cgi?id=MP:0008151) | MP:0008151 |
|  |  | [increased pro-B cell number](http://www.informatics.jax.org/searches/Phat.cgi?id=MP:0008186) | MP:0008186 |
|  |  | [increased immature B cell number](http://www.informatics.jax.org/searches/Phat.cgi?id=MP:0008214) | MP:0008214 |
|  |  | [abnormal pro-B cell differentiation](http://www.informatics.jax.org/searches/Phat.cgi?id=MP:0008233) | MP:0008233 |
|  |  | [abnormal blood vessel endothelium morphology](http://www.informatics.jax.org/searches/Phat.cgi?id=MP:0009489) | MP:0009489 |
|  |  | [decreased bone mineral density](http://www.informatics.jax.org/searches/Phat.cgi?id=MP:0010121) | MP:0010121 |
|  | MGI:2181227 | [abnormal inner ear morphology](http://www.informatics.jax.org/searches/Phat.cgi?id=MP:0000026) | MP:0000026 |
|  |  | [abnormal malleus morphology](http://www.informatics.jax.org/searches/Phat.cgi?id=MP:0000029) | MP:0000029 |
|  |  | [abnormal otic capsule morphology](http://www.informatics.jax.org/searches/Phat.cgi?id=MP:0000039) | MP:0000039 |
|  |  | [abnormal middle ear morphology](http://www.informatics.jax.org/searches/Phat.cgi?id=MP:0000049) | MP:0000049 |
|  |  | [fragile skeleton](http://www.informatics.jax.org/searches/Phat.cgi?id=MP:0000061) | MP:0000061 |
|  |  | [osteoporosis](http://www.informatics.jax.org/searches/Phat.cgi?id=MP:0000066) | MP:0000066 |
|  |  | [abnormal cancellous bone morphology](http://www.informatics.jax.org/searches/Phat.cgi?id=MP:0000130) | MP:0000130 |
|  |  | [decreased body weight](http://www.informatics.jax.org/searches/Phat.cgi?id=MP:0001262) | MP:0001262 |
|  |  | [abnormal skeleton physiology](http://www.informatics.jax.org/searches/Phat.cgi?id=MP:0001533) | MP:0001533 |
|  |  | [abnormal osteoclast physiology](http://www.informatics.jax.org/searches/Phat.cgi?id=MP:0001541) | MP:0001541 |
|  |  | [postnatal growth retardation](http://www.informatics.jax.org/searches/Phat.cgi?id=MP:0001732) | MP:0001732 |
|  |  | [deafness](http://www.informatics.jax.org/searches/Phat.cgi?id=MP:0001967) | MP:0001967 |
|  |  | [postnatal lethality](http://www.informatics.jax.org/searches/Phat.cgi?id=MP:0002082) | MP:0002082 |
|  |  | [abnormal skeleton development](http://www.informatics.jax.org/searches/Phat.cgi?id=MP:0002113) | MP:0002113 |
|  |  | [abnormal bone remodeling](http://www.informatics.jax.org/searches/Phat.cgi?id=MP:0002998) | MP:0002998 |
|  |  | [abnormal long bone epiphyseal plate morphology](http://www.informatics.jax.org/searches/Phat.cgi?id=MP:0003055) | MP:0003055 |
|  |  | [short femur](http://www.informatics.jax.org/searches/Phat.cgi?id=MP:0003109) | MP:0003109 |
|  |  | [abnormal bone structure](http://www.informatics.jax.org/searches/Phat.cgi?id=MP:0003795) | MP:0003795 |
|  |  | [abnormal stapes footplate](http://www.informatics.jax.org/searches/Phat.cgi?id=MP:0004290) | MP:0004290 |
|  |  | [abnormal distortion product otoacoustic emission](http://www.informatics.jax.org/searches/Phat.cgi?id=MP:0004736) | MP:0004736 |
|  |  | [absent distortion product otoacoustic emissions](http://www.informatics.jax.org/searches/Phat.cgi?id=MP:0004737) | MP:0004737 |
|  |  | [increased susceptibility to age-related hearing loss](http://www.informatics.jax.org/searches/Phat.cgi?id=MP:0004748) | MP:0004748 |
|  |  | [decreased brainstem auditory evoked potential](http://www.informatics.jax.org/searches/Phat.cgi?id=MP:0004765) | MP:0004765 |
|  |  | [otosclerosis](http://www.informatics.jax.org/searches/Phat.cgi?id=MP:0004897) | MP:0004897 |
|  |  | [increased osteoclast cell number](http://www.informatics.jax.org/searches/Phat.cgi?id=MP:0004984) | MP:0004984 |
|  |  | [decreased B cell number](http://www.informatics.jax.org/searches/Phat.cgi?id=MP:0005017) | MP:0005017 |
|  |  | [abnormal middle ear ossicle morphology](http://www.informatics.jax.org/searches/Phat.cgi?id=MP:0005105) | MP:0005105 |
|  |  | [abnormal incus morphology](http://www.informatics.jax.org/searches/Phat.cgi?id=MP:0005106) | MP:0005106 |
|  |  | [abnormal stapes morphology](http://www.informatics.jax.org/searches/Phat.cgi?id=MP:0005107) | MP:0005107 |
|  |  | [abnormal osteoclast differentiation](http://www.informatics.jax.org/searches/Phat.cgi?id=MP:0008396) | MP:0008396 |
|  |  | [decreased bone mineral density](http://www.informatics.jax.org/searches/Phat.cgi?id=MP:0010121) | MP:0010121 |
|  | MGI:2183229 | [fragile skeleton](http://www.informatics.jax.org/searches/Phat.cgi?id=MP:0000061) | MP:0000061 |
|  |  | [osteoporosis](http://www.informatics.jax.org/searches/Phat.cgi?id=MP:0000066) | MP:0000066 |
|  |  | [abnormal parietal bone morphology](http://www.informatics.jax.org/searches/Phat.cgi?id=MP:0000109) | MP:0000109 |
|  |  | [abnormal long bone epiphysis morphology](http://www.informatics.jax.org/searches/Phat.cgi?id=MP:0000131) | MP:0000131 |
|  |  | [decreased body size](http://www.informatics.jax.org/searches/Phat.cgi?id=MP:0001265) | MP:0001265 |
|  |  | [postnatal lethality](http://www.informatics.jax.org/searches/Phat.cgi?id=MP:0002082) | MP:0002082 |
|  |  | [abnormal cortical bone morphology](http://www.informatics.jax.org/searches/Phat.cgi?id=MP:0003796) | MP:0003796 |
|  |  | [abnormal osteoclast morphology](http://www.informatics.jax.org/searches/Phat.cgi?id=MP:0004982) | MP:0004982 |
|  |  | [abnormal osteoblast morphology](http://www.informatics.jax.org/searches/Phat.cgi?id=MP:0004986) | MP:0004986 |
|  |  | [arterial calcification](http://www.informatics.jax.org/searches/Phat.cgi?id=MP:0006133) | MP:0006133 |
|  |  | [decreased bone mineral density](http://www.informatics.jax.org/searches/Phat.cgi?id=MP:0010121) | MP:0010121 |
| [Gckr](http://www.informatics.jax.org/javawi2/servlet/WIFetch?page=markerDetail&id=MGI:1096345) | MGI:2384075 | [abnormal glucose homeostasis](http://www.informatics.jax.org/searches/Phat.cgi?id=MP:0002078) | MP:0002078 |
|  |  | [increased circulating insulin level](http://www.informatics.jax.org/searches/Phat.cgi?id=MP:0002079) | MP:0002079 |
|  |  | [impaired glucose tolerance](http://www.informatics.jax.org/searches/Phat.cgi?id=MP:0005293) | MP:0005293 |
|  |  | [insulin resistance](http://www.informatics.jax.org/searches/Phat.cgi?id=MP:0005331) | MP:0005331 |
|  |  | [decreased glycogen level](http://www.informatics.jax.org/searches/Phat.cgi?id=MP:0005439) | MP:0005439 |
|  |  | [increased circulating glucose level](http://www.informatics.jax.org/searches/Phat.cgi?id=MP:0005559) | MP:0005559 |
|  |  | [abnormal enzyme/coenzyme activity](http://www.informatics.jax.org/searches/Phat.cgi?id=MP:0005584) | MP:0005584 |
|  | MGI:2386670 | [decreased circulating insulin level](http://www.informatics.jax.org/searches/Phat.cgi?id=MP:0002727) | MP:0002727 |
|  |  | [impaired glucose tolerance](http://www.informatics.jax.org/searches/Phat.cgi?id=MP:0005293) | MP:0005293 |
|  |  | [abnormal enzyme/coenzyme activity](http://www.informatics.jax.org/searches/Phat.cgi?id=MP:0005584) | MP:0005584 |
| [Irf4](http://www.informatics.jax.org/javawi2/servlet/WIFetch?page=markerDetail&id=MGI:1096873) | MGI:2387941 | [abnormal spleen morphology](http://www.informatics.jax.org/searches/Phat.cgi?id=MP:0000689) | MP:0000689 |
|  |  | [enlarged spleen](http://www.informatics.jax.org/searches/Phat.cgi?id=MP:0000691) | MP:0000691 |
|  |  | [enlarged lymph nodes](http://www.informatics.jax.org/searches/Phat.cgi?id=MP:0000702) | MP:0000702 |
|  |  | [abnormal immune system cell morphology](http://www.informatics.jax.org/searches/Phat.cgi?id=MP:0000716) | MP:0000716 |
|  |  | [abnormal immune system physiology](http://www.informatics.jax.org/searches/Phat.cgi?id=MP:0001790) | MP:0001790 |
|  |  | [arrested B cell differentiation](http://www.informatics.jax.org/searches/Phat.cgi?id=MP:0001802) | MP:0001802 |
|  |  | [decreased IgM level](http://www.informatics.jax.org/searches/Phat.cgi?id=MP:0001806) | MP:0001806 |
|  |  | [abnormal B cell differentiation](http://www.informatics.jax.org/searches/Phat.cgi?id=MP:0002144) | MP:0002144 |
|  |  | [altered tumor susceptibility](http://www.informatics.jax.org/searches/Phat.cgi?id=MP:0002166) | MP:0002166 |
|  |  | [abnormal lymph node morphology](http://www.informatics.jax.org/searches/Phat.cgi?id=MP:0002339) | MP:0002339 |
|  |  | [abnormal pre-B cell morphology](http://www.informatics.jax.org/searches/Phat.cgi?id=MP:0002403) | MP:0002403 |
|  |  | [increased susceptibility to bacterial infection](http://www.informatics.jax.org/searches/Phat.cgi?id=MP:0002412) | MP:0002412 |
|  |  | [increased susceptibility to viral infection](http://www.informatics.jax.org/searches/Phat.cgi?id=MP:0002418) | MP:0002418 |
|  |  | [abnormal CD4-positive T cell morphology](http://www.informatics.jax.org/searches/Phat.cgi?id=MP:0002432) | MP:0002432 |
|  |  | [decreased immunoglobulin level](http://www.informatics.jax.org/searches/Phat.cgi?id=MP:0002460) | MP:0002460 |
|  |  | [increased pre-B cell number](http://www.informatics.jax.org/searches/Phat.cgi?id=MP:0003132) | MP:0003132 |
|  |  | [increased lymphocyte cell number](http://www.informatics.jax.org/searches/Phat.cgi?id=MP:0005013) | MP:0005013 |
|  |  | [decreased lymphocyte cell number](http://www.informatics.jax.org/searches/Phat.cgi?id=MP:0005016) | MP:0005016 |
|  |  | [decreased T cell number](http://www.informatics.jax.org/searches/Phat.cgi?id=MP:0005018) | MP:0005018 |
|  |  | [decreased B cell proliferation](http://www.informatics.jax.org/searches/Phat.cgi?id=MP:0005093) | MP:0005093 |
|  |  | [decreased T cell proliferation](http://www.informatics.jax.org/searches/Phat.cgi?id=MP:0005095) | MP:0005095 |
|  |  | [absent B cells](http://www.informatics.jax.org/searches/Phat.cgi?id=MP:0008071) | MP:0008071 |
|  |  | [abnormal T-helper 1 cell differentiation](http://www.informatics.jax.org/searches/Phat.cgi?id=MP:0008088) | MP:0008088 |
|  |  | [absent plasma cells](http://www.informatics.jax.org/searches/Phat.cgi?id=MP:0008100) | MP:0008100 |
|  |  | [decreased plasmacytoid dendritic cell number](http://www.informatics.jax.org/searches/Phat.cgi?id=MP:0008124) | MP:0008124 |
|  |  | [decreased dendritic cell number](http://www.informatics.jax.org/searches/Phat.cgi?id=MP:0008127) | MP:0008127 |
|  |  | [absent spleen germinal center](http://www.informatics.jax.org/searches/Phat.cgi?id=MP:0008474) | MP:0008474 |
|  |  | [absent lymph node germinal center](http://www.informatics.jax.org/searches/Phat.cgi?id=MP:0008523) | MP:0008523 |
|  | MGI:3712352 | [abnormal B cell differentiation](http://www.informatics.jax.org/searches/Phat.cgi?id=MP:0002144) | MP:0002144 |
|  |  | [abnormal memory B cell morphology](http://www.informatics.jax.org/searches/Phat.cgi?id=MP:0002440) | MP:0002440 |
|  |  | [abnormal B cell physiology](http://www.informatics.jax.org/searches/Phat.cgi?id=MP:0002459) | MP:0002459 |
|  |  | [abnormal class switch recombination](http://www.informatics.jax.org/searches/Phat.cgi?id=MP:0004816) | MP:0004816 |
|  |  | [decreased B cell proliferation](http://www.informatics.jax.org/searches/Phat.cgi?id=MP:0005093) | MP:0005093 |
|  |  | [absent plasma cells](http://www.informatics.jax.org/searches/Phat.cgi?id=MP:0008100) | MP:0008100 |
|  |  | [absent spleen germinal center](http://www.informatics.jax.org/searches/Phat.cgi?id=MP:0008474) | MP:0008474 |
|  |  | [decreased IgG1 level](http://www.informatics.jax.org/searches/Phat.cgi?id=MP:0008495) | MP:0008495 |
| [Tnfsf11](http://www.informatics.jax.org/javawi2/servlet/WIFetch?page=markerDetail&id=MGI:1100089) | MGI:1859962 | [osteopetrosis](http://www.informatics.jax.org/searches/Phat.cgi?id=MP:0000067) | MP:0000067 |
|  |  | [abnormal neurocranium morphology](http://www.informatics.jax.org/searches/Phat.cgi?id=MP:0000074) | MP:0000074 |
|  |  | [failure of tooth eruption](http://www.informatics.jax.org/searches/Phat.cgi?id=MP:0000121) | MP:0000121 |
|  |  | [extramedullary hematopoiesis](http://www.informatics.jax.org/searches/Phat.cgi?id=MP:0000240) | MP:0000240 |
|  |  | [abnormal immune system morphology](http://www.informatics.jax.org/searches/Phat.cgi?id=MP:0000685) | MP:0000685 |
|  |  | [postnatal growth retardation](http://www.informatics.jax.org/searches/Phat.cgi?id=MP:0001732) | MP:0001732 |
|  |  | [abnormal immune system physiology](http://www.informatics.jax.org/searches/Phat.cgi?id=MP:0001790) | MP:0001790 |
|  |  | [thymus hypoplasia](http://www.informatics.jax.org/searches/Phat.cgi?id=MP:0001823) | MP:0001823 |
|  |  | [arrested T cell differentiation](http://www.informatics.jax.org/searches/Phat.cgi?id=MP:0001825) | MP:0001825 |
|  |  | [abnormal lactation](http://www.informatics.jax.org/searches/Phat.cgi?id=MP:0001882) | MP:0001882 |
|  |  | [macrocytic anemia](http://www.informatics.jax.org/searches/Phat.cgi?id=MP:0002811) | MP:0002811 |
|  |  | [abnormal long bone epiphyseal plate morphology](http://www.informatics.jax.org/searches/Phat.cgi?id=MP:0003055) | MP:0003055 |
|  |  | [abnormal facial morphology](http://www.informatics.jax.org/searches/Phat.cgi?id=MP:0003743) | MP:0003743 |
|  |  | [decreased length of long bones](http://www.informatics.jax.org/searches/Phat.cgi?id=MP:0004686) | MP:0004686 |
|  |  | [increased spleen weight](http://www.informatics.jax.org/searches/Phat.cgi?id=MP:0004952) | MP:0004952 |
|  |  | [decreased thymus weight](http://www.informatics.jax.org/searches/Phat.cgi?id=MP:0004956) | MP:0004956 |
|  |  | [decreased osteoclast cell number](http://www.informatics.jax.org/searches/Phat.cgi?id=MP:0004985) | MP:0004985 |
|  |  | [abnormal mammary gland growth during pregnancy](http://www.informatics.jax.org/searches/Phat.cgi?id=MP:0006269) | MP:0006269 |
|  |  | [small Peyer's patches](http://www.informatics.jax.org/searches/Phat.cgi?id=MP:0008135) | MP:0008135 |
|  |  | [increased diameter of long bones](http://www.informatics.jax.org/searches/Phat.cgi?id=MP:0008151) | MP:0008151 |
|  |  | [decreased mature B cell number](http://www.informatics.jax.org/searches/Phat.cgi?id=MP:0008211) | MP:0008211 |
|  |  | [decreased immature B cell number](http://www.informatics.jax.org/searches/Phat.cgi?id=MP:0008215) | MP:0008215 |
|  |  | [abnormal pro-B cell differentiation](http://www.informatics.jax.org/searches/Phat.cgi?id=MP:0008233) | MP:0008233 |
|  |  | [absent peripheral lymph nodes](http://www.informatics.jax.org/searches/Phat.cgi?id=MP:0008464) | MP:0008464 |
|  |  | [absent mesenteric lymph nodes](http://www.informatics.jax.org/searches/Phat.cgi?id=MP:0008465) | MP:0008465 |
|  |  | [decreased interferon-gamma secretion](http://www.informatics.jax.org/searches/Phat.cgi?id=MP:0008567) | MP:0008567 |
|  |  | [decreased interleukin-2 secretion](http://www.informatics.jax.org/searches/Phat.cgi?id=MP:0008688) | MP:0008688 |
|  |  | [decreased interleukin-4 secretion](http://www.informatics.jax.org/searches/Phat.cgi?id=MP:0008700) | MP:0008700 |
|  |  | [decreased interleukin-5 secretion](http://www.informatics.jax.org/searches/Phat.cgi?id=MP:0008703) | MP:0008703 |
|  |  | [decreased interleukin-6 secretion](http://www.informatics.jax.org/searches/Phat.cgi?id=MP:0008706) | MP:0008706 |
|  |  | [increased bone mineral density](http://www.informatics.jax.org/searches/Phat.cgi?id=MP:0010120) | MP:0010120 |
|  | MGI:2386263 | [failure of tooth eruption](http://www.informatics.jax.org/searches/Phat.cgi?id=MP:0000121) | MP:0000121 |
|  |  | [abnormal chondrocyte morphology](http://www.informatics.jax.org/searches/Phat.cgi?id=MP:0000166) | MP:0000166 |
|  |  | [longitudinally short skull](http://www.informatics.jax.org/searches/Phat.cgi?id=MP:0000442) | MP:0000442 |
|  |  | [abnormal spleen morphology](http://www.informatics.jax.org/searches/Phat.cgi?id=MP:0000689) | MP:0000689 |
|  |  | [abnormal cervical lymph node morphology](http://www.informatics.jax.org/searches/Phat.cgi?id=MP:0002351) | MP:0002351 |
|  |  | [abnormal spleen marginal zone morphology](http://www.informatics.jax.org/searches/Phat.cgi?id=MP:0002362) | MP:0002362 |
|  |  | [short tibia](http://www.informatics.jax.org/searches/Phat.cgi?id=MP:0002764) | MP:0002764 |
|  |  | [abnormal long bone epiphyseal plate morphology](http://www.informatics.jax.org/searches/Phat.cgi?id=MP:0003055) | MP:0003055 |
|  |  | [increased width of hypertrophic chondrocyte zone](http://www.informatics.jax.org/searches/Phat.cgi?id=MP:0003408) | MP:0003408 |
|  |  | [abnormal long bone epiphyseal plate proliferative zone](http://www.informatics.jax.org/searches/Phat.cgi?id=MP:0003662) | MP:0003662 |
|  |  | [abnormal pelvic girdle bone morphology](http://www.informatics.jax.org/searches/Phat.cgi?id=MP:0004509) | MP:0004509 |
|  |  | [decreased length of long bones](http://www.informatics.jax.org/searches/Phat.cgi?id=MP:0004686) | MP:0004686 |
|  |  | [short vertebral column](http://www.informatics.jax.org/searches/Phat.cgi?id=MP:0004704) | MP:0004704 |
|  |  | [decreased osteoclast cell number](http://www.informatics.jax.org/searches/Phat.cgi?id=MP:0004985) | MP:0004985 |
|  |  | [increased long bone epiphyseal plate size](http://www.informatics.jax.org/searches/Phat.cgi?id=MP:0006398) | MP:0006398 |
|  |  | [decreased Langerhans cell number](http://www.informatics.jax.org/searches/Phat.cgi?id=MP:0008119) | MP:0008119 |
|  |  | [increased diameter of tibia](http://www.informatics.jax.org/searches/Phat.cgi?id=MP:0008162) | MP:0008162 |
|  |  | [absent peripheral lymph nodes](http://www.informatics.jax.org/searches/Phat.cgi?id=MP:0008464) | MP:0008464 |
|  |  | [absent mesenteric lymph nodes](http://www.informatics.jax.org/searches/Phat.cgi?id=MP:0008465) | MP:0008465 |
|  |  | [abnormal spleen B cell follicle morphology](http://www.informatics.jax.org/searches/Phat.cgi?id=MP:0008470) | MP:0008470 |
|  |  | [absent cervical lymph nodes](http://www.informatics.jax.org/searches/Phat.cgi?id=MP:0009633) | MP:0009633 |
| [Tnfaip3](http://www.informatics.jax.org/javawi2/servlet/WIFetch?page=markerDetail&id=MGI:1196377) | MGI:3055173 | [increased granulocyte number](http://www.informatics.jax.org/searches/Phat.cgi?id=MP:0000322) | MP:0000322 |
|  |  | [thickened epidermis](http://www.informatics.jax.org/searches/Phat.cgi?id=MP:0001219) | MP:0001219 |
|  |  | [thick dermal layer](http://www.informatics.jax.org/searches/Phat.cgi?id=MP:0001245) | MP:0001245 |
|  |  | [decreased body size](http://www.informatics.jax.org/searches/Phat.cgi?id=MP:0001265) | MP:0001265 |
|  |  | [abnormal inflammatory response](http://www.informatics.jax.org/searches/Phat.cgi?id=MP:0001845) | MP:0001845 |
|  |  | [increased inflammatory response](http://www.informatics.jax.org/searches/Phat.cgi?id=MP:0001846) | MP:0001846 |
|  |  | [intestinal inflammation](http://www.informatics.jax.org/searches/Phat.cgi?id=MP:0001858) | MP:0001858 |
|  |  | [kidney inflammation](http://www.informatics.jax.org/searches/Phat.cgi?id=MP:0001859) | MP:0001859 |
|  |  | [liver inflammation](http://www.informatics.jax.org/searches/Phat.cgi?id=MP:0001860) | MP:0001860 |
|  |  | [postnatal lethality](http://www.informatics.jax.org/searches/Phat.cgi?id=MP:0002082) | MP:0002082 |
|  |  | [premature death](http://www.informatics.jax.org/searches/Phat.cgi?id=MP:0002083) | MP:0002083 |
|  |  | [joint inflammation](http://www.informatics.jax.org/searches/Phat.cgi?id=MP:0002933) | MP:0002933 |
|  |  | [abnormal cytokine secretion](http://www.informatics.jax.org/searches/Phat.cgi?id=MP:0003009) | MP:0003009 |
|  |  | [increased lymphocyte cell number](http://www.informatics.jax.org/searches/Phat.cgi?id=MP:0005013) | MP:0005013 |
|  |  | [cachexia](http://www.informatics.jax.org/searches/Phat.cgi?id=MP:0005150) | MP:0005150 |
|  |  | [increased macrophage cell number](http://www.informatics.jax.org/searches/Phat.cgi?id=MP:0005425) | MP:0005425 |
|  |  | [increased sensitivity to induced morbidity/mortality](http://www.informatics.jax.org/searches/Phat.cgi?id=MP:0009763) | MP:0009763 |
| [Sh2b1](http://www.informatics.jax.org/javawi2/servlet/WIFetch?page=markerDetail&id=MGI:1201407) | MGI:2662436 | [abnormal oocyte morphology](http://www.informatics.jax.org/searches/Phat.cgi?id=MP:0001125) | MP:0001125 |
|  |  | [abnormal ovarian folliculogenesis](http://www.informatics.jax.org/searches/Phat.cgi?id=MP:0001130) | MP:0001130 |
|  |  | [decreased body weight](http://www.informatics.jax.org/searches/Phat.cgi?id=MP:0001262) | MP:0001262 |
|  |  | [postnatal growth retardation](http://www.informatics.jax.org/searches/Phat.cgi?id=MP:0001732) | MP:0001732 |
|  |  | [reduced male fertility](http://www.informatics.jax.org/searches/Phat.cgi?id=MP:0001922) | MP:0001922 |
|  |  | [reduced female fertility](http://www.informatics.jax.org/searches/Phat.cgi?id=MP:0001923) | MP:0001923 |
|  |  | [abnormal ovulation](http://www.informatics.jax.org/searches/Phat.cgi?id=MP:0001928) | MP:0001928 |
|  |  | [testicular hypoplasia](http://www.informatics.jax.org/searches/Phat.cgi?id=MP:0001940) | MP:0001940 |
|  |  | [neonatal lethality](http://www.informatics.jax.org/searches/Phat.cgi?id=MP:0002058) | MP:0002058 |
|  |  | [abnormal epididymis morphology](http://www.informatics.jax.org/searches/Phat.cgi?id=MP:0002631) | MP:0002631 |
|  |  | [asthenozoospermia](http://www.informatics.jax.org/searches/Phat.cgi?id=MP:0002675) | MP:0002675 |
|  |  | [oligozoospermia](http://www.informatics.jax.org/searches/Phat.cgi?id=MP:0002687) | MP:0002687 |
|  |  | [abnormal uterus development](http://www.informatics.jax.org/searches/Phat.cgi?id=MP:0003572) | MP:0003572 |
|  |  | [decreased testis weight](http://www.informatics.jax.org/searches/Phat.cgi?id=MP:0004852) | MP:0004852 |
|  |  | [decreased ovary weight](http://www.informatics.jax.org/searches/Phat.cgi?id=MP:0004856) | MP:0004856 |
|  |  | [ovary hypoplasia](http://www.informatics.jax.org/searches/Phat.cgi?id=MP:0005158) | MP:0005158 |
|  |  | [Leydig cell hypoplasia](http://www.informatics.jax.org/searches/Phat.cgi?id=MP:0005536) | MP:0005536 |
|  |  | [proportional dwarf](http://www.informatics.jax.org/searches/Phat.cgi?id=MP:0008974) | MP:0008974 |
|  | MGI:3055487 | [decreased body size](http://www.informatics.jax.org/searches/Phat.cgi?id=MP:0001265) | MP:0001265 |
|  |  | [hyperglycemia](http://www.informatics.jax.org/searches/Phat.cgi?id=MP:0001559) | MP:0001559 |
|  |  | [reduced fertility](http://www.informatics.jax.org/searches/Phat.cgi?id=MP:0001921) | MP:0001921 |
|  |  | [impaired glucose tolerance](http://www.informatics.jax.org/searches/Phat.cgi?id=MP:0005293) | MP:0005293 |
|  |  | [insulin resistance](http://www.informatics.jax.org/searches/Phat.cgi?id=MP:0005331) | MP:0005331 |
|  |  | [pancreatic islet hyperplasia](http://www.informatics.jax.org/searches/Phat.cgi?id=MP:0005491) | MP:0005491 |
| [Gck](http://www.informatics.jax.org/javawi2/servlet/WIFetch?page=markerDetail&id=MGI:1270854) | MGI:1861929 | [hyperglycemia](http://www.informatics.jax.org/searches/Phat.cgi?id=MP:0001559) | MP:0001559 |
|  |  | [abnormal glucose homeostasis](http://www.informatics.jax.org/searches/Phat.cgi?id=MP:0002078) | MP:0002078 |
|  |  | [impaired glucose tolerance](http://www.informatics.jax.org/searches/Phat.cgi?id=MP:0005293) | MP:0005293 |
|  |  | [embryonic lethality during organogenesis](http://www.informatics.jax.org/searches/Phat.cgi?id=MP:0006207) | MP:0006207 |
|  | MGI:1857469 | [abnormal circulating cholesterol level](http://www.informatics.jax.org/searches/Phat.cgi?id=MP:0000180) | MP:0000180 |
|  |  | [increased circulating triglyceride level](http://www.informatics.jax.org/searches/Phat.cgi?id=MP:0001552) | MP:0001552 |
|  |  | [hyperglycemia](http://www.informatics.jax.org/searches/Phat.cgi?id=MP:0001559) | MP:0001559 |
|  |  | [postnatal lethality](http://www.informatics.jax.org/searches/Phat.cgi?id=MP:0002082) | MP:0002082 |
|  |  | [hepatic steatosis](http://www.informatics.jax.org/searches/Phat.cgi?id=MP:0002628) | MP:0002628 |
|  |  | [impaired glucose tolerance](http://www.informatics.jax.org/searches/Phat.cgi?id=MP:0005293) | MP:0005293 |
|  |  | [abnormal glycogen homeostasis](http://www.informatics.jax.org/searches/Phat.cgi?id=MP:0005438) | MP:0005438 |
|  | MGI:2177703 | [decreased body weight](http://www.informatics.jax.org/searches/Phat.cgi?id=MP:0001262) | MP:0001262 |
|  |  | [hyperglycemia](http://www.informatics.jax.org/searches/Phat.cgi?id=MP:0001559) | MP:0001559 |
|  |  | [postnatal growth retardation](http://www.informatics.jax.org/searches/Phat.cgi?id=MP:0001732) | MP:0001732 |
|  |  | [increased urine glucose level](http://www.informatics.jax.org/searches/Phat.cgi?id=MP:0001759) | MP:0001759 |
|  |  | [increased circulating insulin level](http://www.informatics.jax.org/searches/Phat.cgi?id=MP:0002079) | MP:0002079 |
|  |  | [postnatal lethality](http://www.informatics.jax.org/searches/Phat.cgi?id=MP:0002082) | MP:0002082 |
|  |  | [increased circulating ketone body level](http://www.informatics.jax.org/searches/Phat.cgi?id=MP:0002575) | MP:0002575 |
|  |  | [hepatic steatosis](http://www.informatics.jax.org/searches/Phat.cgi?id=MP:0002628) | MP:0002628 |
|  |  | [decreased circulating insulin level](http://www.informatics.jax.org/searches/Phat.cgi?id=MP:0002727) | MP:0002727 |
|  |  | [decreased insulin secretion](http://www.informatics.jax.org/searches/Phat.cgi?id=MP:0003059) | MP:0003059 |
|  |  | [abnormal pancreatic beta cell physiology](http://www.informatics.jax.org/searches/Phat.cgi?id=MP:0003562) | MP:0003562 |
|  |  | [impaired glucose tolerance](http://www.informatics.jax.org/searches/Phat.cgi?id=MP:0005293) | MP:0005293 |
|  |  | [insulin resistance](http://www.informatics.jax.org/searches/Phat.cgi?id=MP:0005331) | MP:0005331 |
|  |  | [pancreatic islet hyperplasia](http://www.informatics.jax.org/searches/Phat.cgi?id=MP:0005491) | MP:0005491 |
|  |  | [increased pancreatic beta cell mass](http://www.informatics.jax.org/searches/Phat.cgi?id=MP:0009113) | MP:0009113 |
|  | MGI:2177710 | [increased circulating free fatty acid level](http://www.informatics.jax.org/searches/Phat.cgi?id=MP:0001554) | MP:0001554 |
|  |  | [hyperglycemia](http://www.informatics.jax.org/searches/Phat.cgi?id=MP:0001559) | MP:0001559 |
|  |  | [postnatal lethality](http://www.informatics.jax.org/searches/Phat.cgi?id=MP:0002082) | MP:0002082 |
|  |  | [hepatic steatosis](http://www.informatics.jax.org/searches/Phat.cgi?id=MP:0002628) | MP:0002628 |
|  |  | [decreased circulating insulin level](http://www.informatics.jax.org/searches/Phat.cgi?id=MP:0002727) | MP:0002727 |
|  |  | [decreased insulin secretion](http://www.informatics.jax.org/searches/Phat.cgi?id=MP:0003059) | MP:0003059 |
|  |  | [impaired glucose tolerance](http://www.informatics.jax.org/searches/Phat.cgi?id=MP:0005293) | MP:0005293 |
|  |  | [decreased glycogen level](http://www.informatics.jax.org/searches/Phat.cgi?id=MP:0005439) | MP:0005439 |
| [Cdk6](http://www.informatics.jax.org/javawi2/servlet/WIFetch?page=markerDetail&id=MGI:1277162) | MGI:3053975 | [decreased leukocyte cell number](http://www.informatics.jax.org/searches/Phat.cgi?id=MP:0000221) | MP:0000221 |
|  |  | [extramedullary hematopoiesis](http://www.informatics.jax.org/searches/Phat.cgi?id=MP:0000240) | MP:0000240 |
|  |  | [thin ventricular wall](http://www.informatics.jax.org/searches/Phat.cgi?id=MP:0000280) | MP:0000280 |
|  |  | [decreased cell proliferation](http://www.informatics.jax.org/searches/Phat.cgi?id=MP:0000352) | MP:0000352 |
|  |  | [liver hypoplasia](http://www.informatics.jax.org/searches/Phat.cgi?id=MP:0000600) | MP:0000600 |
|  |  | [abnormal spleen morphology](http://www.informatics.jax.org/searches/Phat.cgi?id=MP:0000689) | MP:0000689 |
|  |  | [small spleen](http://www.informatics.jax.org/searches/Phat.cgi?id=MP:0000692) | MP:0000692 |
|  |  | [spleen hypoplasia](http://www.informatics.jax.org/searches/Phat.cgi?id=MP:0000694) | MP:0000694 |
|  |  | [abnormal thymus morphology](http://www.informatics.jax.org/searches/Phat.cgi?id=MP:0000703) | MP:0000703 |
|  |  | [abnormal spermatogenesis](http://www.informatics.jax.org/searches/Phat.cgi?id=MP:0001156) | MP:0001156 |
|  |  | [decreased body weight](http://www.informatics.jax.org/searches/Phat.cgi?id=MP:0001262) | MP:0001262 |
|  |  | [decreased embryo size](http://www.informatics.jax.org/searches/Phat.cgi?id=MP:0001698) | MP:0001698 |
|  |  | [thymus hypoplasia](http://www.informatics.jax.org/searches/Phat.cgi?id=MP:0001823) | MP:0001823 |
|  |  | [reduced female fertility](http://www.informatics.jax.org/searches/Phat.cgi?id=MP:0001923) | MP:0001923 |
|  |  | [infertility](http://www.informatics.jax.org/searches/Phat.cgi?id=MP:0001924) | MP:0001924 |
|  |  | [abnormal gametogenesis](http://www.informatics.jax.org/searches/Phat.cgi?id=MP:0001929) | MP:0001929 |
|  |  | [abnormal oogenesis](http://www.informatics.jax.org/searches/Phat.cgi?id=MP:0001931) | MP:0001931 |
|  |  | [neonatal lethality](http://www.informatics.jax.org/searches/Phat.cgi?id=MP:0002058) | MP:0002058 |
|  |  | [abnormal hematopoiesis](http://www.informatics.jax.org/searches/Phat.cgi?id=MP:0002123) | MP:0002123 |
|  |  | [abnormal red blood cell](http://www.informatics.jax.org/searches/Phat.cgi?id=MP:0002124) | MP:0002124 |
|  |  | [abnormal spleen red pulp morphology](http://www.informatics.jax.org/searches/Phat.cgi?id=MP:0002356) | MP:0002356 |
|  |  | [abnormal hematopoietic system morphology/development](http://www.informatics.jax.org/searches/Phat.cgi?id=MP:0002396) | MP:0002396 |
|  |  | [abnormal erythrocyte morphology](http://www.informatics.jax.org/searches/Phat.cgi?id=MP:0002447) | MP:0002447 |
|  |  | [increased mean corpuscular volume](http://www.informatics.jax.org/searches/Phat.cgi?id=MP:0002590) | MP:0002590 |
|  |  | [decreased mature ovarian follicle number](http://www.informatics.jax.org/searches/Phat.cgi?id=MP:0002682) | MP:0002682 |
|  |  | [decreased erythrocyte cell number](http://www.informatics.jax.org/searches/Phat.cgi?id=MP:0002875) | MP:0002875 |
|  |  | [thymus atrophy](http://www.informatics.jax.org/searches/Phat.cgi?id=MP:0003644) | MP:0003644 |
|  |  | [decreased myocardial fiber number](http://www.informatics.jax.org/searches/Phat.cgi?id=MP:0004567) | MP:0004567 |
|  |  | [decreased hematopoietic stem cell number](http://www.informatics.jax.org/searches/Phat.cgi?id=MP:0004810) | MP:0004810 |
|  |  | [decreased T cell proliferation](http://www.informatics.jax.org/searches/Phat.cgi?id=MP:0005095) | MP:0005095 |
|  |  | [increased mean corpuscular hemoglobin concentration](http://www.informatics.jax.org/searches/Phat.cgi?id=MP:0005641) | MP:0005641 |
|  |  | [embryonic lethality during organogenesis](http://www.informatics.jax.org/searches/Phat.cgi?id=MP:0006207) | MP:0006207 |
|  |  | [lethality throughout fetal growth and development](http://www.informatics.jax.org/searches/Phat.cgi?id=MP:0006208) | MP:0006208 |
|  |  | [abnormal common myeloid progenitor cell morphology](http://www.informatics.jax.org/searches/Phat.cgi?id=MP:0006410) | MP:0006410 |
|  |  | [increased single-positive T cell number](http://www.informatics.jax.org/searches/Phat.cgi?id=MP:0008082) | MP:0008082 |
|  |  | [decreased megakaryocyte cell number](http://www.informatics.jax.org/searches/Phat.cgi?id=MP:0008255) | MP:0008255 |
| [G6pc2](http://www.informatics.jax.org/javawi2/servlet/WIFetch?page=markerDetail&id=MGI:1277193) | MGI:3819785 | [decreased circulating triglyceride level](http://www.informatics.jax.org/searches/Phat.cgi?id=MP:0002644) | MP:0002644 |
|  |  | [decreased circulating glucose level](http://www.informatics.jax.org/searches/Phat.cgi?id=MP:0005560) | MP:0005560 |
| [Lrp5](http://www.informatics.jax.org/javawi2/servlet/WIFetch?page=markerDetail&id=MGI:1278315) | MGI:2448955 | [abnormal parietal bone morphology](http://www.informatics.jax.org/searches/Phat.cgi?id=MP:0000109) | MP:0000109 |
|  |  | [abnormal femur morphology](http://www.informatics.jax.org/searches/Phat.cgi?id=MP:0000559) | MP:0000559 |
|  |  | [abnormal liver physiology](http://www.informatics.jax.org/searches/Phat.cgi?id=MP:0000609) | MP:0000609 |
|  |  | [abnormal limb morphology](http://www.informatics.jax.org/searches/Phat.cgi?id=MP:0002109) | MP:0002109 |
|  |  | [decreased circulating insulin level](http://www.informatics.jax.org/searches/Phat.cgi?id=MP:0002727) | MP:0002727 |
|  |  | [decreased insulin secretion](http://www.informatics.jax.org/searches/Phat.cgi?id=MP:0003059) | MP:0003059 |
|  |  | [increased cholesterol level](http://www.informatics.jax.org/searches/Phat.cgi?id=MP:0003982) | MP:0003982 |
|  |  | [abnormal calcium ion homeostasis](http://www.informatics.jax.org/searches/Phat.cgi?id=MP:0004231) | MP:0004231 |
|  |  | [increased circulating VLDL cholesterol level](http://www.informatics.jax.org/searches/Phat.cgi?id=MP:0005145) | MP:0005145 |
|  |  | [impaired glucose tolerance](http://www.informatics.jax.org/searches/Phat.cgi?id=MP:0005293) | MP:0005293 |
|  |  | [insulin resistance](http://www.informatics.jax.org/searches/Phat.cgi?id=MP:0005331) | MP:0005331 |
|  |  | [decreased diameter of femur](http://www.informatics.jax.org/searches/Phat.cgi?id=MP:0008152) | MP:0008152 |
|  |  | [decreased diameter of tibia](http://www.informatics.jax.org/searches/Phat.cgi?id=MP:0008156) | MP:0008156 |
|  | MGI:3513196 |  |  |
|  | MGI:3604604 | [abnormal retina morphology](http://www.informatics.jax.org/searches/Phat.cgi?id=MP:0001325) | MP:0001325 |
|  |  | [retinal degeneration](http://www.informatics.jax.org/searches/Phat.cgi?id=MP:0001326) | MP:0001326 |
|  |  | [hypoactivity](http://www.informatics.jax.org/searches/Phat.cgi?id=MP:0001402) | MP:0001402 |
|  |  | [gliosis](http://www.informatics.jax.org/searches/Phat.cgi?id=MP:0002183) | MP:0002183 |
|  |  | [increased thigmotaxis](http://www.informatics.jax.org/searches/Phat.cgi?id=MP:0002797) | MP:0002797 |
|  |  | [abnormal retinal layer morphology](http://www.informatics.jax.org/searches/Phat.cgi?id=MP:0003727) | MP:0003727 |
|  |  | [abnormal retinal outer nuclear layer morphology](http://www.informatics.jax.org/searches/Phat.cgi?id=MP:0003731) | MP:0003731 |
|  |  | [abnormal retinal outer plexiform layer morphology](http://www.informatics.jax.org/searches/Phat.cgi?id=MP:0003732) | MP:0003732 |
|  |  | [abnormal retinal pigment epithelium morphology](http://www.informatics.jax.org/searches/Phat.cgi?id=MP:0005201) | MP:0005201 |
|  |  | [abnormal retinal nerve fiber layer morphology](http://www.informatics.jax.org/searches/Phat.cgi?id=MP:0006303) | MP:0006303 |
|  |  | [retinal ganglion cell degeneration](http://www.informatics.jax.org/searches/Phat.cgi?id=MP:0008067) | MP:0008067 |
|  |  | [retinal photoreceptor degeneration](http://www.informatics.jax.org/searches/Phat.cgi?id=MP:0008450) | MP:0008450 |
|  |  | [disorganized retinal inner nuclear layer](http://www.informatics.jax.org/searches/Phat.cgi?id=MP:0008512) | MP:0008512 |
|  |  | [thin retinal inner plexiform layer](http://www.informatics.jax.org/searches/Phat.cgi?id=MP:0008513) | MP:0008513 |
|  |  | [thin retinal outer nuclear layer](http://www.informatics.jax.org/searches/Phat.cgi?id=MP:0008515) | MP:0008515 |
|  |  | [disorganized retinal outer nuclear layer](http://www.informatics.jax.org/searches/Phat.cgi?id=MP:0008516) | MP:0008516 |
|  |  | [thin retinal outer plexiform layer](http://www.informatics.jax.org/searches/Phat.cgi?id=MP:0008519) | MP:0008519 |
|  |  | [disorganized retinal outer plexiform layer](http://www.informatics.jax.org/searches/Phat.cgi?id=MP:0008520) | MP:0008520 |
|  | MGI:3612485 | [abnormal osteogenesis](http://www.informatics.jax.org/searches/Phat.cgi?id=MP:0000057) | MP:0000057 |
|  |  | [decreased bone density](http://www.informatics.jax.org/searches/Phat.cgi?id=MP:0000063) | MP:0000063 |
|  |  | [abnormal cancellous bone morphology](http://www.informatics.jax.org/searches/Phat.cgi?id=MP:0000130) | MP:0000130 |
|  |  | [abnormal bone mineralization](http://www.informatics.jax.org/searches/Phat.cgi?id=MP:0002896) | MP:0002896 |
|  |  | [abnormal bone structure](http://www.informatics.jax.org/searches/Phat.cgi?id=MP:0003795) | MP:0003795 |
|  |  | [decreased bone mass](http://www.informatics.jax.org/searches/Phat.cgi?id=MP:0004016) | MP:0004016 |
|  |  | [decreased osteoclast cell number](http://www.informatics.jax.org/searches/Phat.cgi?id=MP:0004985) | MP:0004985 |
|  |  | [decreased osteoblast cell number](http://www.informatics.jax.org/searches/Phat.cgi?id=MP:0004989) | MP:0004989 |
|  |  | [abnormal osteoblast physiology](http://www.informatics.jax.org/searches/Phat.cgi?id=MP:0005006) | MP:0005006 |
|  | MGI:3663738 | [decreased bone density](http://www.informatics.jax.org/searches/Phat.cgi?id=MP:0000063) | MP:0000063 |
|  |  | [abnormal cancellous bone morphology](http://www.informatics.jax.org/searches/Phat.cgi?id=MP:0000130) | MP:0000130 |
|  |  | [abnormal cranium morphology](http://www.informatics.jax.org/searches/Phat.cgi?id=MP:0000438) | MP:0000438 |
|  |  | [abnormal forelimb morphology](http://www.informatics.jax.org/searches/Phat.cgi?id=MP:0000550) | MP:0000550 |
|  |  | [abnormal carpal bone morphology](http://www.informatics.jax.org/searches/Phat.cgi?id=MP:0000554) | MP:0000554 |
|  |  | [abnormal hindlimb morphology](http://www.informatics.jax.org/searches/Phat.cgi?id=MP:0000556) | MP:0000556 |
|  |  | [oligodactyly](http://www.informatics.jax.org/searches/Phat.cgi?id=MP:0000565) | MP:0000565 |
|  |  | [synostosis](http://www.informatics.jax.org/searches/Phat.cgi?id=MP:0000566) | MP:0000566 |
|  |  | [persistence of hyaloid capillary system](http://www.informatics.jax.org/searches/Phat.cgi?id=MP:0001289) | MP:0001289 |
|  |  | [abnormal limb morphology](http://www.informatics.jax.org/searches/Phat.cgi?id=MP:0002109) | MP:0002109 |
|  |  | [monodactyly](http://www.informatics.jax.org/searches/Phat.cgi?id=MP:0003800) | MP:0003800 |
|  |  | [decreased bone mass](http://www.informatics.jax.org/searches/Phat.cgi?id=MP:0004016) | MP:0004016 |
|  |  | [abnormal tarsal bone morphology](http://www.informatics.jax.org/searches/Phat.cgi?id=MP:0005104) | MP:0005104 |
|  |  | [decreased bone mineral density](http://www.informatics.jax.org/searches/Phat.cgi?id=MP:0010121) | MP:0010121 |
| [Ccr6](http://www.informatics.jax.org/javawi2/servlet/WIFetch?page=markerDetail&id=MGI:1333797) | MGI:2179552 | [abnormal Peyer's patch morphology](http://www.informatics.jax.org/searches/Phat.cgi?id=MP:0000696) | MP:0000696 |
|  |  | [abnormal choroid plexus morphology](http://www.informatics.jax.org/searches/Phat.cgi?id=MP:0000820) | MP:0000820 |
|  |  | [decreased IgA level](http://www.informatics.jax.org/searches/Phat.cgi?id=MP:0001807) | MP:0001807 |
|  |  | [increased activated T cell number](http://www.informatics.jax.org/searches/Phat.cgi?id=MP:0001829) | MP:0001829 |
|  |  | [decreased inflammatory response](http://www.informatics.jax.org/searches/Phat.cgi?id=MP:0001876) | MP:0001876 |
|  |  | [abnormal gut-associated lymphoid tissue morphology](http://www.informatics.jax.org/searches/Phat.cgi?id=MP:0002378) | MP:0002378 |
|  |  | [abnormal Peyer's patch germinal center morphology](http://www.informatics.jax.org/searches/Phat.cgi?id=MP:0002391) | MP:0002391 |
|  |  | [abnormal Peyer's patch T cell area](http://www.informatics.jax.org/searches/Phat.cgi?id=MP:0002392) | MP:0002392 |
|  |  | [increased susceptibility to viral infection](http://www.informatics.jax.org/searches/Phat.cgi?id=MP:0002418) | MP:0002418 |
|  |  | [abnormal CD4-positive T cell morphology](http://www.informatics.jax.org/searches/Phat.cgi?id=MP:0002432) | MP:0002432 |
|  |  | [abnormal T cell physiology](http://www.informatics.jax.org/searches/Phat.cgi?id=MP:0002444) | MP:0002444 |
|  |  | [abnormal macrophage physiology](http://www.informatics.jax.org/searches/Phat.cgi?id=MP:0002451) | MP:0002451 |
|  |  | [decreased IgE level](http://www.informatics.jax.org/searches/Phat.cgi?id=MP:0002492) | MP:0002492 |
|  |  | [increased IgM level](http://www.informatics.jax.org/searches/Phat.cgi?id=MP:0002494) | MP:0002494 |
|  |  | [abnormal lymphocyte morphology](http://www.informatics.jax.org/searches/Phat.cgi?id=MP:0002619) | MP:0002619 |
|  |  | [abnormal nitric oxide homeostasis](http://www.informatics.jax.org/searches/Phat.cgi?id=MP:0003957) | MP:0003957 |
|  |  | [decreased susceptibility to experimental autoimmune encephalomyelitis](http://www.informatics.jax.org/searches/Phat.cgi?id=MP:0004800) | MP:0004800 |
|  |  | [increased B-1 B cell number](http://www.informatics.jax.org/searches/Phat.cgi?id=MP:0004977) | MP:0004977 |
|  |  | [increased T cell number](http://www.informatics.jax.org/searches/Phat.cgi?id=MP:0005015) | MP:0005015 |
|  |  | [decreased B cell number](http://www.informatics.jax.org/searches/Phat.cgi?id=MP:0005017) | MP:0005017 |
|  |  | [increased double-positive T cell number](http://www.informatics.jax.org/searches/Phat.cgi?id=MP:0005091) | MP:0005091 |
|  |  | [abnormal Langerhans cell physiology](http://www.informatics.jax.org/searches/Phat.cgi?id=MP:0005362) | MP:0005362 |
|  |  | [abnormal CD4-positive T cell physiology](http://www.informatics.jax.org/searches/Phat.cgi?id=MP:0005463) | MP:0005463 |
|  |  | [decreased susceptibility to type I hypersensitivity reaction](http://www.informatics.jax.org/searches/Phat.cgi?id=MP:0005597) | MP:0005597 |
|  |  | [abnormal myeloid dendritic cell morphology](http://www.informatics.jax.org/searches/Phat.cgi?id=MP:0006173) | MP:0006173 |
|  |  | [decreased CD4-positive T cell number](http://www.informatics.jax.org/searches/Phat.cgi?id=MP:0008075) | MP:0008075 |
|  |  | [decreased CD8-positive T cell number](http://www.informatics.jax.org/searches/Phat.cgi?id=MP:0008079) | MP:0008079 |
|  |  | [abnormal follicular dendritic cell antigen presentation](http://www.informatics.jax.org/searches/Phat.cgi?id=MP:0008198) | MP:0008198 |
|  |  | [abnormal peritoneal macrophage morphology](http://www.informatics.jax.org/searches/Phat.cgi?id=MP:0008244) | MP:0008244 |
|  |  | [decreased IgG3 level](http://www.informatics.jax.org/searches/Phat.cgi?id=MP:0008498) | MP:0008498 |
|  |  | [decreased tumor necrosis factor secretion](http://www.informatics.jax.org/searches/Phat.cgi?id=MP:0008561) | MP:0008561 |
|  |  | [decreased interleukin-10 secretion](http://www.informatics.jax.org/searches/Phat.cgi?id=MP:0008661) | MP:0008661 |
|  |  | [decreased interleukin-12 secretion](http://www.informatics.jax.org/searches/Phat.cgi?id=MP:0008664) | MP:0008664 |
|  |  | [decreased interleukin-5 secretion](http://www.informatics.jax.org/searches/Phat.cgi?id=MP:0008703) | MP:0008703 |
|  |  | [abnormal chemokine secretion](http://www.informatics.jax.org/searches/Phat.cgi?id=MP:0008722) | MP:0008722 |
|  |  | [impaired eosinophil recruitment](http://www.informatics.jax.org/searches/Phat.cgi?id=MP:0008723) | MP:0008723 |
|  |  | [abnormal intraepithelial T cell number](http://www.informatics.jax.org/searches/Phat.cgi?id=MP:0008895) | MP:0008895 |
|  | MGI:2179613 | [increased leukocyte cell number](http://www.informatics.jax.org/searches/Phat.cgi?id=MP:0000218) | MP:0000218 |
|  |  | [decreased leukocyte cell number](http://www.informatics.jax.org/searches/Phat.cgi?id=MP:0000221) | MP:0000221 |
|  |  | [abnormal Peyer's patch morphology](http://www.informatics.jax.org/searches/Phat.cgi?id=MP:0000696) | MP:0000696 |
|  |  | [decreased inflammatory response](http://www.informatics.jax.org/searches/Phat.cgi?id=MP:0001876) | MP:0001876 |
|  |  | [abnormal Peyer's patch follicle morphology](http://www.informatics.jax.org/searches/Phat.cgi?id=MP:0002389) | MP:0002389 |
|  |  | [abnormal effector T cell morphology](http://www.informatics.jax.org/searches/Phat.cgi?id=MP:0002435) | MP:0002435 |
|  |  | [abnormal CD4-positive T cell physiology](http://www.informatics.jax.org/searches/Phat.cgi?id=MP:0005463) | MP:0005463 |
|  |  | [abnormal myeloid dendritic cell morphology](http://www.informatics.jax.org/searches/Phat.cgi?id=MP:0006173) | MP:0006173 |
| [Efemp1](http://www.informatics.jax.org/javawi2/servlet/WIFetch?page=markerDetail&id=MGI:1339998) | MGI:3604387 | [no abnormal phenotype detected](http://www.informatics.jax.org/searches/Phat.cgi?id=MP:0002169) | MP:0002169 |
|  | MGI:3776015 | [decreased bone density](http://www.informatics.jax.org/searches/Phat.cgi?id=MP:0000063) | MP:0000063 |
|  |  | [osteoporosis](http://www.informatics.jax.org/searches/Phat.cgi?id=MP:0000066) | MP:0000066 |
|  |  | [abnormal xiphoid process](http://www.informatics.jax.org/searches/Phat.cgi?id=MP:0000159) | MP:0000159 |
|  |  | [kyphosis](http://www.informatics.jax.org/searches/Phat.cgi?id=MP:0000160) | MP:0000160 |
|  |  | [rectal prolapse](http://www.informatics.jax.org/searches/Phat.cgi?id=MP:0000493) | MP:0000493 |
|  |  | [abnormal spleen morphology](http://www.informatics.jax.org/searches/Phat.cgi?id=MP:0000689) | MP:0000689 |
|  |  | [herniated abdominal wall](http://www.informatics.jax.org/searches/Phat.cgi?id=MP:0000757) | MP:0000757 |
|  |  | [abnormal uterus morphology](http://www.informatics.jax.org/searches/Phat.cgi?id=MP:0001120) | MP:0001120 |
|  |  | [abnormal testis morphology](http://www.informatics.jax.org/searches/Phat.cgi?id=MP:0001146) | MP:0001146 |
|  |  | [decreased body size](http://www.informatics.jax.org/searches/Phat.cgi?id=MP:0001265) | MP:0001265 |
|  |  | [abnormal coat appearance](http://www.informatics.jax.org/searches/Phat.cgi?id=MP:0001510) | MP:0001510 |
|  |  | [reduced fertility](http://www.informatics.jax.org/searches/Phat.cgi?id=MP:0001921) | MP:0001921 |
|  |  | [infertility](http://www.informatics.jax.org/searches/Phat.cgi?id=MP:0001924) | MP:0001924 |
|  |  | [decreased litter size](http://www.informatics.jax.org/searches/Phat.cgi?id=MP:0001935) | MP:0001935 |
|  |  | [abnormal hair growth](http://www.informatics.jax.org/searches/Phat.cgi?id=MP:0002073) | MP:0002073 |
|  |  | [premature death](http://www.informatics.jax.org/searches/Phat.cgi?id=MP:0002083) | MP:0002083 |
|  |  | [abnormal eye morphology](http://www.informatics.jax.org/searches/Phat.cgi?id=MP:0002092) | MP:0002092 |
|  |  | [abnormal reproductive system morphology](http://www.informatics.jax.org/searches/Phat.cgi?id=MP:0002160) | MP:0002160 |
|  |  | [other aberrant phenotype](http://www.informatics.jax.org/searches/Phat.cgi?id=MP:0002168) | MP:0002168 |
|  |  | [coarse hair](http://www.informatics.jax.org/searches/Phat.cgi?id=MP:0002832) | MP:0002832 |
|  |  | [liver degeneration](http://www.informatics.jax.org/searches/Phat.cgi?id=MP:0003103) | MP:0003103 |
|  |  | [abnormal body wall morphology](http://www.informatics.jax.org/searches/Phat.cgi?id=MP:0003385) | MP:0003385 |
|  |  | [decreased liver weight](http://www.informatics.jax.org/searches/Phat.cgi?id=MP:0003402) | MP:0003402 |
|  |  | [premature aging](http://www.informatics.jax.org/searches/Phat.cgi?id=MP:0003786) | MP:0003786 |
|  |  | [decreased kidney weight](http://www.informatics.jax.org/searches/Phat.cgi?id=MP:0003918) | MP:0003918 |
|  |  | [decreased skeletal muscle mass](http://www.informatics.jax.org/searches/Phat.cgi?id=MP:0004819) | MP:0004819 |
|  |  | [decreased spleen weight](http://www.informatics.jax.org/searches/Phat.cgi?id=MP:0004953) | MP:0004953 |
|  |  | [kidney atrophy](http://www.informatics.jax.org/searches/Phat.cgi?id=MP:0004970) | MP:0004970 |
|  |  | [premature hair loss](http://www.informatics.jax.org/searches/Phat.cgi?id=MP:0005114) | MP:0005114 |
|  |  | [herniated intestine](http://www.informatics.jax.org/searches/Phat.cgi?id=MP:0005155) | MP:0005155 |
|  |  | [loose skin](http://www.informatics.jax.org/searches/Phat.cgi?id=MP:0005421) | MP:0005421 |
|  |  | [decreased adipose tissue amount](http://www.informatics.jax.org/searches/Phat.cgi?id=MP:0005454) | MP:0005454 |
|  |  | [inguinal hernia](http://www.informatics.jax.org/searches/Phat.cgi?id=MP:0006077) | MP:0006077 |
|  |  | [decreased subcutaneous adipose tissue amount](http://www.informatics.jax.org/searches/Phat.cgi?id=MP:0008844) | MP:0008844 |
| [Kcnh2](http://www.informatics.jax.org/javawi2/servlet/WIFetch?page=markerDetail&id=MGI:1341722) | MGI:2652959 | [decreased heart rate](http://www.informatics.jax.org/searches/Phat.cgi?id=MP:0005333) | MP:0005333 |
| [Lipg](http://www.informatics.jax.org/javawi2/servlet/WIFetch?page=markerDetail&id=MGI:1341803) | MGI:2653887 | [increased circulating HDL cholesterol level](http://www.informatics.jax.org/searches/Phat.cgi?id=MP:0001556) | MP:0001556 |
|  |  | [increased circulating phospholipid level](http://www.informatics.jax.org/searches/Phat.cgi?id=MP:0003980) | MP:0003980 |
|  |  | [increased circulating cholesterol level](http://www.informatics.jax.org/searches/Phat.cgi?id=MP:0005178) | MP:0005178 |
|  |  | [abnormal cholesterol homeostasis](http://www.informatics.jax.org/searches/Phat.cgi?id=MP:0005278) | MP:0005278 |
|  | MGI:2450672 | [increased circulating LDL cholesterol level](http://www.informatics.jax.org/searches/Phat.cgi?id=MP:0000182) | MP:0000182 |
|  |  | [abnormal circulating HDL cholesterol level](http://www.informatics.jax.org/searches/Phat.cgi?id=MP:0000184) | MP:0000184 |
|  |  | [increased circulating triglyceride level](http://www.informatics.jax.org/searches/Phat.cgi?id=MP:0001552) | MP:0001552 |
|  |  | [increased circulating HDL cholesterol level](http://www.informatics.jax.org/searches/Phat.cgi?id=MP:0001556) | MP:0001556 |
|  |  | [increased circulating phospholipid level](http://www.informatics.jax.org/searches/Phat.cgi?id=MP:0003980) | MP:0003980 |
|  |  | [abnormal vascular endothelial cell physiology](http://www.informatics.jax.org/searches/Phat.cgi?id=MP:0004003) | MP:0004003 |
|  |  | [increased circulating VLDL cholesterol level](http://www.informatics.jax.org/searches/Phat.cgi?id=MP:0005145) | MP:0005145 |
|  |  | [increased circulating cholesterol level](http://www.informatics.jax.org/searches/Phat.cgi?id=MP:0005178) | MP:0005178 |
|  |  | [atherosclerotic lesions](http://www.informatics.jax.org/searches/Phat.cgi?id=MP:0005338) | MP:0005338 |
|  |  | [decreased susceptibility to atherosclerosis](http://www.informatics.jax.org/searches/Phat.cgi?id=MP:0005341) | MP:0005341 |
|  | MGI:3529187 |  |  |
| [Map3k1](http://www.informatics.jax.org/javawi2/servlet/WIFetch?page=markerDetail&id=MGI:1346872) | MGI:2179711 | [decreased metastatic potential](http://www.informatics.jax.org/searches/Phat.cgi?id=MP:0001273) | MP:0001273 |
|  |  | [eyelids open at birth](http://www.informatics.jax.org/searches/Phat.cgi?id=MP:0001302) | MP:0001302 |
|  |  | [heart inflammation](http://www.informatics.jax.org/searches/Phat.cgi?id=MP:0001853) | MP:0001853 |
|  |  | [abnormal cell migration](http://www.informatics.jax.org/searches/Phat.cgi?id=MP:0003091) | MP:0003091 |
|  |  | [increased cardiomyocyte apoptosis](http://www.informatics.jax.org/searches/Phat.cgi?id=MP:0003222) | MP:0003222 |
|  |  | [altered tumor morphology](http://www.informatics.jax.org/searches/Phat.cgi?id=MP:0003448) | MP:0003448 |
|  |  | [abnormal heart left ventricle morphology](http://www.informatics.jax.org/searches/Phat.cgi?id=MP:0003921) | MP:0003921 |
|  |  | [increased response of heart to induced stress](http://www.informatics.jax.org/searches/Phat.cgi?id=MP:0004485) | MP:0004485 |
|  |  | [abnormal blood vessel healing](http://www.informatics.jax.org/searches/Phat.cgi?id=MP:0004883) | MP:0004883 |
|  |  | [abnormal vascular smooth muscle physiology](http://www.informatics.jax.org/searches/Phat.cgi?id=MP:0005595) | MP:0005595 |
|  |  | [decreased ventricle muscle contractility](http://www.informatics.jax.org/searches/Phat.cgi?id=MP:0005598) | MP:0005598 |
|  |  | [congestive heart failure](http://www.informatics.jax.org/searches/Phat.cgi?id=MP:0006138) | MP:0006138 |
|  |  | [mammary gland tumor](http://www.informatics.jax.org/searches/Phat.cgi?id=MP:0006318) | MP:0006318 |
|  |  | [abnormal eyelid development](http://www.informatics.jax.org/searches/Phat.cgi?id=MP:0009651) | MP:0009651 |
|  |  | [increased sensitivity to induced morbidity/mortality](http://www.informatics.jax.org/searches/Phat.cgi?id=MP:0009763) | MP:0009763 |
| [Abcg2](http://www.informatics.jax.org/javawi2/servlet/WIFetch?page=markerDetail&id=MGI:1347061) | MGI:2447196 | [abnormal bile color](http://www.informatics.jax.org/searches/Phat.cgi?id=MP:0000341) | MP:0000341 |
|  |  | [increased circulating bilirubin level](http://www.informatics.jax.org/searches/Phat.cgi?id=MP:0005344) | MP:0005344 |
|  |  | [phototoxicity](http://www.informatics.jax.org/searches/Phat.cgi?id=MP:0005653) | MP:0005653 |
|  |  | [porphyria](http://www.informatics.jax.org/searches/Phat.cgi?id=MP:0005654) | MP:0005654 |
|  | MGI:3578727 | [abnormal hematopoietic system morphology/development](http://www.informatics.jax.org/searches/Phat.cgi?id=MP:0002396) | MP:0002396 |
|  |  | [increased physiological sensitivity to xenobiotic](http://www.informatics.jax.org/searches/Phat.cgi?id=MP:0008873) | MP:0008873 |
| [Fto](http://www.informatics.jax.org/javawi2/servlet/WIFetch?page=markerDetail&id=MGI:1347093) | MGI:3841156 | [decreased body length](http://www.informatics.jax.org/searches/Phat.cgi?id=MP:0001258) | MP:0001258 |
|  |  | [decreased body weight](http://www.informatics.jax.org/searches/Phat.cgi?id=MP:0001262) | MP:0001262 |
|  |  | [hypoactivity](http://www.informatics.jax.org/searches/Phat.cgi?id=MP:0001402) | MP:0001402 |
|  |  | [polyphagia](http://www.informatics.jax.org/searches/Phat.cgi?id=MP:0001433) | MP:0001433 |
|  |  | [postnatal growth retardation](http://www.informatics.jax.org/searches/Phat.cgi?id=MP:0001732) | MP:0001732 |
|  |  | [postnatal lethality](http://www.informatics.jax.org/searches/Phat.cgi?id=MP:0002082) | MP:0002082 |
|  |  | [decreased lean body mass](http://www.informatics.jax.org/searches/Phat.cgi?id=MP:0003961) | MP:0003961 |
|  |  | [increased energy expenditure](http://www.informatics.jax.org/searches/Phat.cgi?id=MP:0004889) | MP:0004889 |
|  |  | [increased oxygen consumption](http://www.informatics.jax.org/searches/Phat.cgi?id=MP:0005289) | MP:0005289 |
|  |  | [abnormal circulating hormone level](http://www.informatics.jax.org/searches/Phat.cgi?id=MP:0005418) | MP:0005418 |
|  |  | [decreased adipose tissue amount](http://www.informatics.jax.org/searches/Phat.cgi?id=MP:0005454) | MP:0005454 |
|  |  | [increased resistance to diet-induced obesity](http://www.informatics.jax.org/searches/Phat.cgi?id=MP:0005659) | MP:0005659 |
|  |  | [increased circulating adrenaline level](http://www.informatics.jax.org/searches/Phat.cgi?id=MP:0005662) | MP:0005662 |
|  |  | [decreased circulating leptin level](http://www.informatics.jax.org/searches/Phat.cgi?id=MP:0005668) | MP:0005668 |
|  |  | [increased carbon dioxide production](http://www.informatics.jax.org/searches/Phat.cgi?id=MP:0008963) | MP:0008963 |
|  |  | [increased basal metabolism](http://www.informatics.jax.org/searches/Phat.cgi?id=MP:0008965) | MP:0008965 |
|  |  | [decreased white fat cell size](http://www.informatics.jax.org/searches/Phat.cgi?id=MP:0009133) | MP:0009133 |
|  |  | [decreased gonadal fat pad weight](http://www.informatics.jax.org/searches/Phat.cgi?id=MP:0009283) | MP:0009283 |
| [Slco1b2](http://www.informatics.jax.org/javawi2/servlet/WIFetch?page=markerDetail&id=MGI:1351899) | MGI:3785252 | [abnormal circulating serum albumin level](http://www.informatics.jax.org/searches/Phat.cgi?id=MP:0000199) | MP:0000199 |
|  |  | [abnormal liver physiology](http://www.informatics.jax.org/searches/Phat.cgi?id=MP:0000609) | MP:0000609 |
|  |  | [increased circulating alanine transaminase level](http://www.informatics.jax.org/searches/Phat.cgi?id=MP:0002941) | MP:0002941 |
|  |  | [increased circulating chloride level](http://www.informatics.jax.org/searches/Phat.cgi?id=MP:0003019) | MP:0003019 |
|  |  | [increased circulating cholesterol level](http://www.informatics.jax.org/searches/Phat.cgi?id=MP:0005178) | MP:0005178 |
|  |  | [increased circulating bilirubin level](http://www.informatics.jax.org/searches/Phat.cgi?id=MP:0005344) | MP:0005344 |
|  |  | [abnormal circulating protein level](http://www.informatics.jax.org/searches/Phat.cgi?id=MP:0005416) | MP:0005416 |
|  |  | [increased circulating total protein level](http://www.informatics.jax.org/searches/Phat.cgi?id=MP:0005568) | MP:0005568 |
|  |  | [increased circulating sodium level](http://www.informatics.jax.org/searches/Phat.cgi?id=MP:0005633) | MP:0005633 |
|  |  | [abnormal circulating mineral level](http://www.informatics.jax.org/searches/Phat.cgi?id=MP:0006357) | MP:0006357 |
|  |  | [decreased circulating amylase level](http://www.informatics.jax.org/searches/Phat.cgi?id=MP:0008805) | MP:0008805 |
|  |  | [decreased physiological sensitivity to xenobiotic](http://www.informatics.jax.org/searches/Phat.cgi?id=MP:0008874) | MP:0008874 |
|  |  | [abnormal xenobiotic pharmacokinetics](http://www.informatics.jax.org/searches/Phat.cgi?id=MP:0008875) | MP:0008875 |
|  |  | [decreased sensitivity to xenobiotic induced morbidity/mortality](http://www.informatics.jax.org/searches/Phat.cgi?id=MP:0009767) | MP:0009767 |
| [Esr1](http://www.informatics.jax.org/javawi2/servlet/WIFetch?page=markerDetail&id=MGI:1352467) | MGI:2150897 | [increased white adipose tissue amount](http://www.informatics.jax.org/searches/Phat.cgi?id=MP:0000008) | MP:0000008 |
|  |  | [kyphoscoliosis](http://www.informatics.jax.org/searches/Phat.cgi?id=MP:0000069) | MP:0000069 |
|  |  | [abnormal vertebrae morphology](http://www.informatics.jax.org/searches/Phat.cgi?id=MP:0000137) | MP:0000137 |
|  |  | [abnormal bone marrow cell number](http://www.informatics.jax.org/searches/Phat.cgi?id=MP:0000172) | MP:0000172 |
|  |  | [abnormal circulating cholesterol level](http://www.informatics.jax.org/searches/Phat.cgi?id=MP:0000180) | MP:0000180 |
|  |  | [impaired fertilization](http://www.informatics.jax.org/searches/Phat.cgi?id=MP:0000242) | MP:0000242 |
|  |  | [abnormal stomach glandular epithelium morphology](http://www.informatics.jax.org/searches/Phat.cgi?id=MP:0000473) | MP:0000473 |
|  |  | [cortical renal glomerulopathies](http://www.informatics.jax.org/searches/Phat.cgi?id=MP:0000523) | MP:0000523 |
|  |  | [abnormal hepatocyte morphology](http://www.informatics.jax.org/searches/Phat.cgi?id=MP:0000607) | MP:0000607 |
|  |  | [abnormal mammary gland development](http://www.informatics.jax.org/searches/Phat.cgi?id=MP:0000628) | MP:0000628 |
|  |  | [mammary gland hyperplasia](http://www.informatics.jax.org/searches/Phat.cgi?id=MP:0000630) | MP:0000630 |
|  |  | [decreased thymocyte number](http://www.informatics.jax.org/searches/Phat.cgi?id=MP:0000715) | MP:0000715 |
|  |  | [abnormal hypothalamus morphology](http://www.informatics.jax.org/searches/Phat.cgi?id=MP:0000837) | MP:0000837 |
|  |  | [abnormal uterus morphology](http://www.informatics.jax.org/searches/Phat.cgi?id=MP:0001120) | MP:0001120 |
|  |  | [uterus hypoplasia](http://www.informatics.jax.org/searches/Phat.cgi?id=MP:0001121) | MP:0001121 |
|  |  | [abnormal ovary morphology](http://www.informatics.jax.org/searches/Phat.cgi?id=MP:0001126) | MP:0001126 |
|  |  | [impaired ovarian folliculogenesis](http://www.informatics.jax.org/searches/Phat.cgi?id=MP:0001129) | MP:0001129 |
|  |  | [absent corpus luteum](http://www.informatics.jax.org/searches/Phat.cgi?id=MP:0001134) | MP:0001134 |
|  |  | [abnormal vagina epithelium morphology](http://www.informatics.jax.org/searches/Phat.cgi?id=MP:0001140) | MP:0001140 |
|  |  | [abnormal testis morphology](http://www.informatics.jax.org/searches/Phat.cgi?id=MP:0001146) | MP:0001146 |
|  |  | [seminiferous tubule degeneration](http://www.informatics.jax.org/searches/Phat.cgi?id=MP:0001154) | MP:0001154 |
|  |  | [abnormal spermatogenesis](http://www.informatics.jax.org/searches/Phat.cgi?id=MP:0001156) | MP:0001156 |
|  |  | [increased body length](http://www.informatics.jax.org/searches/Phat.cgi?id=MP:0001257) | MP:0001257 |
|  |  | [decreased body length](http://www.informatics.jax.org/searches/Phat.cgi?id=MP:0001258) | MP:0001258 |
|  |  | [increased body weight](http://www.informatics.jax.org/searches/Phat.cgi?id=MP:0001260) | MP:0001260 |
|  |  | [obese](http://www.informatics.jax.org/searches/Phat.cgi?id=MP:0001261) | MP:0001261 |
|  |  | [decreased body weight](http://www.informatics.jax.org/searches/Phat.cgi?id=MP:0001262) | MP:0001262 |
|  |  | [increased aggression towards females](http://www.informatics.jax.org/searches/Phat.cgi?id=MP:0001356) | MP:0001356 |
|  |  | [abnormal mating receptivity](http://www.informatics.jax.org/searches/Phat.cgi?id=MP:0001376) | MP:0001376 |
|  |  | [reduced male mating frequency](http://www.informatics.jax.org/searches/Phat.cgi?id=MP:0001380) | MP:0001380 |
|  |  | [abnormal pup retrieval](http://www.informatics.jax.org/searches/Phat.cgi?id=MP:0001384) | MP:0001384 |
|  |  | [pup cannibalization](http://www.informatics.jax.org/searches/Phat.cgi?id=MP:0001385) | MP:0001385 |
|  |  | [abnormal maternal nurturing](http://www.informatics.jax.org/searches/Phat.cgi?id=MP:0001386) | MP:0001386 |
|  |  | [hyperactivity](http://www.informatics.jax.org/searches/Phat.cgi?id=MP:0001399) | MP:0001399 |
|  |  | [increased circulating HDL cholesterol level](http://www.informatics.jax.org/searches/Phat.cgi?id=MP:0001556) | MP:0001556 |
|  |  | [hepatic necrosis](http://www.informatics.jax.org/searches/Phat.cgi?id=MP:0001654) | MP:0001654 |
|  |  | [failure of embryo implantation](http://www.informatics.jax.org/searches/Phat.cgi?id=MP:0001728) | MP:0001728 |
|  |  | [increased circulating luteinizing hormone level](http://www.informatics.jax.org/searches/Phat.cgi?id=MP:0001751) | MP:0001751 |
|  |  | [thymus hypoplasia](http://www.informatics.jax.org/searches/Phat.cgi?id=MP:0001823) | MP:0001823 |
|  |  | [liver inflammation](http://www.informatics.jax.org/searches/Phat.cgi?id=MP:0001860) | MP:0001860 |
|  |  | [mammary adenocarcinoma](http://www.informatics.jax.org/searches/Phat.cgi?id=MP:0001883) | MP:0001883 |
|  |  | [mammary gland alveolar hyperplasia](http://www.informatics.jax.org/searches/Phat.cgi?id=MP:0001884) | MP:0001884 |
|  |  | [mammary gland duct hyperplasia](http://www.informatics.jax.org/searches/Phat.cgi?id=MP:0001885) | MP:0001885 |
|  |  | [reduced male fertility](http://www.informatics.jax.org/searches/Phat.cgi?id=MP:0001922) | MP:0001922 |
|  |  | [male infertility](http://www.informatics.jax.org/searches/Phat.cgi?id=MP:0001925) | MP:0001925 |
|  |  | [female infertility](http://www.informatics.jax.org/searches/Phat.cgi?id=MP:0001926) | MP:0001926 |
|  |  | [premature death](http://www.informatics.jax.org/searches/Phat.cgi?id=MP:0002083) | MP:0002083 |
|  |  | [abnormal muscle morphology](http://www.informatics.jax.org/searches/Phat.cgi?id=MP:0002108) | MP:0002108 |
|  |  | [abnormal skeleton development](http://www.informatics.jax.org/searches/Phat.cgi?id=MP:0002113) | MP:0002113 |
|  |  | [abnormal hematopoiesis](http://www.informatics.jax.org/searches/Phat.cgi?id=MP:0002123) | MP:0002123 |
|  |  | [abnormal kidney physiology](http://www.informatics.jax.org/searches/Phat.cgi?id=MP:0002136) | MP:0002136 |
|  |  | [abnormal B cell differentiation](http://www.informatics.jax.org/searches/Phat.cgi?id=MP:0002144) | MP:0002144 |
|  |  | [abnormal innervation](http://www.informatics.jax.org/searches/Phat.cgi?id=MP:0002184) | MP:0002184 |
|  |  | [abnormal seminiferous tubule morphology](http://www.informatics.jax.org/searches/Phat.cgi?id=MP:0002216) | MP:0002216 |
|  |  | [abnormal B cell number](http://www.informatics.jax.org/searches/Phat.cgi?id=MP:0002458) | MP:0002458 |
|  |  | [abnormal immunoglobulin level](http://www.informatics.jax.org/searches/Phat.cgi?id=MP:0002490) | MP:0002490 |
|  |  | [abnormal sexual interaction](http://www.informatics.jax.org/searches/Phat.cgi?id=MP:0002566) | MP:0002566 |
|  |  | [increased vertical activity](http://www.informatics.jax.org/searches/Phat.cgi?id=MP:0002574) | MP:0002574 |
|  |  | [abnormal epididymis morphology](http://www.informatics.jax.org/searches/Phat.cgi?id=MP:0002631) | MP:0002631 |
|  |  | [small uterus](http://www.informatics.jax.org/searches/Phat.cgi?id=MP:0002637) | MP:0002637 |
|  |  | [asthenozoospermia](http://www.informatics.jax.org/searches/Phat.cgi?id=MP:0002675) | MP:0002675 |
|  |  | [oligozoospermia](http://www.informatics.jax.org/searches/Phat.cgi?id=MP:0002687) | MP:0002687 |
|  |  | [abnormal nociception after inflammation](http://www.informatics.jax.org/searches/Phat.cgi?id=MP:0002736) | MP:0002736 |
|  |  | [glomerulonephritis](http://www.informatics.jax.org/searches/Phat.cgi?id=MP:0002743) | MP:0002743 |
|  |  | [short tibia](http://www.informatics.jax.org/searches/Phat.cgi?id=MP:0002764) | MP:0002764 |
|  |  | [decreased circulating luteinizing hormone level](http://www.informatics.jax.org/searches/Phat.cgi?id=MP:0002773) | MP:0002773 |
|  |  | [decreased circulating testosterone level](http://www.informatics.jax.org/searches/Phat.cgi?id=MP:0002780) | MP:0002780 |
|  |  | [increased circulating testosterone level](http://www.informatics.jax.org/searches/Phat.cgi?id=MP:0002781) | MP:0002781 |
|  |  | [decreased circulating follicle stimulating hormone level](http://www.informatics.jax.org/searches/Phat.cgi?id=MP:0002790) | MP:0002790 |
|  |  | [decreased heart weight](http://www.informatics.jax.org/searches/Phat.cgi?id=MP:0002834) | MP:0002834 |
|  |  | [abnormal bone mineralization](http://www.informatics.jax.org/searches/Phat.cgi?id=MP:0002896) | MP:0002896 |
|  |  | [abnormal long bone epiphyseal plate morphology](http://www.informatics.jax.org/searches/Phat.cgi?id=MP:0003055) | MP:0003055 |
|  |  | [short femur](http://www.informatics.jax.org/searches/Phat.cgi?id=MP:0003109) | MP:0003109 |
|  |  | [abnormal response/metabolism to endogenous compounds](http://www.informatics.jax.org/searches/Phat.cgi?id=MP:0003638) | MP:0003638 |
|  |  | [abnormal male reproductive system physiology](http://www.informatics.jax.org/searches/Phat.cgi?id=MP:0003698) | MP:0003698 |
|  |  | [abnormal female reproductive system physiology](http://www.informatics.jax.org/searches/Phat.cgi?id=MP:0003699) | MP:0003699 |
|  |  | [abnormal thymus physiology](http://www.informatics.jax.org/searches/Phat.cgi?id=MP:0003763) | MP:0003763 |
|  |  | [abnormal bone structure](http://www.informatics.jax.org/searches/Phat.cgi?id=MP:0003795) | MP:0003795 |
|  |  | [abnormal cortical bone morphology](http://www.informatics.jax.org/searches/Phat.cgi?id=MP:0003796) | MP:0003796 |
|  |  | [decreased aggression towards males](http://www.informatics.jax.org/searches/Phat.cgi?id=MP:0003862) | MP:0003862 |
|  |  | [impaired passive avoidance behavior](http://www.informatics.jax.org/searches/Phat.cgi?id=MP:0004000) | MP:0004000 |
|  |  | [long femur](http://www.informatics.jax.org/searches/Phat.cgi?id=MP:0004348) | MP:0004348 |
|  |  | [short ulna](http://www.informatics.jax.org/searches/Phat.cgi?id=MP:0004359) | MP:0004359 |
|  |  | [altered response of heart to induced stress](http://www.informatics.jax.org/searches/Phat.cgi?id=MP:0004484) | MP:0004484 |
|  |  | [decreased length of long bones](http://www.informatics.jax.org/searches/Phat.cgi?id=MP:0004686) | MP:0004686 |
|  |  | [decreased circulating insulin-like growth factor I level](http://www.informatics.jax.org/searches/Phat.cgi?id=MP:0004701) | MP:0004701 |
|  |  | [abnormal efferent ductules of testis](http://www.informatics.jax.org/searches/Phat.cgi?id=MP:0004728) | MP:0004728 |
|  |  | [increased anti-double stranded DNA antibody level](http://www.informatics.jax.org/searches/Phat.cgi?id=MP:0004762) | MP:0004762 |
|  |  | [abnormal class switch recombination](http://www.informatics.jax.org/searches/Phat.cgi?id=MP:0004816) | MP:0004816 |
|  |  | [ovary hemorrhage](http://www.informatics.jax.org/searches/Phat.cgi?id=MP:0004834) | MP:0004834 |
|  |  | [abnormal testis weight](http://www.informatics.jax.org/searches/Phat.cgi?id=MP:0004850) | MP:0004850 |
|  |  | [decreased testis weight](http://www.informatics.jax.org/searches/Phat.cgi?id=MP:0004852) | MP:0004852 |
|  |  | [decreased energy expenditure](http://www.informatics.jax.org/searches/Phat.cgi?id=MP:0004890) | MP:0004890 |
|  |  | [uterus atrophy](http://www.informatics.jax.org/searches/Phat.cgi?id=MP:0004894) | MP:0004894 |
|  |  | [abnormal endometrium morphology](http://www.informatics.jax.org/searches/Phat.cgi?id=MP:0004896) | MP:0004896 |
|  |  | [decreased male germ cell number](http://www.informatics.jax.org/searches/Phat.cgi?id=MP:0004901) | MP:0004901 |
|  |  | [abnormal uterus weight](http://www.informatics.jax.org/searches/Phat.cgi?id=MP:0004903) | MP:0004903 |
|  |  | [decreased uterus weight](http://www.informatics.jax.org/searches/Phat.cgi?id=MP:0004905) | MP:0004905 |
|  |  | [abnormal epididymis epithelium morphology](http://www.informatics.jax.org/searches/Phat.cgi?id=MP:0004933) | MP:0004933 |
|  |  | [decreased spleen weight](http://www.informatics.jax.org/searches/Phat.cgi?id=MP:0004953) | MP:0004953 |
|  |  | [decreased thymus weight](http://www.informatics.jax.org/searches/Phat.cgi?id=MP:0004956) | MP:0004956 |
|  |  | [decreased osteoblast cell number](http://www.informatics.jax.org/searches/Phat.cgi?id=MP:0004989) | MP:0004989 |
|  |  | [decreased bone strength](http://www.informatics.jax.org/searches/Phat.cgi?id=MP:0004991) | MP:0004991 |
|  |  | [decreased B cell number](http://www.informatics.jax.org/searches/Phat.cgi?id=MP:0005017) | MP:0005017 |
|  |  | [increased double-positive T cell number](http://www.informatics.jax.org/searches/Phat.cgi?id=MP:0005091) | MP:0005091 |
|  |  | [decreased circulating prolactin level](http://www.informatics.jax.org/searches/Phat.cgi?id=MP:0005121) | MP:0005121 |
|  |  | [cachexia](http://www.informatics.jax.org/searches/Phat.cgi?id=MP:0005150) | MP:0005150 |
|  |  | [proteinuria](http://www.informatics.jax.org/searches/Phat.cgi?id=MP:0005160) | MP:0005160 |
|  |  | [increased circulating cholesterol level](http://www.informatics.jax.org/searches/Phat.cgi?id=MP:0005178) | MP:0005178 |
|  |  | [decreased circulating estradiol level](http://www.informatics.jax.org/searches/Phat.cgi?id=MP:0005181) | MP:0005181 |
|  |  | [increased circulating estradiol level](http://www.informatics.jax.org/searches/Phat.cgi?id=MP:0005182) | MP:0005182 |
|  |  | [abnormal gastric mucosa morphology](http://www.informatics.jax.org/searches/Phat.cgi?id=MP:0005209) | MP:0005209 |
|  |  | [impaired glucose tolerance](http://www.informatics.jax.org/searches/Phat.cgi?id=MP:0005293) | MP:0005293 |
|  |  | [abnormal renal glomerulus morphology](http://www.informatics.jax.org/searches/Phat.cgi?id=MP:0005325) | MP:0005325 |
|  |  | [abnormal mesangial cell](http://www.informatics.jax.org/searches/Phat.cgi?id=MP:0005327) | MP:0005327 |
|  |  | [insulin resistance](http://www.informatics.jax.org/searches/Phat.cgi?id=MP:0005331) | MP:0005331 |
|  |  | [atherosclerotic lesions](http://www.informatics.jax.org/searches/Phat.cgi?id=MP:0005338) | MP:0005338 |
|  |  | [increased susceptibility to atherosclerosis](http://www.informatics.jax.org/searches/Phat.cgi?id=MP:0005339) | MP:0005339 |
|  |  | [abnormal circulating protein level](http://www.informatics.jax.org/searches/Phat.cgi?id=MP:0005416) | MP:0005416 |
|  |  | [hyporesponsive to tactile stimuli](http://www.informatics.jax.org/searches/Phat.cgi?id=MP:0005498) | MP:0005498 |
|  |  | [increased circulating glucose level](http://www.informatics.jax.org/searches/Phat.cgi?id=MP:0005559) | MP:0005559 |
|  |  | [teratozoospermia](http://www.informatics.jax.org/searches/Phat.cgi?id=MP:0005578) | MP:0005578 |
|  |  | [decreased lung weight](http://www.informatics.jax.org/searches/Phat.cgi?id=MP:0005631) | MP:0005631 |
|  |  | [abnormal pituitary gland physiology](http://www.informatics.jax.org/searches/Phat.cgi?id=MP:0005646) | MP:0005646 |
|  |  | [decreased aggression](http://www.informatics.jax.org/searches/Phat.cgi?id=MP:0005656) | MP:0005656 |
|  |  | [increased circulating leptin level](http://www.informatics.jax.org/searches/Phat.cgi?id=MP:0005669) | MP:0005669 |
|  |  | [testis tumor](http://www.informatics.jax.org/searches/Phat.cgi?id=MP:0006262) | MP:0006262 |
|  |  | [abnormal seminiferous epithelium morphology](http://www.informatics.jax.org/searches/Phat.cgi?id=MP:0006304) | MP:0006304 |
|  |  | [abnormal kidney protein excretion](http://www.informatics.jax.org/searches/Phat.cgi?id=MP:0006315) | MP:0006315 |
|  |  | [mammary gland tumor](http://www.informatics.jax.org/searches/Phat.cgi?id=MP:0006318) | MP:0006318 |
|  |  | [decreased long bone epiphyseal plate size](http://www.informatics.jax.org/searches/Phat.cgi?id=MP:0006396) | MP:0006396 |
|  |  | [dilated rete testis](http://www.informatics.jax.org/searches/Phat.cgi?id=MP:0006423) | MP:0006423 |
|  |  | [ovary tumor](http://www.informatics.jax.org/searches/Phat.cgi?id=MP:0008000) | MP:0008000 |
|  |  | [decreased CD4-positive T cell number](http://www.informatics.jax.org/searches/Phat.cgi?id=MP:0008075) | MP:0008075 |
|  |  | [decreased CD8-positive T cell number](http://www.informatics.jax.org/searches/Phat.cgi?id=MP:0008079) | MP:0008079 |
|  |  | [increased plasma cell number](http://www.informatics.jax.org/searches/Phat.cgi?id=MP:0008097) | MP:0008097 |
|  |  | [decreased mature B cell number](http://www.informatics.jax.org/searches/Phat.cgi?id=MP:0008211) | MP:0008211 |
|  |  | [decreased immature B cell number](http://www.informatics.jax.org/searches/Phat.cgi?id=MP:0008215) | MP:0008215 |
|  |  | [abnormal bone ossification](http://www.informatics.jax.org/searches/Phat.cgi?id=MP:0008271) | MP:0008271 |
|  |  | [decreased lactotroph cell number](http://www.informatics.jax.org/searches/Phat.cgi?id=MP:0008332) | MP:0008332 |
|  |  | [increased spleen germinal center number](http://www.informatics.jax.org/searches/Phat.cgi?id=MP:0008481) | MP:0008481 |
|  |  | [abnormal mesonephros morphology](http://www.informatics.jax.org/searches/Phat.cgi?id=MP:0008487) | MP:0008487 |
|  |  | [increased IgG3 level](http://www.informatics.jax.org/searches/Phat.cgi?id=MP:0008502) | MP:0008502 |
|  |  | [abnormal spleen physiology](http://www.informatics.jax.org/searches/Phat.cgi?id=MP:0008737) | MP:0008737 |
|  |  | [abnormal lymph node cell ratio](http://www.informatics.jax.org/searches/Phat.cgi?id=MP:0008828) | MP:0008828 |
|  |  | [anovulation](http://www.informatics.jax.org/searches/Phat.cgi?id=MP:0008869) | MP:0008869 |
|  |  | [abnormal physiological response to xenobiotic](http://www.informatics.jax.org/searches/Phat.cgi?id=MP:0008872) | MP:0008872 |
|  |  | [decreased physiological sensitivity to xenobiotic](http://www.informatics.jax.org/searches/Phat.cgi?id=MP:0008874) | MP:0008874 |
|  |  | [detached sperm flagellum](http://www.informatics.jax.org/searches/Phat.cgi?id=MP:0008893) | MP:0008893 |
|  |  | [increased white fat cell size](http://www.informatics.jax.org/searches/Phat.cgi?id=MP:0009118) | MP:0009118 |
|  |  | [increased white fat cell number](http://www.informatics.jax.org/searches/Phat.cgi?id=MP:0009130) | MP:0009130 |
|  |  | [dilated efferent ductules of testis](http://www.informatics.jax.org/searches/Phat.cgi?id=MP:0009140) | MP:0009140 |
|  |  | [abnormal internal male genitalia morphology](http://www.informatics.jax.org/searches/Phat.cgi?id=MP:0009205) | MP:0009205 |
|  |  | [dilated seminiferous tubules](http://www.informatics.jax.org/searches/Phat.cgi?id=MP:0009257) | MP:0009257 |
|  |  | [increased gonadal fat pad weight](http://www.informatics.jax.org/searches/Phat.cgi?id=MP:0009285) | MP:0009285 |
|  |  | [increased epididymal fat pad weight](http://www.informatics.jax.org/searches/Phat.cgi?id=MP:0009288) | MP:0009288 |
|  |  | [increased inguinal fat pad weight](http://www.informatics.jax.org/searches/Phat.cgi?id=MP:0009292) | MP:0009292 |
|  |  | [increased parametrial fat pad weight](http://www.informatics.jax.org/searches/Phat.cgi?id=MP:0009300) | MP:0009300 |
|  |  | [increased renal fat pad weight](http://www.informatics.jax.org/searches/Phat.cgi?id=MP:0009302) | MP:0009302 |
|  |  | [increased retroperitoneal fat pad weight](http://www.informatics.jax.org/searches/Phat.cgi?id=MP:0009304) | MP:0009304 |
|  |  | [ovarian follicular cyst](http://www.informatics.jax.org/searches/Phat.cgi?id=MP:0009444) | MP:0009444 |
|  |  | [abnormal superovulation](http://www.informatics.jax.org/searches/Phat.cgi?id=MP:0009648) | MP:0009648 |
|  |  | [decreased birth weight](http://www.informatics.jax.org/searches/Phat.cgi?id=MP:0009674) | MP:0009674 |
|  |  | [abnormal chondrocyte physiology](http://www.informatics.jax.org/searches/Phat.cgi?id=MP:0009780) | MP:0009780 |
|  |  | [spermatic granuloma](http://www.informatics.jax.org/searches/Phat.cgi?id=MP:0009824) | MP:0009824 |
|  |  | [delayed tumor appearance](http://www.informatics.jax.org/searches/Phat.cgi?id=MP:0009828) | MP:0009828 |
|  |  | [failure of ejaculation](http://www.informatics.jax.org/searches/Phat.cgi?id=MP:0009856) | MP:0009856 |
|  |  | [increased total body fat amount](http://www.informatics.jax.org/searches/Phat.cgi?id=MP:0010024) | MP:0010024 |
|  |  | [decreased bone mineral density](http://www.informatics.jax.org/searches/Phat.cgi?id=MP:0010121) | MP:0010121 |
|  |  | [decreased bone mineral content](http://www.informatics.jax.org/searches/Phat.cgi?id=MP:0010124) | MP:0010124 |
|  | MGI:2386760 | [uterus hypoplasia](http://www.informatics.jax.org/searches/Phat.cgi?id=MP:0001121) | MP:0001121 |
|  |  | [abnormal ovary morphology](http://www.informatics.jax.org/searches/Phat.cgi?id=MP:0001126) | MP:0001126 |
|  |  | [abnormal ovarian folliculogenesis](http://www.informatics.jax.org/searches/Phat.cgi?id=MP:0001130) | MP:0001130 |
|  |  | [abnormal ovarian follicle morphology](http://www.informatics.jax.org/searches/Phat.cgi?id=MP:0001131) | MP:0001131 |
|  |  | [absent corpus luteum](http://www.informatics.jax.org/searches/Phat.cgi?id=MP:0001134) | MP:0001134 |
|  |  | [abnormal osteoclast physiology](http://www.informatics.jax.org/searches/Phat.cgi?id=MP:0001541) | MP:0001541 |
|  |  | [reduced female fertility](http://www.informatics.jax.org/searches/Phat.cgi?id=MP:0001923) | MP:0001923 |
|  |  | [male infertility](http://www.informatics.jax.org/searches/Phat.cgi?id=MP:0001925) | MP:0001925 |
|  |  | [female infertility](http://www.informatics.jax.org/searches/Phat.cgi?id=MP:0001926) | MP:0001926 |
|  |  | [ovary cysts](http://www.informatics.jax.org/searches/Phat.cgi?id=MP:0002016) | MP:0002016 |
|  |  | [abnormal seminiferous tubule morphology](http://www.informatics.jax.org/searches/Phat.cgi?id=MP:0002216) | MP:0002216 |
|  |  | [abnormal Sertoli cell morphology](http://www.informatics.jax.org/searches/Phat.cgi?id=MP:0002784) | MP:0002784 |
|  |  | [abnormal bone mineralization](http://www.informatics.jax.org/searches/Phat.cgi?id=MP:0002896) | MP:0002896 |
|  |  | [short femur](http://www.informatics.jax.org/searches/Phat.cgi?id=MP:0003109) | MP:0003109 |
|  |  | [impaired granulosa cell differentiation](http://www.informatics.jax.org/searches/Phat.cgi?id=MP:0003357) | MP:0003357 |
|  |  | [abnormal response/metabolism to endogenous compounds](http://www.informatics.jax.org/searches/Phat.cgi?id=MP:0003638) | MP:0003638 |
|  |  | [abnormal bone structure](http://www.informatics.jax.org/searches/Phat.cgi?id=MP:0003795) | MP:0003795 |
|  |  | [decreased bone mass](http://www.informatics.jax.org/searches/Phat.cgi?id=MP:0004016) | MP:0004016 |
|  |  | [decreased circulating insulin-like growth factor I level](http://www.informatics.jax.org/searches/Phat.cgi?id=MP:0004701) | MP:0004701 |
|  |  | [decreased male germ cell number](http://www.informatics.jax.org/searches/Phat.cgi?id=MP:0004901) | MP:0004901 |
|  |  | [decreased uterus weight](http://www.informatics.jax.org/searches/Phat.cgi?id=MP:0004905) | MP:0004905 |
|  |  | [increased circulating estradiol level](http://www.informatics.jax.org/searches/Phat.cgi?id=MP:0005182) | MP:0005182 |
|  |  | [increased susceptibility to atherosclerosis](http://www.informatics.jax.org/searches/Phat.cgi?id=MP:0005339) | MP:0005339 |
|  |  | [abnormal rete testis morphology](http://www.informatics.jax.org/searches/Phat.cgi?id=MP:0006416) | MP:0006416 |
|  |  | [small vagina](http://www.informatics.jax.org/searches/Phat.cgi?id=MP:0008983) | MP:0008983 |
|  |  | [vagina hypoplasia](http://www.informatics.jax.org/searches/Phat.cgi?id=MP:0008984) | MP:0008984 |
|  |  | [absent estrous cycle](http://www.informatics.jax.org/searches/Phat.cgi?id=MP:0009009) | MP:0009009 |
|  |  | [thin uterus](http://www.informatics.jax.org/searches/Phat.cgi?id=MP:0009081) | MP:0009081 |
|  |  | [abnormal secondary ovarian follicle morphology](http://www.informatics.jax.org/searches/Phat.cgi?id=MP:0009363) | MP:0009363 |
|  |  | [increased bone mineral density](http://www.informatics.jax.org/searches/Phat.cgi?id=MP:0010120) | MP:0010120 |
|  |  | [decreased bone mineral density](http://www.informatics.jax.org/searches/Phat.cgi?id=MP:0010121) | MP:0010121 |
|  | MGI:3833893 | [infertility](http://www.informatics.jax.org/searches/Phat.cgi?id=MP:0001924) | MP:0001924 |
|  |  | [abnormal response/metabolism to endogenous compounds](http://www.informatics.jax.org/searches/Phat.cgi?id=MP:0003638) | MP:0003638 |
|  |  | [increased susceptibility to atherosclerosis](http://www.informatics.jax.org/searches/Phat.cgi?id=MP:0005339) | MP:0005339 |
|  | MGI:3842661 |  |  |
| [Angptl3](http://www.informatics.jax.org/javawi2/servlet/WIFetch?page=markerDetail&id=MGI:1353627) | MGI:3610614 | [decreased body size](http://www.informatics.jax.org/searches/Phat.cgi?id=MP:0001265) | MP:0001265 |
|  |  | [perinatal lethality](http://www.informatics.jax.org/searches/Phat.cgi?id=MP:0002081) | MP:0002081 |
|  |  | [premature death](http://www.informatics.jax.org/searches/Phat.cgi?id=MP:0002083) | MP:0002083 |
|  |  | [decreased circulating triglyceride level](http://www.informatics.jax.org/searches/Phat.cgi?id=MP:0002644) | MP:0002644 |
|  |  | [decreased circulating cholesterol level](http://www.informatics.jax.org/searches/Phat.cgi?id=MP:0005179) | MP:0005179 |
|  |  | [abnormal enzyme/coenzyme activity](http://www.informatics.jax.org/searches/Phat.cgi?id=MP:0005584) | MP:0005584 |
|  | MGI:3623491 | [abnormal liver physiology](http://www.informatics.jax.org/searches/Phat.cgi?id=MP:0000609) | MP:0000609 |
|  |  | [decreased circulating triglyceride level](http://www.informatics.jax.org/searches/Phat.cgi?id=MP:0002644) | MP:0002644 |
|  |  | [decreased circulating free fatty acid level](http://www.informatics.jax.org/searches/Phat.cgi?id=MP:0002702) | MP:0002702 |
|  |  | [increased liver weight](http://www.informatics.jax.org/searches/Phat.cgi?id=MP:0002981) | MP:0002981 |
|  |  | [decreased circulating VLDL cholesterol level](http://www.informatics.jax.org/searches/Phat.cgi?id=MP:0005146) | MP:0005146 |
|  |  | [decreased circulating cholesterol level](http://www.informatics.jax.org/searches/Phat.cgi?id=MP:0005179) | MP:0005179 |
|  |  | [decreased epididymal fat pad weight](http://www.informatics.jax.org/searches/Phat.cgi?id=MP:0009289) | MP:0009289 |
|  | MGI:3849576 | [decreased circulating triglyceride level](http://www.informatics.jax.org/searches/Phat.cgi?id=MP:0002644) | MP:0002644 |
|  |  | [decreased circulating cholesterol level](http://www.informatics.jax.org/searches/Phat.cgi?id=MP:0005179) | MP:0005179 |
| [Rgs1](http://www.informatics.jax.org/javawi2/servlet/WIFetch?page=markerDetail&id=MGI:1354694) | MGI:3044786 | [abnormal spleen germinal center morphology](http://www.informatics.jax.org/searches/Phat.cgi?id=MP:0002359) | MP:0002359 |
|  |  | [abnormal dendritic cell physiology](http://www.informatics.jax.org/searches/Phat.cgi?id=MP:0002376) | MP:0002376 |
|  |  | [abnormal B cell physiology](http://www.informatics.jax.org/searches/Phat.cgi?id=MP:0002459) | MP:0002459 |
|  |  | [increased immunoglobulin level](http://www.informatics.jax.org/searches/Phat.cgi?id=MP:0002461) | MP:0002461 |
|  |  | [decreased plasma cell number](http://www.informatics.jax.org/searches/Phat.cgi?id=MP:0008098) | MP:0008098 |
|  |  | [small Peyer's patches](http://www.informatics.jax.org/searches/Phat.cgi?id=MP:0008135) | MP:0008135 |
|  |  | [increased marginal zone B cell number](http://www.informatics.jax.org/searches/Phat.cgi?id=MP:0008181) | MP:0008181 |
| [Icosl](http://www.informatics.jax.org/javawi2/servlet/WIFetch?page=markerDetail&id=MGI:1354701) | MGI:2669961 | [abnormal spleen morphology](http://www.informatics.jax.org/searches/Phat.cgi?id=MP:0000689) | MP:0000689 |
|  |  | [abnormal immune system physiology](http://www.informatics.jax.org/searches/Phat.cgi?id=MP:0001790) | MP:0001790 |
|  |  | [decreased inflammatory response](http://www.informatics.jax.org/searches/Phat.cgi?id=MP:0001876) | MP:0001876 |
|  |  | [abnormal class switch recombination](http://www.informatics.jax.org/searches/Phat.cgi?id=MP:0004816) | MP:0004816 |
|  |  | [abnormal T-helper 2 cell differentiation](http://www.informatics.jax.org/searches/Phat.cgi?id=MP:0008092) | MP:0008092 |
|  |  | [decreased spleen germinal center number](http://www.informatics.jax.org/searches/Phat.cgi?id=MP:0008482) | MP:0008482 |
|  |  | [decreased spleen germinal center size](http://www.informatics.jax.org/searches/Phat.cgi?id=MP:0008484) | MP:0008484 |
|  |  | [decreased IgG1 level](http://www.informatics.jax.org/searches/Phat.cgi?id=MP:0008495) | MP:0008495 |
|  | MGI:2673557 | [abnormal humoral immune response](http://www.informatics.jax.org/searches/Phat.cgi?id=MP:0001800) | MP:0001800 |
|  |  | [decreased IgA level](http://www.informatics.jax.org/searches/Phat.cgi?id=MP:0001807) | MP:0001807 |
|  |  | [abnormal spleen germinal center morphology](http://www.informatics.jax.org/searches/Phat.cgi?id=MP:0002359) | MP:0002359 |
|  |  | [abnormal immune serum protein physiology](http://www.informatics.jax.org/searches/Phat.cgi?id=MP:0002723) | MP:0002723 |
|  |  | [decreased IgG2a level](http://www.informatics.jax.org/searches/Phat.cgi?id=MP:0008496) | MP:0008496 |
|  |  | [decreased IgG2b level](http://www.informatics.jax.org/searches/Phat.cgi?id=MP:0008497) | MP:0008497 |
|  |  | [increased interferon-gamma secretion](http://www.informatics.jax.org/searches/Phat.cgi?id=MP:0008566) | MP:0008566 |
|  |  | [decreased interleukin-10 secretion](http://www.informatics.jax.org/searches/Phat.cgi?id=MP:0008661) | MP:0008661 |
|  |  | [decreased interleukin-4 secretion](http://www.informatics.jax.org/searches/Phat.cgi?id=MP:0008700) | MP:0008700 |
|  | MGI:2683493 | [small spleen](http://www.informatics.jax.org/searches/Phat.cgi?id=MP:0000692) | MP:0000692 |
|  |  | [decreased IgG level](http://www.informatics.jax.org/searches/Phat.cgi?id=MP:0001805) | MP:0001805 |
|  |  | [decreased IgM level](http://www.informatics.jax.org/searches/Phat.cgi?id=MP:0001806) | MP:0001806 |
|  |  | [abnormal T cell differentiation](http://www.informatics.jax.org/searches/Phat.cgi?id=MP:0002145) | MP:0002145 |
|  |  | [abnormal immunoglobulin level](http://www.informatics.jax.org/searches/Phat.cgi?id=MP:0002490) | MP:0002490 |
|  |  | [decreased IgE level](http://www.informatics.jax.org/searches/Phat.cgi?id=MP:0002492) | MP:0002492 |
|  |  | [abnormal T-helper 2 physiology](http://www.informatics.jax.org/searches/Phat.cgi?id=MP:0005466) | MP:0005466 |
|  |  | [decreased IgG1 level](http://www.informatics.jax.org/searches/Phat.cgi?id=MP:0008495) | MP:0008495 |
|  |  | [decreased IgG2a level](http://www.informatics.jax.org/searches/Phat.cgi?id=MP:0008496) | MP:0008496 |
|  |  | [abnormal interleukin secretion](http://www.informatics.jax.org/searches/Phat.cgi?id=MP:0008568) | MP:0008568 |
|  |  | [abnormal interferon level](http://www.informatics.jax.org/searches/Phat.cgi?id=MP:0008750) | MP:0008750 |
|  | MGI:4364624 |  |  |
| [Plek](http://www.informatics.jax.org/javawi2/servlet/WIFetch?page=markerDetail&id=MGI:1860485) | MGI:3845059 | [decreased platelet cell number](http://www.informatics.jax.org/searches/Phat.cgi?id=MP:0003179) | MP:0003179 |
|  |  | [abnormal platelet activation](http://www.informatics.jax.org/searches/Phat.cgi?id=MP:0006298) | MP:0006298 |
|  |  | [decreased platelet aggregation](http://www.informatics.jax.org/searches/Phat.cgi?id=MP:0009549) | MP:0009549 |
| [Apoa5](http://www.informatics.jax.org/javawi2/servlet/WIFetch?page=markerDetail&id=MGI:1913363) | MGI:3586505 | [increased circulating triglyceride level](http://www.informatics.jax.org/searches/Phat.cgi?id=MP:0001552) | MP:0001552 |
|  |  | [increased circulating VLDL cholesterol level](http://www.informatics.jax.org/searches/Phat.cgi?id=MP:0005145) | MP:0005145 |
|  | MGI:3609233 | [no abnormal phenotype detected](http://www.informatics.jax.org/searches/Phat.cgi?id=MP:0002169) | MP:0002169 |
| [Abcg8](http://www.informatics.jax.org/javawi2/servlet/WIFetch?page=markerDetail&id=MGI:1914720) | MGI:2447647 | [abnormal circulating cholesterol level](http://www.informatics.jax.org/searches/Phat.cgi?id=MP:0000180) | MP:0000180 |
|  |  | [increased mean platelet volume](http://www.informatics.jax.org/searches/Phat.cgi?id=MP:0002599) | MP:0002599 |
|  |  | [decreased platelet cell number](http://www.informatics.jax.org/searches/Phat.cgi?id=MP:0003179) | MP:0003179 |
|  |  | [abnormal circulating lipid level](http://www.informatics.jax.org/searches/Phat.cgi?id=MP:0003949) | MP:0003949 |
|  |  | [abnormal bile composition](http://www.informatics.jax.org/searches/Phat.cgi?id=MP:0004773) | MP:0004773 |
|  |  | [decreased circulating cholesterol level](http://www.informatics.jax.org/searches/Phat.cgi?id=MP:0005179) | MP:0005179 |
|  |  | [abnormal cholesterol homeostasis](http://www.informatics.jax.org/searches/Phat.cgi?id=MP:0005278) | MP:0005278 |
|  |  | [abnormal lipid absorption](http://www.informatics.jax.org/searches/Phat.cgi?id=MP:0005342) | MP:0005342 |
|  | MGI:3514037 | [increased circulating triglyceride level](http://www.informatics.jax.org/searches/Phat.cgi?id=MP:0001552) | MP:0001552 |
|  |  | [abnormal liver/biliary system physiology](http://www.informatics.jax.org/searches/Phat.cgi?id=MP:0002139) | MP:0002139 |
|  |  | [decreased cholesterol level](http://www.informatics.jax.org/searches/Phat.cgi?id=MP:0003983) | MP:0003983 |
|  |  | [decreased circulating cholesterol level](http://www.informatics.jax.org/searches/Phat.cgi?id=MP:0005179) | MP:0005179 |
|  |  | [increased circulating plant sterol concentration](http://www.informatics.jax.org/searches/Phat.cgi?id=MP:0010078) | MP:0010078 |
| [Lingo1](http://www.informatics.jax.org/javawi2/servlet/WIFetch?page=markerDetail&id=MGI:1915522) | MGI:3623893 | [abnormal myelination](http://www.informatics.jax.org/searches/Phat.cgi?id=MP:0000920) | MP:0000920 |
| [Ifih1](http://www.informatics.jax.org/javawi2/servlet/WIFetch?page=markerDetail&id=MGI:1918836) | MGI:3663677 | [increased susceptibility to viral infection](http://www.informatics.jax.org/searches/Phat.cgi?id=MP:0002418) | MP:0002418 |
|  |  | [abnormal cytokine secretion](http://www.informatics.jax.org/searches/Phat.cgi?id=MP:0003009) | MP:0003009 |
|  |  | [decreased interferon-alpha secretion](http://www.informatics.jax.org/searches/Phat.cgi?id=MP:0008563) | MP:0008563 |
|  |  | [decreased interleukin-6 secretion](http://www.informatics.jax.org/searches/Phat.cgi?id=MP:0008706) | MP:0008706 |
|  |  | [increased sensitivity to induced morbidity/mortality](http://www.informatics.jax.org/searches/Phat.cgi?id=MP:0009763) | MP:0009763 |
|  | MGI:3801032 | [abnormal dendritic cell physiology](http://www.informatics.jax.org/searches/Phat.cgi?id=MP:0002376) | MP:0002376 |
|  |  | [increased susceptibility to viral infection](http://www.informatics.jax.org/searches/Phat.cgi?id=MP:0002418) | MP:0002418 |
|  |  | [abnormal cell physiology](http://www.informatics.jax.org/searches/Phat.cgi?id=MP:0005621) | MP:0005621 |
|  |  | [abnormal interferon level](http://www.informatics.jax.org/searches/Phat.cgi?id=MP:0008750) | MP:0008750 |
|  |  | [abnormal interleukin level](http://www.informatics.jax.org/searches/Phat.cgi?id=MP:0008751) | MP:0008751 |
| [Atg16l1](http://www.informatics.jax.org/javawi2/servlet/WIFetch?page=markerDetail&id=MGI:1924290) | MGI:3818483 | [neonatal lethality](http://www.informatics.jax.org/searches/Phat.cgi?id=MP:0002058) | MP:0002058 |
|  |  | [abnormal macrophage physiology](http://www.informatics.jax.org/searches/Phat.cgi?id=MP:0002451) | MP:0002451 |
|  |  | [abnormal autophagy](http://www.informatics.jax.org/searches/Phat.cgi?id=MP:0008260) | MP:0008260 |
|  |  | [increased susceptibility to induced colitis](http://www.informatics.jax.org/searches/Phat.cgi?id=MP:0008537) | MP:0008537 |
|  |  | [increased interleukin-1 beta secretion](http://www.informatics.jax.org/searches/Phat.cgi?id=MP:0008657) | MP:0008657 |
|  |  | [increased interleukin-18 secretion](http://www.informatics.jax.org/searches/Phat.cgi?id=MP:0008684) | MP:0008684 |
| [Ubash3a](http://www.informatics.jax.org/javawi2/servlet/WIFetch?page=markerDetail&id=MGI:1926074) | MGI:3051962 | [no abnormal phenotype detected](http://www.informatics.jax.org/searches/Phat.cgi?id=MP:0002169) | MP:0002169 |
|  |  | [abnormal cytokine secretion](http://www.informatics.jax.org/searches/Phat.cgi?id=MP:0003009) | MP:0003009 |
|  |  | [increased susceptibility to experimental autoimmune encephalomyelitis](http://www.informatics.jax.org/searches/Phat.cgi?id=MP:0004799) | MP:0004799 |
|  |  | [increased T cell proliferation](http://www.informatics.jax.org/searches/Phat.cgi?id=MP:0005348) | MP:0005348 |
|  |  | [increased mature B cell number](http://www.informatics.jax.org/searches/Phat.cgi?id=MP:0008210) | MP:0008210 |
|  |  | [increased splenocyte number](http://www.informatics.jax.org/searches/Phat.cgi?id=MP:0009338) | MP:0009338 |
| [Tnip1](http://www.informatics.jax.org/javawi2/servlet/WIFetch?page=markerDetail&id=MGI:1926194) | MGI:3835790 | [decreased hematocrit](http://www.informatics.jax.org/searches/Phat.cgi?id=MP:0000208) | MP:0000208 |
|  |  | [liver hypoplasia](http://www.informatics.jax.org/searches/Phat.cgi?id=MP:0000600) | MP:0000600 |
|  |  | [anemia](http://www.informatics.jax.org/searches/Phat.cgi?id=MP:0001577) | MP:0001577 |
|  |  | [perinatal lethality](http://www.informatics.jax.org/searches/Phat.cgi?id=MP:0002081) | MP:0002081 |
|  |  | [pallor](http://www.informatics.jax.org/searches/Phat.cgi?id=MP:0003717) | MP:0003717 |
|  |  | [increased hepatocyte apoptosis](http://www.informatics.jax.org/searches/Phat.cgi?id=MP:0003887) | MP:0003887 |
|  |  | [decreased fetal size](http://www.informatics.jax.org/searches/Phat.cgi?id=MP:0004200) | MP:0004200 |
|  |  | [increased apoptosis](http://www.informatics.jax.org/searches/Phat.cgi?id=MP:0006042) | MP:0006042 |
|  |  | [decreased fetal weight](http://www.informatics.jax.org/searches/Phat.cgi?id=MP:0009431) | MP:0009431 |
| [Mlxipl](http://www.informatics.jax.org/javawi2/servlet/WIFetch?page=markerDetail&id=MGI:1927999) | MGI:3043871 | [abnormal liver physiology](http://www.informatics.jax.org/searches/Phat.cgi?id=MP:0000609) | MP:0000609 |
|  |  | [decreased brown adipose tissue amount](http://www.informatics.jax.org/searches/Phat.cgi?id=MP:0001780) | MP:0001780 |
|  |  | [decreased white adipose tissue amount](http://www.informatics.jax.org/searches/Phat.cgi?id=MP:0001783) | MP:0001783 |
|  |  | [abnormal glucose homeostasis](http://www.informatics.jax.org/searches/Phat.cgi?id=MP:0002078) | MP:0002078 |
|  |  | [increased circulating insulin level](http://www.informatics.jax.org/searches/Phat.cgi?id=MP:0002079) | MP:0002079 |
|  |  | [abnormal lipid homeostasis](http://www.informatics.jax.org/searches/Phat.cgi?id=MP:0002118) | MP:0002118 |
|  |  | [decreased circulating free fatty acid level](http://www.informatics.jax.org/searches/Phat.cgi?id=MP:0002702) | MP:0002702 |
|  |  | [increased liver weight](http://www.informatics.jax.org/searches/Phat.cgi?id=MP:0002981) | MP:0002981 |
|  |  | [decreased circulating cholesterol level](http://www.informatics.jax.org/searches/Phat.cgi?id=MP:0005179) | MP:0005179 |
|  |  | [decreased fatty acid level](http://www.informatics.jax.org/searches/Phat.cgi?id=MP:0005282) | MP:0005282 |
|  |  | [insulin resistance](http://www.informatics.jax.org/searches/Phat.cgi?id=MP:0005331) | MP:0005331 |
|  |  | [increased glycogen level](http://www.informatics.jax.org/searches/Phat.cgi?id=MP:0005440) | MP:0005440 |
|  |  | [decreased body temperature](http://www.informatics.jax.org/searches/Phat.cgi?id=MP:0005534) | MP:0005534 |
|  |  | [increased circulating glucose level](http://www.informatics.jax.org/searches/Phat.cgi?id=MP:0005559) | MP:0005559 |
|  |  | [decreased epididymal fat pad weight](http://www.informatics.jax.org/searches/Phat.cgi?id=MP:0009289) | MP:0009289 |
|  |  | [increased sensitivity to induced morbidity/mortality](http://www.informatics.jax.org/searches/Phat.cgi?id=MP:0009763) | MP:0009763 |
| [Pcsk9](http://www.informatics.jax.org/javawi2/servlet/WIFetch?page=markerDetail&id=MGI:2140260) | MGI:3577021 | [decreased circulating LDL cholesterol level](http://www.informatics.jax.org/searches/Phat.cgi?id=MP:0000183) | MP:0000183 |
|  |  | [decreased circulating HDL cholesterol level](http://www.informatics.jax.org/searches/Phat.cgi?id=MP:0000186) | MP:0000186 |
|  |  | [decreased circulating cholesterol level](http://www.informatics.jax.org/searches/Phat.cgi?id=MP:0005179) | MP:0005179 |
| [Slc2a9](http://www.informatics.jax.org/javawi2/servlet/WIFetch?page=markerDetail&id=MGI:2152844) | MGI:4361278 | [hydronephrosis](http://www.informatics.jax.org/searches/Phat.cgi?id=MP:0000519) | MP:0000519 |
|  |  | [abnormal kidney cortex](http://www.informatics.jax.org/searches/Phat.cgi?id=MP:0000521) | MP:0000521 |
|  |  | [decreased body weight](http://www.informatics.jax.org/searches/Phat.cgi?id=MP:0001262) | MP:0001262 |
|  |  | [polyuria](http://www.informatics.jax.org/searches/Phat.cgi?id=MP:0001762) | MP:0001762 |
|  |  | [kidney inflammation](http://www.informatics.jax.org/searches/Phat.cgi?id=MP:0001859) | MP:0001859 |
|  |  | [prenatal lethality](http://www.informatics.jax.org/searches/Phat.cgi?id=MP:0002080) | MP:0002080 |
|  |  | [abnormal renal tubule morphology](http://www.informatics.jax.org/searches/Phat.cgi?id=MP:0002703) | MP:0002703 |
|  |  | [tubular nephritis](http://www.informatics.jax.org/searches/Phat.cgi?id=MP:0002704) | MP:0002704 |
|  |  | [dilated renal tubules](http://www.informatics.jax.org/searches/Phat.cgi?id=MP:0002705) | MP:0002705 |
|  |  | [nephrolithiasis](http://www.informatics.jax.org/searches/Phat.cgi?id=MP:0002708) | MP:0002708 |
|  |  | [decreased urine osmolality](http://www.informatics.jax.org/searches/Phat.cgi?id=MP:0002988) | MP:0002988 |
|  |  | [renal interstitial fibrosis](http://www.informatics.jax.org/searches/Phat.cgi?id=MP:0003215) | MP:0003215 |
|  |  | [kidney cysts](http://www.informatics.jax.org/searches/Phat.cgi?id=MP:0003675) | MP:0003675 |
|  |  | [increased drinking behavior](http://www.informatics.jax.org/searches/Phat.cgi?id=MP:0003911) | MP:0003911 |
|  |  | [increased circulating creatinine level](http://www.informatics.jax.org/searches/Phat.cgi?id=MP:0005553) | MP:0005553 |
|  |  | [abnormal kidney excretion](http://www.informatics.jax.org/searches/Phat.cgi?id=MP:0005555) | MP:0005555 |
|  |  | [increased potassium excretion](http://www.informatics.jax.org/searches/Phat.cgi?id=MP:0005619) | MP:0005619 |
|  |  | [abnormal kidney organic anion excretion](http://www.informatics.jax.org/searches/Phat.cgi?id=MP:0006272) | MP:0006272 |
|  |  | [natriuresis](http://www.informatics.jax.org/searches/Phat.cgi?id=MP:0006275) | MP:0006275 |
|  |  | [increased blood uric acid level](http://www.informatics.jax.org/searches/Phat.cgi?id=MP:0008821) | MP:0008821 |
|  |  | [decreased urine pH](http://www.informatics.jax.org/searches/Phat.cgi?id=MP:0009350) | MP:0009350 |
|  |  | [abnormal urine homeostasis](http://www.informatics.jax.org/searches/Phat.cgi?id=MP:0009643) | MP:0009643 |
|  |  | [increased circulating magnesium level](http://www.informatics.jax.org/searches/Phat.cgi?id=MP:0010092) | MP:0010092 |
|  |  | [abnormal renal reabsorption](http://www.informatics.jax.org/searches/Phat.cgi?id=MP:0010107) | MP:0010107 |
|  | MGI:4399337 |  |  |
| [Tnfsf15](http://www.informatics.jax.org/javawi2/servlet/WIFetch?page=markerDetail&id=MGI:2180140) | MGI:3797013 | [decreased susceptibility to experimental autoimmune encephalomyelitis](http://www.informatics.jax.org/searches/Phat.cgi?id=MP:0004800) | MP:0004800 |
|  |  | [abnormal dendritic cell differentiation](http://www.informatics.jax.org/searches/Phat.cgi?id=MP:0008115) | MP:0008115 |
| [Il23r](http://www.informatics.jax.org/javawi2/servlet/WIFetch?page=markerDetail&id=MGI:2181693) | MGI:3665279 | [abnormal response/metabolism to endogenous compounds](http://www.informatics.jax.org/searches/Phat.cgi?id=MP:0003638) | MP:0003638 |
|  | MGI:4355925 | [abnormal CD4-positive T cell physiology](http://www.informatics.jax.org/searches/Phat.cgi?id=MP:0005463) | MP:0005463 |
|  |  | [decreased interleukin-9 secretion](http://www.informatics.jax.org/searches/Phat.cgi?id=MP:0008712) | MP:0008712 |
| [Il27](http://www.informatics.jax.org/javawi2/servlet/WIFetch?page=markerDetail&id=MGI:2384409) | MGI:3842663 | [increased susceptibility to experimental autoimmune encephalomyelitis](http://www.informatics.jax.org/searches/Phat.cgi?id=MP:0004799) | MP:0004799 |
|  |  | [increased susceptibility to type IV hypersensitivity reaction](http://www.informatics.jax.org/searches/Phat.cgi?id=MP:0005617) | MP:0005617 |
|  |  | [abnormal CD4-positive T cell differentiation](http://www.informatics.jax.org/searches/Phat.cgi?id=MP:0008076) | MP:0008076 |
| [Nod2](http://www.informatics.jax.org/javawi2/servlet/WIFetch?page=markerDetail&id=MGI:2429397) | MGI:2680581 | [abnormal macrophage physiology](http://www.informatics.jax.org/searches/Phat.cgi?id=MP:0002451) | MP:0002451 |
|  |  | [decreased susceptibility to endotoxin shock](http://www.informatics.jax.org/searches/Phat.cgi?id=MP:0008734) | MP:0008734 |
|  | MGI:3529594 | [altered susceptibility to infection](http://www.informatics.jax.org/searches/Phat.cgi?id=MP:0001793) | MP:0001793 |
|  |  | [decreased IgG level](http://www.informatics.jax.org/searches/Phat.cgi?id=MP:0001805) | MP:0001805 |
|  |  | [increased susceptibility to bacterial infection](http://www.informatics.jax.org/searches/Phat.cgi?id=MP:0002412) | MP:0002412 |
|  |  | [abnormal macrophage physiology](http://www.informatics.jax.org/searches/Phat.cgi?id=MP:0002451) | MP:0002451 |
|  |  | [decreased tumor necrosis factor secretion](http://www.informatics.jax.org/searches/Phat.cgi?id=MP:0008561) | MP:0008561 |
|  |  | [decreased interleukin-6 secretion](http://www.informatics.jax.org/searches/Phat.cgi?id=MP:0008706) | MP:0008706 |
|  |  | [decreased susceptibility to endotoxin shock](http://www.informatics.jax.org/searches/Phat.cgi?id=MP:0008734) | MP:0008734 |
|  | MGI:3530578 | [weight loss](http://www.informatics.jax.org/searches/Phat.cgi?id=MP:0001263) | MP:0001263 |
|  |  | [abnormal macrophage morphology](http://www.informatics.jax.org/searches/Phat.cgi?id=MP:0002446) | MP:0002446 |
|  |  | [increased susceptibility to induced colitis](http://www.informatics.jax.org/searches/Phat.cgi?id=MP:0008537) | MP:0008537 |
|  |  | [increased interleukin-1 beta secretion](http://www.informatics.jax.org/searches/Phat.cgi?id=MP:0008657) | MP:0008657 |
|  |  | [abnormal cytokine level](http://www.informatics.jax.org/searches/Phat.cgi?id=MP:0008713) | MP:0008713 |
|  |  | [increased sensitivity to xenobiotic induced morbidity/mortality](http://www.informatics.jax.org/searches/Phat.cgi?id=MP:0009766) | MP:0009766 |
|  | MGI:3622092 | [abnormal cytokine secretion](http://www.informatics.jax.org/searches/Phat.cgi?id=MP:0003009) | MP:0003009 |
|  | MGI:3722087 | [abnormal immune system morphology](http://www.informatics.jax.org/searches/Phat.cgi?id=MP:0000685) | MP:0000685 |
|  |  | [abnormal Peyer's patch morphology](http://www.informatics.jax.org/searches/Phat.cgi?id=MP:0000696) | MP:0000696 |
|  |  | [abnormal immune system physiology](http://www.informatics.jax.org/searches/Phat.cgi?id=MP:0001790) | MP:0001790 |
|  |  | [abnormal lymph node B cell domain](http://www.informatics.jax.org/searches/Phat.cgi?id=MP:0002344) | MP:0002344 |
|  |  | [abnormal response to infection](http://www.informatics.jax.org/searches/Phat.cgi?id=MP:0005025) | MP:0005025 |
|  |  | [increased double-negative T cell number](http://www.informatics.jax.org/searches/Phat.cgi?id=MP:0005090) | MP:0005090 |
|  |  | [increased CD4-positive T cell number](http://www.informatics.jax.org/searches/Phat.cgi?id=MP:0008074) | MP:0008074 |
|  |  | [increased Peyer's patch number](http://www.informatics.jax.org/searches/Phat.cgi?id=MP:0008132) | MP:0008132 |
|  |  | [enlarged Peyer's patches](http://www.informatics.jax.org/searches/Phat.cgi?id=MP:0008136) | MP:0008136 |
|  |  | [increased susceptibility to induced colitis](http://www.informatics.jax.org/searches/Phat.cgi?id=MP:0008537) | MP:0008537 |
|  |  | [abnormal cytokine level](http://www.informatics.jax.org/searches/Phat.cgi?id=MP:0008713) | MP:0008713 |
|  |  | [abnormal interferon level](http://www.informatics.jax.org/searches/Phat.cgi?id=MP:0008750) | MP:0008750 |
|  |  | [abnormal interleukin level](http://www.informatics.jax.org/searches/Phat.cgi?id=MP:0008751) | MP:0008751 |
|  |  | [abnormal tumor necrosis factor level](http://www.informatics.jax.org/searches/Phat.cgi?id=MP:0008752) | MP:0008752 |
| [Bank1](http://www.informatics.jax.org/javawi2/servlet/WIFetch?page=markerDetail&id=MGI:2442120) | MGI:3686872 | [abnormal spleen germinal center morphology](http://www.informatics.jax.org/searches/Phat.cgi?id=MP:0002359) | MP:0002359 |
|  |  | [abnormal B cell physiology](http://www.informatics.jax.org/searches/Phat.cgi?id=MP:0002459) | MP:0002459 |
|  |  | [increased IgM level](http://www.informatics.jax.org/searches/Phat.cgi?id=MP:0002494) | MP:0002494 |
|  |  | [abnormal lymphocyte physiology](http://www.informatics.jax.org/searches/Phat.cgi?id=MP:0003945) | MP:0003945 |
|  |  | [increased B cell proliferation](http://www.informatics.jax.org/searches/Phat.cgi?id=MP:0005154) | MP:0005154 |
|  |  | [increased B-1a cell number](http://www.informatics.jax.org/searches/Phat.cgi?id=MP:0008167) | MP:0008167 |
|  |  | [increased mature B cell number](http://www.informatics.jax.org/searches/Phat.cgi?id=MP:0008210) | MP:0008210 |
|  |  | [increased spleen germinal center number](http://www.informatics.jax.org/searches/Phat.cgi?id=MP:0008481) | MP:0008481 |
|  |  | [increased spleen germinal center size](http://www.informatics.jax.org/searches/Phat.cgi?id=MP:0008483) | MP:0008483 |
|  |  | [increased IgG2a level](http://www.informatics.jax.org/searches/Phat.cgi?id=MP:0008500) | MP:0008500 |
|  |  | [abnormal splenic cell ratio](http://www.informatics.jax.org/searches/Phat.cgi?id=MP:0008826) | MP:0008826 |
| [Slc30a8](http://www.informatics.jax.org/javawi2/servlet/WIFetch?page=markerDetail&id=MGI:2442682) | MGI:4362061 | [decreased insulin secretion](http://www.informatics.jax.org/searches/Phat.cgi?id=MP:0003059) | MP:0003059 |
|  |  | [abnormal pancreatic beta cell physiology](http://www.informatics.jax.org/searches/Phat.cgi?id=MP:0003562) | MP:0003562 |
|  |  | [impaired glucose tolerance](http://www.informatics.jax.org/searches/Phat.cgi?id=MP:0005293) | MP:0005293 |
|  |  | [increased circulating glucose level](http://www.informatics.jax.org/searches/Phat.cgi?id=MP:0005559) | MP:0005559 |
|  |  | [increased susceptibility to diet-induced obesity](http://www.informatics.jax.org/searches/Phat.cgi?id=MP:0005658) | MP:0005658 |
|  |  | [abnormal zinc homeostasis](http://www.informatics.jax.org/searches/Phat.cgi?id=MP:0009615) | MP:0009615 |
| [Alpl](http://www.informatics.jax.org/javawi2/servlet/WIFetch?page=markerDetail&id=MGI:87983) | MGI:1857124 | [decreased body size](http://www.informatics.jax.org/searches/Phat.cgi?id=MP:0001265) | MP:0001265 |
|  |  | [seizures](http://www.informatics.jax.org/searches/Phat.cgi?id=MP:0002064) | MP:0002064 |
|  |  | [postnatal lethality](http://www.informatics.jax.org/searches/Phat.cgi?id=MP:0002082) | MP:0002082 |
|  |  | [abnormal tooth morphology](http://www.informatics.jax.org/searches/Phat.cgi?id=MP:0002100) | MP:0002100 |
|  |  | [abnormal blood homeostasis](http://www.informatics.jax.org/searches/Phat.cgi?id=MP:0009642) | MP:0009642 |
|  | MGI:2183411 | [fragile skeleton](http://www.informatics.jax.org/searches/Phat.cgi?id=MP:0000061) | MP:0000061 |
|  |  | [increased bone density](http://www.informatics.jax.org/searches/Phat.cgi?id=MP:0000062) | MP:0000062 |
|  |  | [decreased bone density](http://www.informatics.jax.org/searches/Phat.cgi?id=MP:0000063) | MP:0000063 |
|  |  | [abnormal parietal bone morphology](http://www.informatics.jax.org/searches/Phat.cgi?id=MP:0000109) | MP:0000109 |
|  |  | [abnormal cancellous bone morphology](http://www.informatics.jax.org/searches/Phat.cgi?id=MP:0000130) | MP:0000130 |
|  |  | [abnormal long bone metaphysis morphology](http://www.informatics.jax.org/searches/Phat.cgi?id=MP:0000133) | MP:0000133 |
|  |  | [decreased leukocyte cell number](http://www.informatics.jax.org/searches/Phat.cgi?id=MP:0000221) | MP:0000221 |
|  |  | [abnormal small intestine morphology](http://www.informatics.jax.org/searches/Phat.cgi?id=MP:0000496) | MP:0000496 |
|  |  | [abnormal spleen morphology](http://www.informatics.jax.org/searches/Phat.cgi?id=MP:0000689) | MP:0000689 |
|  |  | [small spleen](http://www.informatics.jax.org/searches/Phat.cgi?id=MP:0000692) | MP:0000692 |
|  |  | [weakness](http://www.informatics.jax.org/searches/Phat.cgi?id=MP:0000746) | MP:0000746 |
|  |  | [abnormal spinal nerve morphology](http://www.informatics.jax.org/searches/Phat.cgi?id=MP:0001077) | MP:0001077 |
|  |  | [lung hemorrhage](http://www.informatics.jax.org/searches/Phat.cgi?id=MP:0001182) | MP:0001182 |
|  |  | [decreased body weight](http://www.informatics.jax.org/searches/Phat.cgi?id=MP:0001262) | MP:0001262 |
|  |  | [impaired coordination](http://www.informatics.jax.org/searches/Phat.cgi?id=MP:0001405) | MP:0001405 |
|  |  | [internal hemorrhage](http://www.informatics.jax.org/searches/Phat.cgi?id=MP:0001634) | MP:0001634 |
|  |  | [abnormal digestion](http://www.informatics.jax.org/searches/Phat.cgi?id=MP:0001664) | MP:0001664 |
|  |  | [intracranial hemorrhage](http://www.informatics.jax.org/searches/Phat.cgi?id=MP:0001915) | MP:0001915 |
|  |  | [apnea](http://www.informatics.jax.org/searches/Phat.cgi?id=MP:0001957) | MP:0001957 |
|  |  | [seizures](http://www.informatics.jax.org/searches/Phat.cgi?id=MP:0002064) | MP:0002064 |
|  |  | [postnatal lethality](http://www.informatics.jax.org/searches/Phat.cgi?id=MP:0002082) | MP:0002082 |
|  |  | [abnormal muscle morphology](http://www.informatics.jax.org/searches/Phat.cgi?id=MP:0002108) | MP:0002108 |
|  |  | [abnormal thymus cortex morphology](http://www.informatics.jax.org/searches/Phat.cgi?id=MP:0002371) | MP:0002371 |
|  |  | [abnormal bone mineralization](http://www.informatics.jax.org/searches/Phat.cgi?id=MP:0002896) | MP:0002896 |
|  |  | [decreased circulating alkaline phosphatase level](http://www.informatics.jax.org/searches/Phat.cgi?id=MP:0002966) | MP:0002966 |
|  |  | [abnormal osteoblast morphology](http://www.informatics.jax.org/searches/Phat.cgi?id=MP:0004986) | MP:0004986 |
|  |  | [abnormal osteoblast physiology](http://www.informatics.jax.org/searches/Phat.cgi?id=MP:0005006) | MP:0005006 |
|  |  | [cachexia](http://www.informatics.jax.org/searches/Phat.cgi?id=MP:0005150) | MP:0005150 |
|  |  | [decreased adipose tissue amount](http://www.informatics.jax.org/searches/Phat.cgi?id=MP:0005454) | MP:0005454 |
|  |  | [decreased long bone epiphyseal plate size](http://www.informatics.jax.org/searches/Phat.cgi?id=MP:0006396) | MP:0006396 |
|  |  | [pale spleen](http://www.informatics.jax.org/searches/Phat.cgi?id=MP:0009246) | MP:0009246 |
| [Ank3](http://www.informatics.jax.org/javawi2/servlet/WIFetch?page=markerDetail&id=MGI:88026) | MGI:2179751 | [tremors](http://www.informatics.jax.org/searches/Phat.cgi?id=MP:0000745) | MP:0000745 |
|  |  | [small cerebellum](http://www.informatics.jax.org/searches/Phat.cgi?id=MP:0000852) | MP:0000852 |
|  |  | [Purkinje cell degeneration](http://www.informatics.jax.org/searches/Phat.cgi?id=MP:0000876) | MP:0000876 |
|  |  | [decreased Purkinje cell number](http://www.informatics.jax.org/searches/Phat.cgi?id=MP:0000880) | MP:0000880 |
|  |  | [thin cerebellar molecular layer](http://www.informatics.jax.org/searches/Phat.cgi?id=MP:0000890) | MP:0000890 |
|  |  | [convulsive seizures](http://www.informatics.jax.org/searches/Phat.cgi?id=MP:0000947) | MP:0000947 |
|  |  | [ataxia](http://www.informatics.jax.org/searches/Phat.cgi?id=MP:0001393) | MP:0001393 |
|  |  | [hypoactivity](http://www.informatics.jax.org/searches/Phat.cgi?id=MP:0001402) | MP:0001402 |
|  |  | [abnormal gait](http://www.informatics.jax.org/searches/Phat.cgi?id=MP:0001406) | MP:0001406 |
|  |  | [premature death](http://www.informatics.jax.org/searches/Phat.cgi?id=MP:0002083) | MP:0002083 |
|  |  | [impaired ability to fire action potentials](http://www.informatics.jax.org/searches/Phat.cgi?id=MP:0002578) | MP:0002578 |
| [Apob](http://www.informatics.jax.org/javawi2/servlet/WIFetch?page=markerDetail&id=MGI:88052) | MGI:1857303 | [decreased circulating LDL cholesterol level](http://www.informatics.jax.org/searches/Phat.cgi?id=MP:0000183) | MP:0000183 |
|  |  | [decreased circulating triglyceride level](http://www.informatics.jax.org/searches/Phat.cgi?id=MP:0002644) | MP:0002644 |
|  | MGI:1857304 | [increased circulating LDL cholesterol level](http://www.informatics.jax.org/searches/Phat.cgi?id=MP:0000182) | MP:0000182 |
|  |  | [increased circulating triglyceride level](http://www.informatics.jax.org/searches/Phat.cgi?id=MP:0001552) | MP:0001552 |
|  |  | [increased circulating insulin level](http://www.informatics.jax.org/searches/Phat.cgi?id=MP:0002079) | MP:0002079 |
|  |  | [decreased circulating cholesterol level](http://www.informatics.jax.org/searches/Phat.cgi?id=MP:0005179) | MP:0005179 |
|  |  | [abnormal retinal pigment epithelium morphology](http://www.informatics.jax.org/searches/Phat.cgi?id=MP:0005201) | MP:0005201 |
|  |  | [impaired glucose tolerance](http://www.informatics.jax.org/searches/Phat.cgi?id=MP:0005293) | MP:0005293 |
|  |  | [atherosclerotic lesions](http://www.informatics.jax.org/searches/Phat.cgi?id=MP:0005338) | MP:0005338 |
|  |  | [abnormal eye electrophysiology](http://www.informatics.jax.org/searches/Phat.cgi?id=MP:0005551) | MP:0005551 |
|  |  | [increased circulating glucose level](http://www.informatics.jax.org/searches/Phat.cgi?id=MP:0005559) | MP:0005559 |
|  | MGI:1857625 | [decreased circulating HDL cholesterol level](http://www.informatics.jax.org/searches/Phat.cgi?id=MP:0000186) | MP:0000186 |
|  |  | [exencephaly](http://www.informatics.jax.org/searches/Phat.cgi?id=MP:0000914) | MP:0000914 |
|  |  | [abnormal embryogenesis/ development](http://www.informatics.jax.org/searches/Phat.cgi?id=MP:0001672) | MP:0001672 |
|  |  | [abnormal yolk sac morphology](http://www.informatics.jax.org/searches/Phat.cgi?id=MP:0001718) | MP:0001718 |
|  |  | [decreased circulating cholesterol level](http://www.informatics.jax.org/searches/Phat.cgi?id=MP:0005179) | MP:0005179 |
|  |  | [abnormal cholesterol homeostasis](http://www.informatics.jax.org/searches/Phat.cgi?id=MP:0005278) | MP:0005278 |
|  |  | [embryonic lethality during organogenesis](http://www.informatics.jax.org/searches/Phat.cgi?id=MP:0006207) | MP:0006207 |
|  | MGI:2157090 | [reduced male fertility](http://www.informatics.jax.org/searches/Phat.cgi?id=MP:0001922) | MP:0001922 |
|  |  | [prenatal lethality](http://www.informatics.jax.org/searches/Phat.cgi?id=MP:0002080) | MP:0002080 |
|  |  | [asthenozoospermia](http://www.informatics.jax.org/searches/Phat.cgi?id=MP:0002675) | MP:0002675 |
|  |  | [oligozoospermia](http://www.informatics.jax.org/searches/Phat.cgi?id=MP:0002687) | MP:0002687 |
|  |  | [impaired acrosome reaction](http://www.informatics.jax.org/searches/Phat.cgi?id=MP:0004542) | MP:0004542 |
| [Apoc1](http://www.informatics.jax.org/javawi2/servlet/WIFetch?page=markerDetail&id=MGI:88053) | MGI:2157218 | [abnormal circulating cholesterol level](http://www.informatics.jax.org/searches/Phat.cgi?id=MP:0000180) | MP:0000180 |
|  |  | [abnormal lipid level](http://www.informatics.jax.org/searches/Phat.cgi?id=MP:0001547) | MP:0001547 |
|  |  | [increased circulating triglyceride level](http://www.informatics.jax.org/searches/Phat.cgi?id=MP:0001552) | MP:0001552 |
|  |  | [increased cholesterol level](http://www.informatics.jax.org/searches/Phat.cgi?id=MP:0003982) | MP:0003982 |
|  |  | [abnormal bile composition](http://www.informatics.jax.org/searches/Phat.cgi?id=MP:0004773) | MP:0004773 |
|  |  | [abnormal phospholipid level](http://www.informatics.jax.org/searches/Phat.cgi?id=MP:0004777) | MP:0004777 |
|  |  | [increased bile salt level](http://www.informatics.jax.org/searches/Phat.cgi?id=MP:0004789) | MP:0004789 |
|  |  | [increased circulating cholesterol level](http://www.informatics.jax.org/searches/Phat.cgi?id=MP:0005178) | MP:0005178 |
|  |  | [increased liver triglyceride level](http://www.informatics.jax.org/searches/Phat.cgi?id=MP:0009355) | MP:0009355 |
|  | MGI:3697323 | [increased circulating cholesterol level](http://www.informatics.jax.org/searches/Phat.cgi?id=MP:0005178) | MP:0005178 |
|  |  | [abnormal cholesterol homeostasis](http://www.informatics.jax.org/searches/Phat.cgi?id=MP:0005278) | MP:0005278 |
| [Apoe](http://www.informatics.jax.org/javawi2/servlet/WIFetch?page=markerDetail&id=MGI:88057) | MGI:1857129 | [abnormal cochlea morphology](http://www.informatics.jax.org/searches/Phat.cgi?id=MP:0000031) | MP:0000031 |
|  |  | [organ of Corti degeneration](http://www.informatics.jax.org/searches/Phat.cgi?id=MP:0000043) | MP:0000043 |
|  |  | [abnormal stria vascularis](http://www.informatics.jax.org/searches/Phat.cgi?id=MP:0000048) | MP:0000048 |
|  |  | [increased bone density](http://www.informatics.jax.org/searches/Phat.cgi?id=MP:0000062) | MP:0000062 |
|  |  | [decreased bone density](http://www.informatics.jax.org/searches/Phat.cgi?id=MP:0000063) | MP:0000063 |
|  |  | [abnormal cancellous bone morphology](http://www.informatics.jax.org/searches/Phat.cgi?id=MP:0000130) | MP:0000130 |
|  |  | [abnormal vertebrae morphology](http://www.informatics.jax.org/searches/Phat.cgi?id=MP:0000137) | MP:0000137 |
|  |  | [abnormal circulating cholesterol level](http://www.informatics.jax.org/searches/Phat.cgi?id=MP:0000180) | MP:0000180 |
|  |  | [increased circulating LDL cholesterol level](http://www.informatics.jax.org/searches/Phat.cgi?id=MP:0000182) | MP:0000182 |
|  |  | [decreased circulating HDL cholesterol level](http://www.informatics.jax.org/searches/Phat.cgi?id=MP:0000186) | MP:0000186 |
|  |  | [decreased hematocrit](http://www.informatics.jax.org/searches/Phat.cgi?id=MP:0000208) | MP:0000208 |
|  |  | [abnormal blood flow velocity](http://www.informatics.jax.org/searches/Phat.cgi?id=MP:0000233) | MP:0000233 |
|  |  | [macrocytosis](http://www.informatics.jax.org/searches/Phat.cgi?id=MP:0000248) | MP:0000248 |
|  |  | [abnormal blood vessel physiology](http://www.informatics.jax.org/searches/Phat.cgi?id=MP:0000249) | MP:0000249 |
|  |  | [echinocytosis](http://www.informatics.jax.org/searches/Phat.cgi?id=MP:0000256) | MP:0000256 |
|  |  | [abnormal heart morphology](http://www.informatics.jax.org/searches/Phat.cgi?id=MP:0000266) | MP:0000266 |
|  |  | [abnormal aorta morphology](http://www.informatics.jax.org/searches/Phat.cgi?id=MP:0000272) | MP:0000272 |
|  |  | [enlarged heart](http://www.informatics.jax.org/searches/Phat.cgi?id=MP:0000274) | MP:0000274 |
|  |  | [abnormal ventricular septum morphology](http://www.informatics.jax.org/searches/Phat.cgi?id=MP:0000281) | MP:0000281 |
|  |  | [abnormal cardiac stroke volume](http://www.informatics.jax.org/searches/Phat.cgi?id=MP:0000304) | MP:0000304 |
|  |  | [altered response to myocardial infarction](http://www.informatics.jax.org/searches/Phat.cgi?id=MP:0000343) | MP:0000343 |
|  |  | [decreased hair follicle number](http://www.informatics.jax.org/searches/Phat.cgi?id=MP:0000379) | MP:0000379 |
|  |  | [alopecia](http://www.informatics.jax.org/searches/Phat.cgi?id=MP:0000414) | MP:0000414 |
|  |  | [partial hair loss](http://www.informatics.jax.org/searches/Phat.cgi?id=MP:0000415) | MP:0000415 |
|  |  | [abnormal femur morphology](http://www.informatics.jax.org/searches/Phat.cgi?id=MP:0000559) | MP:0000559 |
|  |  | [amyloidosis](http://www.informatics.jax.org/searches/Phat.cgi?id=MP:0000604) | MP:0000604 |
|  |  | [abnormal liver physiology](http://www.informatics.jax.org/searches/Phat.cgi?id=MP:0000609) | MP:0000609 |
|  |  | [abnormal adrenal gland morphology](http://www.informatics.jax.org/searches/Phat.cgi?id=MP:0000639) | MP:0000639 |
|  |  | [abnormal immune system morphology](http://www.informatics.jax.org/searches/Phat.cgi?id=MP:0000685) | MP:0000685 |
|  |  | [enlarged spleen](http://www.informatics.jax.org/searches/Phat.cgi?id=MP:0000691) | MP:0000691 |
|  |  | [enlarged lymph nodes](http://www.informatics.jax.org/searches/Phat.cgi?id=MP:0000702) | MP:0000702 |
|  |  | [enlarged thymus](http://www.informatics.jax.org/searches/Phat.cgi?id=MP:0000709) | MP:0000709 |
|  |  | [abnormal skin condition](http://www.informatics.jax.org/searches/Phat.cgi?id=MP:0001191) | MP:0001191 |
|  |  | [thick skin](http://www.informatics.jax.org/searches/Phat.cgi?id=MP:0001200) | MP:0001200 |
|  |  | [skin lesions](http://www.informatics.jax.org/searches/Phat.cgi?id=MP:0001212) | MP:0001212 |
|  |  | [hyperkeratosis](http://www.informatics.jax.org/searches/Phat.cgi?id=MP:0001242) | MP:0001242 |
|  |  | [abnormal dermal layer morphology](http://www.informatics.jax.org/searches/Phat.cgi?id=MP:0001243) | MP:0001243 |
|  |  | [thick dermal layer](http://www.informatics.jax.org/searches/Phat.cgi?id=MP:0001245) | MP:0001245 |
|  |  | [mixed cellular infiltration to dermis](http://www.informatics.jax.org/searches/Phat.cgi?id=MP:0001246) | MP:0001246 |
|  |  | [decreased body length](http://www.informatics.jax.org/searches/Phat.cgi?id=MP:0001258) | MP:0001258 |
|  |  | [decreased body weight](http://www.informatics.jax.org/searches/Phat.cgi?id=MP:0001262) | MP:0001262 |
|  |  | [abnormal retina morphology](http://www.informatics.jax.org/searches/Phat.cgi?id=MP:0001325) | MP:0001325 |
|  |  | [dry eyes](http://www.informatics.jax.org/searches/Phat.cgi?id=MP:0001337) | MP:0001337 |
|  |  | [meibomian gland atrophy](http://www.informatics.jax.org/searches/Phat.cgi?id=MP:0001345) | MP:0001345 |
|  |  | [increased anxiety-related response](http://www.informatics.jax.org/searches/Phat.cgi?id=MP:0001363) | MP:0001363 |
|  |  | [abnormal stationary movement](http://www.informatics.jax.org/searches/Phat.cgi?id=MP:0001388) | MP:0001388 |
|  |  | [abnormal locomotor activity](http://www.informatics.jax.org/searches/Phat.cgi?id=MP:0001392) | MP:0001392 |
|  |  | [abnormal response to new environment](http://www.informatics.jax.org/searches/Phat.cgi?id=MP:0001413) | MP:0001413 |
|  |  | [decreased exploration in new environment](http://www.informatics.jax.org/searches/Phat.cgi?id=MP:0001417) | MP:0001417 |
|  |  | [abnormal spatial learning](http://www.informatics.jax.org/searches/Phat.cgi?id=MP:0001463) | MP:0001463 |
|  |  | [increased startle reflex](http://www.informatics.jax.org/searches/Phat.cgi?id=MP:0001488) | MP:0001488 |
|  |  | [decreased startle reflex](http://www.informatics.jax.org/searches/Phat.cgi?id=MP:0001489) | MP:0001489 |
|  |  | [abnormal kindling response](http://www.informatics.jax.org/searches/Phat.cgi?id=MP:0001499) | MP:0001499 |
|  |  | [abnormal coat appearance](http://www.informatics.jax.org/searches/Phat.cgi?id=MP:0001510) | MP:0001510 |
|  |  | [abnormal skeleton physiology](http://www.informatics.jax.org/searches/Phat.cgi?id=MP:0001533) | MP:0001533 |
|  |  | [abnormal lipid level](http://www.informatics.jax.org/searches/Phat.cgi?id=MP:0001547) | MP:0001547 |
|  |  | [hyperlipidemia](http://www.informatics.jax.org/searches/Phat.cgi?id=MP:0001548) | MP:0001548 |
|  |  | [increased circulating triglyceride level](http://www.informatics.jax.org/searches/Phat.cgi?id=MP:0001552) | MP:0001552 |
|  |  | [increased circulating HDL cholesterol level](http://www.informatics.jax.org/searches/Phat.cgi?id=MP:0001556) | MP:0001556 |
|  |  | [anemia](http://www.informatics.jax.org/searches/Phat.cgi?id=MP:0001577) | MP:0001577 |
|  |  | [abnormal blood vessel morphology](http://www.informatics.jax.org/searches/Phat.cgi?id=MP:0001614) | MP:0001614 |
|  |  | [cardiac hypertrophy](http://www.informatics.jax.org/searches/Phat.cgi?id=MP:0001625) | MP:0001625 |
|  |  | [necrosis](http://www.informatics.jax.org/searches/Phat.cgi?id=MP:0001651) | MP:0001651 |
|  |  | [hypersecretion of corticosterone](http://www.informatics.jax.org/searches/Phat.cgi?id=MP:0001744) | MP:0001744 |
|  |  | [increased circulating corticosterone level](http://www.informatics.jax.org/searches/Phat.cgi?id=MP:0001745) | MP:0001745 |
|  |  | [decreased brown adipose tissue amount](http://www.informatics.jax.org/searches/Phat.cgi?id=MP:0001780) | MP:0001780 |
|  |  | [decreased white adipose tissue amount](http://www.informatics.jax.org/searches/Phat.cgi?id=MP:0001783) | MP:0001783 |
|  |  | [abnormal immune system physiology](http://www.informatics.jax.org/searches/Phat.cgi?id=MP:0001790) | MP:0001790 |
|  |  | [heart inflammation](http://www.informatics.jax.org/searches/Phat.cgi?id=MP:0001853) | MP:0001853 |
|  |  | [lung inflammation](http://www.informatics.jax.org/searches/Phat.cgi?id=MP:0001861) | MP:0001861 |
|  |  | [abnormal lung capacity](http://www.informatics.jax.org/searches/Phat.cgi?id=MP:0001942) | MP:0001942 |
|  |  | [deafness](http://www.informatics.jax.org/searches/Phat.cgi?id=MP:0001967) | MP:0001967 |
|  |  | [abnormal skin morphology](http://www.informatics.jax.org/searches/Phat.cgi?id=MP:0002060) | MP:0002060 |
|  |  | [abnormal fear/anxiety-related behavior](http://www.informatics.jax.org/searches/Phat.cgi?id=MP:0002065) | MP:0002065 |
|  |  | [abnormal eating/drinking behavior](http://www.informatics.jax.org/searches/Phat.cgi?id=MP:0002069) | MP:0002069 |
|  |  | [prenatal lethality](http://www.informatics.jax.org/searches/Phat.cgi?id=MP:0002080) | MP:0002080 |
|  |  | [premature death](http://www.informatics.jax.org/searches/Phat.cgi?id=MP:0002083) | MP:0002083 |
|  |  | [abnormal lipid homeostasis](http://www.informatics.jax.org/searches/Phat.cgi?id=MP:0002118) | MP:0002118 |
|  |  | [abnormal red blood cell](http://www.informatics.jax.org/searches/Phat.cgi?id=MP:0002124) | MP:0002124 |
|  |  | [abnormal cardiovascular system morphology](http://www.informatics.jax.org/searches/Phat.cgi?id=MP:0002127) | MP:0002127 |
|  |  | [abnormal kidney morphology](http://www.informatics.jax.org/searches/Phat.cgi?id=MP:0002135) | MP:0002135 |
|  |  | [abnormal brain morphology](http://www.informatics.jax.org/searches/Phat.cgi?id=MP:0002152) | MP:0002152 |
|  |  | [abnormal gland physiology](http://www.informatics.jax.org/searches/Phat.cgi?id=MP:0002164) | MP:0002164 |
|  |  | [gliosis](http://www.informatics.jax.org/searches/Phat.cgi?id=MP:0002183) | MP:0002183 |
|  |  | [abnormal artery morphology](http://www.informatics.jax.org/searches/Phat.cgi?id=MP:0002191) | MP:0002191 |
|  |  | [neurodegeneration](http://www.informatics.jax.org/searches/Phat.cgi?id=MP:0002229) | MP:0002229 |
|  |  | [abnormal respiratory alveoli morphology](http://www.informatics.jax.org/searches/Phat.cgi?id=MP:0002270) | MP:0002270 |
|  |  | [abnormal respiratory function](http://www.informatics.jax.org/searches/Phat.cgi?id=MP:0002327) | MP:0002327 |
|  |  | [abnormal airway resistance](http://www.informatics.jax.org/searches/Phat.cgi?id=MP:0002328) | MP:0002328 |
|  |  | [abnormal lung compliance](http://www.informatics.jax.org/searches/Phat.cgi?id=MP:0002333) | MP:0002333 |
|  |  | [abnormal dendritic cell physiology](http://www.informatics.jax.org/searches/Phat.cgi?id=MP:0002376) | MP:0002376 |
|  |  | [abnormal cell-mediated immunity](http://www.informatics.jax.org/searches/Phat.cgi?id=MP:0002421) | MP:0002421 |
|  |  | [abnormal macrophage morphology](http://www.informatics.jax.org/searches/Phat.cgi?id=MP:0002446) | MP:0002446 |
|  |  | [abnormal erythrocyte morphology](http://www.informatics.jax.org/searches/Phat.cgi?id=MP:0002447) | MP:0002447 |
|  |  | [abnormal macrophage physiology](http://www.informatics.jax.org/searches/Phat.cgi?id=MP:0002451) | MP:0002451 |
|  |  | [increased immunoglobulin level](http://www.informatics.jax.org/searches/Phat.cgi?id=MP:0002461) | MP:0002461 |
|  |  | [increased IgG level](http://www.informatics.jax.org/searches/Phat.cgi?id=MP:0002493) | MP:0002493 |
|  |  | [increased IgM level](http://www.informatics.jax.org/searches/Phat.cgi?id=MP:0002494) | MP:0002494 |
|  |  | [reticulocytosis](http://www.informatics.jax.org/searches/Phat.cgi?id=MP:0002640) | MP:0002640 |
|  |  | [poikilocytosis](http://www.informatics.jax.org/searches/Phat.cgi?id=MP:0002643) | MP:0002643 |
|  |  | [decreased circulating triglyceride level](http://www.informatics.jax.org/searches/Phat.cgi?id=MP:0002644) | MP:0002644 |
|  |  | [abnormal sciatic nerve](http://www.informatics.jax.org/searches/Phat.cgi?id=MP:0002651) | MP:0002651 |
|  |  | [abnormal renal tubule morphology](http://www.informatics.jax.org/searches/Phat.cgi?id=MP:0002703) | MP:0002703 |
|  |  | [abnormal immune serum protein physiology](http://www.informatics.jax.org/searches/Phat.cgi?id=MP:0002723) | MP:0002723 |
|  |  | [abnormal thermal nociception](http://www.informatics.jax.org/searches/Phat.cgi?id=MP:0002733) | MP:0002733 |
|  |  | [glomerulonephritis](http://www.informatics.jax.org/searches/Phat.cgi?id=MP:0002743) | MP:0002743 |
|  |  | [dilated heart left ventricle](http://www.informatics.jax.org/searches/Phat.cgi?id=MP:0002753) | MP:0002753 |
|  |  | [decreased vertical activity](http://www.informatics.jax.org/searches/Phat.cgi?id=MP:0002757) | MP:0002757 |
|  |  | [abnormal retinal vasculature morphology](http://www.informatics.jax.org/searches/Phat.cgi?id=MP:0002792) | MP:0002792 |
|  |  | [increased thigmotaxis](http://www.informatics.jax.org/searches/Phat.cgi?id=MP:0002797) | MP:0002797 |
|  |  | [increased heart weight](http://www.informatics.jax.org/searches/Phat.cgi?id=MP:0002833) | MP:0002833 |
|  |  | [decreased blood pressure](http://www.informatics.jax.org/searches/Phat.cgi?id=MP:0002843) | MP:0002843 |
|  |  | [cochlear ganglion degeneration](http://www.informatics.jax.org/searches/Phat.cgi?id=MP:0002857) | MP:0002857 |
|  |  | [albuminuria](http://www.informatics.jax.org/searches/Phat.cgi?id=MP:0002871) | MP:0002871 |
|  |  | [decreased hemoglobin content](http://www.informatics.jax.org/searches/Phat.cgi?id=MP:0002874) | MP:0002874 |
|  |  | [increased insulin sensitivity](http://www.informatics.jax.org/searches/Phat.cgi?id=MP:0002891) | MP:0002891 |
|  |  | [thick ventricular wall](http://www.informatics.jax.org/searches/Phat.cgi?id=MP:0002953) | MP:0002953 |
|  |  | [decreased infarction size](http://www.informatics.jax.org/searches/Phat.cgi?id=MP:0003038) | MP:0003038 |
|  |  | [abnormal impulse conducting system conduction](http://www.informatics.jax.org/searches/Phat.cgi?id=MP:0003137) | MP:0003137 |
|  |  | [cardiac fibrosis](http://www.informatics.jax.org/searches/Phat.cgi?id=MP:0003141) | MP:0003141 |
|  |  | [abnormal tectorial membrane morphology](http://www.informatics.jax.org/searches/Phat.cgi?id=MP:0003149) | MP:0003149 |
|  |  | [impaired muscle relaxation](http://www.informatics.jax.org/searches/Phat.cgi?id=MP:0003157) | MP:0003157 |
|  |  | [amyloid beta deposits](http://www.informatics.jax.org/searches/Phat.cgi?id=MP:0003329) | MP:0003329 |
|  |  | [hypopituitarism](http://www.informatics.jax.org/searches/Phat.cgi?id=MP:0003348) | MP:0003348 |
|  |  | [increased cardiac output](http://www.informatics.jax.org/searches/Phat.cgi?id=MP:0003394) | MP:0003394 |
|  |  | [abnormal nervous system physiology](http://www.informatics.jax.org/searches/Phat.cgi?id=MP:0003633) | MP:0003633 |
|  |  | [abnormal response/metabolism to endogenous compounds](http://www.informatics.jax.org/searches/Phat.cgi?id=MP:0003638) | MP:0003638 |
|  |  | [abnormal axon outgrowth](http://www.informatics.jax.org/searches/Phat.cgi?id=MP:0003651) | MP:0003651 |
|  |  | [oxidative stress](http://www.informatics.jax.org/searches/Phat.cgi?id=MP:0003674) | MP:0003674 |
|  |  | [abnormal glial cell physiology](http://www.informatics.jax.org/searches/Phat.cgi?id=MP:0003690) | MP:0003690 |
|  |  | [xanthoma](http://www.informatics.jax.org/searches/Phat.cgi?id=MP:0003692) | MP:0003692 |
|  |  | [increased autoantibody level](http://www.informatics.jax.org/searches/Phat.cgi?id=MP:0003725) | MP:0003725 |
|  |  | [decreased autoantibody level](http://www.informatics.jax.org/searches/Phat.cgi?id=MP:0003726) | MP:0003726 |
|  |  | [abnormal retinal inner nuclear layer morphology](http://www.informatics.jax.org/searches/Phat.cgi?id=MP:0003733) | MP:0003733 |
|  |  | [abnormal bone structure](http://www.informatics.jax.org/searches/Phat.cgi?id=MP:0003795) | MP:0003795 |
|  |  | [vascular smooth muscle cell hypoplasia](http://www.informatics.jax.org/searches/Phat.cgi?id=MP:0003814) | MP:0003814 |
|  |  | [enhanced coordination](http://www.informatics.jax.org/searches/Phat.cgi?id=MP:0003858) | MP:0003858 |
|  |  | [abnormal myelin sheath morphology](http://www.informatics.jax.org/searches/Phat.cgi?id=MP:0003871) | MP:0003871 |
|  |  | [decreased macrophage cell number](http://www.informatics.jax.org/searches/Phat.cgi?id=MP:0003884) | MP:0003884 |
|  |  | [abnormal ST interval](http://www.informatics.jax.org/searches/Phat.cgi?id=MP:0003897) | MP:0003897 |
|  |  | [increased eating behavior](http://www.informatics.jax.org/searches/Phat.cgi?id=MP:0003909) | MP:0003909 |
|  |  | [increased drinking behavior](http://www.informatics.jax.org/searches/Phat.cgi?id=MP:0003911) | MP:0003911 |
|  |  | [decreased kidney weight](http://www.informatics.jax.org/searches/Phat.cgi?id=MP:0003918) | MP:0003918 |
|  |  | [abnormal cholesterol level](http://www.informatics.jax.org/searches/Phat.cgi?id=MP:0003947) | MP:0003947 |
|  |  | [abnormal circulating lipid level](http://www.informatics.jax.org/searches/Phat.cgi?id=MP:0003949) | MP:0003949 |
|  |  | [increased circulating VLDL triglyceride level](http://www.informatics.jax.org/searches/Phat.cgi?id=MP:0003975) | MP:0003975 |
|  |  | [increased circulating phospholipid level](http://www.informatics.jax.org/searches/Phat.cgi?id=MP:0003980) | MP:0003980 |
|  |  | [decreased cholesterol level](http://www.informatics.jax.org/searches/Phat.cgi?id=MP:0003983) | MP:0003983 |
|  |  | [arteriosclerosis](http://www.informatics.jax.org/searches/Phat.cgi?id=MP:0003991) | MP:0003991 |
|  |  | [aortic dissection](http://www.informatics.jax.org/searches/Phat.cgi?id=MP:0004044) | MP:0004044 |
|  |  | [abnormal coronary artery morphology](http://www.informatics.jax.org/searches/Phat.cgi?id=MP:0004111) | MP:0004111 |
|  |  | [abnormal aortic arch morphology](http://www.informatics.jax.org/searches/Phat.cgi?id=MP:0004113) | MP:0004113 |
|  |  | [increased cortical bone thickness](http://www.informatics.jax.org/searches/Phat.cgi?id=MP:0004148) | MP:0004148 |
|  |  | [abnormal basilar membrane](http://www.informatics.jax.org/searches/Phat.cgi?id=MP:0004308) | MP:0004308 |
|  |  | [cochlear inner hair cell degeneration](http://www.informatics.jax.org/searches/Phat.cgi?id=MP:0004398) | MP:0004398 |
|  |  | [cochlear outer hair cell degeneration](http://www.informatics.jax.org/searches/Phat.cgi?id=MP:0004404) | MP:0004404 |
|  |  | [abnormal spiral modiolar artery morphology](http://www.informatics.jax.org/searches/Phat.cgi?id=MP:0004629) | MP:0004629 |
|  |  | [spiral modiolar artery stenosis](http://www.informatics.jax.org/searches/Phat.cgi?id=MP:0004630) | MP:0004630 |
|  |  | [increased anti-double stranded DNA antibody level](http://www.informatics.jax.org/searches/Phat.cgi?id=MP:0004762) | MP:0004762 |
|  |  | [decreased brainstem auditory evoked potential](http://www.informatics.jax.org/searches/Phat.cgi?id=MP:0004765) | MP:0004765 |
|  |  | [abnormal synaptic vesicle morphology](http://www.informatics.jax.org/searches/Phat.cgi?id=MP:0004769) | MP:0004769 |
|  |  | [increased macrophage derived foam cell number](http://www.informatics.jax.org/searches/Phat.cgi?id=MP:0004778) | MP:0004778 |
|  |  | [increased anti-nuclear antigen antibody level](http://www.informatics.jax.org/searches/Phat.cgi?id=MP:0004794) | MP:0004794 |
|  |  | [increased susceptibility to systemic lupus erythematosus](http://www.informatics.jax.org/searches/Phat.cgi?id=MP:0004801) | MP:0004801 |
|  |  | [increased mean arterial blood pressure](http://www.informatics.jax.org/searches/Phat.cgi?id=MP:0004875) | MP:0004875 |
|  |  | [decreased systemic vascular resistance](http://www.informatics.jax.org/searches/Phat.cgi?id=MP:0004879) | MP:0004879 |
|  |  | [abnormal blood vessel healing](http://www.informatics.jax.org/searches/Phat.cgi?id=MP:0004883) | MP:0004883 |
|  |  | [increased uterus weight](http://www.informatics.jax.org/searches/Phat.cgi?id=MP:0004904) | MP:0004904 |
|  |  | [skin inflammation](http://www.informatics.jax.org/searches/Phat.cgi?id=MP:0004947) | MP:0004947 |
|  |  | [increased spleen weight](http://www.informatics.jax.org/searches/Phat.cgi?id=MP:0004952) | MP:0004952 |
|  |  | [abnormal osteoblast physiology](http://www.informatics.jax.org/searches/Phat.cgi?id=MP:0005006) | MP:0005006 |
|  |  | [increased B cell number](http://www.informatics.jax.org/searches/Phat.cgi?id=MP:0005014) | MP:0005014 |
|  |  | [increased susceptibility to parasitic infection](http://www.informatics.jax.org/searches/Phat.cgi?id=MP:0005027) | MP:0005027 |
|  |  | [thrombosis](http://www.informatics.jax.org/searches/Phat.cgi?id=MP:0005048) | MP:0005048 |
|  |  | [decreased acute inflammation](http://www.informatics.jax.org/searches/Phat.cgi?id=MP:0005087) | MP:0005087 |
|  |  | [decreased cardiac muscle contractility](http://www.informatics.jax.org/searches/Phat.cgi?id=MP:0005140) | MP:0005140 |
|  |  | [increased circulating VLDL cholesterol level](http://www.informatics.jax.org/searches/Phat.cgi?id=MP:0005145) | MP:0005145 |
|  |  | [decreased circulating VLDL cholesterol level](http://www.informatics.jax.org/searches/Phat.cgi?id=MP:0005146) | MP:0005146 |
|  |  | [proteinuria](http://www.informatics.jax.org/searches/Phat.cgi?id=MP:0005160) | MP:0005160 |
|  |  | [abnormal blood-brain barrier function](http://www.informatics.jax.org/searches/Phat.cgi?id=MP:0005167) | MP:0005167 |
|  |  | [increased circulating cholesterol level](http://www.informatics.jax.org/searches/Phat.cgi?id=MP:0005178) | MP:0005178 |
|  |  | [decreased circulating cholesterol level](http://www.informatics.jax.org/searches/Phat.cgi?id=MP:0005179) | MP:0005179 |
|  |  | [abnormal cholesterol homeostasis](http://www.informatics.jax.org/searches/Phat.cgi?id=MP:0005278) | MP:0005278 |
|  |  | [improved glucose tolerance](http://www.informatics.jax.org/searches/Phat.cgi?id=MP:0005292) | MP:0005292 |
|  |  | [increased triglyceride level](http://www.informatics.jax.org/searches/Phat.cgi?id=MP:0005317) | MP:0005317 |
|  |  | [abnormal enzyme/ coenzyme level](http://www.informatics.jax.org/searches/Phat.cgi?id=MP:0005319) | MP:0005319 |
|  |  | [abnormal renal glomerulus morphology](http://www.informatics.jax.org/searches/Phat.cgi?id=MP:0005325) | MP:0005325 |
|  |  | [abnormal gonadal fat pad morphology](http://www.informatics.jax.org/searches/Phat.cgi?id=MP:0005335) | MP:0005335 |
|  |  | [atherosclerotic lesions](http://www.informatics.jax.org/searches/Phat.cgi?id=MP:0005338) | MP:0005338 |
|  |  | [increased susceptibility to atherosclerosis](http://www.informatics.jax.org/searches/Phat.cgi?id=MP:0005339) | MP:0005339 |
|  |  | [altered susceptibility to atherosclerosis](http://www.informatics.jax.org/searches/Phat.cgi?id=MP:0005340) | MP:0005340 |
|  |  | [decreased susceptibility to atherosclerosis](http://www.informatics.jax.org/searches/Phat.cgi?id=MP:0005341) | MP:0005341 |
|  |  | [abnormal fat-soluble vitamin level](http://www.informatics.jax.org/searches/Phat.cgi?id=MP:0005401) | MP:0005401 |
|  |  | [vascular stenosis](http://www.informatics.jax.org/searches/Phat.cgi?id=MP:0005412) | MP:0005412 |
|  |  | [abnormal circulating protein level](http://www.informatics.jax.org/searches/Phat.cgi?id=MP:0005416) | MP:0005416 |
|  |  | [increased weight gain](http://www.informatics.jax.org/searches/Phat.cgi?id=MP:0005455) | MP:0005455 |
|  |  | [vascular smooth muscle cell hyperplasia](http://www.informatics.jax.org/searches/Phat.cgi?id=MP:0005489) | MP:0005489 |
|  |  | [abnormal macrophage recruitment](http://www.informatics.jax.org/searches/Phat.cgi?id=MP:0005495) | MP:0005495 |
|  |  | [impaired macrophage recruitment](http://www.informatics.jax.org/searches/Phat.cgi?id=MP:0005496) | MP:0005496 |
|  |  | [abnormal eye electrophysiology](http://www.informatics.jax.org/searches/Phat.cgi?id=MP:0005551) | MP:0005551 |
|  |  | [increased circulating creatinine level](http://www.informatics.jax.org/searches/Phat.cgi?id=MP:0005553) | MP:0005553 |
|  |  | [increased circulating glucose level](http://www.informatics.jax.org/searches/Phat.cgi?id=MP:0005559) | MP:0005559 |
|  |  | [decreased circulating glucose level](http://www.informatics.jax.org/searches/Phat.cgi?id=MP:0005560) | MP:0005560 |
|  |  | [abnormal enzyme/coenzyme activity](http://www.informatics.jax.org/searches/Phat.cgi?id=MP:0005584) | MP:0005584 |
|  |  | [decreased vasodilation](http://www.informatics.jax.org/searches/Phat.cgi?id=MP:0005591) | MP:0005591 |
|  |  | [abnormal vascular smooth muscle physiology](http://www.informatics.jax.org/searches/Phat.cgi?id=MP:0005595) | MP:0005595 |
|  |  | [decreased susceptibility to type IV hypersensitivity reaction](http://www.informatics.jax.org/searches/Phat.cgi?id=MP:0005616) | MP:0005616 |
|  |  | [increased resistance to diet-induced obesity](http://www.informatics.jax.org/searches/Phat.cgi?id=MP:0005659) | MP:0005659 |
|  |  | [decreased circulating leptin level](http://www.informatics.jax.org/searches/Phat.cgi?id=MP:0005668) | MP:0005668 |
|  |  | [abnormal vascular endothelial cell morphology](http://www.informatics.jax.org/searches/Phat.cgi?id=MP:0006055) | MP:0006055 |
|  |  | [decreased cerebral infarction size](http://www.informatics.jax.org/searches/Phat.cgi?id=MP:0006058) | MP:0006058 |
|  |  | [increased cerebral infarction size](http://www.informatics.jax.org/searches/Phat.cgi?id=MP:0006060) | MP:0006060 |
|  |  | [abnormal circulating homocysteine level](http://www.informatics.jax.org/searches/Phat.cgi?id=MP:0006076) | MP:0006076 |
|  |  | [arterial calcification](http://www.informatics.jax.org/searches/Phat.cgi?id=MP:0006133) | MP:0006133 |
|  |  | [arterial occlusion](http://www.informatics.jax.org/searches/Phat.cgi?id=MP:0006134) | MP:0006134 |
|  |  | [increased systolic blood pressure](http://www.informatics.jax.org/searches/Phat.cgi?id=MP:0006144) | MP:0006144 |
|  |  | [decreased systolic blood pressure](http://www.informatics.jax.org/searches/Phat.cgi?id=MP:0006264) | MP:0006264 |
|  |  | [aortic aneurysm](http://www.informatics.jax.org/searches/Phat.cgi?id=MP:0006278) | MP:0006278 |
|  |  | [abnormal T cell number](http://www.informatics.jax.org/searches/Phat.cgi?id=MP:0006387) | MP:0006387 |
|  |  | [delayed cellular replicative senescence](http://www.informatics.jax.org/searches/Phat.cgi?id=MP:0008009) | MP:0008009 |
|  |  | [impaired lipolysis](http://www.informatics.jax.org/searches/Phat.cgi?id=MP:0008033) | MP:0008033 |
|  |  | [abnormal CD4-positive T cell number](http://www.informatics.jax.org/searches/Phat.cgi?id=MP:0008073) | MP:0008073 |
|  |  | [increased CD4-positive T cell number](http://www.informatics.jax.org/searches/Phat.cgi?id=MP:0008074) | MP:0008074 |
|  |  | [decreased CD4-positive T cell number](http://www.informatics.jax.org/searches/Phat.cgi?id=MP:0008075) | MP:0008075 |
|  |  | [decreased CD8-positive T cell number](http://www.informatics.jax.org/searches/Phat.cgi?id=MP:0008079) | MP:0008079 |
|  |  | [increased T-helper 2 cell number](http://www.informatics.jax.org/searches/Phat.cgi?id=MP:0008090) | MP:0008090 |
|  |  | [abnormal dendrite morphology](http://www.informatics.jax.org/searches/Phat.cgi?id=MP:0008143) | MP:0008143 |
|  |  | [decreased follicular B cell number](http://www.informatics.jax.org/searches/Phat.cgi?id=MP:0008174) | MP:0008174 |
|  |  | [increased marginal zone B cell number](http://www.informatics.jax.org/searches/Phat.cgi?id=MP:0008181) | MP:0008181 |
|  |  | [decreased marginal zone B cell number](http://www.informatics.jax.org/searches/Phat.cgi?id=MP:0008182) | MP:0008182 |
|  |  | [abnormal B cell activation](http://www.informatics.jax.org/searches/Phat.cgi?id=MP:0008217) | MP:0008217 |
|  |  | [abnormal adrenal cortex morphology](http://www.informatics.jax.org/searches/Phat.cgi?id=MP:0008288) | MP:0008288 |
|  |  | [abnormal adrenal medulla morphology](http://www.informatics.jax.org/searches/Phat.cgi?id=MP:0008289) | MP:0008289 |
|  |  | [hypochromic macrocytic anemia](http://www.informatics.jax.org/searches/Phat.cgi?id=MP:0008389) | MP:0008389 |
|  |  | [abnormal spatial working memory](http://www.informatics.jax.org/searches/Phat.cgi?id=MP:0008428) | MP:0008428 |
|  |  | [thin retinal inner nuclear layer](http://www.informatics.jax.org/searches/Phat.cgi?id=MP:0008511) | MP:0008511 |
|  |  | [thin retinal outer nuclear layer](http://www.informatics.jax.org/searches/Phat.cgi?id=MP:0008515) | MP:0008515 |
|  |  | [impaired olfaction](http://www.informatics.jax.org/searches/Phat.cgi?id=MP:0008544) | MP:0008544 |
|  |  | [decreased interferon-gamma secretion](http://www.informatics.jax.org/searches/Phat.cgi?id=MP:0008567) | MP:0008567 |
|  |  | [decreased survivor rate](http://www.informatics.jax.org/searches/Phat.cgi?id=MP:0008770) | MP:0008770 |
|  |  | [abnormal blood vessel endothelium morphology](http://www.informatics.jax.org/searches/Phat.cgi?id=MP:0009489) | MP:0009489 |
|  |  | [abnormal synapse morphology](http://www.informatics.jax.org/searches/Phat.cgi?id=MP:0009538) | MP:0009538 |
|  |  | [abnormal urine homeostasis](http://www.informatics.jax.org/searches/Phat.cgi?id=MP:0009643) | MP:0009643 |
|  |  | [increased sensitivity to induced morbidity/mortality](http://www.informatics.jax.org/searches/Phat.cgi?id=MP:0009763) | MP:0009763 |
|  |  | [decreased sensitivity to induced morbidity/mortality](http://www.informatics.jax.org/searches/Phat.cgi?id=MP:0009764) | MP:0009764 |
|  |  | [increased prostaglandin level](http://www.informatics.jax.org/searches/Phat.cgi?id=MP:0009814) | MP:0009814 |
|  |  | [abnormal aorta elastic tissue morphology](http://www.informatics.jax.org/searches/Phat.cgi?id=MP:0009862) | MP:0009862 |
|  |  | [abnormal aorta endothelium morphology](http://www.informatics.jax.org/searches/Phat.cgi?id=MP:0009864) | MP:0009864 |
|  |  | [abnormal aorta wall morphology](http://www.informatics.jax.org/searches/Phat.cgi?id=MP:0009866) | MP:0009866 |
|  |  | [abnormal descending thoracic aorta morphology](http://www.informatics.jax.org/searches/Phat.cgi?id=MP:0009868) | MP:0009868 |
|  |  | [abnormal descending aorta morphology](http://www.informatics.jax.org/searches/Phat.cgi?id=MP:0009869) | MP:0009869 |
|  |  | [abnormal abdominal aorta morphology](http://www.informatics.jax.org/searches/Phat.cgi?id=MP:0009870) | MP:0009870 |
|  |  | [skin fibrosis](http://www.informatics.jax.org/searches/Phat.cgi?id=MP:0009932) | MP:0009932 |
|  | MGI:2137814 | [abnormal circulating cholesterol level](http://www.informatics.jax.org/searches/Phat.cgi?id=MP:0000180) | MP:0000180 |
|  |  | [abnormal circulating LDL cholesterol level](http://www.informatics.jax.org/searches/Phat.cgi?id=MP:0000181) | MP:0000181 |
|  |  | [decreased circulating HDL cholesterol level](http://www.informatics.jax.org/searches/Phat.cgi?id=MP:0000186) | MP:0000186 |
|  |  | [abnormal cerebral cortex morphology](http://www.informatics.jax.org/searches/Phat.cgi?id=MP:0000788) | MP:0000788 |
|  |  | [abnormal hippocampus morphology](http://www.informatics.jax.org/searches/Phat.cgi?id=MP:0000807) | MP:0000807 |
|  |  | [abnormal body weight](http://www.informatics.jax.org/searches/Phat.cgi?id=MP:0001259) | MP:0001259 |
|  |  | [increased circulating triglyceride level](http://www.informatics.jax.org/searches/Phat.cgi?id=MP:0001552) | MP:0001552 |
|  |  | [abnormal digestive system physiology](http://www.informatics.jax.org/searches/Phat.cgi?id=MP:0001663) | MP:0001663 |
|  |  | [premature death](http://www.informatics.jax.org/searches/Phat.cgi?id=MP:0002083) | MP:0002083 |
|  |  | [abnormal macrophage morphology](http://www.informatics.jax.org/searches/Phat.cgi?id=MP:0002446) | MP:0002446 |
|  |  | [abnormal macrophage physiology](http://www.informatics.jax.org/searches/Phat.cgi?id=MP:0002451) | MP:0002451 |
|  |  | [abnormal neuron morphology](http://www.informatics.jax.org/searches/Phat.cgi?id=MP:0002882) | MP:0002882 |
|  |  | [aortic elastic tissue lesions](http://www.informatics.jax.org/searches/Phat.cgi?id=MP:0002964) | MP:0002964 |
|  |  | [xanthoma](http://www.informatics.jax.org/searches/Phat.cgi?id=MP:0003692) | MP:0003692 |
|  |  | [increased macrophage derived foam cell number](http://www.informatics.jax.org/searches/Phat.cgi?id=MP:0004778) | MP:0004778 |
|  |  | [uterus atrophy](http://www.informatics.jax.org/searches/Phat.cgi?id=MP:0004894) | MP:0004894 |
|  |  | [increased circulating VLDL cholesterol level](http://www.informatics.jax.org/searches/Phat.cgi?id=MP:0005145) | MP:0005145 |
|  |  | [decreased circulating VLDL cholesterol level](http://www.informatics.jax.org/searches/Phat.cgi?id=MP:0005146) | MP:0005146 |
|  |  | [increased circulating cholesterol level](http://www.informatics.jax.org/searches/Phat.cgi?id=MP:0005178) | MP:0005178 |
|  |  | [decreased circulating cholesterol level](http://www.informatics.jax.org/searches/Phat.cgi?id=MP:0005179) | MP:0005179 |
|  |  | [abnormal cholesterol homeostasis](http://www.informatics.jax.org/searches/Phat.cgi?id=MP:0005278) | MP:0005278 |
|  |  | [increased triglyceride level](http://www.informatics.jax.org/searches/Phat.cgi?id=MP:0005317) | MP:0005317 |
|  |  | [atherosclerotic lesions](http://www.informatics.jax.org/searches/Phat.cgi?id=MP:0005338) | MP:0005338 |
|  |  | [increased susceptibility to atherosclerosis](http://www.informatics.jax.org/searches/Phat.cgi?id=MP:0005339) | MP:0005339 |
|  |  | [decreased susceptibility to atherosclerosis](http://www.informatics.jax.org/searches/Phat.cgi?id=MP:0005341) | MP:0005341 |
|  |  | [impaired macrophage recruitment](http://www.informatics.jax.org/searches/Phat.cgi?id=MP:0005496) | MP:0005496 |
|  | MGI:2152302 | [increased circulating LDL cholesterol level](http://www.informatics.jax.org/searches/Phat.cgi?id=MP:0000182) | MP:0000182 |
|  |  | [increased circulating triglyceride level](http://www.informatics.jax.org/searches/Phat.cgi?id=MP:0001552) | MP:0001552 |
|  |  | [increased circulating HDL cholesterol level](http://www.informatics.jax.org/searches/Phat.cgi?id=MP:0001556) | MP:0001556 |
|  |  | [abnormal lipid homeostasis](http://www.informatics.jax.org/searches/Phat.cgi?id=MP:0002118) | MP:0002118 |
|  |  | [xanthoma](http://www.informatics.jax.org/searches/Phat.cgi?id=MP:0003692) | MP:0003692 |
|  |  | [increased circulating VLDL cholesterol level](http://www.informatics.jax.org/searches/Phat.cgi?id=MP:0005145) | MP:0005145 |
|  |  | [increased circulating cholesterol level](http://www.informatics.jax.org/searches/Phat.cgi?id=MP:0005178) | MP:0005178 |
|  |  | [atherosclerotic lesions](http://www.informatics.jax.org/searches/Phat.cgi?id=MP:0005338) | MP:0005338 |
|  |  | [abnormal circulating protein level](http://www.informatics.jax.org/searches/Phat.cgi?id=MP:0005416) | MP:0005416 |
| [Bdnf](http://www.informatics.jax.org/javawi2/servlet/WIFetch?page=markerDetail&id=MGI:88145) | MGI:1857136 | [small mesencephalic trigeminal nucleus](http://www.informatics.jax.org/searches/Phat.cgi?id=MP:0000907) | MP:0000907 |
|  |  | [decreased sensory neuron number](http://www.informatics.jax.org/searches/Phat.cgi?id=MP:0000966) | MP:0000966 |
|  |  | [abnormal sensory neuron innervation](http://www.informatics.jax.org/searches/Phat.cgi?id=MP:0000968) | MP:0000968 |
|  |  | [small L4 dorsal root ganglion](http://www.informatics.jax.org/searches/Phat.cgi?id=MP:0001021) | MP:0001021 |
|  |  | [abnormal trigeminal nerve morphology](http://www.informatics.jax.org/searches/Phat.cgi?id=MP:0001065) | MP:0001065 |
|  |  | [small geniculate ganglion](http://www.informatics.jax.org/searches/Phat.cgi?id=MP:0001083) | MP:0001083 |
|  |  | [small petrosal ganglion](http://www.informatics.jax.org/searches/Phat.cgi?id=MP:0001085) | MP:0001085 |
|  |  | [small nodose ganglion](http://www.informatics.jax.org/searches/Phat.cgi?id=MP:0001088) | MP:0001088 |
|  |  | [small trigeminal ganglion](http://www.informatics.jax.org/searches/Phat.cgi?id=MP:0001093) | MP:0001093 |
|  |  | [decreased body size](http://www.informatics.jax.org/searches/Phat.cgi?id=MP:0001265) | MP:0001265 |
|  |  | [circling](http://www.informatics.jax.org/searches/Phat.cgi?id=MP:0001394) | MP:0001394 |
|  |  | [hyperactivity](http://www.informatics.jax.org/searches/Phat.cgi?id=MP:0001399) | MP:0001399 |
|  |  | [impaired coordination](http://www.informatics.jax.org/searches/Phat.cgi?id=MP:0001405) | MP:0001405 |
|  |  | [head bobbing](http://www.informatics.jax.org/searches/Phat.cgi?id=MP:0001410) | MP:0001410 |
|  |  | [spinning](http://www.informatics.jax.org/searches/Phat.cgi?id=MP:0001411) | MP:0001411 |
|  |  | [impaired limb coordination](http://www.informatics.jax.org/searches/Phat.cgi?id=MP:0001524) | MP:0001524 |
|  |  | [impaired balance](http://www.informatics.jax.org/searches/Phat.cgi?id=MP:0001525) | MP:0001525 |
|  |  | [postnatal growth retardation](http://www.informatics.jax.org/searches/Phat.cgi?id=MP:0001732) | MP:0001732 |
|  |  | [deafness](http://www.informatics.jax.org/searches/Phat.cgi?id=MP:0001967) | MP:0001967 |
|  |  | [postnatal lethality](http://www.informatics.jax.org/searches/Phat.cgi?id=MP:0002082) | MP:0002082 |
|  |  | [no abnormal phenotype detected](http://www.informatics.jax.org/searches/Phat.cgi?id=MP:0002169) | MP:0002169 |
|  |  | [abnormal innervation](http://www.informatics.jax.org/searches/Phat.cgi?id=MP:0002184) | MP:0002184 |
|  |  | [abnormal cochlear ganglion morphology](http://www.informatics.jax.org/searches/Phat.cgi?id=MP:0002855) | MP:0002855 |
|  |  | [abnormal vestibular ganglion morphology](http://www.informatics.jax.org/searches/Phat.cgi?id=MP:0002856) | MP:0002856 |
|  |  | [abnormal neuron morphology](http://www.informatics.jax.org/searches/Phat.cgi?id=MP:0002882) | MP:0002882 |
|  |  | [abnormal neuron apoptosis](http://www.informatics.jax.org/searches/Phat.cgi?id=MP:0003202) | MP:0003202 |
|  |  | [increased neuron apoptosis](http://www.informatics.jax.org/searches/Phat.cgi?id=MP:0003203) | MP:0003203 |
|  |  | [small vestibular ganglion](http://www.informatics.jax.org/searches/Phat.cgi?id=MP:0003987) | MP:0003987 |
|  |  | [abnormal crista ampullaris morphology](http://www.informatics.jax.org/searches/Phat.cgi?id=MP:0004249) | MP:0004249 |
|  |  | [abnormal type I vestibular cell](http://www.informatics.jax.org/searches/Phat.cgi?id=MP:0004428) | MP:0004428 |
|  |  | [abnormal vestibular nerve morphology](http://www.informatics.jax.org/searches/Phat.cgi?id=MP:0004718) | MP:0004718 |
|  |  | [head tilt](http://www.informatics.jax.org/searches/Phat.cgi?id=MP:0005191) | MP:0005191 |
|  |  | [abnormal fungiform papillae](http://www.informatics.jax.org/searches/Phat.cgi?id=MP:0006257) | MP:0006257 |
|  |  | [abnormal gustatory papillae taste buds](http://www.informatics.jax.org/searches/Phat.cgi?id=MP:0006260) | MP:0006260 |
|  |  | [increased neuron number](http://www.informatics.jax.org/searches/Phat.cgi?id=MP:0008947) | MP:0008947 |
|  | MGI:1891516 | [abnormal hair cycle](http://www.informatics.jax.org/searches/Phat.cgi?id=MP:0000427) | MP:0000427 |
|  |  | [abnormal intestine morphology](http://www.informatics.jax.org/searches/Phat.cgi?id=MP:0000477) | MP:0000477 |
|  |  | [abnormal colon morphology](http://www.informatics.jax.org/searches/Phat.cgi?id=MP:0000495) | MP:0000495 |
|  |  | [small dorsal root ganglion](http://www.informatics.jax.org/searches/Phat.cgi?id=MP:0000964) | MP:0000964 |
|  |  | [decreased sensory neuron number](http://www.informatics.jax.org/searches/Phat.cgi?id=MP:0000966) | MP:0000966 |
|  |  | [abnormal sympathetic system morphology](http://www.informatics.jax.org/searches/Phat.cgi?id=MP:0001007) | MP:0001007 |
|  |  | [abnormal adrenergic neuron morphology](http://www.informatics.jax.org/searches/Phat.cgi?id=MP:0001026) | MP:0001026 |
|  |  | [decreased body weight](http://www.informatics.jax.org/searches/Phat.cgi?id=MP:0001262) | MP:0001262 |
|  |  | [reduced long term potentiation](http://www.informatics.jax.org/searches/Phat.cgi?id=MP:0001473) | MP:0001473 |
|  |  | [impaired limb coordination](http://www.informatics.jax.org/searches/Phat.cgi?id=MP:0001524) | MP:0001524 |
|  |  | [impaired balance](http://www.informatics.jax.org/searches/Phat.cgi?id=MP:0001525) | MP:0001525 |
|  |  | [postnatal growth retardation](http://www.informatics.jax.org/searches/Phat.cgi?id=MP:0001732) | MP:0001732 |
|  |  | [impaired synaptic plasticity](http://www.informatics.jax.org/searches/Phat.cgi?id=MP:0001900) | MP:0001900 |
|  |  | [postnatal lethality](http://www.informatics.jax.org/searches/Phat.cgi?id=MP:0002082) | MP:0002082 |
|  |  | [abnormal innervation](http://www.informatics.jax.org/searches/Phat.cgi?id=MP:0002184) | MP:0002184 |
|  |  | [abnormal keratinocyte morphology](http://www.informatics.jax.org/searches/Phat.cgi?id=MP:0002655) | MP:0002655 |
|  |  | [abnormal excitatory postsynaptic potential](http://www.informatics.jax.org/searches/Phat.cgi?id=MP:0002912) | MP:0002912 |
|  |  | [abnormal cochlear OHC afferent innervation](http://www.informatics.jax.org/searches/Phat.cgi?id=MP:0004747) | MP:0004747 |
|  | MGI:1929986 | [abnormal dorsal root ganglion morphology](http://www.informatics.jax.org/searches/Phat.cgi?id=MP:0000961) | MP:0000961 |
|  |  | [small dorsal root ganglion](http://www.informatics.jax.org/searches/Phat.cgi?id=MP:0000964) | MP:0000964 |
|  |  | [decreased sensory neuron number](http://www.informatics.jax.org/searches/Phat.cgi?id=MP:0000966) | MP:0000966 |
|  |  | [abnormal sensory neuron innervation](http://www.informatics.jax.org/searches/Phat.cgi?id=MP:0000968) | MP:0000968 |
|  |  | [small L4 dorsal root ganglion](http://www.informatics.jax.org/searches/Phat.cgi?id=MP:0001021) | MP:0001021 |
|  |  | [small geniculate ganglion](http://www.informatics.jax.org/searches/Phat.cgi?id=MP:0001083) | MP:0001083 |
|  |  | [small petrosal ganglion](http://www.informatics.jax.org/searches/Phat.cgi?id=MP:0001085) | MP:0001085 |
|  |  | [small nodose ganglion](http://www.informatics.jax.org/searches/Phat.cgi?id=MP:0001088) | MP:0001088 |
|  |  | [small trigeminal ganglion](http://www.informatics.jax.org/searches/Phat.cgi?id=MP:0001093) | MP:0001093 |
|  |  | [increased body weight](http://www.informatics.jax.org/searches/Phat.cgi?id=MP:0001260) | MP:0001260 |
|  |  | [increased aggression towards males](http://www.informatics.jax.org/searches/Phat.cgi?id=MP:0001354) | MP:0001354 |
|  |  | [increased anxiety-related response](http://www.informatics.jax.org/searches/Phat.cgi?id=MP:0001363) | MP:0001363 |
|  |  | [decreased exploration in new environment](http://www.informatics.jax.org/searches/Phat.cgi?id=MP:0001417) | MP:0001417 |
|  |  | [polyphagia](http://www.informatics.jax.org/searches/Phat.cgi?id=MP:0001433) | MP:0001433 |
|  |  | [neonatal lethality](http://www.informatics.jax.org/searches/Phat.cgi?id=MP:0002058) | MP:0002058 |
|  |  | [increased circulating insulin level](http://www.informatics.jax.org/searches/Phat.cgi?id=MP:0002079) | MP:0002079 |
|  |  | [postnatal lethality](http://www.informatics.jax.org/searches/Phat.cgi?id=MP:0002082) | MP:0002082 |
|  |  | [abnormal CNS synaptic transmission](http://www.informatics.jax.org/searches/Phat.cgi?id=MP:0002206) | MP:0002206 |
|  |  | [abnormal somatic nervous system morphology](http://www.informatics.jax.org/searches/Phat.cgi?id=MP:0002752) | MP:0002752 |
|  |  | [decreased vertical activity](http://www.informatics.jax.org/searches/Phat.cgi?id=MP:0002757) | MP:0002757 |
|  |  | [abnormal vestibular ganglion morphology](http://www.informatics.jax.org/searches/Phat.cgi?id=MP:0002856) | MP:0002856 |
|  |  | [increased neuron apoptosis](http://www.informatics.jax.org/searches/Phat.cgi?id=MP:0003203) | MP:0003203 |
|  |  | [increased susceptibility to age related obesity](http://www.informatics.jax.org/searches/Phat.cgi?id=MP:0003212) | MP:0003212 |
|  |  | [abnormal locomotor activation](http://www.informatics.jax.org/searches/Phat.cgi?id=MP:0003313) | MP:0003313 |
|  |  | [abnormal vestibulocochlear ganglion morphology](http://www.informatics.jax.org/searches/Phat.cgi?id=MP:0003703) | MP:0003703 |
|  |  | [abnormal serotonergic neuron morphology](http://www.informatics.jax.org/searches/Phat.cgi?id=MP:0003877) | MP:0003877 |
|  |  | [abnormal barrel cortex morphology](http://www.informatics.jax.org/searches/Phat.cgi?id=MP:0003989) | MP:0003989 |
|  |  | [abnormal serotonin concentration](http://www.informatics.jax.org/searches/Phat.cgi?id=MP:0005322) | MP:0005322 |
|  |  | [weight gain](http://www.informatics.jax.org/searches/Phat.cgi?id=MP:0005456) | MP:0005456 |
|  |  | [small hippocampus](http://www.informatics.jax.org/searches/Phat.cgi?id=MP:0008283) | MP:0008283 |
|  |  | [abnormal neuron differentiation](http://www.informatics.jax.org/searches/Phat.cgi?id=MP:0009937) | MP:0009937 |
|  | MGI:1930038 | [decreased brain size](http://www.informatics.jax.org/searches/Phat.cgi?id=MP:0000774) | MP:0000774 |
|  |  | [decreased sensory neuron number](http://www.informatics.jax.org/searches/Phat.cgi?id=MP:0000966) | MP:0000966 |
|  |  | [abnormal sensory neuron innervation](http://www.informatics.jax.org/searches/Phat.cgi?id=MP:0000968) | MP:0000968 |
|  |  | [small petrosal ganglion](http://www.informatics.jax.org/searches/Phat.cgi?id=MP:0001085) | MP:0001085 |
|  |  | [small nodose ganglion](http://www.informatics.jax.org/searches/Phat.cgi?id=MP:0001088) | MP:0001088 |
|  |  | [small trigeminal ganglion](http://www.informatics.jax.org/searches/Phat.cgi?id=MP:0001093) | MP:0001093 |
|  |  | [hyperactivity](http://www.informatics.jax.org/searches/Phat.cgi?id=MP:0001399) | MP:0001399 |
|  |  | [abnormal motor coordination/ balance](http://www.informatics.jax.org/searches/Phat.cgi?id=MP:0001516) | MP:0001516 |
|  |  | [impaired righting response](http://www.informatics.jax.org/searches/Phat.cgi?id=MP:0001523) | MP:0001523 |
|  |  | [postnatal growth retardation](http://www.informatics.jax.org/searches/Phat.cgi?id=MP:0001732) | MP:0001732 |
|  |  | [postnatal lethality](http://www.informatics.jax.org/searches/Phat.cgi?id=MP:0002082) | MP:0002082 |
|  |  | [small vestibular ganglion](http://www.informatics.jax.org/searches/Phat.cgi?id=MP:0003987) | MP:0003987 |
|  |  | [vestibular ganglion degeneration](http://www.informatics.jax.org/searches/Phat.cgi?id=MP:0004298) | MP:0004298 |
|  |  | [abnormal cochlear OHC afferent innervation](http://www.informatics.jax.org/searches/Phat.cgi?id=MP:0004747) | MP:0004747 |
|  | MGI:1930110 | [shortened head](http://www.informatics.jax.org/searches/Phat.cgi?id=MP:0000435) | MP:0000435 |
|  |  | [abnormal cerebral cortex morphology](http://www.informatics.jax.org/searches/Phat.cgi?id=MP:0000788) | MP:0000788 |
|  |  | [abnormal visual cortex morphology](http://www.informatics.jax.org/searches/Phat.cgi?id=MP:0000805) | MP:0000805 |
|  |  | [abnormal hippocampus morphology](http://www.informatics.jax.org/searches/Phat.cgi?id=MP:0000807) | MP:0000807 |
|  |  | [abnormal substantia nigra morphology](http://www.informatics.jax.org/searches/Phat.cgi?id=MP:0000836) | MP:0000836 |
|  |  | [abnormal cerebellum external granule cell layer morphology](http://www.informatics.jax.org/searches/Phat.cgi?id=MP:0000872) | MP:0000872 |
|  |  | [small mesencephalic trigeminal nucleus](http://www.informatics.jax.org/searches/Phat.cgi?id=MP:0000907) | MP:0000907 |
|  |  | [abnormal somatic sensory system morphology](http://www.informatics.jax.org/searches/Phat.cgi?id=MP:0000959) | MP:0000959 |
|  |  | [abnormal sensory ganglion morphology](http://www.informatics.jax.org/searches/Phat.cgi?id=MP:0000960) | MP:0000960 |
|  |  | [small dorsal root ganglion](http://www.informatics.jax.org/searches/Phat.cgi?id=MP:0000964) | MP:0000964 |
|  |  | [decreased sensory neuron number](http://www.informatics.jax.org/searches/Phat.cgi?id=MP:0000966) | MP:0000966 |
|  |  | [abnormal cranial ganglia morphology](http://www.informatics.jax.org/searches/Phat.cgi?id=MP:0001081) | MP:0001081 |
|  |  | [small geniculate ganglion](http://www.informatics.jax.org/searches/Phat.cgi?id=MP:0001083) | MP:0001083 |
|  |  | [small petrosal ganglion](http://www.informatics.jax.org/searches/Phat.cgi?id=MP:0001085) | MP:0001085 |
|  |  | [small nodose ganglion](http://www.informatics.jax.org/searches/Phat.cgi?id=MP:0001088) | MP:0001088 |
|  |  | [obese](http://www.informatics.jax.org/searches/Phat.cgi?id=MP:0001261) | MP:0001261 |
|  |  | [decreased body weight](http://www.informatics.jax.org/searches/Phat.cgi?id=MP:0001262) | MP:0001262 |
|  |  | [decreased body size](http://www.informatics.jax.org/searches/Phat.cgi?id=MP:0001265) | MP:0001265 |
|  |  | [delayed eyelid opening](http://www.informatics.jax.org/searches/Phat.cgi?id=MP:0001290) | MP:0001290 |
|  |  | [ataxia](http://www.informatics.jax.org/searches/Phat.cgi?id=MP:0001393) | MP:0001393 |
|  |  | [impaired coordination](http://www.informatics.jax.org/searches/Phat.cgi?id=MP:0001405) | MP:0001405 |
|  |  | [spinning](http://www.informatics.jax.org/searches/Phat.cgi?id=MP:0001411) | MP:0001411 |
|  |  | [abnormal nest building behavior](http://www.informatics.jax.org/searches/Phat.cgi?id=MP:0001447) | MP:0001447 |
|  |  | [hunched posture](http://www.informatics.jax.org/searches/Phat.cgi?id=MP:0001505) | MP:0001505 |
|  |  | [limb grasping](http://www.informatics.jax.org/searches/Phat.cgi?id=MP:0001513) | MP:0001513 |
|  |  | [impaired righting response](http://www.informatics.jax.org/searches/Phat.cgi?id=MP:0001523) | MP:0001523 |
|  |  | [reduced fertility](http://www.informatics.jax.org/searches/Phat.cgi?id=MP:0001921) | MP:0001921 |
|  |  | [abnormal motor capabilities/coordination/movement](http://www.informatics.jax.org/searches/Phat.cgi?id=MP:0002066) | MP:0002066 |
|  |  | [postnatal lethality](http://www.informatics.jax.org/searches/Phat.cgi?id=MP:0002082) | MP:0002082 |
|  |  | [premature death](http://www.informatics.jax.org/searches/Phat.cgi?id=MP:0002083) | MP:0002083 |
|  |  | [abnormal vestibular ganglion morphology](http://www.informatics.jax.org/searches/Phat.cgi?id=MP:0002856) | MP:0002856 |
|  |  | [abnormal locomotor activation](http://www.informatics.jax.org/searches/Phat.cgi?id=MP:0003313) | MP:0003313 |
|  |  | [abnormal striatum morphology](http://www.informatics.jax.org/searches/Phat.cgi?id=MP:0004077) | MP:0004077 |
|  |  | [decreased breathing frequency](http://www.informatics.jax.org/searches/Phat.cgi?id=MP:0005574) | MP:0005574 |
|  |  | [increased aggression](http://www.informatics.jax.org/searches/Phat.cgi?id=MP:0005655) | MP:0005655 |
|  |  | [thin cerebral cortex](http://www.informatics.jax.org/searches/Phat.cgi?id=MP:0006254) | MP:0006254 |
|  |  | [abnormal fungiform papillae](http://www.informatics.jax.org/searches/Phat.cgi?id=MP:0006257) | MP:0006257 |
|  |  | [abnormal circumvallate papillae](http://www.informatics.jax.org/searches/Phat.cgi?id=MP:0006258) | MP:0006258 |
|  |  | [abnormal gustatory papillae taste buds](http://www.informatics.jax.org/searches/Phat.cgi?id=MP:0006260) | MP:0006260 |
|  |  | [abnormal medium spiny neuron morphology](http://www.informatics.jax.org/searches/Phat.cgi?id=MP:0008462) | MP:0008462 |
|  |  | [decreased tongue size](http://www.informatics.jax.org/searches/Phat.cgi?id=MP:0009907) | MP:0009907 |
|  |  | [abnormal neuron differentiation](http://www.informatics.jax.org/searches/Phat.cgi?id=MP:0009937) | MP:0009937 |
| [Bmp6](http://www.informatics.jax.org/javawi2/servlet/WIFetch?page=markerDetail&id=MGI:88182) | MGI:2136985 | [abnormal sternum morphology](http://www.informatics.jax.org/searches/Phat.cgi?id=MP:0000157) | MP:0000157 |
|  |  | [abnormal xiphoid process](http://www.informatics.jax.org/searches/Phat.cgi?id=MP:0000159) | MP:0000159 |
|  |  | [postnatal growth retardation](http://www.informatics.jax.org/searches/Phat.cgi?id=MP:0001732) | MP:0001732 |
|  |  | [male infertility](http://www.informatics.jax.org/searches/Phat.cgi?id=MP:0001925) | MP:0001925 |
|  |  | [split xiphoid process](http://www.informatics.jax.org/searches/Phat.cgi?id=MP:0004678) | MP:0004678 |
|  |  | [small xiphoid process](http://www.informatics.jax.org/searches/Phat.cgi?id=MP:0004680) | MP:0004680 |
|  |  | [abnormal sternum ossification](http://www.informatics.jax.org/searches/Phat.cgi?id=MP:0008277) | MP:0008277 |
|  |  | [abnormal sternal manubrium morphology](http://www.informatics.jax.org/searches/Phat.cgi?id=MP:0008785) | MP:0008785 |
| [Cd40](http://www.informatics.jax.org/javawi2/servlet/WIFetch?page=markerDetail&id=MGI:88336) | MGI:1857457 | [decreased neutrophil cell number](http://www.informatics.jax.org/searches/Phat.cgi?id=MP:0000222) | MP:0000222 |
|  |  | [enlarged spleen](http://www.informatics.jax.org/searches/Phat.cgi?id=MP:0000691) | MP:0000691 |
|  |  | [enlarged lymph nodes](http://www.informatics.jax.org/searches/Phat.cgi?id=MP:0000702) | MP:0000702 |
|  |  | [abnormal humoral immune response](http://www.informatics.jax.org/searches/Phat.cgi?id=MP:0001800) | MP:0001800 |
|  |  | [decreased IgA level](http://www.informatics.jax.org/searches/Phat.cgi?id=MP:0001807) | MP:0001807 |
|  |  | [abnormal dendritic cell physiology](http://www.informatics.jax.org/searches/Phat.cgi?id=MP:0002376) | MP:0002376 |
|  |  | [increased susceptibility to bacterial infection](http://www.informatics.jax.org/searches/Phat.cgi?id=MP:0002412) | MP:0002412 |
|  |  | [decreased immunoglobulin level](http://www.informatics.jax.org/searches/Phat.cgi?id=MP:0002460) | MP:0002460 |
|  |  | [decreased IgE level](http://www.informatics.jax.org/searches/Phat.cgi?id=MP:0002492) | MP:0002492 |
|  |  | [glomerulonephritis](http://www.informatics.jax.org/searches/Phat.cgi?id=MP:0002743) | MP:0002743 |
|  |  | [increased anti-double stranded DNA antibody level](http://www.informatics.jax.org/searches/Phat.cgi?id=MP:0004762) | MP:0004762 |
|  |  | [abnormal class switch recombination](http://www.informatics.jax.org/searches/Phat.cgi?id=MP:0004816) | MP:0004816 |
|  |  | [proteinuria](http://www.informatics.jax.org/searches/Phat.cgi?id=MP:0005160) | MP:0005160 |
|  |  | [decreased susceptibility to type II hypersensitivity reaction](http://www.informatics.jax.org/searches/Phat.cgi?id=MP:0005612) | MP:0005612 |
|  |  | [increased marginal zone B cell number](http://www.informatics.jax.org/searches/Phat.cgi?id=MP:0008181) | MP:0008181 |
|  |  | [abnormal B cell activation](http://www.informatics.jax.org/searches/Phat.cgi?id=MP:0008217) | MP:0008217 |
|  |  | [absent spleen germinal center](http://www.informatics.jax.org/searches/Phat.cgi?id=MP:0008474) | MP:0008474 |
|  |  | [decreased IgG1 level](http://www.informatics.jax.org/searches/Phat.cgi?id=MP:0008495) | MP:0008495 |
|  |  | [decreased IgG2a level](http://www.informatics.jax.org/searches/Phat.cgi?id=MP:0008496) | MP:0008496 |
|  |  | [decreased IgG2b level](http://www.informatics.jax.org/searches/Phat.cgi?id=MP:0008497) | MP:0008497 |
|  |  | [decreased IgG3 level](http://www.informatics.jax.org/searches/Phat.cgi?id=MP:0008498) | MP:0008498 |
|  | MGI:2655321 | [abnormal humoral immune response](http://www.informatics.jax.org/searches/Phat.cgi?id=MP:0001800) | MP:0001800 |
|  |  | [decreased IgM level](http://www.informatics.jax.org/searches/Phat.cgi?id=MP:0001806) | MP:0001806 |
|  |  | [decreased IgA level](http://www.informatics.jax.org/searches/Phat.cgi?id=MP:0001807) | MP:0001807 |
|  |  | [decreased B cell number](http://www.informatics.jax.org/searches/Phat.cgi?id=MP:0005017) | MP:0005017 |
|  |  | [decreased B cell proliferation](http://www.informatics.jax.org/searches/Phat.cgi?id=MP:0005093) | MP:0005093 |
|  |  | [decreased mature B cell number](http://www.informatics.jax.org/searches/Phat.cgi?id=MP:0008211) | MP:0008211 |
|  |  | [absent spleen germinal center](http://www.informatics.jax.org/searches/Phat.cgi?id=MP:0008474) | MP:0008474 |
|  |  | [decreased spleen germinal center number](http://www.informatics.jax.org/searches/Phat.cgi?id=MP:0008482) | MP:0008482 |
|  |  | [decreased spleen germinal center size](http://www.informatics.jax.org/searches/Phat.cgi?id=MP:0008484) | MP:0008484 |
|  |  | [decreased IgG1 level](http://www.informatics.jax.org/searches/Phat.cgi?id=MP:0008495) | MP:0008495 |
|  |  | [decreased IgG2a level](http://www.informatics.jax.org/searches/Phat.cgi?id=MP:0008496) | MP:0008496 |
|  |  | [decreased IgG2b level](http://www.informatics.jax.org/searches/Phat.cgi?id=MP:0008497) | MP:0008497 |
|  |  | [decreased IgG3 level](http://www.informatics.jax.org/searches/Phat.cgi?id=MP:0008498) | MP:0008498 |
|  |  | [abnormal lymph node germinal center](http://www.informatics.jax.org/searches/Phat.cgi?id=MP:0008522) | MP:0008522 |
|  |  | [absent lymph node germinal center](http://www.informatics.jax.org/searches/Phat.cgi?id=MP:0008523) | MP:0008523 |
|  | MGI:3707600 | [decreased IgG level](http://www.informatics.jax.org/searches/Phat.cgi?id=MP:0001805) | MP:0001805 |
|  |  | [decreased IgM level](http://www.informatics.jax.org/searches/Phat.cgi?id=MP:0001806) | MP:0001806 |
|  |  | [abnormal immunoglobulin level](http://www.informatics.jax.org/searches/Phat.cgi?id=MP:0002490) | MP:0002490 |
|  |  | [decreased IgE level](http://www.informatics.jax.org/searches/Phat.cgi?id=MP:0002492) | MP:0002492 |
|  |  | [increased IgM level](http://www.informatics.jax.org/searches/Phat.cgi?id=MP:0002494) | MP:0002494 |
|  |  | [abnormal response to transplant](http://www.informatics.jax.org/searches/Phat.cgi?id=MP:0005671) | MP:0005671 |
|  |  | [absent spleen germinal center](http://www.informatics.jax.org/searches/Phat.cgi?id=MP:0008474) | MP:0008474 |
|  |  | [decreased IgG1 level](http://www.informatics.jax.org/searches/Phat.cgi?id=MP:0008495) | MP:0008495 |
|  |  | [decreased IgG2b level](http://www.informatics.jax.org/searches/Phat.cgi?id=MP:0008497) | MP:0008497 |
|  |  | [increased IgG3 level](http://www.informatics.jax.org/searches/Phat.cgi?id=MP:0008502) | MP:0008502 |
| [Cd69](http://www.informatics.jax.org/javawi2/servlet/WIFetch?page=markerDetail&id=MGI:88343) | MGI:2675144 | [decreased susceptibility to induced arthritis](http://www.informatics.jax.org/searches/Phat.cgi?id=MP:0003436) | MP:0003436 |
|  |  | [impaired neutrophil recruitment](http://www.informatics.jax.org/searches/Phat.cgi?id=MP:0008719) | MP:0008719 |
|  |  | [impaired neutrophil migration](http://www.informatics.jax.org/searches/Phat.cgi?id=MP:0008720) | MP:0008720 |
|  | MGI:2180772 | [abnormal humoral immune response](http://www.informatics.jax.org/searches/Phat.cgi?id=MP:0001800) | MP:0001800 |
|  |  | [abnormal B cell differentiation](http://www.informatics.jax.org/searches/Phat.cgi?id=MP:0002144) | MP:0002144 |
|  |  | [increased IgM level](http://www.informatics.jax.org/searches/Phat.cgi?id=MP:0002494) | MP:0002494 |
|  |  | [increased pre-B cell number](http://www.informatics.jax.org/searches/Phat.cgi?id=MP:0003132) | MP:0003132 |
|  |  | [increased immature B cell number](http://www.informatics.jax.org/searches/Phat.cgi?id=MP:0008214) | MP:0008214 |
|  |  | [increased IgG2a level](http://www.informatics.jax.org/searches/Phat.cgi?id=MP:0008500) | MP:0008500 |
| [Ctla4](http://www.informatics.jax.org/javawi2/servlet/WIFetch?page=markerDetail&id=MGI:88556) | MGI:2180668 | [enlarged spleen](http://www.informatics.jax.org/searches/Phat.cgi?id=MP:0000691) | MP:0000691 |
|  |  | [enlarged lymph nodes](http://www.informatics.jax.org/searches/Phat.cgi?id=MP:0000702) | MP:0000702 |
|  |  | [autoimmune response](http://www.informatics.jax.org/searches/Phat.cgi?id=MP:0001844) | MP:0001844 |
|  |  | [myocarditis](http://www.informatics.jax.org/searches/Phat.cgi?id=MP:0001856) | MP:0001856 |
|  |  | [lung inflammation](http://www.informatics.jax.org/searches/Phat.cgi?id=MP:0001861) | MP:0001861 |
|  |  | [vasculitis](http://www.informatics.jax.org/searches/Phat.cgi?id=MP:0001864) | MP:0001864 |
|  |  | [pancreas inflammation](http://www.informatics.jax.org/searches/Phat.cgi?id=MP:0001869) | MP:0001869 |
|  |  | [salivary gland inflammation](http://www.informatics.jax.org/searches/Phat.cgi?id=MP:0001870) | MP:0001870 |
|  |  | [premature death](http://www.informatics.jax.org/searches/Phat.cgi?id=MP:0002083) | MP:0002083 |
|  |  | [abnormal T cell differentiation](http://www.informatics.jax.org/searches/Phat.cgi?id=MP:0002145) | MP:0002145 |
|  |  | [abnormal T cell physiology](http://www.informatics.jax.org/searches/Phat.cgi?id=MP:0002444) | MP:0002444 |
|  |  | [joint inflammation](http://www.informatics.jax.org/searches/Phat.cgi?id=MP:0002933) | MP:0002933 |
|  |  | [abnormal cytokine secretion](http://www.informatics.jax.org/searches/Phat.cgi?id=MP:0003009) | MP:0003009 |
|  |  | [increased length of allograft survival](http://www.informatics.jax.org/searches/Phat.cgi?id=MP:0004751) | MP:0004751 |
|  |  | [abnormal regulatory T cell physiology](http://www.informatics.jax.org/searches/Phat.cgi?id=MP:0004946) | MP:0004946 |
|  |  | [abnormal CD8-positive T cell morphology](http://www.informatics.jax.org/searches/Phat.cgi?id=MP:0005010) | MP:0005010 |
|  |  | [increased T cell number](http://www.informatics.jax.org/searches/Phat.cgi?id=MP:0005015) | MP:0005015 |
|  |  | [increased double-negative T cell number](http://www.informatics.jax.org/searches/Phat.cgi?id=MP:0005090) | MP:0005090 |
|  |  | [decreased double-positive T cell number](http://www.informatics.jax.org/searches/Phat.cgi?id=MP:0005092) | MP:0005092 |
|  |  | [decreased T cell proliferation](http://www.informatics.jax.org/searches/Phat.cgi?id=MP:0005095) | MP:0005095 |
|  |  | [increased T cell proliferation](http://www.informatics.jax.org/searches/Phat.cgi?id=MP:0005348) | MP:0005348 |
|  |  | [increased susceptibility to autoimmune disorder](http://www.informatics.jax.org/searches/Phat.cgi?id=MP:0005350) | MP:0005350 |
|  |  | [abnormal T cell morphology](http://www.informatics.jax.org/searches/Phat.cgi?id=MP:0008037) | MP:0008037 |
|  |  | [decreased CD4-positive T cell number](http://www.informatics.jax.org/searches/Phat.cgi?id=MP:0008075) | MP:0008075 |
|  |  | [abnormal interleukin secretion](http://www.informatics.jax.org/searches/Phat.cgi?id=MP:0008568) | MP:0008568 |
|  |  | [abnormal interferon level](http://www.informatics.jax.org/searches/Phat.cgi?id=MP:0008750) | MP:0008750 |
|  |  | [abnormal splenic cell ratio](http://www.informatics.jax.org/searches/Phat.cgi?id=MP:0008826) | MP:0008826 |
|  |  | [abnormal lymph node cell ratio](http://www.informatics.jax.org/searches/Phat.cgi?id=MP:0008828) | MP:0008828 |
|  | MGI:2180669 | [premature death](http://www.informatics.jax.org/searches/Phat.cgi?id=MP:0002083) | MP:0002083 |
|  |  | [increased interferon-gamma secretion](http://www.informatics.jax.org/searches/Phat.cgi?id=MP:0008566) | MP:0008566 |
|  |  | [increased interleukin-17 secretion](http://www.informatics.jax.org/searches/Phat.cgi?id=MP:0008681) | MP:0008681 |
|  |  | [increased interleukin-2 secretion](http://www.informatics.jax.org/searches/Phat.cgi?id=MP:0008687) | MP:0008687 |
|  |  | [increased interleukin-4 secretion](http://www.informatics.jax.org/searches/Phat.cgi?id=MP:0008699) | MP:0008699 |
|  | MGI:2180670 | [abnormal thymus morphology](http://www.informatics.jax.org/searches/Phat.cgi?id=MP:0000703) | MP:0000703 |
|  |  | [premature death](http://www.informatics.jax.org/searches/Phat.cgi?id=MP:0002083) | MP:0002083 |
|  |  | [decreased T cell number](http://www.informatics.jax.org/searches/Phat.cgi?id=MP:0005018) | MP:0005018 |
|  |  | [increased double-negative T cell number](http://www.informatics.jax.org/searches/Phat.cgi?id=MP:0005090) | MP:0005090 |
|  |  | [decreased double-positive T cell number](http://www.informatics.jax.org/searches/Phat.cgi?id=MP:0005092) | MP:0005092 |
|  |  | [lymph node hypoplasia](http://www.informatics.jax.org/searches/Phat.cgi?id=MP:0008101) | MP:0008101 |
| [Sh2b3](http://www.informatics.jax.org/javawi2/servlet/WIFetch?page=markerDetail&id=MGI:893598) | MGI:2180088 | [abnormal hematopoietic stem cell morphology](http://www.informatics.jax.org/searches/Phat.cgi?id=MP:0004808) | MP:0004808 |
|  | MGI:2383957 | [abnormal leukocyte cell number](http://www.informatics.jax.org/searches/Phat.cgi?id=MP:0000217) | MP:0000217 |
|  |  | [extramedullary hematopoiesis](http://www.informatics.jax.org/searches/Phat.cgi?id=MP:0000240) | MP:0000240 |
|  |  | [abnormal erythropoiesis](http://www.informatics.jax.org/searches/Phat.cgi?id=MP:0000245) | MP:0000245 |
|  |  | [increased bone marrow cell number](http://www.informatics.jax.org/searches/Phat.cgi?id=MP:0000321) | MP:0000321 |
|  |  | [increased cell proliferation](http://www.informatics.jax.org/searches/Phat.cgi?id=MP:0000351) | MP:0000351 |
|  |  | [enlarged spleen](http://www.informatics.jax.org/searches/Phat.cgi?id=MP:0000691) | MP:0000691 |
|  |  | [spleen hyperplasia](http://www.informatics.jax.org/searches/Phat.cgi?id=MP:0000693) | MP:0000693 |
|  |  | [enlarged lymph nodes](http://www.informatics.jax.org/searches/Phat.cgi?id=MP:0000702) | MP:0000702 |
|  |  | [anemia](http://www.informatics.jax.org/searches/Phat.cgi?id=MP:0001577) | MP:0001577 |
|  |  | [abnormal megakaryocyte morphology](http://www.informatics.jax.org/searches/Phat.cgi?id=MP:0002417) | MP:0002417 |
|  |  | [fibrosis](http://www.informatics.jax.org/searches/Phat.cgi?id=MP:0003045) | MP:0003045 |
|  |  | [increased B cell number](http://www.informatics.jax.org/searches/Phat.cgi?id=MP:0005014) | MP:0005014 |
|  |  | [increased platelet cell number](http://www.informatics.jax.org/searches/Phat.cgi?id=MP:0005505) | MP:0005505 |
|  |  | [abnormal granulocyte differentiation](http://www.informatics.jax.org/searches/Phat.cgi?id=MP:0008111) | MP:0008111 |
|  |  | [abnormal monocyte differentiation](http://www.informatics.jax.org/searches/Phat.cgi?id=MP:0008112) | MP:0008112 |
|  |  | [increased megakaryocyte cell number](http://www.informatics.jax.org/searches/Phat.cgi?id=MP:0008254) | MP:0008254 |
| [Bach2](http://www.informatics.jax.org/javawi2/servlet/WIFetch?page=markerDetail&id=MGI:894679) | MGI:3047541 | [spleen hypoplasia](http://www.informatics.jax.org/searches/Phat.cgi?id=MP:0000694) | MP:0000694 |
|  |  | [decreased IgG level](http://www.informatics.jax.org/searches/Phat.cgi?id=MP:0001805) | MP:0001805 |
|  |  | [decreased IgA level](http://www.informatics.jax.org/searches/Phat.cgi?id=MP:0001807) | MP:0001807 |
|  |  | [abnormal B cell differentiation](http://www.informatics.jax.org/searches/Phat.cgi?id=MP:0002144) | MP:0002144 |
|  |  | [abnormal B cell physiology](http://www.informatics.jax.org/searches/Phat.cgi?id=MP:0002459) | MP:0002459 |
|  |  | [increased IgM level](http://www.informatics.jax.org/searches/Phat.cgi?id=MP:0002494) | MP:0002494 |
|  |  | [abnormal somatic hypermutation frequency](http://www.informatics.jax.org/searches/Phat.cgi?id=MP:0004815) | MP:0004815 |
|  |  | [abnormal class switch recombination](http://www.informatics.jax.org/searches/Phat.cgi?id=MP:0004816) | MP:0004816 |
|  |  | [decreased B cell number](http://www.informatics.jax.org/searches/Phat.cgi?id=MP:0005017) | MP:0005017 |
|  |  | [absent germinal center B cells](http://www.informatics.jax.org/searches/Phat.cgi?id=MP:0008179) | MP:0008179 |
|  |  | [decreased mature B cell number](http://www.informatics.jax.org/searches/Phat.cgi?id=MP:0008211) | MP:0008211 |
|  |  | [decreased IgG1 level](http://www.informatics.jax.org/searches/Phat.cgi?id=MP:0008495) | MP:0008495 |
|  |  | [decreased IgG2a level](http://www.informatics.jax.org/searches/Phat.cgi?id=MP:0008496) | MP:0008496 |
|  |  | [decreased IgG2b level](http://www.informatics.jax.org/searches/Phat.cgi?id=MP:0008497) | MP:0008497 |
|  |  | [decreased IgG3 level](http://www.informatics.jax.org/searches/Phat.cgi?id=MP:0008498) | MP:0008498 |
| [Erbb3](http://www.informatics.jax.org/javawi2/servlet/WIFetch?page=markerDetail&id=MGI:95411) | MGI:1929072 | [motor neuron degeneration](http://www.informatics.jax.org/searches/Phat.cgi?id=MP:0000938) | MP:0000938 |
|  |  | [abnormal dorsal root ganglion morphology](http://www.informatics.jax.org/searches/Phat.cgi?id=MP:0000961) | MP:0000961 |
|  |  | [small dorsal root ganglion](http://www.informatics.jax.org/searches/Phat.cgi?id=MP:0000964) | MP:0000964 |
|  |  | [abnormal cranial nerve morphology](http://www.informatics.jax.org/searches/Phat.cgi?id=MP:0001056) | MP:0001056 |
|  |  | [abnormal cranial ganglia morphology](http://www.informatics.jax.org/searches/Phat.cgi?id=MP:0001081) | MP:0001081 |
|  |  | [abnormal PNS glial cell morphology](http://www.informatics.jax.org/searches/Phat.cgi?id=MP:0001105) | MP:0001105 |
|  |  | [absent Schwann cell precursors](http://www.informatics.jax.org/searches/Phat.cgi?id=MP:0001109) | MP:0001109 |
|  |  | [decreased body weight](http://www.informatics.jax.org/searches/Phat.cgi?id=MP:0001262) | MP:0001262 |
|  |  | [no spontaneous movement](http://www.informatics.jax.org/searches/Phat.cgi?id=MP:0001404) | MP:0001404 |
|  |  | [unresponsive to tactile stimuli](http://www.informatics.jax.org/searches/Phat.cgi?id=MP:0001491) | MP:0001491 |
|  |  | [cyanosis](http://www.informatics.jax.org/searches/Phat.cgi?id=MP:0001575) | MP:0001575 |
|  |  | [respiratory failure](http://www.informatics.jax.org/searches/Phat.cgi?id=MP:0001953) | MP:0001953 |
|  |  | [perinatal lethality](http://www.informatics.jax.org/searches/Phat.cgi?id=MP:0002081) | MP:0002081 |
|  |  | [increased neuron apoptosis](http://www.informatics.jax.org/searches/Phat.cgi?id=MP:0003203) | MP:0003203 |
|  |  | [neuron degeneration](http://www.informatics.jax.org/searches/Phat.cgi?id=MP:0003224) | MP:0003224 |
|  |  | [embryonic lethality during organogenesis](http://www.informatics.jax.org/searches/Phat.cgi?id=MP:0006207) | MP:0006207 |
|  | MGI:1929598 | [abnormal adrenal gland morphology](http://www.informatics.jax.org/searches/Phat.cgi?id=MP:0000639) | MP:0000639 |
|  |  | [absent adrenergic chromaffin cells](http://www.informatics.jax.org/searches/Phat.cgi?id=MP:0000645) | MP:0000645 |
|  |  | [abnormal myelination](http://www.informatics.jax.org/searches/Phat.cgi?id=MP:0000920) | MP:0000920 |
|  |  | [motor neuron degeneration](http://www.informatics.jax.org/searches/Phat.cgi?id=MP:0000938) | MP:0000938 |
|  |  | [abnormal dorsal root ganglion morphology](http://www.informatics.jax.org/searches/Phat.cgi?id=MP:0000961) | MP:0000961 |
|  |  | [small dorsal root ganglion](http://www.informatics.jax.org/searches/Phat.cgi?id=MP:0000964) | MP:0000964 |
|  |  | [abnormal sympathetic system morphology](http://www.informatics.jax.org/searches/Phat.cgi?id=MP:0001007) | MP:0001007 |
|  |  | [abnormal sympathetic ganglion morphology](http://www.informatics.jax.org/searches/Phat.cgi?id=MP:0001008) | MP:0001008 |
|  |  | [abnormal superior cervical ganglion morphology](http://www.informatics.jax.org/searches/Phat.cgi?id=MP:0001011) | MP:0001011 |
|  |  | [small superior cervical ganglion](http://www.informatics.jax.org/searches/Phat.cgi?id=MP:0001015) | MP:0001015 |
|  |  | [abnormal cranial nerve morphology](http://www.informatics.jax.org/searches/Phat.cgi?id=MP:0001056) | MP:0001056 |
|  |  | [abnormal cranial ganglia morphology](http://www.informatics.jax.org/searches/Phat.cgi?id=MP:0001081) | MP:0001081 |
|  |  | [abnormal PNS glial cell morphology](http://www.informatics.jax.org/searches/Phat.cgi?id=MP:0001105) | MP:0001105 |
|  |  | [absent Schwann cell precursors](http://www.informatics.jax.org/searches/Phat.cgi?id=MP:0001109) | MP:0001109 |
|  |  | [decreased body weight](http://www.informatics.jax.org/searches/Phat.cgi?id=MP:0001262) | MP:0001262 |
|  |  | [no spontaneous movement](http://www.informatics.jax.org/searches/Phat.cgi?id=MP:0001404) | MP:0001404 |
|  |  | [unresponsive to tactile stimuli](http://www.informatics.jax.org/searches/Phat.cgi?id=MP:0001491) | MP:0001491 |
|  |  | [cyanosis](http://www.informatics.jax.org/searches/Phat.cgi?id=MP:0001575) | MP:0001575 |
|  |  | [respiratory failure](http://www.informatics.jax.org/searches/Phat.cgi?id=MP:0001953) | MP:0001953 |
|  |  | [perinatal lethality](http://www.informatics.jax.org/searches/Phat.cgi?id=MP:0002081) | MP:0002081 |
|  |  | [postnatal lethality](http://www.informatics.jax.org/searches/Phat.cgi?id=MP:0002082) | MP:0002082 |
|  |  | [abnormal neural crest cell migration](http://www.informatics.jax.org/searches/Phat.cgi?id=MP:0002950) | MP:0002950 |
|  |  | [increased neuron apoptosis](http://www.informatics.jax.org/searches/Phat.cgi?id=MP:0003203) | MP:0003203 |
|  |  | [neuron degeneration](http://www.informatics.jax.org/searches/Phat.cgi?id=MP:0003224) | MP:0003224 |
|  |  | [abnormal noradrenaline level](http://www.informatics.jax.org/searches/Phat.cgi?id=MP:0003964) | MP:0003964 |
|  |  | [abnormal nerve conduction](http://www.informatics.jax.org/searches/Phat.cgi?id=MP:0005403) | MP:0005403 |
|  |  | [embryonic lethality during organogenesis](http://www.informatics.jax.org/searches/Phat.cgi?id=MP:0006207) | MP:0006207 |
|  |  | [abnormal prevertebral ganglion morphology](http://www.informatics.jax.org/searches/Phat.cgi?id=MP:0008316) | MP:0008316 |
|  |  | [abnormal paravertebral ganglion morphology](http://www.informatics.jax.org/searches/Phat.cgi?id=MP:0008317) | MP:0008317 |
|  | MGI:3513098 | [postnatal lethality](http://www.informatics.jax.org/searches/Phat.cgi?id=MP:0002082) | MP:0002082 |
|  |  | [no abnormal phenotype detected](http://www.informatics.jax.org/searches/Phat.cgi?id=MP:0002169) | MP:0002169 |
|  |  | [increased resistance to tumor development](http://www.informatics.jax.org/searches/Phat.cgi?id=MP:0005514) | MP:0005514 |
|  |  | [lethality throughout fetal growth and development](http://www.informatics.jax.org/searches/Phat.cgi?id=MP:0006208) | MP:0006208 |
|  |  | [intestinal polyps](http://www.informatics.jax.org/searches/Phat.cgi?id=MP:0008011) | MP:0008011 |
|  |  | [increased susceptibility to induced colitis](http://www.informatics.jax.org/searches/Phat.cgi?id=MP:0008537) | MP:0008537 |
|  | MGI:1928828 | [abnormal heart morphology](http://www.informatics.jax.org/searches/Phat.cgi?id=MP:0000266) | MP:0000266 |
|  |  | [abnormal heart development](http://www.informatics.jax.org/searches/Phat.cgi?id=MP:0000267) | MP:0000267 |
|  |  | [abnormal endocardial cushion morphology](http://www.informatics.jax.org/searches/Phat.cgi?id=MP:0000297) | MP:0000297 |
|  |  | [thin endocardial cushion](http://www.informatics.jax.org/searches/Phat.cgi?id=MP:0000300) | MP:0000300 |
|  |  | [abnormal stomach epithelium morphology](http://www.informatics.jax.org/searches/Phat.cgi?id=MP:0000471) | MP:0000471 |
|  |  | [absent adrenergic chromaffin cells](http://www.informatics.jax.org/searches/Phat.cgi?id=MP:0000645) | MP:0000645 |
|  |  | [abnormal forebrain morphology](http://www.informatics.jax.org/searches/Phat.cgi?id=MP:0000783) | MP:0000783 |
|  |  | [abnormal embryonic neuroepithelial layer differentiation](http://www.informatics.jax.org/searches/Phat.cgi?id=MP:0000786) | MP:0000786 |
|  |  | [abnormal choroid plexus morphology](http://www.informatics.jax.org/searches/Phat.cgi?id=MP:0000820) | MP:0000820 |
|  |  | [abnormal brain ventricle morphology](http://www.informatics.jax.org/searches/Phat.cgi?id=MP:0000822) | MP:0000822 |
|  |  | [abnormal hindbrain morphology](http://www.informatics.jax.org/searches/Phat.cgi?id=MP:0000841) | MP:0000841 |
|  |  | [abnormal pontine flexure](http://www.informatics.jax.org/searches/Phat.cgi?id=MP:0000844) | MP:0000844 |
|  |  | [cerebellum hypoplasia](http://www.informatics.jax.org/searches/Phat.cgi?id=MP:0000851) | MP:0000851 |
|  |  | [abnormal cerebellar plate morphology](http://www.informatics.jax.org/searches/Phat.cgi?id=MP:0000856) | MP:0000856 |
|  |  | [absent cerebellum vermis](http://www.informatics.jax.org/searches/Phat.cgi?id=MP:0000865) | MP:0000865 |
|  |  | [decreased Purkinje cell number](http://www.informatics.jax.org/searches/Phat.cgi?id=MP:0000880) | MP:0000880 |
|  |  | [abnormal midbrain morphology](http://www.informatics.jax.org/searches/Phat.cgi?id=MP:0000897) | MP:0000897 |
|  |  | [abnormal enteric ganglia morphology](http://www.informatics.jax.org/searches/Phat.cgi?id=MP:0001045) | MP:0001045 |
|  |  | [abnormal cranial ganglia morphology](http://www.informatics.jax.org/searches/Phat.cgi?id=MP:0001081) | MP:0001081 |
|  |  | [small petrosal ganglion](http://www.informatics.jax.org/searches/Phat.cgi?id=MP:0001085) | MP:0001085 |
|  |  | [small nodose ganglion](http://www.informatics.jax.org/searches/Phat.cgi?id=MP:0001088) | MP:0001088 |
|  |  | [abnormal trigeminal ganglion morphology](http://www.informatics.jax.org/searches/Phat.cgi?id=MP:0001092) | MP:0001092 |
|  |  | [decreased Schwann cell number](http://www.informatics.jax.org/searches/Phat.cgi?id=MP:0001107) | MP:0001107 |
|  |  | [poor circulation](http://www.informatics.jax.org/searches/Phat.cgi?id=MP:0001633) | MP:0001633 |
|  |  | [abnormal pancreas morphology](http://www.informatics.jax.org/searches/Phat.cgi?id=MP:0001944) | MP:0001944 |
|  |  | [abnormal brain morphology](http://www.informatics.jax.org/searches/Phat.cgi?id=MP:0002152) | MP:0002152 |
|  |  | [abnormal myocardial trabeculae morphology](http://www.informatics.jax.org/searches/Phat.cgi?id=MP:0002189) | MP:0002189 |
|  |  | [abnormal brain ventricle/choroid plexus morphology](http://www.informatics.jax.org/searches/Phat.cgi?id=MP:0002200) | MP:0002200 |
|  |  | [abnormal atrioventricular valve morphology](http://www.informatics.jax.org/searches/Phat.cgi?id=MP:0002745) | MP:0002745 |
|  |  | [abnormal glial cell morphology](http://www.informatics.jax.org/searches/Phat.cgi?id=MP:0003634) | MP:0003634 |
|  |  | [abnormal ventral spinal root morphology](http://www.informatics.jax.org/searches/Phat.cgi?id=MP:0003993) | MP:0003993 |
|  |  | [abnormal cardiac muscle morphology](http://www.informatics.jax.org/searches/Phat.cgi?id=MP:0005329) | MP:0005329 |
|  |  | [abnormal cerebral aqueduct morphology](http://www.informatics.jax.org/searches/Phat.cgi?id=MP:0005537) | MP:0005537 |
|  |  | [abnormal neuronal migration](http://www.informatics.jax.org/searches/Phat.cgi?id=MP:0006009) | MP:0006009 |
|  |  | [embryonic lethality during organogenesis](http://www.informatics.jax.org/searches/Phat.cgi?id=MP:0006207) | MP:0006207 |
|  |  | [abnormal neocortex morphology](http://www.informatics.jax.org/searches/Phat.cgi?id=MP:0008547) | MP:0008547 |
| [Fcer1a](http://www.informatics.jax.org/javawi2/servlet/WIFetch?page=markerDetail&id=MGI:95494) | MGI:2383982 | [allergic response](http://www.informatics.jax.org/searches/Phat.cgi?id=MP:0001878) | MP:0001878 |
|  |  | [abnormal mast cell physiology](http://www.informatics.jax.org/searches/Phat.cgi?id=MP:0002423) | MP:0002423 |
|  |  | [abnormal macrophage physiology](http://www.informatics.jax.org/searches/Phat.cgi?id=MP:0002451) | MP:0002451 |
|  |  | [abnormal type I hypersensitivity reaction](http://www.informatics.jax.org/searches/Phat.cgi?id=MP:0002531) | MP:0002531 |
|  |  | [increased vascular permeability](http://www.informatics.jax.org/searches/Phat.cgi?id=MP:0003070) | MP:0003070 |
|  |  | [reduced thrombolysis](http://www.informatics.jax.org/searches/Phat.cgi?id=MP:0003423) | MP:0003423 |
|  |  | [increased susceptibility to type I hypersensitivity reaction](http://www.informatics.jax.org/searches/Phat.cgi?id=MP:0005596) | MP:0005596 |
|  |  | [decreased susceptibility to type I hypersensitivity reaction](http://www.informatics.jax.org/searches/Phat.cgi?id=MP:0005597) | MP:0005597 |
|  |  | [increased tumor necrosis factor secretion](http://www.informatics.jax.org/searches/Phat.cgi?id=MP:0008560) | MP:0008560 |
|  | MGI:3712201 | [abnormal humoral immune response](http://www.informatics.jax.org/searches/Phat.cgi?id=MP:0001800) | MP:0001800 |
|  |  | [abnormal mast cell physiology](http://www.informatics.jax.org/searches/Phat.cgi?id=MP:0002423) | MP:0002423 |
|  |  | [decreased eosinophil cell number](http://www.informatics.jax.org/searches/Phat.cgi?id=MP:0005012) | MP:0005012 |
|  |  | [decreased circulating interleukin-4 level](http://www.informatics.jax.org/searches/Phat.cgi?id=MP:0008603) | MP:0008603 |
| [Gata2](http://www.informatics.jax.org/javawi2/servlet/WIFetch?page=markerDetail&id=MGI:95662) | MGI:1857743 | [enlarged pericardium](http://www.informatics.jax.org/searches/Phat.cgi?id=MP:0000291) | MP:0000291 |
|  |  | [anemia](http://www.informatics.jax.org/searches/Phat.cgi?id=MP:0001577) | MP:0001577 |
|  |  | [impaired hematopoiesis](http://www.informatics.jax.org/searches/Phat.cgi?id=MP:0001606) | MP:0001606 |
|  |  | [absent vitelline blood vessels](http://www.informatics.jax.org/searches/Phat.cgi?id=MP:0001719) | MP:0001719 |
|  |  | [pale yolk sac](http://www.informatics.jax.org/searches/Phat.cgi?id=MP:0001722) | MP:0001722 |
|  |  | [abnormal embryonic hematopoiesis](http://www.informatics.jax.org/searches/Phat.cgi?id=MP:0003396) | MP:0003396 |
|  |  | [embryonic lethality during organogenesis](http://www.informatics.jax.org/searches/Phat.cgi?id=MP:0006207) | MP:0006207 |
|  | MGI:3628640 | [anemia](http://www.informatics.jax.org/searches/Phat.cgi?id=MP:0001577) | MP:0001577 |
|  |  | [embryonic lethality during organogenesis](http://www.informatics.jax.org/searches/Phat.cgi?id=MP:0006207) | MP:0006207 |
| [H2-Aa](http://www.informatics.jax.org/javawi2/servlet/WIFetch?page=markerDetail&id=MGI:95895) | MGI:2157977 | [enlarged lymph nodes](http://www.informatics.jax.org/searches/Phat.cgi?id=MP:0000702) | MP:0000702 |
|  |  | [thymus hyperplasia](http://www.informatics.jax.org/searches/Phat.cgi?id=MP:0000708) | MP:0000708 |
|  |  | [enlarged thymus](http://www.informatics.jax.org/searches/Phat.cgi?id=MP:0000709) | MP:0000709 |
|  |  | [abnormal MHC II cell surface expression on macrophages](http://www.informatics.jax.org/searches/Phat.cgi?id=MP:0005040) | MP:0005040 |
|  |  | [abnormal T cell morphology](http://www.informatics.jax.org/searches/Phat.cgi?id=MP:0008037) | MP:0008037 |
|  |  | [decreased CD4-positive T cell number](http://www.informatics.jax.org/searches/Phat.cgi?id=MP:0008075) | MP:0008075 |
|  |  | [increased CD8-positive T cell number](http://www.informatics.jax.org/searches/Phat.cgi?id=MP:0008078) | MP:0008078 |
|  |  | [abnormal splenocyte physiology](http://www.informatics.jax.org/searches/Phat.cgi?id=MP:0009333) | MP:0009333 |
| [H2-T23](http://www.informatics.jax.org/javawi2/servlet/WIFetch?page=markerDetail&id=MGI:95957) | MGI:3044027 | [increased susceptibility to bacterial infection](http://www.informatics.jax.org/searches/Phat.cgi?id=MP:0002412) | MP:0002412 |
|  |  | [increased susceptibility to viral infection](http://www.informatics.jax.org/searches/Phat.cgi?id=MP:0002418) | MP:0002418 |
|  |  | [abnormal response/metabolism to endogenous compounds](http://www.informatics.jax.org/searches/Phat.cgi?id=MP:0003638) | MP:0003638 |
|  |  | [increased susceptibility to experimental autoimmune encephalomyelitis](http://www.informatics.jax.org/searches/Phat.cgi?id=MP:0004799) | MP:0004799 |
|  |  | [abnormal CD4-positive T cell physiology](http://www.informatics.jax.org/searches/Phat.cgi?id=MP:0005463) | MP:0005463 |
|  |  | [abnormal memory T cell physiology](http://www.informatics.jax.org/searches/Phat.cgi?id=MP:0008051) | MP:0008051 |
|  | MGI:4419817 |  |  |
| [Mst1](http://www.informatics.jax.org/javawi2/servlet/WIFetch?page=markerDetail&id=MGI:96080) | MGI:2445230 | [abnormal hepatocyte morphology](http://www.informatics.jax.org/searches/Phat.cgi?id=MP:0000607) | MP:0000607 |
|  |  | [abnormal macrophage physiology](http://www.informatics.jax.org/searches/Phat.cgi?id=MP:0002451) | MP:0002451 |
|  |  | [increased susceptibility to induced colitis](http://www.informatics.jax.org/searches/Phat.cgi?id=MP:0008537) | MP:0008537 |
|  | MGI:4431447 |  |  |
| [Hhex](http://www.informatics.jax.org/javawi2/servlet/WIFetch?page=markerDetail&id=MGI:96086) | MGI:2448661 | [abnormal liver development](http://www.informatics.jax.org/searches/Phat.cgi?id=MP:0000596) | MP:0000596 |
|  |  | [abnormal hepatocyte morphology](http://www.informatics.jax.org/searches/Phat.cgi?id=MP:0000607) | MP:0000607 |
|  |  | [impaired hematopoiesis](http://www.informatics.jax.org/searches/Phat.cgi?id=MP:0001606) | MP:0001606 |
|  |  | [embryonic lethality during organogenesis](http://www.informatics.jax.org/searches/Phat.cgi?id=MP:0006207) | MP:0006207 |
|  |  | [abnormal monocyte differentiation](http://www.informatics.jax.org/searches/Phat.cgi?id=MP:0008112) | MP:0008112 |
|  | MGI:2449914 | [thin ventricular wall](http://www.informatics.jax.org/searches/Phat.cgi?id=MP:0000280) | MP:0000280 |
|  |  | [abnormal ventricular septum morphology](http://www.informatics.jax.org/searches/Phat.cgi?id=MP:0000281) | MP:0000281 |
|  |  | [double outlet heart right ventricle](http://www.informatics.jax.org/searches/Phat.cgi?id=MP:0000284) | MP:0000284 |
|  |  | [abnormal endocardial cushion morphology](http://www.informatics.jax.org/searches/Phat.cgi?id=MP:0000297) | MP:0000297 |
|  |  | [abnormal liver development](http://www.informatics.jax.org/searches/Phat.cgi?id=MP:0000596) | MP:0000596 |
|  |  | [abnormal liver morphology](http://www.informatics.jax.org/searches/Phat.cgi?id=MP:0000598) | MP:0000598 |
|  |  | [abnormal blood vessel morphology](http://www.informatics.jax.org/searches/Phat.cgi?id=MP:0001614) | MP:0001614 |
|  |  | [abnormal vasculogenesis](http://www.informatics.jax.org/searches/Phat.cgi?id=MP:0001622) | MP:0001622 |
|  |  | [edema](http://www.informatics.jax.org/searches/Phat.cgi?id=MP:0001785) | MP:0001785 |
|  |  | [pericardial edema](http://www.informatics.jax.org/searches/Phat.cgi?id=MP:0001787) | MP:0001787 |
|  |  | [prenatal lethality](http://www.informatics.jax.org/searches/Phat.cgi?id=MP:0002080) | MP:0002080 |
|  |  | [abnormal myocardial trabeculae morphology](http://www.informatics.jax.org/searches/Phat.cgi?id=MP:0002189) | MP:0002189 |
|  |  | [abnormal atrioventricular valve morphology](http://www.informatics.jax.org/searches/Phat.cgi?id=MP:0002745) | MP:0002745 |
|  |  | [abnormal bile duct development](http://www.informatics.jax.org/searches/Phat.cgi?id=MP:0002929) | MP:0002929 |
|  |  | [abnormal forebrain development](http://www.informatics.jax.org/searches/Phat.cgi?id=MP:0003232) | MP:0003232 |
|  |  | [absent gall bladder](http://www.informatics.jax.org/searches/Phat.cgi?id=MP:0003250) | MP:0003250 |
|  |  | [biliary cyst](http://www.informatics.jax.org/searches/Phat.cgi?id=MP:0003266) | MP:0003266 |
|  |  | [liver cysts](http://www.informatics.jax.org/searches/Phat.cgi?id=MP:0003327) | MP:0003327 |
|  |  | [abnormal thyroid gland development](http://www.informatics.jax.org/searches/Phat.cgi?id=MP:0003421) | MP:0003421 |
|  |  | [decreased heart right ventricle size](http://www.informatics.jax.org/searches/Phat.cgi?id=MP:0003649) | MP:0003649 |
|  |  | [increased endocardial cushion size](http://www.informatics.jax.org/searches/Phat.cgi?id=MP:0003808) | MP:0003808 |
|  |  | [vascular smooth muscle cell hypoplasia](http://www.informatics.jax.org/searches/Phat.cgi?id=MP:0003814) | MP:0003814 |
|  |  | [thin myocardial compact layer](http://www.informatics.jax.org/searches/Phat.cgi?id=MP:0004057) | MP:0004057 |
|  |  | [abnormal outflow tract development](http://www.informatics.jax.org/searches/Phat.cgi?id=MP:0006126) | MP:0006126 |
|  |  | [embryonic lethality during organogenesis](http://www.informatics.jax.org/searches/Phat.cgi?id=MP:0006207) | MP:0006207 |
| [Lipc](http://www.informatics.jax.org/javawi2/servlet/WIFetch?page=markerDetail&id=MGI:96216) | MGI:1857182 | [abnormal circulating cholesterol level](http://www.informatics.jax.org/searches/Phat.cgi?id=MP:0000180) | MP:0000180 |
|  |  | [increased circulating LDL cholesterol level](http://www.informatics.jax.org/searches/Phat.cgi?id=MP:0000182) | MP:0000182 |
|  |  | [small ovary](http://www.informatics.jax.org/searches/Phat.cgi?id=MP:0001127) | MP:0001127 |
|  |  | [abnormal lipid level](http://www.informatics.jax.org/searches/Phat.cgi?id=MP:0001547) | MP:0001547 |
|  |  | [increased circulating triglyceride level](http://www.informatics.jax.org/searches/Phat.cgi?id=MP:0001552) | MP:0001552 |
|  |  | [increased circulating HDL cholesterol level](http://www.informatics.jax.org/searches/Phat.cgi?id=MP:0001556) | MP:0001556 |
|  |  | [abnormal reproductive system physiology](http://www.informatics.jax.org/searches/Phat.cgi?id=MP:0001919) | MP:0001919 |
|  |  | [decreased litter size](http://www.informatics.jax.org/searches/Phat.cgi?id=MP:0001935) | MP:0001935 |
|  |  | [decreased circulating corticosterone level](http://www.informatics.jax.org/searches/Phat.cgi?id=MP:0002665) | MP:0002665 |
|  |  | [decreased ovulation frequency](http://www.informatics.jax.org/searches/Phat.cgi?id=MP:0003355) | MP:0003355 |
|  |  | [increased circulating cholesterol level](http://www.informatics.jax.org/searches/Phat.cgi?id=MP:0005178) | MP:0005178 |
|  |  | [abnormal circulating phospholipid level](http://www.informatics.jax.org/searches/Phat.cgi?id=MP:0006084) | MP:0006084 |
| [Icam1](http://www.informatics.jax.org/javawi2/servlet/WIFetch?page=markerDetail&id=MGI:96392) | MGI:1857292 | [increased neutrophil cell number](http://www.informatics.jax.org/searches/Phat.cgi?id=MP:0000219) | MP:0000219 |
|  |  | [increased monocyte cell number](http://www.informatics.jax.org/searches/Phat.cgi?id=MP:0000220) | MP:0000220 |
|  |  | [abnormal T cell physiology](http://www.informatics.jax.org/searches/Phat.cgi?id=MP:0002444) | MP:0002444 |
|  |  | [abnormal mononuclear leukocyte differentiation](http://www.informatics.jax.org/searches/Phat.cgi?id=MP:0002445) | MP:0002445 |
|  |  | [abnormal cardiac muscle contractility](http://www.informatics.jax.org/searches/Phat.cgi?id=MP:0002972) | MP:0002972 |
|  |  | [decreased susceptibility to autoimmune diabetes](http://www.informatics.jax.org/searches/Phat.cgi?id=MP:0004804) | MP:0004804 |
|  |  | [increased lymphocyte cell number](http://www.informatics.jax.org/searches/Phat.cgi?id=MP:0005013) | MP:0005013 |
|  |  | [decreased circulating cholesterol level](http://www.informatics.jax.org/searches/Phat.cgi?id=MP:0005179) | MP:0005179 |
|  |  | [decreased susceptibility to atherosclerosis](http://www.informatics.jax.org/searches/Phat.cgi?id=MP:0005341) | MP:0005341 |
|  |  | [periinsulitis](http://www.informatics.jax.org/searches/Phat.cgi?id=MP:0005580) | MP:0005580 |
|  |  | [decreased susceptibility to type IV hypersensitivity reaction](http://www.informatics.jax.org/searches/Phat.cgi?id=MP:0005616) | MP:0005616 |
|  |  | [abnormal interleukin level](http://www.informatics.jax.org/searches/Phat.cgi?id=MP:0008751) | MP:0008751 |
|  |  | [abnormal tumor necrosis factor level](http://www.informatics.jax.org/searches/Phat.cgi?id=MP:0008752) | MP:0008752 |
|  |  | [decreased susceptibility to bacterial infection induced morbidity/mortality](http://www.informatics.jax.org/searches/Phat.cgi?id=MP:0009789) | MP:0009789 |
|  | MGI:3588437 | [abnormal macrophage morphology](http://www.informatics.jax.org/searches/Phat.cgi?id=MP:0002446) | MP:0002446 |
|  | MGI:3605161 | [increased leukocyte cell number](http://www.informatics.jax.org/searches/Phat.cgi?id=MP:0000218) | MP:0000218 |
|  |  | [increased neutrophil cell number](http://www.informatics.jax.org/searches/Phat.cgi?id=MP:0000219) | MP:0000219 |
|  |  | [abnormal leukocyte tethering or rolling](http://www.informatics.jax.org/searches/Phat.cgi?id=MP:0003627) | MP:0003627 |
|  |  | [abnormal leukocyte adhesion](http://www.informatics.jax.org/searches/Phat.cgi?id=MP:0003628) | MP:0003628 |
|  | MGI:1857183 | [increased leukocyte cell number](http://www.informatics.jax.org/searches/Phat.cgi?id=MP:0000218) | MP:0000218 |
|  |  | [increased neutrophil cell number](http://www.informatics.jax.org/searches/Phat.cgi?id=MP:0000219) | MP:0000219 |
|  |  | [increased monocyte cell number](http://www.informatics.jax.org/searches/Phat.cgi?id=MP:0000220) | MP:0000220 |
|  |  | [increased body weight](http://www.informatics.jax.org/searches/Phat.cgi?id=MP:0001260) | MP:0001260 |
|  |  | [abnormal retina morphology](http://www.informatics.jax.org/searches/Phat.cgi?id=MP:0001325) | MP:0001325 |
|  |  | [edema](http://www.informatics.jax.org/searches/Phat.cgi?id=MP:0001785) | MP:0001785 |
|  |  | [skin edema](http://www.informatics.jax.org/searches/Phat.cgi?id=MP:0001786) | MP:0001786 |
|  |  | [abnormal immune system physiology](http://www.informatics.jax.org/searches/Phat.cgi?id=MP:0001790) | MP:0001790 |
|  |  | [impaired wound healing](http://www.informatics.jax.org/searches/Phat.cgi?id=MP:0001792) | MP:0001792 |
|  |  | [abnormal inflammatory response](http://www.informatics.jax.org/searches/Phat.cgi?id=MP:0001845) | MP:0001845 |
|  |  | [premature death](http://www.informatics.jax.org/searches/Phat.cgi?id=MP:0002083) | MP:0002083 |
|  |  | [abnormal mast cell physiology](http://www.informatics.jax.org/searches/Phat.cgi?id=MP:0002423) | MP:0002423 |
|  |  | [abnormal macrophage physiology](http://www.informatics.jax.org/searches/Phat.cgi?id=MP:0002451) | MP:0002451 |
|  |  | [abnormal retinal vasculature morphology](http://www.informatics.jax.org/searches/Phat.cgi?id=MP:0002792) | MP:0002792 |
|  |  | [delayed wound healing](http://www.informatics.jax.org/searches/Phat.cgi?id=MP:0002908) | MP:0002908 |
|  |  | [abnormal cell migration](http://www.informatics.jax.org/searches/Phat.cgi?id=MP:0003091) | MP:0003091 |
|  |  | [abnormal leukocyte migration](http://www.informatics.jax.org/searches/Phat.cgi?id=MP:0003156) | MP:0003156 |
|  |  | [large intestinal inflammation](http://www.informatics.jax.org/searches/Phat.cgi?id=MP:0003304) | MP:0003304 |
|  |  | [increased resistance to induced choroidal neovascularization](http://www.informatics.jax.org/searches/Phat.cgi?id=MP:0003434) | MP:0003434 |
|  |  | [abnormal keratinocyte physiology](http://www.informatics.jax.org/searches/Phat.cgi?id=MP:0003453) | MP:0003453 |
|  |  | [abnormal leukocyte tethering or rolling](http://www.informatics.jax.org/searches/Phat.cgi?id=MP:0003627) | MP:0003627 |
|  |  | [increased tumor growth/size](http://www.informatics.jax.org/searches/Phat.cgi?id=MP:0003721) | MP:0003721 |
|  |  | [impaired macrophage migration](http://www.informatics.jax.org/searches/Phat.cgi?id=MP:0003799) | MP:0003799 |
|  |  | [abnormal nitric oxide homeostasis](http://www.informatics.jax.org/searches/Phat.cgi?id=MP:0003957) | MP:0003957 |
|  |  | [increased eosinophil cell number](http://www.informatics.jax.org/searches/Phat.cgi?id=MP:0005011) | MP:0005011 |
|  |  | [increased lymphocyte cell number](http://www.informatics.jax.org/searches/Phat.cgi?id=MP:0005013) | MP:0005013 |
|  |  | [decreased acute inflammation](http://www.informatics.jax.org/searches/Phat.cgi?id=MP:0005087) | MP:0005087 |
|  |  | [abnormal eye physiology](http://www.informatics.jax.org/searches/Phat.cgi?id=MP:0005253) | MP:0005253 |
|  |  | [increased circulating glucose level](http://www.informatics.jax.org/searches/Phat.cgi?id=MP:0005559) | MP:0005559 |
|  |  | [decreased angiogenesis](http://www.informatics.jax.org/searches/Phat.cgi?id=MP:0005602) | MP:0005602 |
|  |  | [decreased susceptibility to type IV hypersensitivity reaction](http://www.informatics.jax.org/searches/Phat.cgi?id=MP:0005616) | MP:0005616 |
|  |  | [abnormal cell physiology](http://www.informatics.jax.org/searches/Phat.cgi?id=MP:0005621) | MP:0005621 |
|  |  | [decreased susceptibility to induced colitis](http://www.informatics.jax.org/searches/Phat.cgi?id=MP:0008539) | MP:0008539 |
|  |  | [impaired neutrophil recruitment](http://www.informatics.jax.org/searches/Phat.cgi?id=MP:0008719) | MP:0008719 |
|  |  | [impaired neutrophil migration](http://www.informatics.jax.org/searches/Phat.cgi?id=MP:0008720) | MP:0008720 |
|  |  | [decreased susceptibility to endotoxin shock](http://www.informatics.jax.org/searches/Phat.cgi?id=MP:0008734) | MP:0008734 |
|  |  | [abnormal cellular extravasation](http://www.informatics.jax.org/searches/Phat.cgi?id=MP:0009858) | MP:0009858 |
| [Il10](http://www.informatics.jax.org/javawi2/servlet/WIFetch?page=markerDetail&id=MGI:96537) | MGI:1857199 | [absent erythroid progenitor cell](http://www.informatics.jax.org/searches/Phat.cgi?id=MP:0000216) | MP:0000216 |
|  |  | [increased neutrophil cell number](http://www.informatics.jax.org/searches/Phat.cgi?id=MP:0000219) | MP:0000219 |
|  |  | [increased granulocyte number](http://www.informatics.jax.org/searches/Phat.cgi?id=MP:0000322) | MP:0000322 |
|  |  | [abnormal enterocyte morphology](http://www.informatics.jax.org/searches/Phat.cgi?id=MP:0000479) | MP:0000479 |
|  |  | [abnormal intestinal epithelium morphology](http://www.informatics.jax.org/searches/Phat.cgi?id=MP:0000488) | MP:0000488 |
|  |  | [abnormal crypts of Lieberkuhn morphology](http://www.informatics.jax.org/searches/Phat.cgi?id=MP:0000490) | MP:0000490 |
|  |  | [rectal prolapse](http://www.informatics.jax.org/searches/Phat.cgi?id=MP:0000493) | MP:0000493 |
|  |  | [abnormal colon morphology](http://www.informatics.jax.org/searches/Phat.cgi?id=MP:0000495) | MP:0000495 |
|  |  | [abnormal intestinal mucosa morphology](http://www.informatics.jax.org/searches/Phat.cgi?id=MP:0000511) | MP:0000511 |
|  |  | [enlarged spleen](http://www.informatics.jax.org/searches/Phat.cgi?id=MP:0000691) | MP:0000691 |
|  |  | [thymus hyperplasia](http://www.informatics.jax.org/searches/Phat.cgi?id=MP:0000708) | MP:0000708 |
|  |  | [weight loss](http://www.informatics.jax.org/searches/Phat.cgi?id=MP:0001263) | MP:0001263 |
|  |  | [anemia](http://www.informatics.jax.org/searches/Phat.cgi?id=MP:0001577) | MP:0001577 |
|  |  | [abnormal myelopoiesis](http://www.informatics.jax.org/searches/Phat.cgi?id=MP:0001601) | MP:0001601 |
|  |  | [postnatal growth retardation](http://www.informatics.jax.org/searches/Phat.cgi?id=MP:0001732) | MP:0001732 |
|  |  | [abnormal iron level](http://www.informatics.jax.org/searches/Phat.cgi?id=MP:0001770) | MP:0001770 |
|  |  | [abnormal immune system physiology](http://www.informatics.jax.org/searches/Phat.cgi?id=MP:0001790) | MP:0001790 |
|  |  | [thymus hypoplasia](http://www.informatics.jax.org/searches/Phat.cgi?id=MP:0001823) | MP:0001823 |
|  |  | [abnormal thymus involution](http://www.informatics.jax.org/searches/Phat.cgi?id=MP:0001824) | MP:0001824 |
|  |  | [abnormal inflammatory response](http://www.informatics.jax.org/searches/Phat.cgi?id=MP:0001845) | MP:0001845 |
|  |  | [intestinal inflammation](http://www.informatics.jax.org/searches/Phat.cgi?id=MP:0001858) | MP:0001858 |
|  |  | [abnormal reproductive system physiology](http://www.informatics.jax.org/searches/Phat.cgi?id=MP:0001919) | MP:0001919 |
|  |  | [premature death](http://www.informatics.jax.org/searches/Phat.cgi?id=MP:0002083) | MP:0002083 |
|  |  | [decreased airway responsiveness](http://www.informatics.jax.org/searches/Phat.cgi?id=MP:0002335) | MP:0002335 |
|  |  | [increased IgE level](http://www.informatics.jax.org/searches/Phat.cgi?id=MP:0002497) | MP:0002497 |
|  |  | [chronic inflammation](http://www.informatics.jax.org/searches/Phat.cgi?id=MP:0002499) | MP:0002499 |
|  |  | [microcytic anemia](http://www.informatics.jax.org/searches/Phat.cgi?id=MP:0002810) | MP:0002810 |
|  |  | [colitis](http://www.informatics.jax.org/searches/Phat.cgi?id=MP:0002816) | MP:0002816 |
|  |  | [decreased hemoglobin content](http://www.informatics.jax.org/searches/Phat.cgi?id=MP:0002874) | MP:0002874 |
|  |  | [decreased erythrocyte cell number](http://www.informatics.jax.org/searches/Phat.cgi?id=MP:0002875) | MP:0002875 |
|  |  | [intestinal adenocarcinoma](http://www.informatics.jax.org/searches/Phat.cgi?id=MP:0002957) | MP:0002957 |
|  |  | [abnormal cytokine secretion](http://www.informatics.jax.org/searches/Phat.cgi?id=MP:0003009) | MP:0003009 |
|  |  | [hypoferremia](http://www.informatics.jax.org/searches/Phat.cgi?id=MP:0004151) | MP:0004151 |
|  |  | [abnormal regulatory T cell physiology](http://www.informatics.jax.org/searches/Phat.cgi?id=MP:0004946) | MP:0004946 |
|  |  | [increased thymus weight](http://www.informatics.jax.org/searches/Phat.cgi?id=MP:0004955) | MP:0004955 |
|  |  | [decreased lymphocyte cell number](http://www.informatics.jax.org/searches/Phat.cgi?id=MP:0005016) | MP:0005016 |
|  |  | [abnormal response to infection](http://www.informatics.jax.org/searches/Phat.cgi?id=MP:0005025) | MP:0005025 |
|  |  | [increased susceptibility to parasitic infection](http://www.informatics.jax.org/searches/Phat.cgi?id=MP:0005027) | MP:0005027 |
|  |  | [abnormal level of surface class II molecules](http://www.informatics.jax.org/searches/Phat.cgi?id=MP:0005042) | MP:0005042 |
|  |  | [absent spleen white pulp](http://www.informatics.jax.org/searches/Phat.cgi?id=MP:0005046) | MP:0005046 |
|  |  | [decreased T cell proliferation](http://www.informatics.jax.org/searches/Phat.cgi?id=MP:0005095) | MP:0005095 |
|  |  | [cachexia](http://www.informatics.jax.org/searches/Phat.cgi?id=MP:0005150) | MP:0005150 |
|  |  | [increased macrophage cell number](http://www.informatics.jax.org/searches/Phat.cgi?id=MP:0005425) | MP:0005425 |
|  |  | [abnormal CD4-positive T cell physiology](http://www.informatics.jax.org/searches/Phat.cgi?id=MP:0005463) | MP:0005463 |
|  |  | [increased T cell apoptosis](http://www.informatics.jax.org/searches/Phat.cgi?id=MP:0006413) | MP:0006413 |
|  |  | [abnormal T-helper 1 cell differentiation](http://www.informatics.jax.org/searches/Phat.cgi?id=MP:0008088) | MP:0008088 |
|  |  | [abnormal T-helper 2 cell differentiation](http://www.informatics.jax.org/searches/Phat.cgi?id=MP:0008092) | MP:0008092 |
|  |  | [hypochromic anemia](http://www.informatics.jax.org/searches/Phat.cgi?id=MP:0008387) | MP:0008387 |
|  |  | [enlarged mesenteric lymph nodes](http://www.informatics.jax.org/searches/Phat.cgi?id=MP:0008466) | MP:0008466 |
|  |  | [increased spleen red pulp amount](http://www.informatics.jax.org/searches/Phat.cgi?id=MP:0008476) | MP:0008476 |
|  |  | [decreased spleen red pulp amount](http://www.informatics.jax.org/searches/Phat.cgi?id=MP:0008477) | MP:0008477 |
|  |  | [increased IgG1 level](http://www.informatics.jax.org/searches/Phat.cgi?id=MP:0008499) | MP:0008499 |
|  |  | [increased IgG2a level](http://www.informatics.jax.org/searches/Phat.cgi?id=MP:0008500) | MP:0008500 |
|  |  | [increased susceptibility to induced colitis](http://www.informatics.jax.org/searches/Phat.cgi?id=MP:0008537) | MP:0008537 |
|  |  | [increased circulating tumor necrosis factor level](http://www.informatics.jax.org/searches/Phat.cgi?id=MP:0008553) | MP:0008553 |
|  |  | [increased tumor necrosis factor secretion](http://www.informatics.jax.org/searches/Phat.cgi?id=MP:0008560) | MP:0008560 |
|  |  | [increased interferon-gamma secretion](http://www.informatics.jax.org/searches/Phat.cgi?id=MP:0008566) | MP:0008566 |
|  |  | [increased circulating interferon-gamma level](http://www.informatics.jax.org/searches/Phat.cgi?id=MP:0008577) | MP:0008577 |
|  |  | [increased circulating interleukin-6 level](http://www.informatics.jax.org/searches/Phat.cgi?id=MP:0008596) | MP:0008596 |
|  |  | [increased circulating interleukin-2 level](http://www.informatics.jax.org/searches/Phat.cgi?id=MP:0008599) | MP:0008599 |
|  |  | [increased circulating interleukin-17 level](http://www.informatics.jax.org/searches/Phat.cgi?id=MP:0008614) | MP:0008614 |
|  |  | [increased circulating interleukin-12 level](http://www.informatics.jax.org/searches/Phat.cgi?id=MP:0008617) | MP:0008617 |
|  |  | [increased circulating interleukin-1 alpha level](http://www.informatics.jax.org/searches/Phat.cgi?id=MP:0008638) | MP:0008638 |
|  |  | [increased circulating interleukin-1 beta level](http://www.informatics.jax.org/searches/Phat.cgi?id=MP:0008641) | MP:0008641 |
|  |  | [decreased interleukin-10 secretion](http://www.informatics.jax.org/searches/Phat.cgi?id=MP:0008661) | MP:0008661 |
|  |  | [increased interleukin-12a secretion](http://www.informatics.jax.org/searches/Phat.cgi?id=MP:0008666) | MP:0008666 |
|  |  | [increased interleukin-12b secretion](http://www.informatics.jax.org/searches/Phat.cgi?id=MP:0008669) | MP:0008669 |
|  |  | [increased interleukin-13 secretion](http://www.informatics.jax.org/searches/Phat.cgi?id=MP:0008672) | MP:0008672 |
|  |  | [increased interleukin-6 secretion](http://www.informatics.jax.org/searches/Phat.cgi?id=MP:0008705) | MP:0008705 |
|  |  | [abnormal cytokine level](http://www.informatics.jax.org/searches/Phat.cgi?id=MP:0008713) | MP:0008713 |
|  |  | [abnormal chemokine level](http://www.informatics.jax.org/searches/Phat.cgi?id=MP:0008721) | MP:0008721 |
|  |  | [increased susceptibility to endotoxin shock](http://www.informatics.jax.org/searches/Phat.cgi?id=MP:0008735) | MP:0008735 |
|  |  | [abnormal interferon level](http://www.informatics.jax.org/searches/Phat.cgi?id=MP:0008750) | MP:0008750 |
|  |  | [abnormal interleukin level](http://www.informatics.jax.org/searches/Phat.cgi?id=MP:0008751) | MP:0008751 |
|  |  | [abnormal tumor necrosis factor level](http://www.informatics.jax.org/searches/Phat.cgi?id=MP:0008752) | MP:0008752 |
|  |  | [decreased spleen iron level](http://www.informatics.jax.org/searches/Phat.cgi?id=MP:0008808) | MP:0008808 |
|  |  | [abnormal splenic cell ratio](http://www.informatics.jax.org/searches/Phat.cgi?id=MP:0008826) | MP:0008826 |
|  |  | [abnormal lymph node cell ratio](http://www.informatics.jax.org/searches/Phat.cgi?id=MP:0008828) | MP:0008828 |
|  |  | [cecum inflammation](http://www.informatics.jax.org/searches/Phat.cgi?id=MP:0009481) | MP:0009481 |
|  |  | [abnormal thymus corticomedullary boundary morphology](http://www.informatics.jax.org/searches/Phat.cgi?id=MP:0009543) | MP:0009543 |
|  |  | [increased leukotriene level](http://www.informatics.jax.org/searches/Phat.cgi?id=MP:0009816) | MP:0009816 |
|  | MGI:3521569 | [spleen hyperplasia](http://www.informatics.jax.org/searches/Phat.cgi?id=MP:0000693) | MP:0000693 |
|  |  | [intestinal inflammation](http://www.informatics.jax.org/searches/Phat.cgi?id=MP:0001858) | MP:0001858 |
|  |  | [hypersensitivity](http://www.informatics.jax.org/searches/Phat.cgi?id=MP:0002148) | MP:0002148 |
|  |  | [increased susceptibility to bacterial infection](http://www.informatics.jax.org/searches/Phat.cgi?id=MP:0002412) | MP:0002412 |
|  |  | [abnormal innate immunity](http://www.informatics.jax.org/searches/Phat.cgi?id=MP:0002419) | MP:0002419 |
|  |  | [abnormal cytokine secretion](http://www.informatics.jax.org/searches/Phat.cgi?id=MP:0003009) | MP:0003009 |
|  |  | [increased B cell number](http://www.informatics.jax.org/searches/Phat.cgi?id=MP:0005014) | MP:0005014 |
|  |  | [increased T cell number](http://www.informatics.jax.org/searches/Phat.cgi?id=MP:0005015) | MP:0005015 |
|  |  | [increased plasma cell number](http://www.informatics.jax.org/searches/Phat.cgi?id=MP:0008097) | MP:0008097 |
|  |  | [increased myeloid dendritic cell number](http://www.informatics.jax.org/searches/Phat.cgi?id=MP:0008121) | MP:0008121 |
|  |  | [abnormal myeloid leukocyte morphology](http://www.informatics.jax.org/searches/Phat.cgi?id=MP:0008250) | MP:0008250 |
|  |  | [decreased circulating interleukin-10 level](http://www.informatics.jax.org/searches/Phat.cgi?id=MP:0008594) | MP:0008594 |
|  |  | [increased susceptibility to endotoxin shock](http://www.informatics.jax.org/searches/Phat.cgi?id=MP:0008735) | MP:0008735 |
|  |  | [increased sensitivity to induced morbidity/mortality](http://www.informatics.jax.org/searches/Phat.cgi?id=MP:0009763) | MP:0009763 |
| [Il12b](http://www.informatics.jax.org/javawi2/servlet/WIFetch?page=markerDetail&id=MGI:96540) | MGI:1857201 | [lung inflammation](http://www.informatics.jax.org/searches/Phat.cgi?id=MP:0001861) | MP:0001861 |
|  |  | [increased susceptibility to bacterial infection](http://www.informatics.jax.org/searches/Phat.cgi?id=MP:0002412) | MP:0002412 |
|  |  | [abnormal macrophage physiology](http://www.informatics.jax.org/searches/Phat.cgi?id=MP:0002451) | MP:0002451 |
|  |  | [abnormal granulocyte physiology](http://www.informatics.jax.org/searches/Phat.cgi?id=MP:0002462) | MP:0002462 |
|  |  | [small intestinal inflammation](http://www.informatics.jax.org/searches/Phat.cgi?id=MP:0003306) | MP:0003306 |
|  |  | [abnormal lymphocyte physiology](http://www.informatics.jax.org/searches/Phat.cgi?id=MP:0003945) | MP:0003945 |
|  |  | [abnormal CD8-positive T cell physiology](http://www.informatics.jax.org/searches/Phat.cgi?id=MP:0004392) | MP:0004392 |
|  |  | [decreased incidence of chemically-induced tumors](http://www.informatics.jax.org/searches/Phat.cgi?id=MP:0004502) | MP:0004502 |
|  |  | [increased susceptibility to parasitic infection](http://www.informatics.jax.org/searches/Phat.cgi?id=MP:0005027) | MP:0005027 |
|  |  | [abnormal NK cell physiology](http://www.informatics.jax.org/searches/Phat.cgi?id=MP:0005069) | MP:0005069 |
|  |  | [impaired NK cell cytolysis](http://www.informatics.jax.org/searches/Phat.cgi?id=MP:0005070) | MP:0005070 |
|  |  | [abnormal CD4-positive T cell physiology](http://www.informatics.jax.org/searches/Phat.cgi?id=MP:0005463) | MP:0005463 |
|  |  | [abnormal T-helper 1 physiology](http://www.informatics.jax.org/searches/Phat.cgi?id=MP:0005465) | MP:0005465 |
|  |  | [decreased susceptibility to type IV hypersensitivity reaction](http://www.informatics.jax.org/searches/Phat.cgi?id=MP:0005616) | MP:0005616 |
|  |  | [abnormal T-helper 2 cell differentiation](http://www.informatics.jax.org/searches/Phat.cgi?id=MP:0008092) | MP:0008092 |
|  |  | [abnormal interferon secretion](http://www.informatics.jax.org/searches/Phat.cgi?id=MP:0008555) | MP:0008555 |
|  |  | [decreased interferon-gamma secretion](http://www.informatics.jax.org/searches/Phat.cgi?id=MP:0008567) | MP:0008567 |
|  |  | [decreased circulating interferon-gamma level](http://www.informatics.jax.org/searches/Phat.cgi?id=MP:0008578) | MP:0008578 |
|  |  | [increased interleukin-13 secretion](http://www.informatics.jax.org/searches/Phat.cgi?id=MP:0008672) | MP:0008672 |
|  |  | [decreased interleukin-17 secretion](http://www.informatics.jax.org/searches/Phat.cgi?id=MP:0008682) | MP:0008682 |
|  |  | [abnormal chemokine secretion](http://www.informatics.jax.org/searches/Phat.cgi?id=MP:0008722) | MP:0008722 |
| [Il2](http://www.informatics.jax.org/javawi2/servlet/WIFetch?page=markerDetail&id=MGI:96548) | MGI:1857191 | [abnormal bone marrow cell number](http://www.informatics.jax.org/searches/Phat.cgi?id=MP:0000172) | MP:0000172 |
|  |  | [decreased hematocrit](http://www.informatics.jax.org/searches/Phat.cgi?id=MP:0000208) | MP:0000208 |
|  |  | [abnormal intestinal epithelium morphology](http://www.informatics.jax.org/searches/Phat.cgi?id=MP:0000488) | MP:0000488 |
|  |  | [crypts of Lieberkuhn abscesses](http://www.informatics.jax.org/searches/Phat.cgi?id=MP:0000491) | MP:0000491 |
|  |  | [rectal prolapse](http://www.informatics.jax.org/searches/Phat.cgi?id=MP:0000493) | MP:0000493 |
|  |  | [intestinal ulcer](http://www.informatics.jax.org/searches/Phat.cgi?id=MP:0000512) | MP:0000512 |
|  |  | [amyloidosis](http://www.informatics.jax.org/searches/Phat.cgi?id=MP:0000604) | MP:0000604 |
|  |  | [abnormal spleen morphology](http://www.informatics.jax.org/searches/Phat.cgi?id=MP:0000689) | MP:0000689 |
|  |  | [enlarged spleen](http://www.informatics.jax.org/searches/Phat.cgi?id=MP:0000691) | MP:0000691 |
|  |  | [spleen hyperplasia](http://www.informatics.jax.org/searches/Phat.cgi?id=MP:0000693) | MP:0000693 |
|  |  | [enlarged lymph nodes](http://www.informatics.jax.org/searches/Phat.cgi?id=MP:0000702) | MP:0000702 |
|  |  | [abnormal lymphocyte cell number](http://www.informatics.jax.org/searches/Phat.cgi?id=MP:0000717) | MP:0000717 |
|  |  | [hunched posture](http://www.informatics.jax.org/searches/Phat.cgi?id=MP:0001505) | MP:0001505 |
|  |  | [anemia](http://www.informatics.jax.org/searches/Phat.cgi?id=MP:0001577) | MP:0001577 |
|  |  | [chronic diarrhea](http://www.informatics.jax.org/searches/Phat.cgi?id=MP:0001665) | MP:0001665 |
|  |  | [decreased IgG level](http://www.informatics.jax.org/searches/Phat.cgi?id=MP:0001805) | MP:0001805 |
|  |  | [increased activated T cell number](http://www.informatics.jax.org/searches/Phat.cgi?id=MP:0001829) | MP:0001829 |
|  |  | [increased inflammatory response](http://www.informatics.jax.org/searches/Phat.cgi?id=MP:0001846) | MP:0001846 |
|  |  | [heart inflammation](http://www.informatics.jax.org/searches/Phat.cgi?id=MP:0001853) | MP:0001853 |
|  |  | [liver inflammation](http://www.informatics.jax.org/searches/Phat.cgi?id=MP:0001860) | MP:0001860 |
|  |  | [lung inflammation](http://www.informatics.jax.org/searches/Phat.cgi?id=MP:0001861) | MP:0001861 |
|  |  | [vasculitis](http://www.informatics.jax.org/searches/Phat.cgi?id=MP:0001864) | MP:0001864 |
|  |  | [pancreas inflammation](http://www.informatics.jax.org/searches/Phat.cgi?id=MP:0001869) | MP:0001869 |
|  |  | [premature death](http://www.informatics.jax.org/searches/Phat.cgi?id=MP:0002083) | MP:0002083 |
|  |  | [increased susceptibility to viral infection](http://www.informatics.jax.org/searches/Phat.cgi?id=MP:0002418) | MP:0002418 |
|  |  | [abnormal reticulocyte morphology](http://www.informatics.jax.org/searches/Phat.cgi?id=MP:0002424) | MP:0002424 |
|  |  | [abnormal blood cell morphology/development](http://www.informatics.jax.org/searches/Phat.cgi?id=MP:0002429) | MP:0002429 |
|  |  | [abnormal CD8-positive, alpha-beta cytotoxic T cell morphology](http://www.informatics.jax.org/searches/Phat.cgi?id=MP:0002436) | MP:0002436 |
|  |  | [increased IgG level](http://www.informatics.jax.org/searches/Phat.cgi?id=MP:0002493) | MP:0002493 |
|  |  | [increased IgA level](http://www.informatics.jax.org/searches/Phat.cgi?id=MP:0002495) | MP:0002495 |
|  |  | [increased IgE level](http://www.informatics.jax.org/searches/Phat.cgi?id=MP:0002497) | MP:0002497 |
|  |  | [low mean erythrocyte cell number](http://www.informatics.jax.org/searches/Phat.cgi?id=MP:0002594) | MP:0002594 |
|  |  | [abnormal lymphocyte morphology](http://www.informatics.jax.org/searches/Phat.cgi?id=MP:0002619) | MP:0002619 |
|  |  | [abnormal immune system organ morphology](http://www.informatics.jax.org/searches/Phat.cgi?id=MP:0002722) | MP:0002722 |
|  |  | [colitis](http://www.informatics.jax.org/searches/Phat.cgi?id=MP:0002816) | MP:0002816 |
|  |  | [intestinal adenocarcinoma](http://www.informatics.jax.org/searches/Phat.cgi?id=MP:0002957) | MP:0002957 |
|  |  | [increased pre-B cell number](http://www.informatics.jax.org/searches/Phat.cgi?id=MP:0003132) | MP:0003132 |
|  |  | [rectal hemorrhage](http://www.informatics.jax.org/searches/Phat.cgi?id=MP:0003293) | MP:0003293 |
|  |  | [abnormal intestinal goblet cells](http://www.informatics.jax.org/searches/Phat.cgi?id=MP:0003449) | MP:0003449 |
|  |  | [abnormal thymocyte activation](http://www.informatics.jax.org/searches/Phat.cgi?id=MP:0003850) | MP:0003850 |
|  |  | [abnormal lymphocyte physiology](http://www.informatics.jax.org/searches/Phat.cgi?id=MP:0003945) | MP:0003945 |
|  |  | [increased length of allograft survival](http://www.informatics.jax.org/searches/Phat.cgi?id=MP:0004751) | MP:0004751 |
|  |  | [abnormal class switch recombination](http://www.informatics.jax.org/searches/Phat.cgi?id=MP:0004816) | MP:0004816 |
|  |  | [increased susceptibility to autoimmune hemolytic anemia](http://www.informatics.jax.org/searches/Phat.cgi?id=MP:0004827) | MP:0004827 |
|  |  | [abnormal regulatory T cell physiology](http://www.informatics.jax.org/searches/Phat.cgi?id=MP:0004946) | MP:0004946 |
|  |  | [increased regulatory T cell number](http://www.informatics.jax.org/searches/Phat.cgi?id=MP:0004973) | MP:0004973 |
|  |  | [decreased regulatory T cell number](http://www.informatics.jax.org/searches/Phat.cgi?id=MP:0004974) | MP:0004974 |
|  |  | [abnormal CD8-positive T cell morphology](http://www.informatics.jax.org/searches/Phat.cgi?id=MP:0005010) | MP:0005010 |
|  |  | [increased T cell number](http://www.informatics.jax.org/searches/Phat.cgi?id=MP:0005015) | MP:0005015 |
|  |  | [diarrhea](http://www.informatics.jax.org/searches/Phat.cgi?id=MP:0005036) | MP:0005036 |
|  |  | [abnormal level of surface class II molecules](http://www.informatics.jax.org/searches/Phat.cgi?id=MP:0005042) | MP:0005042 |
|  |  | [impaired NK cell cytolysis](http://www.informatics.jax.org/searches/Phat.cgi?id=MP:0005070) | MP:0005070 |
|  |  | [abnormal cytotoxic T cell physiology](http://www.informatics.jax.org/searches/Phat.cgi?id=MP:0005078) | MP:0005078 |
|  |  | [defective cytotoxic T cell cytolysis](http://www.informatics.jax.org/searches/Phat.cgi?id=MP:0005079) | MP:0005079 |
|  |  | [decreased double-negative T cell number](http://www.informatics.jax.org/searches/Phat.cgi?id=MP:0005089) | MP:0005089 |
|  |  | [increased double-negative T cell number](http://www.informatics.jax.org/searches/Phat.cgi?id=MP:0005090) | MP:0005090 |
|  |  | [decreased double-positive T cell number](http://www.informatics.jax.org/searches/Phat.cgi?id=MP:0005092) | MP:0005092 |
|  |  | [decreased T cell proliferation](http://www.informatics.jax.org/searches/Phat.cgi?id=MP:0005095) | MP:0005095 |
|  |  | [cachexia](http://www.informatics.jax.org/searches/Phat.cgi?id=MP:0005150) | MP:0005150 |
|  |  | [increased B cell proliferation](http://www.informatics.jax.org/searches/Phat.cgi?id=MP:0005154) | MP:0005154 |
|  |  | [increased circulating bilirubin level](http://www.informatics.jax.org/searches/Phat.cgi?id=MP:0005344) | MP:0005344 |
|  |  | [increased T cell proliferation](http://www.informatics.jax.org/searches/Phat.cgi?id=MP:0005348) | MP:0005348 |
|  |  | [increased susceptibility to autoimmune disorder](http://www.informatics.jax.org/searches/Phat.cgi?id=MP:0005350) | MP:0005350 |
|  |  | [abnormal CD4-positive T cell physiology](http://www.informatics.jax.org/searches/Phat.cgi?id=MP:0005463) | MP:0005463 |
|  |  | [increased mean corpuscular hemoglobin](http://www.informatics.jax.org/searches/Phat.cgi?id=MP:0005561) | MP:0005561 |
|  |  | [abnormal response to transplant](http://www.informatics.jax.org/searches/Phat.cgi?id=MP:0005671) | MP:0005671 |
|  |  | [increased CD4-positive T cell number](http://www.informatics.jax.org/searches/Phat.cgi?id=MP:0008074) | MP:0008074 |
|  |  | [abnormal CD4-positive T cell differentiation](http://www.informatics.jax.org/searches/Phat.cgi?id=MP:0008076) | MP:0008076 |
|  |  | [increased CD8-positive T cell number](http://www.informatics.jax.org/searches/Phat.cgi?id=MP:0008078) | MP:0008078 |
|  |  | [increased single-positive T cell number](http://www.informatics.jax.org/searches/Phat.cgi?id=MP:0008082) | MP:0008082 |
|  |  | [abnormal T-helper 1 cell differentiation](http://www.informatics.jax.org/searches/Phat.cgi?id=MP:0008088) | MP:0008088 |
|  |  | [increased plasma cell number](http://www.informatics.jax.org/searches/Phat.cgi?id=MP:0008097) | MP:0008097 |
|  |  | [lymph node hyperplasia](http://www.informatics.jax.org/searches/Phat.cgi?id=MP:0008102) | MP:0008102 |
|  |  | [increased germinal center B cell number](http://www.informatics.jax.org/searches/Phat.cgi?id=MP:0008177) | MP:0008177 |
|  |  | [increased pro-B cell number](http://www.informatics.jax.org/searches/Phat.cgi?id=MP:0008186) | MP:0008186 |
|  |  | [abnormal gamma-delta intraepithelial T cell morphology](http://www.informatics.jax.org/searches/Phat.cgi?id=MP:0008349) | MP:0008349 |
|  |  | [abnormal CD8 positive, alpha-beta intraepithelial T cell morphology](http://www.informatics.jax.org/searches/Phat.cgi?id=MP:0008401) | MP:0008401 |
|  |  | [enlarged mesenteric lymph nodes](http://www.informatics.jax.org/searches/Phat.cgi?id=MP:0008466) | MP:0008466 |
|  |  | [increased spleen red pulp amount](http://www.informatics.jax.org/searches/Phat.cgi?id=MP:0008476) | MP:0008476 |
|  |  | [increased IgG1 level](http://www.informatics.jax.org/searches/Phat.cgi?id=MP:0008499) | MP:0008499 |
|  |  | [increased IgG2a level](http://www.informatics.jax.org/searches/Phat.cgi?id=MP:0008500) | MP:0008500 |
|  |  | [increased IgG2b level](http://www.informatics.jax.org/searches/Phat.cgi?id=MP:0008501) | MP:0008501 |
| [Il2ra](http://www.informatics.jax.org/javawi2/servlet/WIFetch?page=markerDetail&id=MGI:96549) | MGI:1857192 | [decreased hematocrit](http://www.informatics.jax.org/searches/Phat.cgi?id=MP:0000208) | MP:0000208 |
|  |  | [lymphoid hyperplasia](http://www.informatics.jax.org/searches/Phat.cgi?id=MP:0000688) | MP:0000688 |
|  |  | [enlarged spleen](http://www.informatics.jax.org/searches/Phat.cgi?id=MP:0000691) | MP:0000691 |
|  |  | [enlarged lymph nodes](http://www.informatics.jax.org/searches/Phat.cgi?id=MP:0000702) | MP:0000702 |
|  |  | [decreased body size](http://www.informatics.jax.org/searches/Phat.cgi?id=MP:0001265) | MP:0001265 |
|  |  | [anemia](http://www.informatics.jax.org/searches/Phat.cgi?id=MP:0001577) | MP:0001577 |
|  |  | [hemolytic anemia](http://www.informatics.jax.org/searches/Phat.cgi?id=MP:0001585) | MP:0001585 |
|  |  | [abnormal induced morbidity/mortality](http://www.informatics.jax.org/searches/Phat.cgi?id=MP:0001657) | MP:0001657 |
|  |  | [abnormal T cell activation](http://www.informatics.jax.org/searches/Phat.cgi?id=MP:0001828) | MP:0001828 |
|  |  | [increased activated T cell number](http://www.informatics.jax.org/searches/Phat.cgi?id=MP:0001829) | MP:0001829 |
|  |  | [premature death](http://www.informatics.jax.org/searches/Phat.cgi?id=MP:0002083) | MP:0002083 |
|  |  | [abnormal lymph organ size](http://www.informatics.jax.org/searches/Phat.cgi?id=MP:0002221) | MP:0002221 |
|  |  | [abnormal T cell physiology](http://www.informatics.jax.org/searches/Phat.cgi?id=MP:0002444) | MP:0002444 |
|  |  | [abnormal B cell physiology](http://www.informatics.jax.org/searches/Phat.cgi?id=MP:0002459) | MP:0002459 |
|  |  | [increased IgG level](http://www.informatics.jax.org/searches/Phat.cgi?id=MP:0002493) | MP:0002493 |
|  |  | [increased IgA level](http://www.informatics.jax.org/searches/Phat.cgi?id=MP:0002495) | MP:0002495 |
|  |  | [colitis](http://www.informatics.jax.org/searches/Phat.cgi?id=MP:0002816) | MP:0002816 |
|  |  | [abnormal regulatory T cell physiology](http://www.informatics.jax.org/searches/Phat.cgi?id=MP:0004946) | MP:0004946 |
|  |  | [increased regulatory T cell number](http://www.informatics.jax.org/searches/Phat.cgi?id=MP:0004973) | MP:0004973 |
|  |  | [decreased regulatory T cell number](http://www.informatics.jax.org/searches/Phat.cgi?id=MP:0004974) | MP:0004974 |
|  |  | [increased lymphocyte cell number](http://www.informatics.jax.org/searches/Phat.cgi?id=MP:0005013) | MP:0005013 |
|  |  | [diarrhea](http://www.informatics.jax.org/searches/Phat.cgi?id=MP:0005036) | MP:0005036 |
|  |  | [cachexia](http://www.informatics.jax.org/searches/Phat.cgi?id=MP:0005150) | MP:0005150 |
|  |  | [abnormal T cell morphology](http://www.informatics.jax.org/searches/Phat.cgi?id=MP:0008037) | MP:0008037 |
|  |  | [increased memory T cell number](http://www.informatics.jax.org/searches/Phat.cgi?id=MP:0008049) | MP:0008049 |
|  |  | [increased IgG1 level](http://www.informatics.jax.org/searches/Phat.cgi?id=MP:0008499) | MP:0008499 |
|  |  | [increased IgG2a level](http://www.informatics.jax.org/searches/Phat.cgi?id=MP:0008500) | MP:0008500 |
|  |  | [increased IgG2b level](http://www.informatics.jax.org/searches/Phat.cgi?id=MP:0008501) | MP:0008501 |
|  | MGI:4399557 |  |  |
| [Il5](http://www.informatics.jax.org/javawi2/servlet/WIFetch?page=markerDetail&id=MGI:96557) | MGI:1861948 | [increased neutrophil cell number](http://www.informatics.jax.org/searches/Phat.cgi?id=MP:0000219) | MP:0000219 |
|  |  | [increased body weight](http://www.informatics.jax.org/searches/Phat.cgi?id=MP:0001260) | MP:0001260 |
|  |  | [abnormal mating receptivity](http://www.informatics.jax.org/searches/Phat.cgi?id=MP:0001376) | MP:0001376 |
|  |  | [decreased airway responsiveness](http://www.informatics.jax.org/searches/Phat.cgi?id=MP:0002335) | MP:0002335 |
|  |  | [granulomatous inflammation](http://www.informatics.jax.org/searches/Phat.cgi?id=MP:0002500) | MP:0002500 |
|  |  | [liver fibrosis](http://www.informatics.jax.org/searches/Phat.cgi?id=MP:0003333) | MP:0003333 |
|  |  | [enlarged placenta](http://www.informatics.jax.org/searches/Phat.cgi?id=MP:0004260) | MP:0004260 |
|  |  | [increased eosinophil cell number](http://www.informatics.jax.org/searches/Phat.cgi?id=MP:0005011) | MP:0005011 |
|  |  | [increased lymphocyte cell number](http://www.informatics.jax.org/searches/Phat.cgi?id=MP:0005013) | MP:0005013 |
|  |  | [increased interferon-gamma secretion](http://www.informatics.jax.org/searches/Phat.cgi?id=MP:0008566) | MP:0008566 |
|  |  | [prolonged estrous cycle](http://www.informatics.jax.org/searches/Phat.cgi?id=MP:0009006) | MP:0009006 |
|  | MGI:3688245 | [abnormal mast cell morphology](http://www.informatics.jax.org/searches/Phat.cgi?id=MP:0000359) | MP:0000359 |
|  |  | [altered susceptibility to infection](http://www.informatics.jax.org/searches/Phat.cgi?id=MP:0001793) | MP:0001793 |
|  |  | [abnormal humoral immune response](http://www.informatics.jax.org/searches/Phat.cgi?id=MP:0001800) | MP:0001800 |
|  |  | [abnormal mast cell physiology](http://www.informatics.jax.org/searches/Phat.cgi?id=MP:0002423) | MP:0002423 |
|  |  | [decreased IgE level](http://www.informatics.jax.org/searches/Phat.cgi?id=MP:0002492) | MP:0002492 |
|  |  | [granulomatous inflammation](http://www.informatics.jax.org/searches/Phat.cgi?id=MP:0002500) | MP:0002500 |
|  |  | [abnormal intestinal goblet cells](http://www.informatics.jax.org/searches/Phat.cgi?id=MP:0003449) | MP:0003449 |
|  |  | [decreased eosinophil cell number](http://www.informatics.jax.org/searches/Phat.cgi?id=MP:0005012) | MP:0005012 |
|  |  | [increased susceptibility to parasitic infection](http://www.informatics.jax.org/searches/Phat.cgi?id=MP:0005027) | MP:0005027 |
|  |  | [abnormal interleukin level](http://www.informatics.jax.org/searches/Phat.cgi?id=MP:0008751) | MP:0008751 |
|  | MGI:3689810 | [granulomatous inflammation](http://www.informatics.jax.org/searches/Phat.cgi?id=MP:0002500) | MP:0002500 |
|  |  | [increased susceptibility to parasitic infection](http://www.informatics.jax.org/searches/Phat.cgi?id=MP:0005027) | MP:0005027 |
|  | MGI:3689812 | [altered susceptibility to infection](http://www.informatics.jax.org/searches/Phat.cgi?id=MP:0001793) | MP:0001793 |
|  |  | [abnormal immunoglobulin level](http://www.informatics.jax.org/searches/Phat.cgi?id=MP:0002490) | MP:0002490 |
|  |  | [granulomatous inflammation](http://www.informatics.jax.org/searches/Phat.cgi?id=MP:0002500) | MP:0002500 |
|  |  | [abnormal intestinal goblet cells](http://www.informatics.jax.org/searches/Phat.cgi?id=MP:0003449) | MP:0003449 |
|  |  | [increased eosinophil cell number](http://www.informatics.jax.org/searches/Phat.cgi?id=MP:0005011) | MP:0005011 |
|  |  | [increased susceptibility to parasitic infection](http://www.informatics.jax.org/searches/Phat.cgi?id=MP:0005027) | MP:0005027 |
|  |  | [abnormal interleukin level](http://www.informatics.jax.org/searches/Phat.cgi?id=MP:0008751) | MP:0008751 |
|  | MGI:4399459 |  |  |
| [Lcat](http://www.informatics.jax.org/javawi2/servlet/WIFetch?page=markerDetail&id=MGI:96755) | MGI:2180695 | [decreased circulating LDL cholesterol level](http://www.informatics.jax.org/searches/Phat.cgi?id=MP:0000183) | MP:0000183 |
|  |  | [decreased circulating HDL cholesterol level](http://www.informatics.jax.org/searches/Phat.cgi?id=MP:0000186) | MP:0000186 |
|  |  | [enlarged liver](http://www.informatics.jax.org/searches/Phat.cgi?id=MP:0000599) | MP:0000599 |
|  |  | [abnormal adrenal gland morphology](http://www.informatics.jax.org/searches/Phat.cgi?id=MP:0000639) | MP:0000639 |
|  |  | [abnormal lipid level](http://www.informatics.jax.org/searches/Phat.cgi?id=MP:0001547) | MP:0001547 |
|  |  | [increased circulating triglyceride level](http://www.informatics.jax.org/searches/Phat.cgi?id=MP:0001552) | MP:0001552 |
|  |  | [abnormal lipid homeostasis](http://www.informatics.jax.org/searches/Phat.cgi?id=MP:0002118) | MP:0002118 |
|  |  | [decreased circulating triglyceride level](http://www.informatics.jax.org/searches/Phat.cgi?id=MP:0002644) | MP:0002644 |
|  |  | [abnormal renal tubule morphology](http://www.informatics.jax.org/searches/Phat.cgi?id=MP:0002703) | MP:0002703 |
|  |  | [decreased circulating insulin level](http://www.informatics.jax.org/searches/Phat.cgi?id=MP:0002727) | MP:0002727 |
|  |  | [albuminuria](http://www.informatics.jax.org/searches/Phat.cgi?id=MP:0002871) | MP:0002871 |
|  |  | [increased circulating phospholipid level](http://www.informatics.jax.org/searches/Phat.cgi?id=MP:0003980) | MP:0003980 |
|  |  | [increased cholesterol level](http://www.informatics.jax.org/searches/Phat.cgi?id=MP:0003982) | MP:0003982 |
|  |  | [decreased cholesterol level](http://www.informatics.jax.org/searches/Phat.cgi?id=MP:0003983) | MP:0003983 |
|  |  | [abnormal proximal convoluted tubule morphology](http://www.informatics.jax.org/searches/Phat.cgi?id=MP:0004756) | MP:0004756 |
|  |  | [increased circulating VLDL cholesterol level](http://www.informatics.jax.org/searches/Phat.cgi?id=MP:0005145) | MP:0005145 |
|  |  | [increased circulating cholesterol level](http://www.informatics.jax.org/searches/Phat.cgi?id=MP:0005178) | MP:0005178 |
|  |  | [decreased circulating cholesterol level](http://www.informatics.jax.org/searches/Phat.cgi?id=MP:0005179) | MP:0005179 |
|  |  | [glomerulosclerosis](http://www.informatics.jax.org/searches/Phat.cgi?id=MP:0005264) | MP:0005264 |
|  |  | [abnormal cholesterol homeostasis](http://www.informatics.jax.org/searches/Phat.cgi?id=MP:0005278) | MP:0005278 |
|  |  | [abnormal renal glomerulus morphology](http://www.informatics.jax.org/searches/Phat.cgi?id=MP:0005325) | MP:0005325 |
|  |  | [abnormal podocyte](http://www.informatics.jax.org/searches/Phat.cgi?id=MP:0005326) | MP:0005326 |
|  |  | [abnormal mesangial cell](http://www.informatics.jax.org/searches/Phat.cgi?id=MP:0005327) | MP:0005327 |
|  |  | [abnormal circulating protein level](http://www.informatics.jax.org/searches/Phat.cgi?id=MP:0005416) | MP:0005416 |
|  |  | [decreased circulating glucose level](http://www.informatics.jax.org/searches/Phat.cgi?id=MP:0005560) | MP:0005560 |
|  |  | [abnormal enzyme/coenzyme activity](http://www.informatics.jax.org/searches/Phat.cgi?id=MP:0005584) | MP:0005584 |
|  |  | [podocyte foot process effacement](http://www.informatics.jax.org/searches/Phat.cgi?id=MP:0008140) | MP:0008140 |
|  | MGI:2445879 | [decreased circulating HDL cholesterol level](http://www.informatics.jax.org/searches/Phat.cgi?id=MP:0000186) | MP:0000186 |
|  |  | [abnormal lipid level](http://www.informatics.jax.org/searches/Phat.cgi?id=MP:0001547) | MP:0001547 |
|  |  | [increased circulating triglyceride level](http://www.informatics.jax.org/searches/Phat.cgi?id=MP:0001552) | MP:0001552 |
|  |  | [hemolytic anemia](http://www.informatics.jax.org/searches/Phat.cgi?id=MP:0001585) | MP:0001585 |
|  |  | [reticulocytosis](http://www.informatics.jax.org/searches/Phat.cgi?id=MP:0002640) | MP:0002640 |
|  |  | [abnormal erythrocyte lysis](http://www.informatics.jax.org/searches/Phat.cgi?id=MP:0003657) | MP:0003657 |
|  |  | [decreased circulating VLDL cholesterol level](http://www.informatics.jax.org/searches/Phat.cgi?id=MP:0005146) | MP:0005146 |
|  |  | [proteinuria](http://www.informatics.jax.org/searches/Phat.cgi?id=MP:0005160) | MP:0005160 |
|  |  | [decreased circulating cholesterol level](http://www.informatics.jax.org/searches/Phat.cgi?id=MP:0005179) | MP:0005179 |
|  |  | [glomerulosclerosis](http://www.informatics.jax.org/searches/Phat.cgi?id=MP:0005264) | MP:0005264 |
|  |  | [abnormal cholesterol homeostasis](http://www.informatics.jax.org/searches/Phat.cgi?id=MP:0005278) | MP:0005278 |
|  |  | [abnormal renal glomerulus morphology](http://www.informatics.jax.org/searches/Phat.cgi?id=MP:0005325) | MP:0005325 |
|  |  | [abnormal mesangial cell](http://www.informatics.jax.org/searches/Phat.cgi?id=MP:0005327) | MP:0005327 |
|  |  | [abnormal circulating phospholipid level](http://www.informatics.jax.org/searches/Phat.cgi?id=MP:0006084) | MP:0006084 |
|  | MGI:4399266 |  |  |
| [Ldlr](http://www.informatics.jax.org/javawi2/servlet/WIFetch?page=markerDetail&id=MGI:96765) | MGI:1857212 | [abnormal microglial cell morphology](http://www.informatics.jax.org/searches/Phat.cgi?id=MP:0000136) | MP:0000136 |
|  |  | [abnormal circulating cholesterol level](http://www.informatics.jax.org/searches/Phat.cgi?id=MP:0000180) | MP:0000180 |
|  |  | [abnormal circulating LDL cholesterol level](http://www.informatics.jax.org/searches/Phat.cgi?id=MP:0000181) | MP:0000181 |
|  |  | [increased circulating LDL cholesterol level](http://www.informatics.jax.org/searches/Phat.cgi?id=MP:0000182) | MP:0000182 |
|  |  | [decreased circulating LDL cholesterol level](http://www.informatics.jax.org/searches/Phat.cgi?id=MP:0000183) | MP:0000183 |
|  |  | [decreased circulating HDL cholesterol level](http://www.informatics.jax.org/searches/Phat.cgi?id=MP:0000186) | MP:0000186 |
|  |  | [abnormal triglyceride level](http://www.informatics.jax.org/searches/Phat.cgi?id=MP:0000187) | MP:0000187 |
|  |  | [increased neutrophil cell number](http://www.informatics.jax.org/searches/Phat.cgi?id=MP:0000219) | MP:0000219 |
|  |  | [increased monocyte cell number](http://www.informatics.jax.org/searches/Phat.cgi?id=MP:0000220) | MP:0000220 |
|  |  | [altered response to myocardial infarction](http://www.informatics.jax.org/searches/Phat.cgi?id=MP:0000343) | MP:0000343 |
|  |  | [alopecia](http://www.informatics.jax.org/searches/Phat.cgi?id=MP:0000414) | MP:0000414 |
|  |  | [abnormal liver morphology](http://www.informatics.jax.org/searches/Phat.cgi?id=MP:0000598) | MP:0000598 |
|  |  | [amyloidosis](http://www.informatics.jax.org/searches/Phat.cgi?id=MP:0000604) | MP:0000604 |
|  |  | [abnormal liver physiology](http://www.informatics.jax.org/searches/Phat.cgi?id=MP:0000609) | MP:0000609 |
|  |  | [abnormal adrenal gland morphology](http://www.informatics.jax.org/searches/Phat.cgi?id=MP:0000639) | MP:0000639 |
|  |  | [decreased corpus callosum size](http://www.informatics.jax.org/searches/Phat.cgi?id=MP:0000781) | MP:0000781 |
|  |  | [abnormal hippocampus morphology](http://www.informatics.jax.org/searches/Phat.cgi?id=MP:0000807) | MP:0000807 |
|  |  | [abnormal cerebellum morphology](http://www.informatics.jax.org/searches/Phat.cgi?id=MP:0000849) | MP:0000849 |
|  |  | [abnormal Purkinje cell morphology](http://www.informatics.jax.org/searches/Phat.cgi?id=MP:0000877) | MP:0000877 |
|  |  | [decreased Purkinje cell number](http://www.informatics.jax.org/searches/Phat.cgi?id=MP:0000880) | MP:0000880 |
|  |  | [abnormal CNS glial cell morphology](http://www.informatics.jax.org/searches/Phat.cgi?id=MP:0000952) | MP:0000952 |
|  |  | [scaly skin](http://www.informatics.jax.org/searches/Phat.cgi?id=MP:0001192) | MP:0001192 |
|  |  | [thick skin](http://www.informatics.jax.org/searches/Phat.cgi?id=MP:0001200) | MP:0001200 |
|  |  | [spontaneous skin ulceration](http://www.informatics.jax.org/searches/Phat.cgi?id=MP:0001209) | MP:0001209 |
|  |  | [skin lesions](http://www.informatics.jax.org/searches/Phat.cgi?id=MP:0001212) | MP:0001212 |
|  |  | [abnormal dermal layer morphology](http://www.informatics.jax.org/searches/Phat.cgi?id=MP:0001243) | MP:0001243 |
|  |  | [thick dermal layer](http://www.informatics.jax.org/searches/Phat.cgi?id=MP:0001245) | MP:0001245 |
|  |  | [decreased body weight](http://www.informatics.jax.org/searches/Phat.cgi?id=MP:0001262) | MP:0001262 |
|  |  | [dry eyes](http://www.informatics.jax.org/searches/Phat.cgi?id=MP:0001337) | MP:0001337 |
|  |  | [meibomian gland atrophy](http://www.informatics.jax.org/searches/Phat.cgi?id=MP:0001345) | MP:0001345 |
|  |  | [decreased anxiety-related response](http://www.informatics.jax.org/searches/Phat.cgi?id=MP:0001364) | MP:0001364 |
|  |  | [hyperactivity](http://www.informatics.jax.org/searches/Phat.cgi?id=MP:0001399) | MP:0001399 |
|  |  | [hypoactivity](http://www.informatics.jax.org/searches/Phat.cgi?id=MP:0001402) | MP:0001402 |
|  |  | [abnormal spatial learning](http://www.informatics.jax.org/searches/Phat.cgi?id=MP:0001463) | MP:0001463 |
|  |  | [abnormal lipid level](http://www.informatics.jax.org/searches/Phat.cgi?id=MP:0001547) | MP:0001547 |
|  |  | [hyperlipidemia](http://www.informatics.jax.org/searches/Phat.cgi?id=MP:0001548) | MP:0001548 |
|  |  | [increased circulating triglyceride level](http://www.informatics.jax.org/searches/Phat.cgi?id=MP:0001552) | MP:0001552 |
|  |  | [increased circulating HDL cholesterol level](http://www.informatics.jax.org/searches/Phat.cgi?id=MP:0001556) | MP:0001556 |
|  |  | [hyperglycemia](http://www.informatics.jax.org/searches/Phat.cgi?id=MP:0001559) | MP:0001559 |
|  |  | [skin edema](http://www.informatics.jax.org/searches/Phat.cgi?id=MP:0001786) | MP:0001786 |
|  |  | [decreased IgG level](http://www.informatics.jax.org/searches/Phat.cgi?id=MP:0001805) | MP:0001805 |
|  |  | [abnormal glucose homeostasis](http://www.informatics.jax.org/searches/Phat.cgi?id=MP:0002078) | MP:0002078 |
|  |  | [increased circulating insulin level](http://www.informatics.jax.org/searches/Phat.cgi?id=MP:0002079) | MP:0002079 |
|  |  | [prenatal lethality](http://www.informatics.jax.org/searches/Phat.cgi?id=MP:0002080) | MP:0002080 |
|  |  | [postnatal lethality](http://www.informatics.jax.org/searches/Phat.cgi?id=MP:0002082) | MP:0002082 |
|  |  | [premature death](http://www.informatics.jax.org/searches/Phat.cgi?id=MP:0002083) | MP:0002083 |
|  |  | [abnormal lipid homeostasis](http://www.informatics.jax.org/searches/Phat.cgi?id=MP:0002118) | MP:0002118 |
|  |  | [abnormal brain morphology](http://www.informatics.jax.org/searches/Phat.cgi?id=MP:0002152) | MP:0002152 |
|  |  | [abnormal astrocyte morphology](http://www.informatics.jax.org/searches/Phat.cgi?id=MP:0002182) | MP:0002182 |
|  |  | [abnormal mononuclear leukocyte differentiation](http://www.informatics.jax.org/searches/Phat.cgi?id=MP:0002445) | MP:0002445 |
|  |  | [abnormal macrophage physiology](http://www.informatics.jax.org/searches/Phat.cgi?id=MP:0002451) | MP:0002451 |
|  |  | [increased IgM level](http://www.informatics.jax.org/searches/Phat.cgi?id=MP:0002494) | MP:0002494 |
|  |  | [abnormal blood coagulation](http://www.informatics.jax.org/searches/Phat.cgi?id=MP:0002551) | MP:0002551 |
|  |  | [hepatic steatosis](http://www.informatics.jax.org/searches/Phat.cgi?id=MP:0002628) | MP:0002628 |
|  |  | [decreased circulating triglyceride level](http://www.informatics.jax.org/searches/Phat.cgi?id=MP:0002644) | MP:0002644 |
|  |  | [decreased circulating corticosterone level](http://www.informatics.jax.org/searches/Phat.cgi?id=MP:0002665) | MP:0002665 |
|  |  | [decreased circulating insulin level](http://www.informatics.jax.org/searches/Phat.cgi?id=MP:0002727) | MP:0002727 |
|  |  | [increased liver weight](http://www.informatics.jax.org/searches/Phat.cgi?id=MP:0002981) | MP:0002981 |
|  |  | [decreased infarction size](http://www.informatics.jax.org/searches/Phat.cgi?id=MP:0003038) | MP:0003038 |
|  |  | [abnormal impulse conducting system conduction](http://www.informatics.jax.org/searches/Phat.cgi?id=MP:0003137) | MP:0003137 |
|  |  | [amyloid beta deposits](http://www.informatics.jax.org/searches/Phat.cgi?id=MP:0003329) | MP:0003329 |
|  |  | [abnormal response/metabolism to endogenous compounds](http://www.informatics.jax.org/searches/Phat.cgi?id=MP:0003638) | MP:0003638 |
|  |  | [xanthoma](http://www.informatics.jax.org/searches/Phat.cgi?id=MP:0003692) | MP:0003692 |
|  |  | [maternal effect](http://www.informatics.jax.org/searches/Phat.cgi?id=MP:0003718) | MP:0003718 |
|  |  | [decreased macrophage cell number](http://www.informatics.jax.org/searches/Phat.cgi?id=MP:0003884) | MP:0003884 |
|  |  | [abnormal ST interval](http://www.informatics.jax.org/searches/Phat.cgi?id=MP:0003897) | MP:0003897 |
|  |  | [increased eating behavior](http://www.informatics.jax.org/searches/Phat.cgi?id=MP:0003909) | MP:0003909 |
|  |  | [decreased lean body mass](http://www.informatics.jax.org/searches/Phat.cgi?id=MP:0003961) | MP:0003961 |
|  |  | [increased circulating VLDL triglyceride level](http://www.informatics.jax.org/searches/Phat.cgi?id=MP:0003975) | MP:0003975 |
|  |  | [decreased circulating VLDL triglyceride level](http://www.informatics.jax.org/searches/Phat.cgi?id=MP:0003976) | MP:0003976 |
|  |  | [increased cholesterol level](http://www.informatics.jax.org/searches/Phat.cgi?id=MP:0003982) | MP:0003982 |
|  |  | [decreased cholesterol level](http://www.informatics.jax.org/searches/Phat.cgi?id=MP:0003983) | MP:0003983 |
|  |  | [arteriosclerosis](http://www.informatics.jax.org/searches/Phat.cgi?id=MP:0003991) | MP:0003991 |
|  |  | [abnormal arteriole morphology](http://www.informatics.jax.org/searches/Phat.cgi?id=MP:0004112) | MP:0004112 |
|  |  | [skin inflammation](http://www.informatics.jax.org/searches/Phat.cgi?id=MP:0004947) | MP:0004947 |
|  |  | [increased lymphocyte cell number](http://www.informatics.jax.org/searches/Phat.cgi?id=MP:0005013) | MP:0005013 |
|  |  | [thrombosis](http://www.informatics.jax.org/searches/Phat.cgi?id=MP:0005048) | MP:0005048 |
|  |  | [abnormal circulating VLDL cholesterol level](http://www.informatics.jax.org/searches/Phat.cgi?id=MP:0005144) | MP:0005144 |
|  |  | [increased circulating VLDL cholesterol level](http://www.informatics.jax.org/searches/Phat.cgi?id=MP:0005145) | MP:0005145 |
|  |  | [decreased circulating VLDL cholesterol level](http://www.informatics.jax.org/searches/Phat.cgi?id=MP:0005146) | MP:0005146 |
|  |  | [increased circulating cholesterol level](http://www.informatics.jax.org/searches/Phat.cgi?id=MP:0005178) | MP:0005178 |
|  |  | [decreased circulating cholesterol level](http://www.informatics.jax.org/searches/Phat.cgi?id=MP:0005179) | MP:0005179 |
|  |  | [abnormal retinal pigment epithelium morphology](http://www.informatics.jax.org/searches/Phat.cgi?id=MP:0005201) | MP:0005201 |
|  |  | [abnormal Bruch membrane morphology](http://www.informatics.jax.org/searches/Phat.cgi?id=MP:0005239) | MP:0005239 |
|  |  | [abnormal cholesterol homeostasis](http://www.informatics.jax.org/searches/Phat.cgi?id=MP:0005278) | MP:0005278 |
|  |  | [improved glucose tolerance](http://www.informatics.jax.org/searches/Phat.cgi?id=MP:0005292) | MP:0005292 |
|  |  | [impaired glucose tolerance](http://www.informatics.jax.org/searches/Phat.cgi?id=MP:0005293) | MP:0005293 |
|  |  | [abnormal circulating amino acid level](http://www.informatics.jax.org/searches/Phat.cgi?id=MP:0005311) | MP:0005311 |
|  |  | [decreased triglyceride level](http://www.informatics.jax.org/searches/Phat.cgi?id=MP:0005318) | MP:0005318 |
|  |  | [insulin resistance](http://www.informatics.jax.org/searches/Phat.cgi?id=MP:0005331) | MP:0005331 |
|  |  | [abnormal fat pad morphology](http://www.informatics.jax.org/searches/Phat.cgi?id=MP:0005334) | MP:0005334 |
|  |  | [atherosclerotic lesions](http://www.informatics.jax.org/searches/Phat.cgi?id=MP:0005338) | MP:0005338 |
|  |  | [increased susceptibility to atherosclerosis](http://www.informatics.jax.org/searches/Phat.cgi?id=MP:0005339) | MP:0005339 |
|  |  | [altered susceptibility to atherosclerosis](http://www.informatics.jax.org/searches/Phat.cgi?id=MP:0005340) | MP:0005340 |
|  |  | [decreased susceptibility to atherosclerosis](http://www.informatics.jax.org/searches/Phat.cgi?id=MP:0005341) | MP:0005341 |
|  |  | [abnormal circulating protein level](http://www.informatics.jax.org/searches/Phat.cgi?id=MP:0005416) | MP:0005416 |
|  |  | [abnormal food intake](http://www.informatics.jax.org/searches/Phat.cgi?id=MP:0005449) | MP:0005449 |
|  |  | [increased adipose tissue amount](http://www.informatics.jax.org/searches/Phat.cgi?id=MP:0005453) | MP:0005453 |
|  |  | [weight gain](http://www.informatics.jax.org/searches/Phat.cgi?id=MP:0005456) | MP:0005456 |
|  |  | [increased percent body fat](http://www.informatics.jax.org/searches/Phat.cgi?id=MP:0005458) | MP:0005458 |
|  |  | [decreased percent body fat](http://www.informatics.jax.org/searches/Phat.cgi?id=MP:0005459) | MP:0005459 |
|  |  | [impaired macrophage recruitment](http://www.informatics.jax.org/searches/Phat.cgi?id=MP:0005496) | MP:0005496 |
|  |  | [abnormal eye electrophysiology](http://www.informatics.jax.org/searches/Phat.cgi?id=MP:0005551) | MP:0005551 |
|  |  | [increased circulating glucose level](http://www.informatics.jax.org/searches/Phat.cgi?id=MP:0005559) | MP:0005559 |
|  |  | [decreased circulating glucose level](http://www.informatics.jax.org/searches/Phat.cgi?id=MP:0005560) | MP:0005560 |
|  |  | [abnormal enzyme/coenzyme activity](http://www.informatics.jax.org/searches/Phat.cgi?id=MP:0005584) | MP:0005584 |
|  |  | [increased resistance to diet-induced obesity](http://www.informatics.jax.org/searches/Phat.cgi?id=MP:0005659) | MP:0005659 |
|  |  | [increased apoptosis](http://www.informatics.jax.org/searches/Phat.cgi?id=MP:0006042) | MP:0006042 |
|  |  | [abnormal choriocapillaris morphology](http://www.informatics.jax.org/searches/Phat.cgi?id=MP:0006238) | MP:0006238 |
|  |  | [abnormal epididymal fat pad morphology](http://www.informatics.jax.org/searches/Phat.cgi?id=MP:0006319) | MP:0006319 |
|  |  | [abnormal macrophage derived foam cell morphology](http://www.informatics.jax.org/searches/Phat.cgi?id=MP:0008243) | MP:0008243 |
|  |  | [abnormal zona fasciculata morphology](http://www.informatics.jax.org/searches/Phat.cgi?id=MP:0008294) | MP:0008294 |
|  |  | [abnormal spatial working memory](http://www.informatics.jax.org/searches/Phat.cgi?id=MP:0008428) | MP:0008428 |
|  |  | [abnormal short term spatial reference memory](http://www.informatics.jax.org/searches/Phat.cgi?id=MP:0008431) | MP:0008431 |
|  |  | [increased circulating tumor necrosis factor level](http://www.informatics.jax.org/searches/Phat.cgi?id=MP:0008553) | MP:0008553 |
|  |  | [decreased interferon-gamma secretion](http://www.informatics.jax.org/searches/Phat.cgi?id=MP:0008567) | MP:0008567 |
|  |  | [abnormal interleukin secretion](http://www.informatics.jax.org/searches/Phat.cgi?id=MP:0008568) | MP:0008568 |
|  |  | [abnormal synaptic bouton morphology](http://www.informatics.jax.org/searches/Phat.cgi?id=MP:0008571) | MP:0008571 |
|  |  | [photoreceptor outer segment degeneration](http://www.informatics.jax.org/searches/Phat.cgi?id=MP:0008584) | MP:0008584 |
|  |  | [increased circulating interleukin-10 level](http://www.informatics.jax.org/searches/Phat.cgi?id=MP:0008593) | MP:0008593 |
|  |  | [decreased circulating interleukin-10 level](http://www.informatics.jax.org/searches/Phat.cgi?id=MP:0008594) | MP:0008594 |
|  |  | [increased circulating interleukin-6 level](http://www.informatics.jax.org/searches/Phat.cgi?id=MP:0008596) | MP:0008596 |
|  |  | [increased circulating interleukin-1 beta level](http://www.informatics.jax.org/searches/Phat.cgi?id=MP:0008641) | MP:0008641 |
|  |  | [decreased survivor rate](http://www.informatics.jax.org/searches/Phat.cgi?id=MP:0008770) | MP:0008770 |
|  |  | [abnormal uterine fat pad morphology](http://www.informatics.jax.org/searches/Phat.cgi?id=MP:0008900) | MP:0008900 |
|  |  | [increased liver triglyceride level](http://www.informatics.jax.org/searches/Phat.cgi?id=MP:0009355) | MP:0009355 |
|  |  | [abnormal synapse morphology](http://www.informatics.jax.org/searches/Phat.cgi?id=MP:0009538) | MP:0009538 |
|  |  | [increased sensitivity to induced morbidity/mortality](http://www.informatics.jax.org/searches/Phat.cgi?id=MP:0009763) | MP:0009763 |
|  |  | [increased grip strength](http://www.informatics.jax.org/searches/Phat.cgi?id=MP:0010052) | MP:0010052 |
|  |  | [increased pruritus](http://www.informatics.jax.org/searches/Phat.cgi?id=MP:0010072) | MP:0010072 |
|  | MGI:4399561 |  |  |
| [Lpl](http://www.informatics.jax.org/javawi2/servlet/WIFetch?page=markerDetail&id=MGI:96820) | MGI:2651805 | [abnormal adipose tissue morphology](http://www.informatics.jax.org/searches/Phat.cgi?id=MP:0000003) | MP:0000003 |
|  |  | [decreased circulating LDL cholesterol level](http://www.informatics.jax.org/searches/Phat.cgi?id=MP:0000183) | MP:0000183 |
|  |  | [decreased circulating HDL cholesterol level](http://www.informatics.jax.org/searches/Phat.cgi?id=MP:0000186) | MP:0000186 |
|  |  | [abnormal liver morphology](http://www.informatics.jax.org/searches/Phat.cgi?id=MP:0000598) | MP:0000598 |
|  |  | [abnormal skeletal muscle morphology](http://www.informatics.jax.org/searches/Phat.cgi?id=MP:0000759) | MP:0000759 |
|  |  | [increased body weight](http://www.informatics.jax.org/searches/Phat.cgi?id=MP:0001260) | MP:0001260 |
|  |  | [increased circulating triglyceride level](http://www.informatics.jax.org/searches/Phat.cgi?id=MP:0001552) | MP:0001552 |
|  |  | [cyanosis](http://www.informatics.jax.org/searches/Phat.cgi?id=MP:0001575) | MP:0001575 |
|  |  | [neonatal lethality](http://www.informatics.jax.org/searches/Phat.cgi?id=MP:0002058) | MP:0002058 |
|  |  | [abnormal lipid homeostasis](http://www.informatics.jax.org/searches/Phat.cgi?id=MP:0002118) | MP:0002118 |
|  |  | [abnormal respiratory alveoli morphology](http://www.informatics.jax.org/searches/Phat.cgi?id=MP:0002270) | MP:0002270 |
|  |  | [abnormal capillary morphology](http://www.informatics.jax.org/searches/Phat.cgi?id=MP:0003658) | MP:0003658 |
|  |  | [pallor](http://www.informatics.jax.org/searches/Phat.cgi?id=MP:0003717) | MP:0003717 |
|  |  | [increased circulating VLDL triglyceride level](http://www.informatics.jax.org/searches/Phat.cgi?id=MP:0003975) | MP:0003975 |
|  |  | [increased cholesterol level](http://www.informatics.jax.org/searches/Phat.cgi?id=MP:0003982) | MP:0003982 |
|  |  | [abnormal lung vasculature morphology](http://www.informatics.jax.org/searches/Phat.cgi?id=MP:0004007) | MP:0004007 |
|  |  | [increased circulating VLDL cholesterol level](http://www.informatics.jax.org/searches/Phat.cgi?id=MP:0005145) | MP:0005145 |
|  |  | [increased circulating cholesterol level](http://www.informatics.jax.org/searches/Phat.cgi?id=MP:0005178) | MP:0005178 |
|  |  | [increased triglyceride level](http://www.informatics.jax.org/searches/Phat.cgi?id=MP:0005317) | MP:0005317 |
|  |  | [increased total fat pad weight](http://www.informatics.jax.org/searches/Phat.cgi?id=MP:0008908) | MP:0008908 |
|  | MGI:2429315 | [abnormal circulating cholesterol level](http://www.informatics.jax.org/searches/Phat.cgi?id=MP:0000180) | MP:0000180 |
|  |  | [decreased circulating HDL cholesterol level](http://www.informatics.jax.org/searches/Phat.cgi?id=MP:0000186) | MP:0000186 |
|  |  | [atelectasis](http://www.informatics.jax.org/searches/Phat.cgi?id=MP:0001177) | MP:0001177 |
|  |  | [hyperlipidemia](http://www.informatics.jax.org/searches/Phat.cgi?id=MP:0001548) | MP:0001548 |
|  |  | [increased circulating triglyceride level](http://www.informatics.jax.org/searches/Phat.cgi?id=MP:0001552) | MP:0001552 |
|  |  | [increased circulating HDL cholesterol level](http://www.informatics.jax.org/searches/Phat.cgi?id=MP:0001556) | MP:0001556 |
|  |  | [neonatal lethality](http://www.informatics.jax.org/searches/Phat.cgi?id=MP:0002058) | MP:0002058 |
|  |  | [abnormal lipid homeostasis](http://www.informatics.jax.org/searches/Phat.cgi?id=MP:0002118) | MP:0002118 |
|  |  | [abnormal circulating lipid level](http://www.informatics.jax.org/searches/Phat.cgi?id=MP:0003949) | MP:0003949 |
|  |  | [increased circulating VLDL triglyceride level](http://www.informatics.jax.org/searches/Phat.cgi?id=MP:0003975) | MP:0003975 |
|  |  | [increased circulating phospholipid level](http://www.informatics.jax.org/searches/Phat.cgi?id=MP:0003980) | MP:0003980 |
|  |  | [abnormal blood vessel healing](http://www.informatics.jax.org/searches/Phat.cgi?id=MP:0004883) | MP:0004883 |
|  |  | [thrombosis](http://www.informatics.jax.org/searches/Phat.cgi?id=MP:0005048) | MP:0005048 |
|  |  | [increased circulating cholesterol level](http://www.informatics.jax.org/searches/Phat.cgi?id=MP:0005178) | MP:0005178 |
|  |  | [lung vascular congestion](http://www.informatics.jax.org/searches/Phat.cgi?id=MP:0010018) | MP:0010018 |
|  |  | [liver vascular congestion](http://www.informatics.jax.org/searches/Phat.cgi?id=MP:0010019) | MP:0010019 |
| [Maf](http://www.informatics.jax.org/javawi2/servlet/WIFetch?page=markerDetail&id=MGI:96909) | MGI:2148636 | [decreased body size](http://www.informatics.jax.org/searches/Phat.cgi?id=MP:0001265) | MP:0001265 |
|  |  | [microphthalmia](http://www.informatics.jax.org/searches/Phat.cgi?id=MP:0001297) | MP:0001297 |
|  |  | [abnormal lens morphology](http://www.informatics.jax.org/searches/Phat.cgi?id=MP:0001303) | MP:0001303 |
|  |  | [perinatal lethality](http://www.informatics.jax.org/searches/Phat.cgi?id=MP:0002081) | MP:0002081 |
|  |  | [postnatal lethality](http://www.informatics.jax.org/searches/Phat.cgi?id=MP:0002082) | MP:0002082 |
|  |  | [abnormal lens development](http://www.informatics.jax.org/searches/Phat.cgi?id=MP:0005545) | MP:0005545 |
|  |  | [lethality throughout fetal growth and development](http://www.informatics.jax.org/searches/Phat.cgi?id=MP:0006208) | MP:0006208 |
|  |  | [decreased interleukin-4 secretion](http://www.informatics.jax.org/searches/Phat.cgi?id=MP:0008700) | MP:0008700 |
| [Kitl](http://www.informatics.jax.org/javawi2/servlet/WIFetch?page=markerDetail&id=MGI:96974) | MGI:4430868 | [decreased mast cell number](http://www.informatics.jax.org/searches/Phat.cgi?id=MP:0000336) | MP:0000336 |
|  |  | [belly spot](http://www.informatics.jax.org/searches/Phat.cgi?id=MP:0000373) | MP:0000373 |
|  |  | [decreased thymocyte number](http://www.informatics.jax.org/searches/Phat.cgi?id=MP:0000715) | MP:0000715 |
|  |  | [reduced male fertility](http://www.informatics.jax.org/searches/Phat.cgi?id=MP:0001922) | MP:0001922 |
|  |  | [increased mean corpuscular volume](http://www.informatics.jax.org/searches/Phat.cgi?id=MP:0002590) | MP:0002590 |
|  |  | [decreased erythrocyte cell number](http://www.informatics.jax.org/searches/Phat.cgi?id=MP:0002875) | MP:0002875 |
|  |  | [decreased testis weight](http://www.informatics.jax.org/searches/Phat.cgi?id=MP:0004852) | MP:0004852 |
|  |  | [decreased T cell number](http://www.informatics.jax.org/searches/Phat.cgi?id=MP:0005018) | MP:0005018 |
|  |  | [decreased double-negative T cell number](http://www.informatics.jax.org/searches/Phat.cgi?id=MP:0005089) | MP:0005089 |
|  |  | [decreased double-positive T cell number](http://www.informatics.jax.org/searches/Phat.cgi?id=MP:0005092) | MP:0005092 |
|  |  | [azoospermia](http://www.informatics.jax.org/searches/Phat.cgi?id=MP:0005159) | MP:0005159 |
|  |  | [absent coat pigmentation](http://www.informatics.jax.org/searches/Phat.cgi?id=MP:0005171) | MP:0005171 |
|  |  | [early reproductive senescence](http://www.informatics.jax.org/searches/Phat.cgi?id=MP:0008995) | MP:0008995 |
|  |  | [abnormal bone marrow cell physiology](http://www.informatics.jax.org/searches/Phat.cgi?id=MP:0009278) | MP:0009278 |
| [Nkx2-3](http://www.informatics.jax.org/javawi2/servlet/WIFetch?page=markerDetail&id=MGI:97348) | MGI:2386680 | [abnormal cell proliferation](http://www.informatics.jax.org/searches/Phat.cgi?id=MP:0000350) | MP:0000350 |
|  |  | [abnormal intestine morphology](http://www.informatics.jax.org/searches/Phat.cgi?id=MP:0000477) | MP:0000477 |
|  |  | [abnormal intestinal epithelium morphology](http://www.informatics.jax.org/searches/Phat.cgi?id=MP:0000488) | MP:0000488 |
|  |  | [abnormal crypts of Lieberkuhn morphology](http://www.informatics.jax.org/searches/Phat.cgi?id=MP:0000490) | MP:0000490 |
|  |  | [abnormal small intestine morphology](http://www.informatics.jax.org/searches/Phat.cgi?id=MP:0000496) | MP:0000496 |
|  |  | [abnormal spleen morphology](http://www.informatics.jax.org/searches/Phat.cgi?id=MP:0000689) | MP:0000689 |
|  |  | [absent spleen](http://www.informatics.jax.org/searches/Phat.cgi?id=MP:0000690) | MP:0000690 |
|  |  | [small spleen](http://www.informatics.jax.org/searches/Phat.cgi?id=MP:0000692) | MP:0000692 |
|  |  | [abnormal blood vessel morphology](http://www.informatics.jax.org/searches/Phat.cgi?id=MP:0001614) | MP:0001614 |
|  |  | [postnatal growth retardation](http://www.informatics.jax.org/searches/Phat.cgi?id=MP:0001732) | MP:0001732 |
|  |  | [postnatal lethality](http://www.informatics.jax.org/searches/Phat.cgi?id=MP:0002082) | MP:0002082 |
|  |  | [abnormal spleen periarteriolar lymphoid sheath morphology](http://www.informatics.jax.org/searches/Phat.cgi?id=MP:0002358) | MP:0002358 |
|  |  | [abnormal spleen marginal sinus morphology](http://www.informatics.jax.org/searches/Phat.cgi?id=MP:0002363) | MP:0002363 |
|  |  | [abnormal Peyer's patch follicle morphology](http://www.informatics.jax.org/searches/Phat.cgi?id=MP:0002389) | MP:0002389 |
|  |  | [abnormal ileum morphology](http://www.informatics.jax.org/searches/Phat.cgi?id=MP:0002581) | MP:0002581 |
|  |  | [abnormal leukocyte migration](http://www.informatics.jax.org/searches/Phat.cgi?id=MP:0003156) | MP:0003156 |
|  |  | [abnormal jejunum morphology](http://www.informatics.jax.org/searches/Phat.cgi?id=MP:0004002) | MP:0004002 |
|  |  | [decreased spleen weight](http://www.informatics.jax.org/searches/Phat.cgi?id=MP:0004953) | MP:0004953 |
|  |  | [decreased lymphocyte cell number](http://www.informatics.jax.org/searches/Phat.cgi?id=MP:0005016) | MP:0005016 |
|  |  | [decreased B cell number](http://www.informatics.jax.org/searches/Phat.cgi?id=MP:0005017) | MP:0005017 |
|  |  | [decreased T cell number](http://www.informatics.jax.org/searches/Phat.cgi?id=MP:0005018) | MP:0005018 |
|  |  | [cachexia](http://www.informatics.jax.org/searches/Phat.cgi?id=MP:0005150) | MP:0005150 |
|  |  | [abnormal mesenteric lymph node morphology](http://www.informatics.jax.org/searches/Phat.cgi?id=MP:0005232) | MP:0005232 |
|  |  | [decreased Peyer's patch number](http://www.informatics.jax.org/searches/Phat.cgi?id=MP:0008133) | MP:0008133 |
|  |  | [small Peyer's patches](http://www.informatics.jax.org/searches/Phat.cgi?id=MP:0008135) | MP:0008135 |
|  |  | [abnormal marginal zone B cell morphology](http://www.informatics.jax.org/searches/Phat.cgi?id=MP:0008180) | MP:0008180 |
|  |  | [absent marginal zone B cells](http://www.informatics.jax.org/searches/Phat.cgi?id=MP:0008183) | MP:0008183 |
|  |  | [absent spleen marginal zone](http://www.informatics.jax.org/searches/Phat.cgi?id=MP:0008234) | MP:0008234 |
|  |  | [abnormal spleen marginal zone macrophage morphology](http://www.informatics.jax.org/searches/Phat.cgi?id=MP:0008240) | MP:0008240 |
|  |  | [abnormal metallophillic macrophage morphology](http://www.informatics.jax.org/searches/Phat.cgi?id=MP:0008241) | MP:0008241 |
|  |  | [abnormal spleen primary B follicle morphology](http://www.informatics.jax.org/searches/Phat.cgi?id=MP:0008471) | MP:0008471 |
|  |  | [intermingled spleen red and white pulp](http://www.informatics.jax.org/searches/Phat.cgi?id=MP:0008475) | MP:0008475 |
|  |  | [decreased spleen white pulp amount](http://www.informatics.jax.org/searches/Phat.cgi?id=MP:0008479) | MP:0008479 |
|  |  | [abnormal splenic cell ratio](http://www.informatics.jax.org/searches/Phat.cgi?id=MP:0008826) | MP:0008826 |
| [Prkcq](http://www.informatics.jax.org/javawi2/servlet/WIFetch?page=markerDetail&id=MGI:97601) | MGI:2664379 | [abnormal T cell activation](http://www.informatics.jax.org/searches/Phat.cgi?id=MP:0001828) | MP:0001828 |
|  | MGI:2664377 | [abnormal T cell physiology](http://www.informatics.jax.org/searches/Phat.cgi?id=MP:0002444) | MP:0002444 |
|  |  | [decreased T cell proliferation](http://www.informatics.jax.org/searches/Phat.cgi?id=MP:0005095) | MP:0005095 |
| [Pln](http://www.informatics.jax.org/javawi2/servlet/WIFetch?page=markerDetail&id=MGI:97622) | MGI:2158357 | [abnormal blood pressure](http://www.informatics.jax.org/searches/Phat.cgi?id=MP:0000230) | MP:0000230 |
|  |  | [abnormal heart shape](http://www.informatics.jax.org/searches/Phat.cgi?id=MP:0000277) | MP:0000277 |
|  |  | [abnormal cardiac stroke volume](http://www.informatics.jax.org/searches/Phat.cgi?id=MP:0000304) | MP:0000304 |
|  |  | [cardiac hypertrophy](http://www.informatics.jax.org/searches/Phat.cgi?id=MP:0001625) | MP:0001625 |
|  |  | [abnormal cardiac output](http://www.informatics.jax.org/searches/Phat.cgi?id=MP:0001627) | MP:0001627 |
|  |  | [premature death](http://www.informatics.jax.org/searches/Phat.cgi?id=MP:0002083) | MP:0002083 |
|  |  | [increased heart weight](http://www.informatics.jax.org/searches/Phat.cgi?id=MP:0002833) | MP:0002833 |
|  |  | [increased blood pressure](http://www.informatics.jax.org/searches/Phat.cgi?id=MP:0002842) | MP:0002842 |
|  |  | [decreased blood pressure](http://www.informatics.jax.org/searches/Phat.cgi?id=MP:0002843) | MP:0002843 |
|  |  | [thick ventricular wall](http://www.informatics.jax.org/searches/Phat.cgi?id=MP:0002953) | MP:0002953 |
|  |  | [abnormal locomotor activation](http://www.informatics.jax.org/searches/Phat.cgi?id=MP:0003313) | MP:0003313 |
|  |  | [abnormal cardiac muscle relaxation](http://www.informatics.jax.org/searches/Phat.cgi?id=MP:0004084) | MP:0004084 |
|  |  | [abnormal myocardial fiber physiology](http://www.informatics.jax.org/searches/Phat.cgi?id=MP:0004215) | MP:0004215 |
|  |  | [abnormal calcium ion homeostasis](http://www.informatics.jax.org/searches/Phat.cgi?id=MP:0004231) | MP:0004231 |
|  |  | [enlarged myocardial fiber](http://www.informatics.jax.org/searches/Phat.cgi?id=MP:0004564) | MP:0004564 |
|  |  | [decreased heart rate](http://www.informatics.jax.org/searches/Phat.cgi?id=MP:0005333) | MP:0005333 |
|  |  | [increased cardiac muscle contractility](http://www.informatics.jax.org/searches/Phat.cgi?id=MP:0005599) | MP:0005599 |
|  |  | [congestive heart failure](http://www.informatics.jax.org/searches/Phat.cgi?id=MP:0006138) | MP:0006138 |
| [Prnp](http://www.informatics.jax.org/javawi2/servlet/WIFetch?page=markerDetail&id=MGI:97769) | MGI:1888773 | [kyphosis](http://www.informatics.jax.org/searches/Phat.cgi?id=MP:0000160) | MP:0000160 |
|  |  | [tremors](http://www.informatics.jax.org/searches/Phat.cgi?id=MP:0000745) | MP:0000745 |
|  |  | [hindlimb paralysis](http://www.informatics.jax.org/searches/Phat.cgi?id=MP:0000755) | MP:0000755 |
|  |  | [abnormal cerebellum morphology](http://www.informatics.jax.org/searches/Phat.cgi?id=MP:0000849) | MP:0000849 |
|  |  | [Purkinje cell degeneration](http://www.informatics.jax.org/searches/Phat.cgi?id=MP:0000876) | MP:0000876 |
|  |  | [abnormal Purkinje cell morphology](http://www.informatics.jax.org/searches/Phat.cgi?id=MP:0000877) | MP:0000877 |
|  |  | [decreased Purkinje cell number](http://www.informatics.jax.org/searches/Phat.cgi?id=MP:0000880) | MP:0000880 |
|  |  | [thin cerebellar molecular layer](http://www.informatics.jax.org/searches/Phat.cgi?id=MP:0000890) | MP:0000890 |
|  |  | [decreased body weight](http://www.informatics.jax.org/searches/Phat.cgi?id=MP:0001262) | MP:0001262 |
|  |  | [decreased anxiety-related response](http://www.informatics.jax.org/searches/Phat.cgi?id=MP:0001364) | MP:0001364 |
|  |  | [ataxia](http://www.informatics.jax.org/searches/Phat.cgi?id=MP:0001393) | MP:0001393 |
|  |  | [hyperactivity](http://www.informatics.jax.org/searches/Phat.cgi?id=MP:0001399) | MP:0001399 |
|  |  | [hypoactivity](http://www.informatics.jax.org/searches/Phat.cgi?id=MP:0001402) | MP:0001402 |
|  |  | [impaired coordination](http://www.informatics.jax.org/searches/Phat.cgi?id=MP:0001405) | MP:0001405 |
|  |  | [abnormal gait](http://www.informatics.jax.org/searches/Phat.cgi?id=MP:0001406) | MP:0001406 |
|  |  | [decreased grooming behavior](http://www.informatics.jax.org/searches/Phat.cgi?id=MP:0001442) | MP:0001442 |
|  |  | [abnormal object recognition memory](http://www.informatics.jax.org/searches/Phat.cgi?id=MP:0001458) | MP:0001458 |
|  |  | [reduced long term potentiation](http://www.informatics.jax.org/searches/Phat.cgi?id=MP:0001473) | MP:0001473 |
|  |  | [limb grasping](http://www.informatics.jax.org/searches/Phat.cgi?id=MP:0001513) | MP:0001513 |
|  |  | [impaired righting response](http://www.informatics.jax.org/searches/Phat.cgi?id=MP:0001523) | MP:0001523 |
|  |  | [impaired balance](http://www.informatics.jax.org/searches/Phat.cgi?id=MP:0001525) | MP:0001525 |
|  |  | [abnormal motor capabilities/coordination/movement](http://www.informatics.jax.org/searches/Phat.cgi?id=MP:0002066) | MP:0002066 |
|  |  | [premature death](http://www.informatics.jax.org/searches/Phat.cgi?id=MP:0002083) | MP:0002083 |
|  |  | [abnormal CNS synaptic transmission](http://www.informatics.jax.org/searches/Phat.cgi?id=MP:0002206) | MP:0002206 |
|  |  | [decreased susceptibility to viral infection](http://www.informatics.jax.org/searches/Phat.cgi?id=MP:0002410) | MP:0002410 |
|  |  | [increased susceptibility to viral infection](http://www.informatics.jax.org/searches/Phat.cgi?id=MP:0002418) | MP:0002418 |
|  |  | [spongiform encephalopathy](http://www.informatics.jax.org/searches/Phat.cgi?id=MP:0002654) | MP:0002654 |
|  |  | [abnormal motor learning](http://www.informatics.jax.org/searches/Phat.cgi?id=MP:0002804) | MP:0002804 |
|  |  | [abnormal inhibitory postsynaptic potential](http://www.informatics.jax.org/searches/Phat.cgi?id=MP:0002911) | MP:0002911 |
|  |  | [abnormal excitatory postsynaptic potential](http://www.informatics.jax.org/searches/Phat.cgi?id=MP:0002912) | MP:0002912 |
|  |  | [abnormal inhibitory postsynaptic currents](http://www.informatics.jax.org/searches/Phat.cgi?id=MP:0002945) | MP:0002945 |
|  |  | [amyloid beta deposits](http://www.informatics.jax.org/searches/Phat.cgi?id=MP:0003329) | MP:0003329 |
|  |  | [abnormal afterhyperpolarization](http://www.informatics.jax.org/searches/Phat.cgi?id=MP:0003412) | MP:0003412 |
|  |  | [abnormal response to novel object](http://www.informatics.jax.org/searches/Phat.cgi?id=MP:0003461) | MP:0003461 |
|  |  | [neuronal intranuclear inclusions](http://www.informatics.jax.org/searches/Phat.cgi?id=MP:0004191) | MP:0004191 |
|  |  | [abnormal behavior](http://www.informatics.jax.org/searches/Phat.cgi?id=MP:0004924) | MP:0004924 |
|  |  | [increased susceptibility to prion infection](http://www.informatics.jax.org/searches/Phat.cgi?id=MP:0005364) | MP:0005364 |
|  |  | [brain vacuoles](http://www.informatics.jax.org/searches/Phat.cgi?id=MP:0008025) | MP:0008025 |
|  |  | [increased sensitivity to induced morbidity/mortality](http://www.informatics.jax.org/searches/Phat.cgi?id=MP:0009763) | MP:0009763 |
|  | MGI:1934272 | [Purkinje cell degeneration](http://www.informatics.jax.org/searches/Phat.cgi?id=MP:0000876) | MP:0000876 |
|  |  | [ataxia](http://www.informatics.jax.org/searches/Phat.cgi?id=MP:0001393) | MP:0001393 |
|  |  | [impaired coordination](http://www.informatics.jax.org/searches/Phat.cgi?id=MP:0001405) | MP:0001405 |
|  |  | [decreased susceptibility to prion infection](http://www.informatics.jax.org/searches/Phat.cgi?id=MP:0005363) | MP:0005363 |
|  | MGI:2387688 | [decreased susceptibility to prion infection](http://www.informatics.jax.org/searches/Phat.cgi?id=MP:0005363) | MP:0005363 |
|  | MGI:2682351 | [ataxia](http://www.informatics.jax.org/searches/Phat.cgi?id=MP:0001393) | MP:0001393 |
|  | MGI:2682338 | [tremors](http://www.informatics.jax.org/searches/Phat.cgi?id=MP:0000745) | MP:0000745 |
|  |  | [abnormal cerebellum morphology](http://www.informatics.jax.org/searches/Phat.cgi?id=MP:0000849) | MP:0000849 |
|  |  | [decreased Purkinje cell number](http://www.informatics.jax.org/searches/Phat.cgi?id=MP:0000880) | MP:0000880 |
|  |  | [thin cerebellar molecular layer](http://www.informatics.jax.org/searches/Phat.cgi?id=MP:0000890) | MP:0000890 |
|  |  | [ataxia](http://www.informatics.jax.org/searches/Phat.cgi?id=MP:0001393) | MP:0001393 |
|  |  | [abnormal muscle physiology](http://www.informatics.jax.org/searches/Phat.cgi?id=MP:0002106) | MP:0002106 |
|  |  | [gliosis](http://www.informatics.jax.org/searches/Phat.cgi?id=MP:0002183) | MP:0002183 |
|  | MGI:3047062 | [impaired fertilization](http://www.informatics.jax.org/searches/Phat.cgi?id=MP:0000242) | MP:0000242 |
|  |  | [male infertility](http://www.informatics.jax.org/searches/Phat.cgi?id=MP:0001925) | MP:0001925 |
|  |  | [impaired acrosome reaction](http://www.informatics.jax.org/searches/Phat.cgi?id=MP:0004542) | MP:0004542 |
|  | MGI:3052798 | [decreased susceptibility to prion infection](http://www.informatics.jax.org/searches/Phat.cgi?id=MP:0005363) | MP:0005363 |
|  | MGI:3052799 | [decreased susceptibility to prion infection](http://www.informatics.jax.org/searches/Phat.cgi?id=MP:0005363) | MP:0005363 |
|  | MGI:3047635 | [male infertility](http://www.informatics.jax.org/searches/Phat.cgi?id=MP:0001925) | MP:0001925 |
|  |  | [teratozoospermia](http://www.informatics.jax.org/searches/Phat.cgi?id=MP:0005578) | MP:0005578 |
|  | MGI:3054245 | [tremors](http://www.informatics.jax.org/searches/Phat.cgi?id=MP:0000745) | MP:0000745 |
|  |  | [ataxia](http://www.informatics.jax.org/searches/Phat.cgi?id=MP:0001393) | MP:0001393 |
| [Ptprd](http://www.informatics.jax.org/javawi2/servlet/WIFetch?page=markerDetail&id=MGI:97812) | MGI:2158795 | [decreased body weight](http://www.informatics.jax.org/searches/Phat.cgi?id=MP:0001262) | MP:0001262 |
|  |  | [abnormal eating behavior](http://www.informatics.jax.org/searches/Phat.cgi?id=MP:0001431) | MP:0001431 |
|  |  | [abnormal spatial learning](http://www.informatics.jax.org/searches/Phat.cgi?id=MP:0001463) | MP:0001463 |
|  |  | [impaired swimming](http://www.informatics.jax.org/searches/Phat.cgi?id=MP:0001522) | MP:0001522 |
|  |  | [premature death](http://www.informatics.jax.org/searches/Phat.cgi?id=MP:0002083) | MP:0002083 |
|  |  | [enhanced paired-pulse facilitation](http://www.informatics.jax.org/searches/Phat.cgi?id=MP:0002919) | MP:0002919 |
|  |  | [enhanced long term potentiation](http://www.informatics.jax.org/searches/Phat.cgi?id=MP:0003008) | MP:0003008 |
| [Il1rl1](http://www.informatics.jax.org/javawi2/servlet/WIFetch?page=markerDetail&id=MGI:98427) | MGI:3043989 | [increased eosinophil cell number](http://www.informatics.jax.org/searches/Phat.cgi?id=MP:0005011) | MP:0005011 |
|  |  | [abnormal response to infection](http://www.informatics.jax.org/searches/Phat.cgi?id=MP:0005025) | MP:0005025 |
|  |  | [decreased interleukin-5 secretion](http://www.informatics.jax.org/searches/Phat.cgi?id=MP:0008703) | MP:0008703 |
|  | MGI:3623124 | [no abnormal phenotype detected](http://www.informatics.jax.org/searches/Phat.cgi?id=MP:0002169) | MP:0002169 |
|  | MGI:2386675 | [abnormal eosinophil physiology](http://www.informatics.jax.org/searches/Phat.cgi?id=MP:0002465) | MP:0002465 |
|  |  | [granulomatous inflammation](http://www.informatics.jax.org/searches/Phat.cgi?id=MP:0002500) | MP:0002500 |
|  |  | [abnormal T-helper 2 physiology](http://www.informatics.jax.org/searches/Phat.cgi?id=MP:0005466) | MP:0005466 |
|  |  | [abnormal interleukin secretion](http://www.informatics.jax.org/searches/Phat.cgi?id=MP:0008568) | MP:0008568 |
|  |  | [decreased interleukin-4 secretion](http://www.informatics.jax.org/searches/Phat.cgi?id=MP:0008700) | MP:0008700 |
|  |  | [decreased interleukin-5 secretion](http://www.informatics.jax.org/searches/Phat.cgi?id=MP:0008703) | MP:0008703 |
| [Mc4r](http://www.informatics.jax.org/javawi2/servlet/WIFetch?page=markerDetail&id=MGI:99457) | MGI:3512050 | [increased body length](http://www.informatics.jax.org/searches/Phat.cgi?id=MP:0001257) | MP:0001257 |
|  |  | [increased body weight](http://www.informatics.jax.org/searches/Phat.cgi?id=MP:0001260) | MP:0001260 |
|  |  | [obese](http://www.informatics.jax.org/searches/Phat.cgi?id=MP:0001261) | MP:0001261 |
|  |  | [polyphagia](http://www.informatics.jax.org/searches/Phat.cgi?id=MP:0001433) | MP:0001433 |
|  |  | [increased circulating insulin level](http://www.informatics.jax.org/searches/Phat.cgi?id=MP:0002079) | MP:0002079 |
|  |  | [no abnormal phenotype detected](http://www.informatics.jax.org/searches/Phat.cgi?id=MP:0002169) | MP:0002169 |
|  |  | [increased lean body mass](http://www.informatics.jax.org/searches/Phat.cgi?id=MP:0003960) | MP:0003960 |
|  |  | [abnormal oxygen consumption](http://www.informatics.jax.org/searches/Phat.cgi?id=MP:0005288) | MP:0005288 |
|  |  | [decreased oxygen consumption](http://www.informatics.jax.org/searches/Phat.cgi?id=MP:0005290) | MP:0005290 |
|  |  | [abnormal food intake](http://www.informatics.jax.org/searches/Phat.cgi?id=MP:0005449) | MP:0005449 |
|  |  | [decreased body mass index](http://www.informatics.jax.org/searches/Phat.cgi?id=MP:0006086) | MP:0006086 |
|  | MGI:2183586 | [increased body weight](http://www.informatics.jax.org/searches/Phat.cgi?id=MP:0001260) | MP:0001260 |
|  |  | [increased body size](http://www.informatics.jax.org/searches/Phat.cgi?id=MP:0001264) | MP:0001264 |
|  |  | [polyphagia](http://www.informatics.jax.org/searches/Phat.cgi?id=MP:0001433) | MP:0001433 |
|  |  | [hyperglycemia](http://www.informatics.jax.org/searches/Phat.cgi?id=MP:0001559) | MP:0001559 |
|  |  | [reduced fertility](http://www.informatics.jax.org/searches/Phat.cgi?id=MP:0001921) | MP:0001921 |
|  |  | [abnormal glucose homeostasis](http://www.informatics.jax.org/searches/Phat.cgi?id=MP:0002078) | MP:0002078 |
|  |  | [increased circulating insulin level](http://www.informatics.jax.org/searches/Phat.cgi?id=MP:0002079) | MP:0002079 |
|  |  | [abnormal circadian phase](http://www.informatics.jax.org/searches/Phat.cgi?id=MP:0002561) | MP:0002561 |
|  |  | [abnormal locomotor activation](http://www.informatics.jax.org/searches/Phat.cgi?id=MP:0003313) | MP:0003313 |
|  |  | [decreased osteoclast cell number](http://www.informatics.jax.org/searches/Phat.cgi?id=MP:0004985) | MP:0004985 |
|  |  | [abnormal food intake](http://www.informatics.jax.org/searches/Phat.cgi?id=MP:0005449) | MP:0005449 |
|  |  | [weight gain](http://www.informatics.jax.org/searches/Phat.cgi?id=MP:0005456) | MP:0005456 |
|  |  | [increased bone mass](http://www.informatics.jax.org/searches/Phat.cgi?id=MP:0005605) | MP:0005605 |
|  |  | [increased susceptibility to diet-induced obesity](http://www.informatics.jax.org/searches/Phat.cgi?id=MP:0005658) | MP:0005658 |
|  |  | [increased circulating leptin level](http://www.informatics.jax.org/searches/Phat.cgi?id=MP:0005669) | MP:0005669 |
| [Cdh13](http://www.informatics.jax.org/javawi2/servlet/WIFetch?page=markerDetail&id=MGI:99551) | MGI:3778287 | [increased metastatic potential](http://www.informatics.jax.org/searches/Phat.cgi?id=MP:0001272) | MP:0001272 |
|  |  | [decreased incidence of induced tumors](http://www.informatics.jax.org/searches/Phat.cgi?id=MP:0002053) | MP:0002053 |
|  |  | [decreased tumor growth/size](http://www.informatics.jax.org/searches/Phat.cgi?id=MP:0003447) | MP:0003447 |
|  |  | [altered tumor morphology](http://www.informatics.jax.org/searches/Phat.cgi?id=MP:0003448) | MP:0003448 |
|  |  | [pathological neovascularization](http://www.informatics.jax.org/searches/Phat.cgi?id=MP:0003711) | MP:0003711 |
|  |  | [increased adiponectin level](http://www.informatics.jax.org/searches/Phat.cgi?id=MP:0004892) | MP:0004892 |
|  |  | [mammary gland tumor](http://www.informatics.jax.org/searches/Phat.cgi?id=MP:0006318) | MP:0006318 |
|  |  | [retinal neovascularization](http://www.informatics.jax.org/searches/Phat.cgi?id=MP:0008852) | MP:0008852 |
| [Abca1](http://www.informatics.jax.org/javawi2/servlet/WIFetch?page=markerDetail&id=MGI:99607) | MGI:1935192 | [decreased circulating LDL cholesterol level](http://www.informatics.jax.org/searches/Phat.cgi?id=MP:0000183) | MP:0000183 |
|  |  | [decreased circulating HDL cholesterol level](http://www.informatics.jax.org/searches/Phat.cgi?id=MP:0000186) | MP:0000186 |
|  |  | [enlarged adrenal glands](http://www.informatics.jax.org/searches/Phat.cgi?id=MP:0000642) | MP:0000642 |
|  |  | [enlarged spleen](http://www.informatics.jax.org/searches/Phat.cgi?id=MP:0000691) | MP:0000691 |
|  |  | [abnormal thymus morphology](http://www.informatics.jax.org/searches/Phat.cgi?id=MP:0000703) | MP:0000703 |
|  |  | [abnormal lung morphology](http://www.informatics.jax.org/searches/Phat.cgi?id=MP:0001175) | MP:0001175 |
|  |  | [decreased body weight](http://www.informatics.jax.org/searches/Phat.cgi?id=MP:0001262) | MP:0001262 |
|  |  | [abnormal placenta development](http://www.informatics.jax.org/searches/Phat.cgi?id=MP:0001712) | MP:0001712 |
|  |  | [impaired macrophage phagocytosis](http://www.informatics.jax.org/searches/Phat.cgi?id=MP:0001798) | MP:0001798 |
|  |  | [hemorrhage](http://www.informatics.jax.org/searches/Phat.cgi?id=MP:0001914) | MP:0001914 |
|  |  | [female infertility](http://www.informatics.jax.org/searches/Phat.cgi?id=MP:0001926) | MP:0001926 |
|  |  | [perinatal lethality](http://www.informatics.jax.org/searches/Phat.cgi?id=MP:0002081) | MP:0002081 |
|  |  | [postnatal lethality](http://www.informatics.jax.org/searches/Phat.cgi?id=MP:0002082) | MP:0002082 |
|  |  | [abnormal lipid homeostasis](http://www.informatics.jax.org/searches/Phat.cgi?id=MP:0002118) | MP:0002118 |
|  |  | [abnormal respiratory alveoli morphology](http://www.informatics.jax.org/searches/Phat.cgi?id=MP:0002270) | MP:0002270 |
|  |  | [abnormal macrophage physiology](http://www.informatics.jax.org/searches/Phat.cgi?id=MP:0002451) | MP:0002451 |
|  |  | [increased cholesterol absorption](http://www.informatics.jax.org/searches/Phat.cgi?id=MP:0002646) | MP:0002646 |
|  |  | [abnormal cytokine secretion](http://www.informatics.jax.org/searches/Phat.cgi?id=MP:0003009) | MP:0003009 |
|  |  | [decreased platelet cell number](http://www.informatics.jax.org/searches/Phat.cgi?id=MP:0003179) | MP:0003179 |
|  |  | [increased cholesterol efflux](http://www.informatics.jax.org/searches/Phat.cgi?id=MP:0003192) | MP:0003192 |
|  |  | [decreased cholesterol efflux](http://www.informatics.jax.org/searches/Phat.cgi?id=MP:0003193) | MP:0003193 |
|  |  | [decreased circulating cholesterol level](http://www.informatics.jax.org/searches/Phat.cgi?id=MP:0005179) | MP:0005179 |
|  |  | [abnormal fat-soluble vitamin level](http://www.informatics.jax.org/searches/Phat.cgi?id=MP:0005401) | MP:0005401 |
|  |  | [abnormal circulating phospholipid level](http://www.informatics.jax.org/searches/Phat.cgi?id=MP:0006084) | MP:0006084 |
|  |  | [abnormal chemokine secretion](http://www.informatics.jax.org/searches/Phat.cgi?id=MP:0008722) | MP:0008722 |
|  |  | [abnormal circulating plant sterol concentration](http://www.informatics.jax.org/searches/Phat.cgi?id=MP:0010075) | MP:0010075 |
|  | MGI:1935193 | [decreased circulating HDL cholesterol level](http://www.informatics.jax.org/searches/Phat.cgi?id=MP:0000186) | MP:0000186 |
|  |  | [heart right ventricle hypertrophy](http://www.informatics.jax.org/searches/Phat.cgi?id=MP:0000276) | MP:0000276 |
|  |  | [abnormal kidney cortex](http://www.informatics.jax.org/searches/Phat.cgi?id=MP:0000521) | MP:0000521 |
|  |  | [abnormal adrenal gland morphology](http://www.informatics.jax.org/searches/Phat.cgi?id=MP:0000639) | MP:0000639 |
|  |  | [abnormal thymus morphology](http://www.informatics.jax.org/searches/Phat.cgi?id=MP:0000703) | MP:0000703 |
|  |  | [abnormal uterus morphology](http://www.informatics.jax.org/searches/Phat.cgi?id=MP:0001120) | MP:0001120 |
|  |  | [abnormal ovary morphology](http://www.informatics.jax.org/searches/Phat.cgi?id=MP:0001126) | MP:0001126 |
|  |  | [lung hemorrhage](http://www.informatics.jax.org/searches/Phat.cgi?id=MP:0001182) | MP:0001182 |
|  |  | [decreased body size](http://www.informatics.jax.org/searches/Phat.cgi?id=MP:0001265) | MP:0001265 |
|  |  | [abnormal placenta labyrinth morphology](http://www.informatics.jax.org/searches/Phat.cgi?id=MP:0001716) | MP:0001716 |
|  |  | [embryonic growth arrest](http://www.informatics.jax.org/searches/Phat.cgi?id=MP:0001730) | MP:0001730 |
|  |  | [reduced female fertility](http://www.informatics.jax.org/searches/Phat.cgi?id=MP:0001923) | MP:0001923 |
|  |  | [respiratory distress](http://www.informatics.jax.org/searches/Phat.cgi?id=MP:0001954) | MP:0001954 |
|  |  | [postnatal lethality](http://www.informatics.jax.org/searches/Phat.cgi?id=MP:0002082) | MP:0002082 |
|  |  | [premature death](http://www.informatics.jax.org/searches/Phat.cgi?id=MP:0002083) | MP:0002083 |
|  |  | [abnormal brain morphology](http://www.informatics.jax.org/searches/Phat.cgi?id=MP:0002152) | MP:0002152 |
|  |  | [heart left ventricle hypertrophy](http://www.informatics.jax.org/searches/Phat.cgi?id=MP:0002625) | MP:0002625 |
|  |  | [hepatic steatosis](http://www.informatics.jax.org/searches/Phat.cgi?id=MP:0002628) | MP:0002628 |
|  |  | [decreased cholesterol absorption](http://www.informatics.jax.org/searches/Phat.cgi?id=MP:0002647) | MP:0002647 |
|  |  | [glomerulonephritis](http://www.informatics.jax.org/searches/Phat.cgi?id=MP:0002743) | MP:0002743 |
|  |  | [abnormal Sertoli cell morphology](http://www.informatics.jax.org/searches/Phat.cgi?id=MP:0002784) | MP:0002784 |
|  |  | [increased cholesterol level](http://www.informatics.jax.org/searches/Phat.cgi?id=MP:0003982) | MP:0003982 |
|  |  | [embryonic growth retardation](http://www.informatics.jax.org/searches/Phat.cgi?id=MP:0003984) | MP:0003984 |
|  |  | [decreased circulating cholesterol level](http://www.informatics.jax.org/searches/Phat.cgi?id=MP:0005179) | MP:0005179 |
|  |  | [decreased circulating estradiol level](http://www.informatics.jax.org/searches/Phat.cgi?id=MP:0005181) | MP:0005181 |
|  |  | [decreased circulating progesterone level](http://www.informatics.jax.org/searches/Phat.cgi?id=MP:0005185) | MP:0005185 |
|  |  | [glomerulosclerosis](http://www.informatics.jax.org/searches/Phat.cgi?id=MP:0005264) | MP:0005264 |
|  |  | [cardiomyopathy](http://www.informatics.jax.org/searches/Phat.cgi?id=MP:0005330) | MP:0005330 |
|  |  | [lethality throughout fetal growth and development](http://www.informatics.jax.org/searches/Phat.cgi?id=MP:0006208) | MP:0006208 |
|  |  | [uterus inflammation](http://www.informatics.jax.org/searches/Phat.cgi?id=MP:0009080) | MP:0009080 |
|  | MGI:3525100 | [abnormal platelet physiology](http://www.informatics.jax.org/searches/Phat.cgi?id=MP:0005464) | MP:0005464 |
|  | MGI:3577725 | [decreased circulating HDL cholesterol level](http://www.informatics.jax.org/searches/Phat.cgi?id=MP:0000186) | MP:0000186 |
|  |  | [decreased circulating cholesterol level](http://www.informatics.jax.org/searches/Phat.cgi?id=MP:0005179) | MP:0005179 |
|  |  | [abnormal circulating phospholipid level](http://www.informatics.jax.org/searches/Phat.cgi?id=MP:0006084) | MP:0006084 |
|  | MGI:3613250 | [abnormal circulating cholesterol level](http://www.informatics.jax.org/searches/Phat.cgi?id=MP:0000180) | MP:0000180 |
|  |  | [decreased circulating HDL cholesterol level](http://www.informatics.jax.org/searches/Phat.cgi?id=MP:0000186) | MP:0000186 |
|  |  | [abnormal kidney physiology](http://www.informatics.jax.org/searches/Phat.cgi?id=MP:0002136) | MP:0002136 |
|  |  | [decreased circulating phospholipid level](http://www.informatics.jax.org/searches/Phat.cgi?id=MP:0003981) | MP:0003981 |
|  |  | [abnormal circulating protein level](http://www.informatics.jax.org/searches/Phat.cgi?id=MP:0005416) | MP:0005416 |
