# Supplementary material for: Laboratory Mouse Models for the Human Genome-Wide Associations
Source: PLoS One. 2010 Nov 1;5(11):e13782. doi: 10.1371/journal.pone.0013782 (PMC2967475; doi:10.1371/journal.pone.0013782)
Supplement: Table S9 — Orthologs that displayed lethality in knocked out models. (0.13 MB DOC) [file pone.0013782.s009.doc]

| Reported Gene(s) | Homolog gene Symbol | MGI ID | Human Disease/Trait | MP term | MP id | Anatomical system | Number of available KO models | N of systems | Type of lethality |
| --- | --- | --- | --- | --- | --- | --- | --- | --- | --- |
| FGFR2 | Fgfr2 | MGI:95523 | Breast cancer | mammary gland tumor | MP:0006318 | tumorigenesis | 11 | 23 | Pre-, perinatal, postnatal |
| ABCA1 | Abca1 | MGI:99607 | Lipid phenotypes | abnormal lipid level | MP:0001547 | homeostasis/metabolism | 5 | 17 | Pre-, perinatal, postnatal |
| BDNF | Bdnf | MGI:88145 | Obesity related phenotypes | increased body weight | MP:0001260 | growth size | 5 | 15 | Pre-, perinatal, postnatal |
| ERBB3 | Erbb3 | MGI:95411 | Type 1 diabetes | Increased susceptibility to autoimmune diabetes | MP:0004803 | immune system | 4 | 16 | Pre-, perinatal, postnatal |
| GCK | Gck | MGI:1270854 | Type 2 diabetes | insulin resistance | MP:0005331 | homeostasis/metabolism | 4 | 8 | Pre-, perinatal, postnatal |
| PTGER4 | Ptger4 | MGI:104311 | Inflammatory bowel disease | intestinal inflammation | MP:0001858 | immune system digestive/alimentary | 3 | 12 | Pre-, perinatal, postnatal |
| SMAD7 | Smad7 | MGI:1100518 | Colorectal cancer | large intestine adenocarcinoma | MP:0009310 | tumorigenesis | 3 | 10 | Pre-, perinatal, postnatal |
| MAF | Maf | MGI:96909 | Obesity related phenotypes | increased body weight | MP:0001260 | growth size | 1 | 5 | Pre-, perinatal, postnatal |
| PTCH1 | Ptch1 | MGI:105373 | Height | abnormal body height | MP:0001253 | growth size | 5 | 10 | Pre-, perinatal |
| STAT3 | Stat3 | MGI:103038 | Inflammatory bowel disease | intestinal inflammation | MP:0001858 | immune system digestive/alimentary | 5 | 9 | Pre-, perinatal |
| APOB | Apob | MGI:88052 | Lipid phenotypes | abnormal lipid level | MP:0001547 | homeostasis/metabolism | 4 | 8 | Pre-, perinatal |
| HFE | Hfe | MGI:109191 | Serum markers of iron status | hypoferremia | MP:0004151 | homeostasis/metabolism | 4 | 10 | Postnatal |
| ANGPTL3 | Angptl3 | MGI:1353627 | Lipid phenotypes | abnormal lipid level | MP:0001547 | homeostasis/metabolism | 3 | 6 | Pre-, perinatal |
| HIST1H1D | Hist1h1d | MGI:107502 | Height | abnormal body height | MP:0001253 | growth size | 3 | 9 | Pre-, perinatal |
| TNFRSF11B | Tnfrsf11b | MGI:109587 | Bone mineral density | osteoporosis | MP:0000066 | skeleton homeostasis/metabolism | 3 | 9 | Postnatal |
| BCL11A | Bcl11a | MGI:106190 | F-cell distribution; Fetal hemoglobin levels | 0 |  | homeostasis/metabolism hematopoietic | 3 | 3 | Pre-, perinatal |
| BMP4 | Bmp4 | MGI:88180 | Colorectal cancer | large intestine adenocarcinoma | MP:0009310 | tumorigenesis | 3 | 19 | Pre-, perinatal |
| JAK2 | Jak2 | MGI:96629 | Inflammatory bowel disease; Myeloproliferative neoplasms | intestinal inflammation; 0 | MP:0001858; 0 | immune system digestive/alimentary tumorigenesis | 3 | 2 | Pre-, perinatal |
| LDLR | Ldlr | MGI:96765 | Coronary disease; Lipid phenotypes | coronary artery stenosis; abnormal lipid level | MP:0003024; MP:0001547 | cardiovascular homeostasis/metabolism | 2 | 14 | Postnatal |
| ALPL | Alpl | MGI:87983 | Folate pathway vitamins and vitamin B12 levels | 0 |  | homeostasis/metabolism | 2 | 12 | Pre-, perinatal |
| GATA2 | Gata2 | MGI:95662 | Plasma eosinophil count | abnormal eosinophil cell number | MP:0002602 | immune system hematopoietic | 2 | 5 | Pre-, perinatal |
| HBB | Hbb | MGI:96020 | F-cell distribution | 0 |  | homeostasis/metabolism | 2 | 8 | Pre-, perinatal |
| HHEX | Hhex | MGI:96086 | Type 2 diabetes | insulin resistance | MP:0005331 | homeostasis/metabolism | 2 | 9 | Pre-, perinatal |
| LPL | Lpl | MGI:96820 | Lipid phenotypes | abnormal lipid level | MP:0001547 | homeostasis/metabolism | 2 | 8 | Pre-, perinatal |
| SH2B1 | Sh2b1 | MGI:1201407 | Obesity related phenotypes | increased body weight | MP:0001260 | growth size | 2 | 7 | Pre-, perinatal |
| CYP17A1 | Cyp17a1 | MGI:88586 | Blood pressure related phenotypes | abnormal blood pressure | MP:0000230 | cardiovascular | 2 | 1 | Pre-, perinatal |
| GLIS3 | Glis3 | MGI:2444289 | Type 1 diabetes | Increased susceptibility to autoimmune diabetes | MP:0004803 | immune system | 2 | 6 | Postnatal |
| HNF1A | Hnf1a | MGI:98504 | CRP concentration | abnormal C-reactive protein physiology | MP:0002484 | immune system | 2 | 9 | Pre-, perinatal |
| HNF4A | Hnf4a | MGI:109128 | Lipid phenotypes | abnormal lipid level | MP:0001547 | homeostasis/metabolism | 2 | 4 | Pre-, perinatal |
| INS | Ins2 | MGI:96573 | Type 1 diabetes | Increased susceptibility to autoimmune diabetes | MP:0004803 | immune system | 2 | 2 | Postnatal |
| TNFAIP3 | Tnfaip3 | MGI:1196377 | Psoriasis; Systemic lupus erythematosus | psoriasis; increased susceptibility to systemic lupus erythematosus | MP:0001193; MP:0004801 | skin/nails immune system | 1 | 10 | Postnatal |
| FTO | Fto | MGI:1347093 | Obesity related phenotypes; Type 2 diabetes | increased body weight; insulin resistance | MP:0001260; MP:0005331 | growth size homeostasis/metabolism | 1 | 5 | Postnatal |
| ATG16L1 | Atg16l1 | MGI:1924290 | Inflammatory bowel disease | intestinal inflammation | MP:0001858 | immune system digestive/alimentary | 1 | 4 | Pre-, perinatal |
| NKX2-3 | Nkx2-3 | MGI:97348 | Inflammatory bowel disease | intestinal inflammation | MP:0001858 | immune system digestive/alimentary | 1 | 8 | Postnatal |
| ATP2B1 | Atp2b1 | MGI:104653 | Blood pressure related phenotypes | abnormal blood pressure | MP:0000230 | cardiovascular | 1 | 3 | Pre-, perinatal |
| CDK6 | Cdk6 | MGI:1277162 | Height | abnormal body height | MP:0001253 | growth size | 1 | 10 | Pre-, perinatal |
| CXCL12 | Cxcl12 | MGI:103556 | Coronary disease | coronary artery stenosis | MP:0003024 | cardiovascular | 1 | 6 | Pre-, perinatal |
| KCNJ2 | Kcnj2 | MGI:104744 | QT interval | abnormal QT interval | MP:0003899 | cardiovascular | 1 | 8 | Pre-, perinatal |
| KIF1B | Kif1b | MGI:108426 | Multiple sclerosis | demyelination | MP:0000921 | nervous | 1 | 6 | Pre-, perinatal |
| MAFB | Mafb | MGI:104555 | Lipid phenotypes | abnormal lipid level | MP:0001547 | homeostasis/metabolism | 1 | 5 | Pre-, perinatal |
| SLC2A9 | Slc2a9 | MGI:2152844 | Serum urate/uric acid | abnormal blood uric acid level | MP:0008820 | homeostasis/metabolism | 1 | 6 | Pre-, perinatal |
| TNIP1 | Tnip1 | MGI:1926194 | Psoriasis | psoriasis | MP:0001193 | skin/nails | 1 | 7 | Pre-, perinatal |
| BRSK1 | Brsk1 | MGI:2685946 | Menarche and/or menopause (age at onset) | late onset of menarche | MP:0003377 | reproductive | 1 | 4 | Pre-, perinatal |
| FOXE1 | Foxe1 | MGI:1353500 | Thyroid cancer | thyroid adenoma | MP:0003496 | tumorigenesis | 1 | 5 | Postnatal |
| GNA12 | Gna12 | MGI:95767 | Height | abnormal body height | MP:0001253 | growth size | 1 | 7 | Pre-, perinatal |
| HMGCR | Hmgcr | MGI:96159 | Lipid phenotypes | abnormal lipid level | MP:0001547 | homeostasis/metabolism | 1 | 1 | Pre-, perinatal |
| IKZF2 | Ikzf2 | MGI:1342541 | Plasma eosinophil count | abnormal eosinophil cell number | MP:0002602 | immune system hematopoietic | 1 | 3 | Postnatal |
| LEF1 | Lef1 | MGI:96770 | CRP concentration | abnormal C-reactive protein physiology | MP:0002484 | immune system | 1 | 10 | Postnatal |
| NKX2-1 | Nkx2-1 | MGI:108067 | Thyroid cancer | thyroid adenoma | MP:0003496 | tumorigenesis | 1 | 7 | Pre-, perinatal |
| SOX17 | Sox17 | MGI:107543 | Intracranial aneurysm | 0 |  | cardiovascular | 1 | 3 | Pre-, perinatal |
| TCF7L2 | Tcf7l2 | MGI:1202879 | Type 2 diabetes | insulin resistance | MP:0005331 | homeostasis/metabolism | 1 | 7 | Pre-, perinatal |
| HNF1B | Hnf1b | MGI:98505 | Prostate cancer | prostate adenocarcinoma | MP:0009220 | tumorigenesis | 1 | 3 | Pre-, perinatal |
| LMTK2 | Lmtk2 | MGI:3036247 | Prostate cancer | prostate adenocarcinoma | MP:0009220 | tumorigenesis | 1 | 3 | Pre-, perinatal |
