# Supplementary material for: Laboratory Mouse Models for the Human Genome-Wide Associations
Source: PLoS One. 2010 Nov 1;5(11):e13782. doi: 10.1371/journal.pone.0013782 (PMC2967475; doi:10.1371/journal.pone.0013782)
Supplement: Table S10 — Comparisons of phenotypic expression between human GWAS genes and ortholog knocked out genes in mice (after excluding knockout genes associated with lethality). (0.22 MB DOC) [file pone.0013782.s010.doc]

| Reported Gene(s) | Homolog gene | MGI ID | Human Disease/Trait | MP term | MP id | Anatomical system | Number of systems in humans | Number of available KO models | Number of systems | Matched anatomical systems and phenotype description | Matching system |
| --- | --- | --- | --- | --- | --- | --- | --- | --- | --- | --- | --- |
| PRNP | Prnp | MGI:97769 | Creutzfeldt-Jakob disease | spongiform encephalopathy | MP:0002654 | nervous | 1 | 10 | 7 | 1 | nervous |
| LRP5 | Lrp5 | MGI:1278315 | Bone mineral density | osteoporosis | MP:0000066 | skeleton homeostasis/metabolism | 2 | 5 | 13 | 2 | skeleton homeostasis/metabolism |
| NOD2 | Nod2 | MGI:2429397 | Inflammatory bowel disease | intestinal inflammation | MP:0001858 | immune system digestive/alimentary | 2 | 5 | 6 | 1 | immune system digestive/alimentary |
| AR | Ar | MGI:88064 | Male-pattern baldness | alopecia | MP:0000414 | skin/nails | 1 | 5 | 14 | 0 |  |
| ESR1 | Esr1 | MGI:1352467 | Bone mineral density | osteoporosis | MP:0000066 | skeleton homeostasis/metabolism | 2 | 4 | 17 | 2 | skeleton homeostasis/metabolism |
| IL5 | Il5 | MGI:96557 | Plasma eosinophil count | abnormal eosinophil cell number | MP:0002602 | immune system hematopoietic | 2 | 4 | 10 | 2 | immune system hematopoietic |
| ICAM1 | Icam1 | MGI:96392 | Soluble ICAM-1 |  |  | homeostasis/metabolism | 1 | 4 | 13 | 1 | homeostasis/metabolism |
| ICOSLG | Icosl | MGI:1354701 | Inflammatory bowel disease | intestinal inflammation | MP:0001858 | immune system digestive/alimentary | 2 | 4 | 3 | 1 | immune system |
| ADAMTS13 | Adamts13 | MGI:2685556 | Plasma levels of liver enzymes | abnormal liver physiology | MP:0000609 | liver/biliary | 1 | 4 | 5 | 0 |  |
| APOE | Apoe | MGI:88057 | Alzheimer's disease; CRP concentration | abnormal C-reactive protein physiology | MP:0002484 | nervous immune system | 1 | 3 | 21 | 2 | nervous immune system |
| IL1RL1 | Il1rl1 | MGI:98427 | Plasma eosinophil count | abnormal eosinophil cell number | MP:0002602 | immune system hematopoietic | 2 | 3 | 3 | 2 | immune system hematopoietic |
| CD40 | Cd40 | MGI:88336 | Rheumatoid arthritis | rheumatoid arthritis | MP:0003561 | immune system skeleton | 2 | 3 | 4 | 1 | immune system |
| CTLA4 | Ctla4 | MGI:88556 | Type 1 diabetes | Increased susceptibility to autoimmune diabetes | MP:0004803 | immune system | 1 | 3 | 9 | 1 | immune system |
| LCAT | Lcat | MGI:96755 | Lipid phenotypes | abnormal lipid level | MP:0001547 | homeostasis/metabolism | 1 | 3 | 4 | 1 | homeostasis/metabolism |
| LIPG | Lipg | MGI:1341803 | Lipid phenotypes | abnormal lipid level | MP:0001547 | homeostasis/metabolism | 1 | 3 | 2 | 1 | homeostasis/metabolism |
| GAB2 | Gab2 | MGI:1333854 | Alzheimer's disease | amyloid beta deposits - neurofibrillary tangles | MP:0003329 /MP:0003214 | nervous | 1 | 3 | 6 | 0 |  |
| GRM5 | Grm5 | MGI:1351342 | Skin/hair/eye color related phenotypes | abnormal coat/hair pigmentation - abnormal skin pigmentation | MP:0002075 /MP:0002095 | skin/nails | 1 | 3 | 4 | 0 |  |
| LEPR | Lepr | MGI:104993 | CRP concentration | abnormal C-reactive protein physiology | MP:0002484 | immune system | 1 | 3 | 6 | 0 |  |
| ABCG8 | Abcg8 | MGI:1914720 | Gallstones; Lipid phenotypes | gallstones; abnormal lipid level | MP:0002830; MP:0001547 | liver/biliary homeostasis/metabolism | 2 | 2 | 3 | 2 | liver/biliary homeostasis/metabolism |
| IL18 | Il18 | MGI:107936 | IL-18 concentration | abnormal circulating interleukin-18 level | MP:0008634 | immune system homeostasis/metabolism | 2 | 2 | 10 | 2 | immune system homeostasis/metabolism |
| KCNQ1 | Kcnq1 | MGI:108083 | QT interval; Type 2 diabetes | abnormal QT interval; insulin resistance | MP:0003899; MP:0005331 | cardiovascular homeostasis/metabolism | 2 | 2 | 6 | 2 | cardiovascular homeostasis/metabolism |
| SH2B3 | Sh2b3 | MGI:893598 | Plasma eosinophil count; Blood pressure related phenotypes; Type 1 diabetes | abnormal eosinophil cell number; abnormal blood pressure; Increased susceptibility to autoimmune diabetes | MP:0002602; MP:0000230; MP:0004803 | immune system hematopoietic cardiovascular homeostasis/metabolism | 3 | 2 | 3 | 2 | immune system hematopoietic |
| IL10 | Il10 | MGI:96537 | Inflammatory bowel disease; Type 1 diabetes | intestinal inflammation; Increased susceptibility to autoimmune diabetes | MP:0001858; MP:0004803 | immune system digestive/alimentary | 2 | 2 | 9 | 2 | immune system digestive/alimentary |
| ABCG2 | Abcg2 | MGI:1347061 | Serum urate/uric acid | abnormal blood uric acid level | MP:0008820 | homeostasis/metabolism | 1 | 2 | 4 | 1 | homeostasis/metabolism |
| APOA5 | Apoa5 | MGI:1913363 | Plasma carotenoid and tocopherol levels |  |  | homeostasis/metabolism | 1 | 2 | 2 | 1 | homeostasis/metabolism |
| APOC1 | Apoc1 | MGI:88053 | Lipid phenotypes | abnormal lipid level | MP:0001547 | homeostasis/metabolism | 1 | 2 | 2 | 1 | homeostasis/metabolism |
| CCR6 | Ccr6 | MGI:1333797 | Inflammatory bowel disease | intestinal inflammation | MP:0001858 | immune system digestive/alimentary | 2 | 2 | 4 | 1 | immune system |
| CD69 | Cd69 | MGI:88343 | Type 1 diabetes | Increased susceptibility to autoimmune diabetes | MP:0004803 | immune system | 1 | 2 | 3 | 1 | immune system |
| EFEMP1 | Efemp1 | MGI:1339998 | Height | abnormal body height | MP:0001253 | growth size | 1 | 2 | 12 | 1 | growth size |
| FCER1A | Fcer1a | MGI:95494 | Serum IgE levels | increased IgE level | MP:0002497 | immune system | 1 | 2 | 4 | 1 | immune system |
| GCKR | Gckr | MGI:1096345 | Lipid phenotypes; CRP concentration | abnormal lipid level; abnormal C-reactive protein physiology | MP:0001547; MP:0002484 | homeostasis/metabolism immune system | 2 | 2 | 1 | 1 | homeostasis/metabolism |
| IFIH1 | Ifih1 | MGI:1918836 | Type 1 diabetes | Increased susceptibility to autoimmune diabetes | MP:0004803 | immune system | 1 | 2 | 4 | 1 | immune system |
| IL23R | Il23r | MGI:2181693 | Inflammatory bowel disease | intestinal inflammation | MP:0001858 | immune system digestive/alimentary | 2 | 2 | 2 | 1 | immune system |
| IRF4 | Irf4 | MGI:1096873 | Chronic lymphocytic leukemia; Skin/hair/eye color related phenotypes | small lymphocytic lymphoma; abnormal coat/hair pigmentation - abnormal skin pigmentation | MP:0009319; MP:0002075 /MP:0002095 | tumorigenesis skin/nails | 2 | 2 | 3 | 1 | tumorigenesis |
| MC4R | Mc4r | MGI:99457 | Obesity related phenotypes | increased body weight | MP:0001260 | growth size | 1 | 2 | 8 | 1 | growth size |
| OPG | Tnfrsf11b | MGI:109587 | Bone mineral density | osteoporosis | MP:0000066 | skeleton homeostasis/metabolism | 2 | 2 | 8 | 1 | skeleton |
| PRKCQ | Prkcq | MGI:97601 | Type 1 diabetes | Increased susceptibility to autoimmune diabetes | MP:0004803 | immune system | 1 | 2 | 2 | 1 | immune system |
| PTPN22 | Ptpn22 | MGI:107170 | Type 1 diabetes; Rheumatoid arthritis | Increased susceptibility to autoimmune diabetes; rheumatoid arthritis | MP:0004803; MP:0003561 | immune system skeleton | 2 | 2 | 8 | 1 | immune system |
| RANKL | Tnfsf11 | MGI:1100089 | Bone mineral density | osteoporosis | MP:0000066 | skeleton homeostasis/metabolism | 2 | 2 | 8 | 1 | skeleton |
| STAT4 | Stat4 | MGI:103062 | Systemic lupus erythematosus | increased susceptibility to systemic lupus erythematosus | MP:0004801 | immune system | 1 | 2 | 5 | 1 | immune system |
| UMOD | Umod | MGI:102674 | Renal function and chronic kidney disease | abnormal kidney physiology | MP:0002136 | renal/urinary | 1 | 2 | 3 | 1 | renal/urinary |
| DNAH11 | Dnahc11 | MGI:1100864 | Lipid phenotypes | abnormal lipid level | MP:0001547 | homeostasis/metabolism | 1 | 2 | 6 | 0 |  |
| IL13 | Il13 | MGI:96541 | Psoriasis | psoriasis | MP:0001193 | skin/nails | 1 | 2 | 4 | 0 |  |
| NCAN | Ncan | MGI:104694 | Lipid phenotypes | abnormal lipid level | MP:0001547 | homeostasis/metabolism | 1 | 2 | 1 | 0 |  |
| IL12B | Il12b | MGI:96540 | Inflammatory bowel disease; Psoriasis | intestinal inflammation; psoriasis | MP:0001858;MP:0001193 | immune system digestive/alimentary skin/nails | 3 | 1 | 6 | 2 | immune system digestive/alimentary |
| MST1 | Mst1 | MGI:96080 | Inflammatory bowel disease | intestinal inflammation | MP:0001858 | immune system digestive/alimentary | 2 | 1 | 3 | 2 | immune system digestive/alimentary |
| PTPN2 | Ptpn22 | MGI:107170 | Type 1 diabetes; Inflammatory bowel disease | Increased susceptibility to autoimmune diabetes; intestinal inflammation | MP:0004803; MP:0001858 | immune system digestive/alimentary | 2 | 1 | 6 | 2 | immune system digestive/alimentary |
| ANK3 | Ank3 | MGI:88026 | Bipolar disorder |  |  | behavior/neurological | 1 | 1 | 3 | 1 | behavior/neurological |
| BACH2 | Bach2 | MGI:894679 | Type 1 diabetes | Increased susceptibility to autoimmune diabetes | MP:0004803 | immune system | 1 | 1 | 2 | 1 | immune system |
| BANK1 | Bank1 | MGI:2442120 | Systemic lupus erythematosus | increased susceptibility to systemic lupus erythematosus | MP:0004801 | immune system | 1 | 1 | 2 | 1 | immune system |
| BMP6 | Bmp6 | MGI:88182 | Height | abnormal body height | MP:0001253 | growth size | 1 | 1 | 3 | 1 | growth size |
| CDH13 | Cdh13 | MGI:99551 | Blood pressure related phenotypes | abnormal blood pressure | MP:0000230 | cardiovascular | 1 | 1 | 3 | 1 | cardiovascular |
| G6PC2 | G6pc2 | MGI:1277193 | Type 2 diabetes | insulin resistance | MP:0005331 | homeostasis/metabolism | 1 | 1 | 1 | 1 | homeostasis/metabolism |
| HLA-DQA1 | H2-Aa | MGI:95895 | Systemic lupus erythematosus; Celiac disease | increased susceptibility to systemic lupus erythematosus | MP:0004801; 0 | immune system digestive/alimentary | 2 | 1 | 2 | 1 | immune system |
| HLA-E | H2-T23 | MGI:95957 | Type 1 diabetes | Increased susceptibility to autoimmune diabetes | MP:0004803 | immune system | 1 | 1 | 2 | 1 | immune system |
| HMGA2 | Hmga2 | MGI:101761 | Height | abnormal body height | MP:0001253 | growth size | 1 | 1 | 5 | 1 | growth size |
| IL2 | Il2 | MGI:96548 | Type 1 diabetes | Increased susceptibility to autoimmune diabetes | MP:0004803 | immune system | 1 | 1 | 13 | 1 | immune system |
| IL27 | Il27 | MGI:2384409 | Type 1 diabetes | Increased susceptibility to autoimmune diabetes | MP:0004803 | immune system | 1 | 1 | 2 | 1 | immune system |
| IL2RA | Il2ra | MGI:96549 | Type 1 diabetes | Increased susceptibility to autoimmune diabetes | MP:0004803 | immune system | 1 | 1 | 5 | 1 | immune system |
| IRGM | Irgm1 | MGI:107567 | Inflammatory bowel disease | intestinal inflammation | MP:0001858 | immune system digestive/alimentary | 2 | 1 | 2 | 1 | immune system |
| KCNH2 | Kcnh2 | MGI:1341722 | QT interval | abnormal QT interval | MP:0003899 | cardiovascular | 1 | 1 | 1 | 1 | cardiovascular |
| KCNJ11 | Kcnj11 | MGI:107501 | Type 2 diabetes | insulin resistance | MP:0005331 | homeostasis/metabolism | 1 | 1 | 3 | 1 | homeostasis/metabolism |
| KITLG | Kitl | MGI:96974 | Skin/hair/eye color related phenotypes | abnormal coat/hair pigmentation - abnormal skin pigmentation | MP:0002075 / MP:0002095 | skin/nails | 1 | 1 | 7 | 1 | skin/nails |
| LINGO1 | Lingo1 | MGI:1915522 | Essential tremor | tremors | MP:0000745 | nervous | 1 | 1 | 1 | 1 | nervous |
| LIPC | Lipc | MGI:96216 | Lipid phenotypes | abnormal lipid level | MP:0001547 | homeostasis/metabolism | 1 | 1 | 3 | 1 | homeostasis/metabolism |
| MAP3K1 | Map3k1 | MGI:1346872 | Breast cancer | mammary gland tumor | MP:0006318 | tumorigenesis | 1 | 1 | 8 | 1 | tumorigenesis |
| MLXIPL | Mlxipl | MGI:1927999 | Lipid phenotypes | abnormal lipid level | MP:0001547 | homeostasis/metabolism | 1 | 1 | 4 | 1 | homeostasis/metabolism |
| PCSK9 | Pcsk9 | MGI:2140260 | Lipid phenotypes | abnormal lipid level | MP:0001547 | homeostasis/metabolism | 1 | 1 | 1 | 1 | homeostasis/metabolism |
| PLEK | Plek | MGI:1860485 | Lipid phenotypes | abnormal lipid level | MP:0001547 | homeostasis/metabolism | 1 | 1 | 2 | 1 | homeostasis/metabolism |
| PLN | Pln | MGI:97622 | QT interval | abnormal QT interval | MP:0003899 | cardiovascular | 1 | 1 | 5 | 1 | cardiovascular |
| PTPRD | Ptprd | MGI:97812 | Restless legs syndrome |  |  | nervous | 1 | 1 | 4 | 1 | nervous |
| RGS1 | Rgs1 | MGI:1354694 | Celiac disease |  |  | digestive/alimentary immune system | 2 | 1 | 2 | 1 | immune system |
| SLC30A8 | Slc30a8 | MGI:2442682 | Type 2 diabetes | insulin resistance | MP:0005331 | homeostasis/metabolism | 1 | 1 | 4 | 1 | homeostasis/metabolism |
| SLCO1B3 | Slco1b2 | MGI:1351899 | Bilirubin levels | abnormal circulating bilirubin level | MP:0001569 | homeostasis/metabolism | 1 | 1 | 3 | 1 | homeostasis/metabolism |
| TNFSF15 | Tnfsf15 | MGI:2180140 | Inflammatory bowel disease | intestinal inflammation | MP:0001858 | immune system digestive/alimentary | 2 | 1 | 2 | 1 | immune system |
| TRAF1 | Traf1 | MGI:101836 | Rheumatoid arthritis | rheumatoid arthritis | MP:0003561 | immune system skeleton | 2 | 1 | 3 | 1 | immune system |
| UBASH3A | Ubash3a | MGI:1926074 | Type 1 diabetes | Increased susceptibility to autoimmune diabetes | MP:0004803 | immune system | 1 | 1 | 2 | 1 | immune system |
| CHI3L1 | Chi3l1 | MGI:1340899 | YKL-40 (chitinase-like protein) concentration |  |  | homeostasis/metabolism | 1 | 1 | 3 | 0 |  |
| DPT | Dpt | MGI:1928392 | Morbidity-free survival | extended life span | MP:0001661 | life span/aging | 1 | 1 | 3 | 0 |  |
| LOXL1 | Loxl1 | MGI:106096 | Exfoliation glaucoma | ocular hypertension | MP:0005258 | vision/eye | 1 | 1 | 4 | 0 |  |
| LRP1B | Lrp1b | MGI:2151136 | Successful cognitive aging |  |  | behavior/neurological | 1 | 1 | 1 | 0 |  |
| LSP1 | Lsp1 | MGI:96832 | Breast cancer | mammary gland tumor | MP:0006318 | tumorigenesis | 1 | 1 | 2 | 0 |  |
| MTNR1B | Mtnr1b | MGI:2181726 | Type 2 diabetes | insulin resistance | MP:0005331 | homeostasis/metabolism | 1 | 1 | 1 | 0 |  |
| PAFAH1B2 | Pafah1b2 | MGI:108415 | Serum markers of iron status | hypoferremia | MP:0004151 | homeostasis/metabolism | 1 | 1 | 3 | 0 |  |
| PAX1 | Pax1 | MGI:97485 | Male-pattern baldness | alopecia | MP:0000414 | skin/nails | 1 | 1 | 2 | 0 |  |
| SLC22A3 | Slc22a3 | MGI:1333817 | Prostate cancer | prostate adenocarcinoma | MP:0009220 | tumorigenesis | 1 | 1 | 1 | 0 |  |
| ABCG5 | Abcg5 | MGI:1351659 | Lipid phenotypes | abnormal lipid level | MP:0001547 | homeostasis/metabolism | 1 | 1 | 0 | 0 |  |
| ARHGEF3 | Arhgef3 | MGI:1918954 | Mean platelet volume | abnormal platelet volume | MP:0002586 | hematopoietic | 1 | 1 | 0 | 0 |  |
| CELSR2 | Celsr2 | MGI:1858235 | Lipid phenotypes | abnormal lipid level | MP:0001547 | homeostasis/metabolism | 1 | 1 | 0 | 0 |  |
| FAM3C | Fam3c | MGI:107892 | Bone mineral density | osteoporosis | MP:0000066 | skeleton homeostasis/metabolism | 2 | 1 | 0 | 0 |  |
| GPR126 | Gpr126 | MGI:1916151 | Height | abnormal body height | MP:0001253 | growth size | 1 | 1 | 0 | 0 |  |
| IL6R | Il6ra | MGI:105304 | IL-6sR concentration | abnormal circulating interleukin-6 level | MP:0008595 | immune system homeostasis/metabolism | 2 | 1 | 0 | 0 |  |
| PLAG1 | Plag1 | MGI:1891916 | Height | abnormal body height | MP:0001253 | growth size | 1 | 1 | 0 | 0 |  |
| PLTP | Pltp | MGI:103151 | Lipid phenotypes | abnormal lipid level | MP:0001547 | homeostasis/metabolism | 1 | 1 | 0 | 0 |  |
| RHPN2 | Rhpn2 | MGI:1289234 | Colorectal cancer | large intestine adenocarcinoma | MP:0009310 | tumorigenesis | 1 | 1 | 0 | 0 |  |
| RNF186 | Rnf186 | MGI:1914075 | Inflammatory bowel disease | intestinal inflammation | MP:0001858 | immune system digestive/alimentary | 2 | 1 | 0 | 0 |  |

MP: mammalian phenotype
